# Mining coral-derived terpene synthases and mechanistic studies of the coral biflorane synthase

**DOI:** 10.1126/sciadv.adv0805

**Published:** 2025-02-26

**Authors:** Bao Chen, Jingjing Mao, Kangwei Xu, Lijun Liu, Wei Lin, Yue-Wei Guo, Ruibo Wu, Chengyuan Wang, Baofu Xu

**Affiliations:** ^1^ Shandong Laboratory of Yantai Drug Discovery, Bohai Rim Advanced Research Institute for Drug Discovery, Yantai, Shandong 264117, China.; ^2^ Shanghai Institute of Materia Medica, Chinese Academy of Sciences, 555 Zu Chong Zhi Road, Zhangjiang Hi-Tech Park, Shanghai 201203, China.; ^3^ Shanghai Institute of Immunity and Infection, Chinese Academy of Sciences, Shanghai 200031, China.; ^4^ Nanjing Drum Tower Hospital, School of Medicine, Nanjing University of Chinese Medicine, Nanjing 210023, China.; ^5^ School of Pharmaceutical Sciences, Sun Yat-sen University, Guangzhou, Guangdong 510006, China.; ^6^ School of Medicine, Shanghai University, Shanghai 200444, China.

## Abstract

Biflorane diterpenoids are unique natural products often seen in marine animals. Recent studies have reported a small number of biflorane synthases. However, the catalytic mechanism and structural basis for biflorane formation remain unclear. To address these issues, we conducted genome mining of terpene synthases from the sea whip coral *Paramuricea clavata*, resulting in the discovery of a biflorane synthase *Pc*TS1. We performed a series of isotope labeling, crystallography, quantum mechanics/molecular mechanics calculations, and mutagenesis studies toward *Pc*TS1 to investigate the mechanism. Isotopic labeling studies, together with calculations, elucidate a cascade of 1,10-cyclization, 1,3-hydride shift, 1,6-cyclization, 1,2-hydride shift, 2,6-cyclization, cyclopropane ring opening, and deprotonation by the generated pyrophosphate, forming the biflorane scaffold. Crystallography, quantum mechanics/molecular mechanics, and mutagenesis studies confirmed the cascade and produced different terpene scaffolds. Our work demonstrated the mechanism of marine biflorane formation, elucidated the second crystal structure of a coral terpene synthase, and realized the terpene skeleton expansion.

## Teaser

Researchers used genome mining to find a coral-derived biforane terpene synthase and conducted an in-depth mechanism study.

## INTRODUCTION

**Fig. 1.**
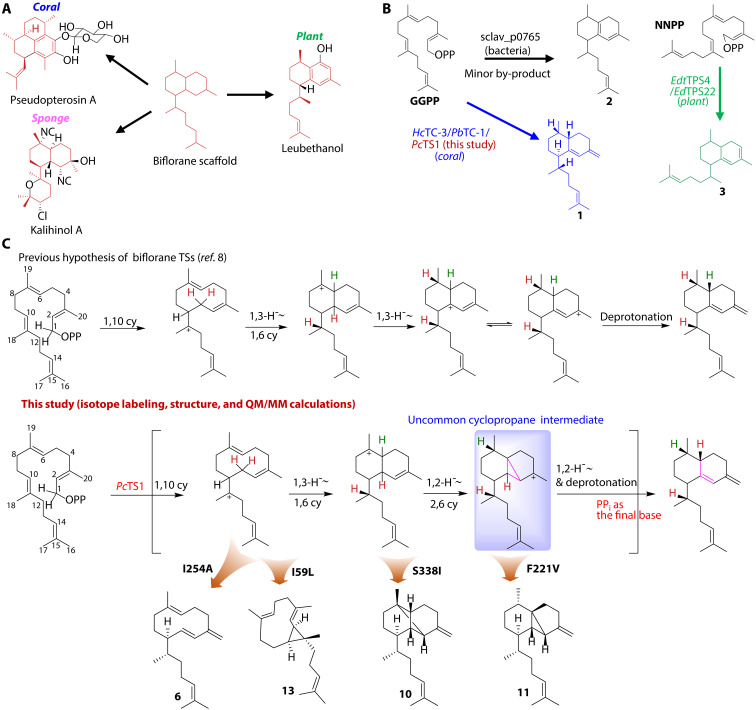
Biflorane (serrulatane) diterpenoids. (**A**) Biflorane framework, along with three representative examples featuring the biflorane skeleton. (**B**) All identified biflorane synthases so far. OPP, pyrophosphate group. (**C**) Mechanism of cyclization from GGPP to biflorane skeleton derivatives, along with characteristic compounds produced by *Pc*TS1 mutants in this study.

The biflorane or serrulatane scaffold is characterized by a structurally unique 6,6-bicyclic framework and a prenyl side chain (Fig. 1A). Biflorane-type diterpenoids are prevalent in corals, sponges, and plants, including pseudopterosin A (*1*), kalihinol A (*2*, *3*), and leubethanol (*4*), from the respective life field (Fig. 1A and fig. S1), with more than 290 representatives having been identified to date (*5*). However, to date, no biflorane natural products have been identified in bacterial sources, while certain nonfunctionalized biflorane diterpenes have been successfully isolated through the enzymatic reaction of bacterial enzymes (*6*–*9*). This family of natural products exhibits diverse biological activities, making them intriguing targets for researchers in the medicinal and total chemical synthesis field. For instance, the coral-derived biflorane compound pseudopterosin A (*1*), with potent anti-inflammatory and analgesic properties, has been successfully applied into the cosmetics industry with a multibillion Euro market value (*10*). To ease the limited supply of pseudopterosin A, a series of total synthesis efforts has been spent. However, no favorable industry-level production yield has been achieved (*11*).

For biosynthesis, only a handful of terpene synthases (TSs) responsible for the formation of biflorane scaffolds have been functionally reported (Fig. 1B) (*6*, *7*, *12*–*15*). The earliest report of biflorane biosynthesis is in 2015 in which Yamada *et al.* (*6*, *7*) examined the biochemical functions of a series of *Streptomyces*-derived TSs by heterologous expression in an engineered *Streptomyces* host. Of the seven expressed TSs, one TS, hydropyrene synthase (*sclav_p0765*), which mainly generates hydropyrene and hydropyrenol from geranylgeranyl diphosphate (GGPP), can also produce isoelisabethatriene as a by-product in a minor production yield (Fig. 1B). Ringel *et al.* (*10*) then applied engineering efforts to hydropyrene synthase, resulting in favorable bioproduction of isoelisabethatrienes A and B in an *Escherichia coli* host, suggesting that finding effective biflorane synthases is of important significance in synthetic biology research of biflorane diterpenoids. In addition to the non-native biflorane synthase mentioned above, several plant- and animal-derived biflorane TSs have been recently found. In 2020, Gericke *et al.* (*12*) identified two *Eremophila* species–derived biflorane synthases, *Edt*TPS4/*Ed*TPS22, which use nerylneryl diphosphate (NNPP) instead of common GGPP as the substrate (Fig. 1B), indicating a divergent biflorane biosynthesis strategy in plants. Scesa *et al.* (*14*) and Burkhardt *et al.* (*15*) characterized coral-derived TSs in 2022, marking a notable advancement in coral terpenoid biosynthesis. Of the reported coral TSs, two of them, *Hc*TC-3 and *Pb*TC-1, can generate elisabethatriene from GGPP (Fig. 1B).

However, the catalytic mechanism for the generation of the marine biflorane scaffold remains unclear. Moreover, until now, only 15 coral-derived TSs have been functionally characterized with solely one of these enzymes, a cembrane synthase, *Er*TC-2 [Protein Data Bank (PDB) ID: 7S5L], having been structurally characterized (*14*). To fill the research gaps of the mechanism and structure in marine biflorane biosynthesis, we performed genome mining and biochemical characterization of sea whip coral–derived TSs, leading to the discovery of one biflorane TS, *Pc*TS1, using GGPP as the substrate. With *Pc*TS1 in hand, we then performed isotope labeling, crystallography, quantum mechanics/molecular mechanics (QM/MM), and mutagenesis studies, enabling us to uncover a cryptic catalytic route very different from the previous hypothesis, featuring one 1,3-hydride shift together with two 1,2-hydride shifts, a cyclopropane containing an intermediate cation and final deprotonation by the pyrophosphate (Fig. 1C). In addition, crystallography and QM/MM-guided mutagenesis enabled us successfully to engineer *Pc*TS1 for producing a variety of different terpene structures, resulting in the expansion of the terpene scaffold space.

## RESULTS

### Finding *Pc*TS1 by functional characterizations of all TSs in a coral

**Fig. 2.**
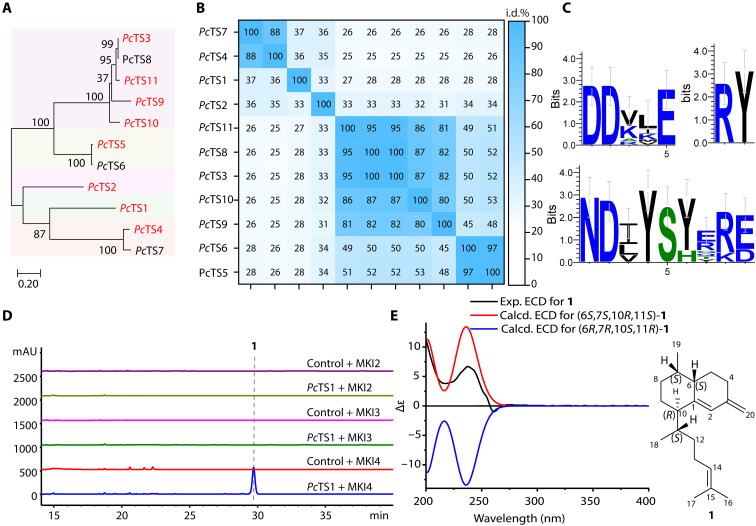
Mining and functional characterizations of TSs from the coral *P. clavata*. (**A**) The phylogenetic analysis of sequences related to TS in *P. clavata* was conducted using the maximum likelihood method. The resulting maximum likelihood–based phylogenetic tree revealed that the 11 identified TS genes were categorized into five branches. TSs colored in red were selected for activity assessment. (**B**) Pairwise identities of 11 TSs found in *P. clavata*. (**C**) Conserved type I TS motifs of 11 TSs in *P. clavata*. (**D**) Functional characterization of *Pc*TS1 using a previously constructed two-step kinase-based C10-to-C20 (MKI2: GPP; MKI3: FPP; MKI4: GGPP) terpene precursor overproduction system (*16*). Extracted metabolites were analyzed through HPLC by monitoring at 210 nm. “Control” shown in the figure refers to the “empty vector pET28a.” (**E**) Comparison of the experimental ECD spectrum (black) and predicted ECD spectra of 6*S*,7*S*,10*R*,11*S*-**1** (red) and 6*R*,7*R*,10*S*,11*R*-**1** (blue). mAU, milli–absorbance unit.

Inspired by the previous reports of coral TSs (*14*, *15*), we performed genome mining of the sea whip coral *Paramuricea clavata*, aiming at finding TSs embedded in the animal genome. In total, 11 TS (Fig. 2A) candidates featuring conserved motifs typical of type I TSs (Fig. 2, B and C, and fig. S2) were categorized into five branches. Subsequently, eight selected representatives were commercially synthesized into the bacterial expression vector pET28a to facilitate the assessment of their activity. With the help of an efficient C10-to-C20 terpene precursor [geranyl diphosphate (GPP), farnesyl diphosphate (FPP), and GGPP] production system (*16*), we identified six functional TSs, with five of them being sesquiterpene synthases producing germacrene B (*17*) or farnesol (*18*) (figs. S3 to S5). The remaining one, *Pc*TS1, showing identities of 68.69 and 97.59% to *Hc*TC-3 and *Pb*TC-1 (*15*), respectively, is a diterpene synthase (Fig. 2D and figs. S3 to S5). Considering that slight natural variations can lead to different terpene scaffold generations (*19*–*23*), we implemented a complete chemical structure analysis process toward the diterpene produced in the GGPP overproduction *E. coli* host harboring the gene encoding *Pc*TS1 (UniProt no. A0A6S7HXL7) to avoid incorrect annotations. Following fermentation of the recombinant bacteria, the major diterpene product (**1**) was purified and structurally elucidated through gas chromatography–mass spectrometry (GC-MS) and one-dimensional (1D) and 2D nuclear magnetic resonance (NMR) analyses (see the Supplementary Materials for detailed planar structure elucidation), resulting in the building of a biflorane skeleton. To determine the absolute stereochemistry of the isolated biflorane product (**1**), we conducted time-dependent density functional theory/electronic circular dichroism (TDDFT/ECD) calculations, confirming the absolute configuration as 6*S*, 7*S*, 10*R*, and 11*S*, identical to elisabethatriene, providing comprehensive evidence and standard spectroscopic data in determining the planar and absolute structure of biflorane scaffolds (Fig. 2E).

### Isotopic labeling experiments reveal the cyclization route by *Pc*TS1

With *Pc*TS1 in hand, we have the opportunity to deeply investigate the missing catalytic mechanisms of the formation of the biflorane scaffold. We first proposed two possible cyclization cascades of *Pc*TS1 (fig. S6). Initially, GGPP is isomerized to geranyllinalyl diphosphate (GLPP) or 2*Z*-GGPP within the enzyme’s pocket, resulting in the formation of a 2*Z* double bond, which is crucial for the subsequent cyclization steps. This isomerization step is consistent with mechanisms observed in other TSs, such as *Cp*PS (*24*) and *Ec*TPS1 (*14*). In Pathway I, a 1,10-cyclization is followed by either a 1,3-hydride shift [previous hypothesis; (*8*)] or two 1,2-hydride shifts, leading to a 1,6-ring closure. This is then followed by another set of hydride shifts (either a 1,3-hydride shift or two 1,2-hydride shifts) to form intermediate carbocation, culminating in deprotonation. Conversely, in Pathway II, the process begins with a 1,6-cyclization, followed by a 1,10 cyclization (fig. S6).

**Fig. 3.**
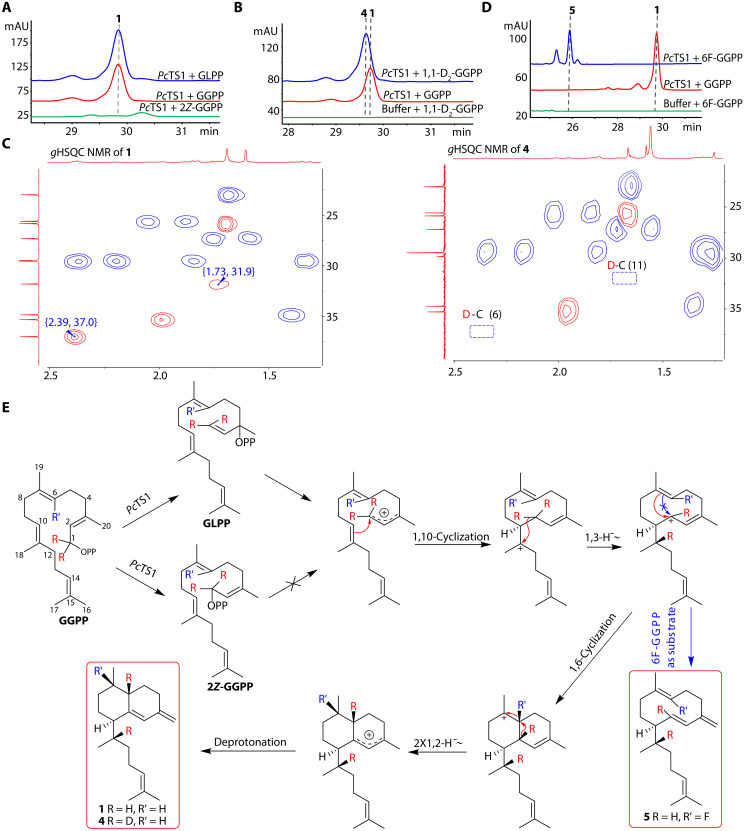
The isotopic labeling experiment reveals the cyclization mechanism of *Pc*TS1. (**A**) Incubation of purified *Pc*TS1 with GLPP yielded **1**, while 2*Z*-GGPP could not produce **1**. (**B**) HPLC analysis of incubation products of *Pc*TS1 with 1,1-D_2_-GGPP indicates the production of deuterium atom–labeled compound **4**. (**C**) HSQC spectra of **1** and **4**. The green dashed box indicates the disappearance of the corresponding HSQC signals in **4**. (**D**) HPLC analysis of incubation products of *Pc*TS1 with 6F-GGPP indicates the production of fluorine atom–labeled compound **5**. (**E**) Proposed cyclization mechanism from GGPP to **1** clarified by isotope labeling studies.

We first investigated the isomerization hypothesis pathway for the formation of the 2*Z* double bond. GGPP, GLPP, and 2*Z*-GGPP were chemically synthesized (fig. S7) (*16*) and incubated with the purified *Pc*TS1 (fig. S8) in vitro. The results revealed that both GGPP and GLPP yielded the same compound **1** (Fig. 3A). However, *Pc*TS1 failed to cyclize 2*Z*-GGPP into compound **1**, in contrast to the mechanism of MicA, which can accept both GLPP and 2*Z*-GGPP as the substrate to produce the 2*Z* form eunicellane compound microeunicellene (*16*). The incapacity of *Pc*TS1 to recognize 2*Z*-GGPP as a substrate might be attributed to its inability to initiate carbocation formation by removing the pyrophosphate group within the catalytic center of *Pc*TS1. Alternatively, 2*Z*-GGPP might not even enter the enzyme pocket because of unsure reasons that need to be further investigated and exceed the scope of this research.

To demonstrate the multiple hydride transfer process, we synthesized 1,1-D_2_-GGPP (Fig. 3B and fig. S7) and incubated it with *Pc*TS1. The deuterated product, compound **4**, was isolated, purified, and characterized using GC-MS and NMR techniques. GC-MS analysis revealed the molecular formula of compound **4** as C_20_H_30_D_2_ (*m/z* 274.3), indicating an increase of 2 Da compared to the unlabeled compound **1**, which corresponds to the replacement of two hydrogen atoms with deuterated atoms in 1,1-D_2_-GGPP (fig. S47). In addition, the NMR comparison between compounds **1** and **4** showed that both share the same biflorane core structure. Also, the absence of correlations for H-6 to C-6 and H-11 to C-11 in the heteronuclear single-quantum coherence (HSQC) spectra of compound **4** (Fig. 3C) indicated the presence of deuterium at the C-6 and C-11 positions. This was also confirmed by the lack of the ^1^H NMR signals for H-6 and H-11 found in compound **1** and the notion that the signal for H-17 in **4** exhibits a singlet instead of a doublet in **1**. In addition, the carbon signals for C-6 and C-11 in compound **4** showed a decrease in intensity, consistent with deuterium isotope effects (*25*, *26*), confirming the presence of deuterium at the C-6 and C-11 positions. Thus, compound **4** was identified as the 6,11-dideuterated derivative of compound **1**. These findings collectively support the proposed cyclization cascades and confirm the occurrence of a 1,3-hydride shift following the 1,10-cyclization and two 1,2-hydride shifts after the 1,6-ring closure in Pathway I or two 1,2-hydride shifts following the 1,6-cyclization and a 1,3-hydride shift after the 1,10-ring closure in Pathway II.

To differentiate the two proposed pathways and provide direct evidence which cyclization process occurs first, we synthesized 6F-GGPP (fig. S9), an excellent substrate for elucidating cyclization pathways (*27*). The fluorine atom at C-6, because of its strong electron-withdrawing effect, inhibits 1,6-cyclization in the catalytic pathway of *Pc*TS1. This inhibition effect is expected to result in the formation of monocyclic products in Pathway I or acyclic products in Pathway II (see hypothesized quenching products in fig. S10). Incubation of 6F-GGPP with *Pc*TS1 followed by high-performance liquid chromatography (HPLC) analysis revealed several different peaks (Fig. 3D), including the main product compound **5**, with a retention time of 25.2 min. This difference in retention time, compared to compound **1** (*t*_R_ = 29.7 min), indicates the formation of the anticipated intermediate shunt fluorinated product. Compound **5** was purified from a 300-ml scale in vitro reaction and characterized using GC-MS and 1D and 2D NMR techniques. Its molecular formula was determined as C_20_H_31_F (*m/z* 290.4) by GC-MS, indicating five degrees of unsaturation. The ^1^H and ^13^C NMR spectra revealed the presence of four methyl groups, six methylene groups, two methine groups, and four double bonds. These features account for four degrees of unsaturation, suggesting that the molecule is monocyclic. Considering the hypothesized shunt products (fig. S10), the analysis of the typical terminal double bond signals and correlations in the ^1^H-^1^H COSY and HMBC spectra not only enabled the determination of two double bonds to be Δ^1,2^ and Δ^3,20^ but also facilitated the establishment of the planar structure of compound **5**. The *E* configuration of the C1/C2 double bond was determined by a large coupling constant (^3^*J*_H-1,H-2_ = 16.3 Hz). The nuclear Overhauser effect correlation between H-10 and H-18 established the trans relative configurations of C-10 and C-11. The isolation and identification of compound **5** confirmed the cyclization order as a 1,10-cyclization followed by a 1,6-ring closure (Fig. 3E).

### Crystal structure of *Pc*TS1

**Fig. 4.**
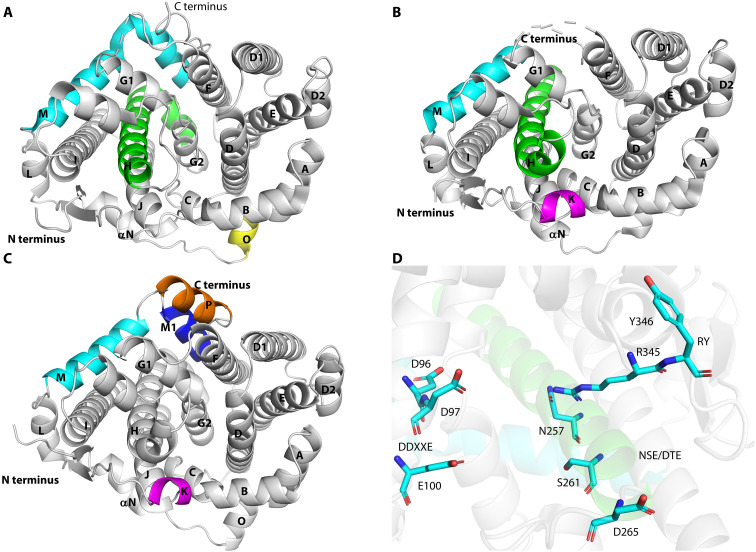
Crystal structure of *Pc*TS1 and structural comparisons. (**A**) Overall structure of *Pc*TS1 (PDB ID: 8ZWZ) showing the typical α-helical fold of type I TCs with two longer helices M (cyan) and H (green), one missing helix K (magenta), and an insertion sequence helix O (yellow) comparing with *Er*TC-2. (**B**) Overall structure of *Er*TC-2 (PDB ID: 7S5L). (**C**) Overall structure of the tFold-generated model of *Pc*TS1 with two more helices K (magenta) and P (brown) comparing with *Pc*TS1. Helix M in the apo form of *Pc*TS1 is split into two helices M (cyan) and M1 (blue). (**D**) Amino acid residues of the conservative region in apo-*Pc*TS1, DDXXE motif on helix D, NSD/DTE motif on helix H, and RY motif on the loop region near helix J.

The structural basis of the cyclization mechanism was studied by determining the crystal structure of *Pc*TS1. Followed by several failure crystallization efforts with full-length *Pc*TS1, we expressed and purified a truncated form of *Pc*TS1, *Pc*TS1^ΔN1–5^, which lacks the first five N-terminal amino acids with catalytic activity unaffected. For clarity, we refer to “*Pc*TS1^ΔN1–5^” as “*Pc*TS1,” unless stated otherwise. We performed crystallization experiments with the purified *Pc*TS1 (fig. S8), enabling us to determine the 3D structure of the apo form of *Pc*TS1 as a monomer at a 3.5 Å resolution (PDB ID: 8ZWZ; Fig. 4A and table S10) using the cembrene A synthase from *Eleutherobia rubra* (PDB ID: 7S5L) as a template (*15*). The crystal structure of apo *Pc*TS1, akin to the previously described coral TS protein *Er*TC-2 (Fig. 4B), exhibits the characteristic class I TS α-helical fold. However, it distinguishes itself with the absence of one helix (K), the elongation of two helices (H and M), and the presence of an additional helix (O) because of an insertion. Notably, *Pc*TS1 shares a remarkable structural resemblance with microbial TS-like proteins, in concert with the similarities observed in *Er*TC-2 (*15*). *Pc*TS1 exhibits an overall structure that closely aligns with the tFold-generated model of *Pc*TS1 (Fig. 4C), with a root-mean-square deviation of 0.94 Å for the Cα atoms. However, the tFold-based model of *Pc*TS1 features two additional helices (K and P) and helix M in the apo form of *Pc*TS1 is split into two helices (M and M1), underscoring the significance of protein crystal structure analysis for achieving more precise outcomes. When compared with TS sequences from a range of species within other coral genera (fig. S12), it is apparent that *Pc*TS1’s aspartate-rich sequence (D^96^D^97^XXE^100^), the NSE(D)/DTE sequence (N^257^DLYS^261^YQRD^265^), and the RY motif (R^345^Y^346^) are conserved across coral species (Fig. 4D).

To further accurately determine the catalytic information inside the center, we performed molecular docking and molecular dynamics (MD) simulation on the basis of the crystal structure. We subsequently used QM/MM calculations to gain catalytic insights.

### QM/MM calculation and mutagenesis studies

**Fig. 5.**
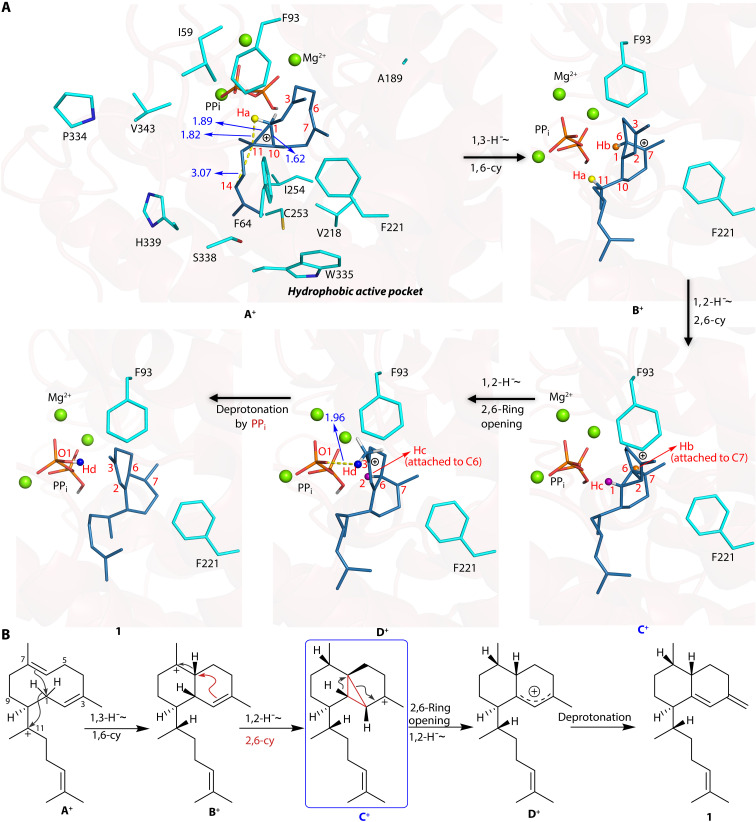
Mechanistic model of initial and intermediate states of *Pc*TS1. The models obtained from QM/MM calculations (**A**) show mechanistic steps corresponding to those in (**B**). A tricyclic **C**^+^ and the final deprotonation base, pyrophosphate, were determined from the current QM/MM experiments.

In the QM/MM calculation (Fig. 5A), the **A**^+^ is observed to bind within a hydrophobic active pocket comprising aromatic residues (F64, F93, F221, W335, and H339), hydrophobic residues (I59, A189, G216, V218, I254, V343, and P344), and polar residues (C253 and S338). Notably, **A**^+^ exhibits a unique structural feature wherein C1, C10, and C11 form a triangular “protonated cyclopropane” configuration, which has been mentioned in previous research (*28*–*32*). The C1-to-C10 and C1-to-C11 distances are 1.62 and 1.89 Å, respectively (fig. S13), which are longer than the typical C─C single bond but shorter than the van der Waals radius of two carbon atoms, suggesting pronounced hyperconjugation effects between the C1─C10 σ bond and the C11’s vacant p-orbital. In addition, **A**^+^ is stabilized by a cation-π interaction between positively charged C11 and Δ^14,15^, with a distance of 3.07 Å (fig. S13). The hydrogen (Ha) on C1 resides above the p-orbital of C11, with a Ha-C11 distance of 1.82 Å, thus facilitating the subsequent 1,3-hydride shift (fig. S13). Following Ha’s shift from C1 to C11, an allylic carbocation forms, leaving C1 and C3 positively charged. Then, C1 moves toward C6 to initiate a 1,6-cyclization, resulting in the formation of **B**^+^. Concurrently, the distance between C11 and Δ^14,15^ elongates to 3.34 Å, as the positive charge shifts to C7 (fig. S13). C7^+^ is stabilized by the nearby aromatic residue F221, with an interaction energy of 4.4 kcal/mol. Herein, the hydride shift and cyclization represent a concerted but asynchronous process, requiring only a slight energy barrier of 1.8 kcal/mol while releasing a substantial heat of 23.5 kcal/mol (fig. S13), making it both kinetically and thermodynamically very feasible. Subsequently, the hydrogen atom (Hb) on C6 transfers to C7, triggering a hybridization transition in C6 from sp3 (tetrahedral) to sp2 (planar) (fig. S14). Simultaneously, as the hydride migrates, C6 gradually approaches the double bond Δ^2,3^, facilitating the formation of a C1-C2-C6 three-membered ring motif, ultimately yielding the **C**^+^ state with a positive charge localized at C3. This **C**^+^ is stabilized by a cation-π interaction with F93, exhibiting an interaction energy of 4.2 kcal/mol. This concerted hydride transfer and cyclization process requires overcoming a slight energy barrier of 2.4 kcal/mol, accompanied by a substantial heat release of 14.6 kcal/mol (fig. S14), presumably stemming from the notion that the enhanced stability achieved the reformation of the three-membered ring motif. It should be noted that C3 bears the most positive charge, while C6 also exhibits a partial charge accumulation, with respective atomic charges of 0.27 and 0.17. As a result, the hydrogen atom (Hc) located at C1 could transfer to C6, thereby destroying the three-membered ring and constructing the 6,6-bicyclic structure, resulting in a **D**^+^ state (Fig. 5). The above transition step needs overcoming a barrier of 11.9 kcal/mol, accompanied by a heat release of 9.9 kcal/mol (fig. S15). In **D**^+^, the vacant p-orbital of C1 conjugates with Δ^2,3^, distributing the positive charge between C1 and C3. While considering an alternative pathway for the formation of **D**^+^, a direct transition from **B**^+^ to **D**^+^ via 1,3-hydride migration (from C1 to C6) seems plausible. However, the hydrogen atoms on C1 and C6 are positioned on opposing sides of the rigid ring in **C**^+^, thereby hindering a direct transfer to C6 because of unfavorable spatial orientation, which is also consistent with above isotope labeling studies that proved that two 1,2-hydride shifts occur during the cyclization process (fig. S14). In **D**^+^, the hydrogen (Hd) positioned on C16 is in close proximity to the oxygen atom (O1) of OPP, with a distance of 1.96 Å (fig. S16); accordingly, the basic O1 abstracts Hd, generating the ultimate product **1** (Fig. 5), indicating that the leaving pyrophosphate group is the final deprotonation base. This transformation involves a tiny barrier of 1.3 kcal/mol and exhibits a notable exothermicity of 20.5 kcal/mol (fig. S16).

**Fig. 6.**
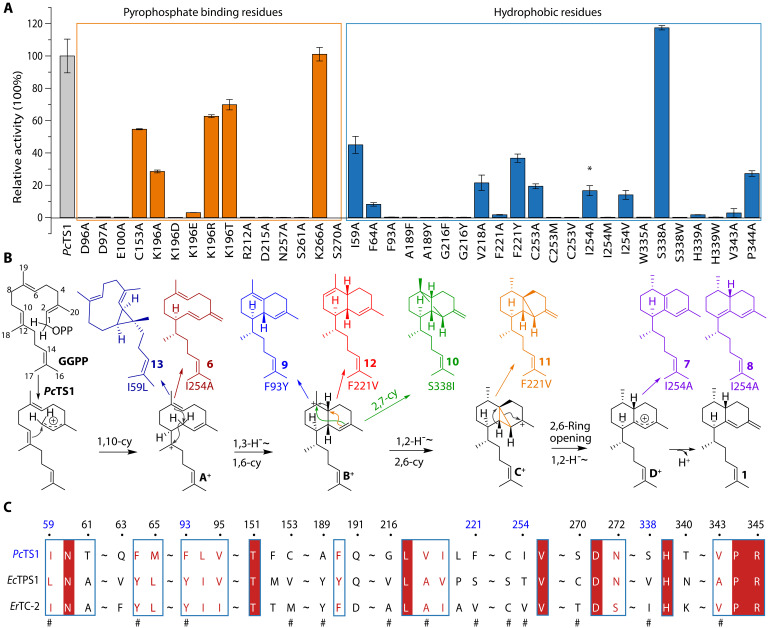
Mutation analysis of *Pc*TS1. (**A**) Relative enzyme activities of *Pc*TS1 and its site-specific mutants. Three biological parallel (*n* = 3) replicates were performed for both wild-type *Pc*TS1 and its mutants. The data are presented as mean values and the standard error of the mean. The asterisk (*) indicates that the mutant can make different products. (**B**) Reaction mechanisms for *Pc*TS1 and the proposed mechanisms for other products generated by indicated *Pc*TS1 mutants *Pc*TS1^I59L^, *Pc*TS1^F93Y^, *Pc*TS1^F221V^, *Pc*TS1^I254A^, and *Pc*TS1^S338I^. (**C**) Alignment of key amino acid residues (# tag) of TSs *Pc*TS1, *Er*TC-2, and *Ec*TPS1.

We next implemented mutational studies (Fig. 6) on the basis of the structural information from crystal experiments and mechanism insights from QM/MM calculations. We first mutated residues interacting with the pyrophosphate group, including C153, K196, R212, D215, S270, and K266. Notably, R212, D215, and S270 were identified as critical for activity. Notably, mutating K196 to A, R, or T partially restored activity, indicating the influence of the amino acid’s acidity on its interaction with the pyrophosphate group, whereas mutations to D or E were ineffective. Subsequently, residues in the hydrophobic active pocket, including I59, F64, F93, A189, G216, V218, F221, C253, I254, W335, S338, H339, V343, and P344, as depicted in Fig. 6A, were investigated. Alanine scanning–based mutations on these residues underscored their significance in biflorane scaffold formation. The *Pc*TS1^I254A^ mutant displayed a distinct product profile from the wild-type *Pc*TS1 (Fig. 6B), with compound **6** identified as an intermediate of 1,10-cyclization. Compounds **7** and **8** were identified as different shunt products compared to **1**, with **7** formed by deprotonation at C6 of **D**^+^ and **8** formed by deprotonation at C10 of **D**^+^. This observation suggests that the enlargement of the active pocket volume, caused by the introduction of alanine, may provide the intermediate with a larger active space, thereby allowing more flexibility in the deprotonation process.

Considering that natural variation–aided engineering has more chances of gaining additional functions of enzymes (*19*), we performed further mutagenesis efforts on the basis of phylogenetics and alignment analysis with several coral TSs with more than 40% identities (Fig. 6C and figs. S12 and S19), resulting in the generations of several structurally unique terpene scaffolds that can link to the carbon cation intermediates we proposed from isotopic labeling and QM/MM experiments. For instance, *Pc*TS1^F93Y^ yielded product **9**, probably generated from **B**^+^ (Fig. 5 and fig. S21). *Pc*TS1^S338I^ produced a cryptic cage structure scaffold **10**, a further cyclized product from **B**^+^ (Fig. 5 and fig. S21). In addition, *Pc*TS1^F221V^ produced two compounds: **11**, a 2,6-cyclization product generated from **C**^+^, and **12**, a different deprotonation product similar to **9**, generated from **B**^+^ (Fig. 5 and fig. S21). Moreover, *Pc*TS1^I59L^ produced a cyclopropane-containing product **13**, generated from **A**^+^. Compound **13** shares a cyclopropane substructure with a minor product produced by spata-13,17-diene synthase when incubated with GGPP (*33*).

## DISCUSSION

In conclusion, we have provided a comprehensive explanation of the mechanism underlying the generation of the biflorane scaffold in corals by implementing a series of isotope labeling, structure, chemical calculations, and mutagenesis experiments toward the biflorane TS *Pc*TS1. Our work clearly demonstrates that the catalytic cascade involves both 1,3- and 1,2-hydride shifts and a cryptic cyclopropane carbon cation. In addition, the final deprotonation base is also identified to be the generated pyrophosphate staying inside the pocket of *Pc*TS1 with the aid of QM/MM calculations. We lastly verified the catalytic mechanism by mutating critical residues pinpointed by structure and QM/MM studies, leading to the production of additional terpene scaffolds. Notably, *Pc*TS1^S338I^ produced a cage structure scaffold **10**. Cage structure–containing natural terpenoids often exhibit attractive biological activities, such as coral-derived suberosenol B (*34*), plant-derived *Daphniphyllum* triterpene alkaloids (*35*), and fungus-derived vinigrol (*36*). Future related bioproduction enhancement, activity evaluations, and structure modifications will be of great interest to be performed. In addition, *Pc*TS1^I59L^ and *Pc*TS1^F221V^ can synthesize cyclopropane-containing terpene scaffolds. The cyclopropane group is often introduced into drugs to improve potency and decrease toxicity because of its important features, including coplanarity and shorter C─C bonds (*37*). The directly synthesized cyclopropane-containing terpene scaffold using engineered TSs would provide an opportunity for easily producing potential drug candidates with a cyclopropane core.

In contrast to marine animals, land plants take advantage of a divergent strategy for generating a biflorane scaffold. In plants, biflorane synthases, *Edt*TPS4/*Ed*TPS22, accept NNPP instead of GGPP as the substrate (*12*). This might lead to different cyclization mechanisms among different life fields, which need to be further investigated in the future. In addition, whether biflorane TSs from other plant species can also use the common GGPP as the substrate remains to be investigated. In addition, no bacterial biflorane diterpenoids have been reported from isolation efforts. Whether the bacteria do not have biflorane synthases evolutionarily or the bacterial biflorane genetic machineries are not working under common laboratory conditions remains to be investigated.

In addition, more than half a million marine animal species worldwide present considerable opportunities for marine drug discovery through genome mining (*38*), yet the scarcity of high-quality genomic sequences for these species poses a major challenge, with less than 500 genomes of low-to-medium quality available in public databases (*39*). This limitation hinders the biosynthesis-guided exploration of natural products derived from marine animals. The collection and sharing of high-quality genomic data on marine animals will be crucial for enabling sustainable chemical research on marine chemodiversity in future studies.

## MATERIALS AND METHODS

### Instruments and reagents

Reversed-phase HPLC was conducted on an Agilent 1260 Infinity LC equipped with an Agilent Zorbax SB-C18 column (4.6 mm by 150 mm, 5 μm). Preparative HPLC was performed on an Agilent 1260 Infinity LC using an Agilent Zorbax SB-C18 column (21.2 mm by 250 mm, 7 μm). The NMR spectra were acquired using a Bruker 600 spectrometer (Bruker Biospin AG, Fällanden, Germany). GC-MS analyses were conducted using an 8890 GC system (Agilent Technologies, Shanghai, China) equipped with a 7650 autosampler (Agilent Technologies, China) and coupled to a 5977C mass spectrometric detector (Agilent Technologies, DE, US). Commercial silica gel (200 to 300 and 300 to 400 mesh, Yantai Xinnuo Chemical Co., Ltd., Yantai, China) was used for column chromatography (CC). All solvents used for CC and HPLC were of analytical grade (Shanghai Chemical Reagents Co., Ltd.) and chromatographic grade (Dikma Technologies Inc., CA, US), respectively. The enzymes necessary for the cloning were obtained from TransGen Biotech. Isopropyl-β-d-thiogalactopyranoside (IPTG) and isoprenol were procured from BBI Life Sciences Corporation and Sigma-Aldrich, respectively. Reagents used for chemical synthesis were procured from Shanghai Macklin Biochemical Technology Co., Ltd.

### Mining and functional characterizations of TSs

The isoprenoid synthase superfamily (InterPro no. IPR008949) was queried against the UniProt database, yielding more than 200,000 matches. These results were refined by taxonomy (*P. clavata*) to obtain 23 relevant matches. The sequences associated with terpene biosynthesis in *P. clavata* underwent phylogenetic analysis with the maximum likelihood method and were annotated using the Family & Domains databases within UniProt (Fig. 2A). Besides the short sequences and polyprenyl synthase related to the synthesis of terpene diphosphate precursors, the 11 identified TS genes, featuring conserved motifs typical of type I TSs (Fig. 2, B and C, and fig. S2), were categorized into five branches (Fig. 2A). Subsequently, eight selected representatives were commercially synthesized into the bacterial expression vector pET28a to facilitate the assessment of their activity. Activity evaluations were carried out by coexpressing the constructed vectors with an in vivo genetic system to facilitate the efficient synthesis of C10-to-C20 terpene precursors (MKI2: GPP; MKI3: FPP; MKI4: GGPP) in *E. coli* (*16*). “MKI” expresses three genes that convert isoprenol into DMAPP, where “MKI” is an abbreviation for these three genes. “M” refers to a kinase, hydroxyethylthiazole kinase (ThiM) from *E. coli*; “K” refers to a kinase, isopentenyl phosphate kinase (ipk) from *Arabidopsis thaliana*; and “I” refers to isopentenyl diphosphate isomerase (idi) from *E. coli*. “MKI4” encodes for four genes responsible for converting isoprenol into GGPP, with “MKI” denoting an abbreviation for the three genes previously mentioned. The “4” signifies the capability of the recombinant strain to produce four times the isoprene units. In another word, a GGPP synthase (bnd3) has been introduced in the “MKI” system for generating the C20 polyprenyl pyrophosphate substrate GGPP. Similarly, the GPP synthase and FPP synthase have been introduced in the “MKI” system to form the MKI2 and MKI3 vectors for generating the C10 polyprenyl pyrophosphate substrate GPP and the C15 polyprenyl pyrophosphate substrate FPP. Following fermentation of the recombinant bacteria, the products were analyzed using HPLC (figs. S3 to S5). Major product fractions presenting previously unobserved HPLC peaks were purified via large-scale fermentations and structurally elucidated through GC-MS and 1D and 2D NMR analyses (see the Supplementary Materials for detailed planar structure elucidation).

### Protein expression and purification

The plasmid carrying the *Pc*TS1 gene was introduced into *E. coli* BL21Gold(DE3) for expression. The transformed *E. coli* strain was cultured in lysogeny broth (LB) medium supplemented with kanamycin (50 mg/ml) for antibiotic selection. Cultures were set up in four flasks, each containing 1 liter of LB, and incubated at 37°C with continuous shaking at 220 rpm until the optical density at 600 nm (OD_600_) reached 0.8. Gene expression was induced by adding IPTG at a final concentration of 0.35 mM. The cultures were then incubated at 16°C for 16 hours with shaking at 200 rpm. Following cultivation, the cells were harvested by centrifugation at 4000 rpm for 10 min at 4°C. The cell pellet was resuspended in a cold lysis buffer (50 mM tris-HCl, pH 8.0, with 150 mM NaCl). The cells were then disrupted using a High-Pressure Homogenizer (Scientz) and subsequently centrifuged at 18,000*g* for 40 min at 4°C. The supernatant containing the target proteins underwent nickel affinity chromatography, being loaded onto a column packed with Ni Sepharose 6 Fast Flow (Cytiva). The column was washed with lysis buffer containing 20 mM imidazole and eluted with lysis buffer containing 500 mM imidazole. The purified proteins were promptly desalted using a PD-10 column (Cytiva), then rapidly frozen in liquid nitrogen, and stored at −80°C until required. Protein purity was evaluated using SDS–polyacrylamide gel electrophoresis.

### Synthesis of 6F-GGPP

#### (2Z,6E)-2-Fluoro-3,7,11-trimethyldodeca-2,6,10-trien-1-ol

See full compound schemes in the Supplementary Materials. The procedure reported by Jin *et al.* (*27*) was followed with certain modifications. To a cooled (0°C) and well-stirred suspension of NaH (1.8 g, 60% dispersion in mineral oil, 45.3 mmol) in dry tetrahydrofuran (THF; 80 ml) was added triethyl 2-fluoro-2-phosphonoacetate (9.2 ml, 45.3 mmol) slowly. Geranylacetone (4.4 ml, 19.8 mmol) in THF (10 ml) was added to the resulting yellow solution. The mixture was stirred for 2.5 hours at room temperature and poured into cold water (100 ml). The organic layer was separated, and the aqueous layer was extracted with Et_2_O (3 × 100 ml). The combined organic extracts were washed with water (2 × 200 ml) and brine (2 × 200 ml) and dried over MgSO_4_. Evaporation of the solvent afforded the fluoro ester, which was purified by flash CC on silica gel with petroleum ether (PE)/ethyl acetate (EA) (200:1) to yield 4.8 g of pure fluoro ester (17.0 mmol, 87%) as mixtures of 2*E* and 2*Z* diastereomers (1:1). The ester (4.8 g, 17.0 mmol) in THF (80 ml) was stirred and cooled at 0°C as LiAlH_4_ (1.2 g, 31.6 mmol) was added in one portion. The suspension was stirred at room temperature for 2 hours and quenched by the addition of 1.3 ml of water dropwise, 1.3 ml of 15% NaOH, and 3.9 ml of water. The mixture was extracted three times with Et_2_O. The combined organic layers were dried with MgSO_4_ and concentrated under reduced pressure. The residue was purified by CC on silica gel with PE/EA (20:1) to give (2*Z*,6*E*)-2-fluoro-3,7,11-trimethyldodeca-2,6,10-trien-1-ol [1.5 g, 6.3 mmol, 37%, TLC *R*_f_ 0.43 (20% EA in PE)] followed by (2*E*,6*E*)-2-fluoro-3,7,11-trimethyldodeca-2,6,10-trien-1-ol). ^1^H NMR (CDCl_3_, 600 MHz) of (2*Z*,6*E*)-2-fluoro-3,7,11-trimethyldodeca-2,6,10-trien-1-ol: δ 1.60 (brs, 6H, CH_3_), 1.67 (m, 6H, CH_3_), 1.95 to 2.14 (m, 8H, CH_2_), 4.23 (dd, 2H, *J* = 22.3, 3.5 Hz, CH_2_OH), 5.10 (m, 2H, vinyl H).

#### (2Z)-1-Bromo-2-fluoro-3,7,11-trimethyldodeca-2,6,10-triene

A solution of alcohol (1.4 g, 5.8 mmol) in dry DCM (30 ml) was stirred and cooled to 0°C. CBr_4_ (1.5 equiv, 2.9 g, 8.8 mmol) was added, and then 2.3 g of PPh_3_ (8.8 mmol) was added slowly. The suspension was stirred at room temperature for 2.5 hours and concentrated under reduced pressure, at which time the reaction was judged complete by TLC analysis. The residue was purified by CC on silica gel with PE to give (2*Z*)-1-bromo-2-fluoro-3,7,11-trimethyldodeca-2,6,10-triene (1.2 g, 4.0 mmol, 69%). ^1^H NMR (CDCl_3_, 600 MHz) of (2*Z*)-1-bromo-2-fluoro-3,7,11-trimethyldodeca-2,6,10-triene: 1.61 (brs, 6H, CH_3_), 1.69 (m, 6H, CH_3_), 1.95 to 2.18 (m, 8H, CH_2_), 4.07 (d, 2H, *J* = 22.9 Hz, CH_2_Br), 5.10 (m, 2H, vinyl H).

#### (6Z,10E)-6-Fluoro-7,11,15-trimethyl-3-oxohexadeca-6,10,14-trienoic acid, ethyl ester

The procedure reported by Jin *et al.* (*27*) was followed. To a suspension of NaH (528.0 mg, 60% dispersion in mineral oil, 13.2 mmol) in dry THF (30 ml) was added ethyl acetoacetate (1.5 ml, 11.8 mmol) dropwise at 0°C. After 10 min, *n*-BuLi (1.6 M in hexane, 7.9 ml, 12.6 mmol) was added slowly over 5 min. It was stirred for an additional 10 min at 0°C, as (2*Z*)-1-bromo-2-fluoro-3,7,11-trimethyldodeca-2,6,10-triene (1.2 g, 4.0 mmol) was added. After 15 min, the TLC analysis showed that the reaction was complete and HCl (3 M, 6 ml) was added. Water (50 ml) was added, and the organic layer was separated. The aqueous layer was extracted with Et_2_O (3 × 50 ml). The combined organic layers were washed with water (2 × 100 ml) and saturated NaCl solution (1 × 100 ml) and then dried over Na_2_SO_4_. The removal of the solvent gave the crude product, and purification by CC using PE/EA (10:1) as the eluting solvent gave (6*Z*,10*E*)-6-fluoro-7,11,15-trimethyl-3-oxohexadeca-6,10,14-trienoic acid, ethyl ester (0.8 g, 2.3 mmol, 57%). ^1^H NMR (CDCl_3_, 600 MHz) of (6*Z*,10*E*)-6-fluoro-7,11,15-trimethyl-3-oxohexadeca-6,10,14-trienoic acid, ethyl ester: 1.26 (t, 3H, *J* = 7.1 Hz, CH_3_), 1.58 (m, 9H, CH_3_), 1.66 (d, 3H, *J* = 1.3 Hz, CH_3_), 1.92 to 2.07 (m, 8H, CH_2_), 2.51 (dt, 2H, *J* = 22.1, 7.4 Hz, CH_2_), 2.72 (t, 2H, *J* = 7.7 Hz, CH_2_), 3.42 (s, 2H, CH_2_), 4.18 (q, 2H, *J* = 7.1 Hz, CH_2_), 5.08 (m, 2H, vinyl H).

#### (2Z,6Z,10E)-3-(Diisopropoxyphosphoryloxy)-6-fluoro-7,11,15-trimethylhexadeca-2,6,10,14-tetraenoic acid, ethyl ester

The enol phosphorylation procedure by Jin *et al.* (*27*) was followed to convert (6*Z*,10*E*)-6-fluoro-7,11,15-trimethyl-3-oxohexadeca-6,10,14-trienoic acid, ethyl ester to the enol phosphate with certain modifications. A suspension of NaH (110.4 mg, 2.8 mmol, 60% in mineral oil) in Et_2_O (40 ml) was stirred and cooled to 0°C as a solution of (6*Z*,10*E*)-6-fluoro-7,11,15-trimethyl-3-oxohexadeca-6,10,14-trienoic acid, ethyl ester (0.8 g, 2.3 mmol) in Et_2_O (5 ml) was added slowly. The mixture was then stirred at 0°C for 30 min until the bubbling stopped. Diisopropyl chlorophosphate (682.0 mg, 3.4 mmol) was added slowly, and stirring was continued at 0°C for an additional 15 min at which time TLC analysis showed that all the substrate was consumed. The reaction was quenched by adding saturated NH_4_Cl solution (30 ml). The mixture was diluted with water (20 ml), and the organic layer was separated. The aqueous layer was extracted with Et_2_O (3 × 50 ml). The combined ethereal extracts were washed with water (2 × 100 ml), dried over MgSO_4_, concentrated to give a light yellow oil, and purified by CC on silica gel with PE/EA (10:3) to afford (2*Z*,6*Z*,10*E*)-3-(diisopropoxyphosphoryloxy)-6-fluoro-7,11,15-trimethylhexadeca-2,6,10,14-tetraenoic acid, ethyl ester (1.2 g, 2.1 mmol, 91%). ^1^H NMR (CDCl_3_, 600 MHz) of (2*Z*,6*Z*,10*E*)-3-(diisopropoxyphosphoryloxy)-6-fluoro-7,11,15-trimethylhexadeca-2,6,10,14-tetraenoic acid, ethyl ester: 1.25 (t, 3H, *J* = 7.1 Hz, CH_3_), 1.35 (d, 12H, *J* = 6.1 Hz, CH_3_), 1.59 (m, 9H, CH_3_), 1.67 (d, 3H, *J* = 1.2 Hz, CH_3_), 1.93–2.09 (m, 8H, CH_2_), 2.48 to 2.63 (m, 4H, CH_2_), 4.14 (q, 2H, *J* = 7.1 Hz, CH_2_), 4.82 (m, 2H, CH), 5.08 (m, 2H, vinyl H), 5.33 (s, 1H, vinyl H).

#### (2E/Z,6Z,10E)-6-Fluoro-3,7,11,15-tetramethylhexadeca-2,6,10,14-tetraenoic acid, ethyl ester

The following procedure is similar to the one reported by Jin *et al.* (*27*) with certain modifications. A suspension of (2*Z*,6*Z*,10*E*)-3-(diisopropoxyphosphoryloxy)-6-fluoro-7,11,15-trimethylhexadeca-2,6,10,14-tetraenoic acid, ethyl ester (1.2 g, 2.1 mmol) in Et_2_O (30 ml) was stirred and cooled at −78°C as Me_2_CuLi (8.4 ml, 4.2 mmol, 0.5 M in Et_2_O) was added. The resulting yellow solution was stirred for 3.5 hours at −78°C. MeI (3.0 g, 21.1 mmol) was added, and the mixture was stirred for 15 min at −78°C. The mixture was poured into an ice-cooled mixture of saturated NH_4_Cl solution and concentrated NH_4_OH [50 ml; 1:1 (v/v) mixture]. The product was extracted with Et_2_O (3 × 50 ml). The combined organic layers were washed with water (3 × 100 ml), dried over Na_2_SO_4_, concentrated, and purified by CC with PE/EA (10:1) to give a mixture of *trans*/*cis* isomers (573.8 mg, 1.6 mmol, 76%). ^1^H NMR (CDCl_3_, 600 MHz) of the mixture: 1.23 (t, 3H, *J* = 7.1 Hz, CH_3_), 1.53 (t, 3H, *J* = 2.3 Hz, CH_3_), 1.56 (d, 6H, *J* = 4.8 Hz, CH_3_), 1.64 (s, 3H, CH_3_), 1.91 to 2.07 (m, 8H, CH_2_), 2.14 (d, 3H, *J* = 1.3 Hz, CH_3_), 2.24 to 2.41 (m, 4H, CH_2_), 4.10 (q, 2H, *J* = 7.1 Hz, CH_2_), 5.07 (m, 2H, vinyl H), 5.64 (p, 1H, *J* = 1.2 Hz, vinyl H).

#### (2E,6Z,10E)-6-Fluoro-3,7,11,15-tetramethylhexadeca-2,6,10,14-tetraen-1-ol

(2*E*/*Z*,6*Z*,10*E*)-6-Fluoro-3,7,11,15-tetramethylhexadeca-2,6,10,14-tetraenoic acid, ethyl ester (573.8 mg, 1.6 mmol) was dissolved in 30 ml of dry Et_2_O and cooled to 0°C. After the addition of diisobutylaluminium hydride (1 M in hexane, 3.5 ml, 3.5 mmol), the reaction mixture was stirred for 2 hours. The reaction was quenched by adding 0.2 ml of water dropwise, 0.2 ml of 15% NaOH, and 0.6 ml of water. The mixture was extracted three times with Et_2_O. The combined organic layers were dried with MgSO_4_ and concentrated under reduced pressure. The residue was purified by CC on silica gel with PE/EA (20:1) to give (2*E*,6*Z*,10*E*)-6-fluoro-3,7,11,15-tetramethylhexadeca-2,6,10,14-tetraen-1-ol (400.6 mg, 1.3 mmol, 81%). ^1^H NMR (CDCl_3_, 600 MHz) of (2*E*,6*Z*,10*E*)-6-fluoro-3,7,11,15-tetramethylhexadeca-2,6,10,14-tetraen-1-ol: δ 1.53 (t, 3H, *J* = 2.5 Hz, CH_3_), 1.58 (d, 6H, *J* = 4.5 Hz, CH_3_), 1.66 (s, 6H, CH_3_), 1.91 to 2.07 (m, 8H, CH_2_), 2.13 to 2.19 (m, 2H, CH_2_), 2.24 to 2.36 (m, 8H, CH_2_), 4.10 (d, 2H, *J* = 6.9 Hz, CH_2_), 5.08 (m, 2H, vinyl H), 5.39 (td, 1H, *J* = 6.9, 1.6 Hz, vinyl H).

#### (2E,6Z,10E)-1-Chloro-6-fluoro-3,7,11,15-tetramethylhexadeca-2,6,10,14-tetraene

Conditions for the following reaction were based on those described by Cane *et al.* (*40*), A solution of (2*E*,6*Z*,10*E*)-6-fluoro-3,7,11,15-tetramethylhexadeca-2,6,10,14-tetraen-1-ol (400.6 mg, 1.3 mmol) and triphenylphosphine (681.2 mg, 2.6 mol) in 5 ml of carbon tetrachloride was heated at reflux at 84°C for 12 hours. The solution was cooled and diluted with pentane (50 ml). The triphenylphosphine oxide precipitated from the solution was removed by vacuum filtration through a fritted-glass funnel. Most of the solvent was removed with a rotary evaporator at aspiratory pressure. The last traces of solvent were removed by pumping at a high vacuum for 1.5 hours. The resulting pale-yellow oil (381.4 mg, 1.2 mmol, 92%) was used directly in the next step.

#### 6F-GGPP

Woodside *et al.*’s procedure (*41*) was used to prepare 6F-GGPP.Tris(tetrabutylammonium) hydrogen pyrophosphate trihydrate [(Bu_4_N)_3_P_2_O_7_H] was prepared as follows. Disodium dihydrogen pyrophosphate (3.1 g, 14 mmol) was dissolved in 25 ml of 4% (v/v) aqueous ammonium hydroxide. The clear solution was loaded onto a 2- by 30-cm column of Dowex AG 50W-X8 cation exchange resin (100 to 200 mesh, H^+^ form), which had been prewashed with deionized water. The free acid was eluted with 150 ml of deionized water, and the eluent was immediately titrated to pH 7.3 with 25% (w/w) aqueous tetrabutylammonium hydroxide (about 15 ml). The resulting solution (~190-ml total volume) was dried by freezing the solution in a dry ice isopropanol bath and lyophilizing for ~2 days to yield a hygroscopic white solid (9.8 g, 77%), which was stored over phosphorus pentoxide until required.

A mixture of (2*E*,6*Z*,10*E*)-1-chloro-6-fluoro-3,7,11,15-tetra-methylhexadeca-2,6,10,14-tetraene (381.4 mg, 1.2 mmol), (Bu_4_N)_3_P_2_O_7_H (3.0 g, 3.1 mmol), and acetonitrile (5 ml) was stirred at room temperature for 2 hours. The solvent was then removed with a rotary evaporator using a 40°C water bath to give a yellow oil, which was dissolved in ion-exchange buffer (1:49 *i*-PrOH:25 mM NH_4_HCO_3_), and the aqueous phase was washed with Et_2_O (2 × 4 ml), collected, and loaded onto a 4- by 15-cm column of Dowex AG 50W-X8 cation exchange resin (100 to 200 mesh, NH_4_^+^ form). The column was eluted with 360 ml (two column volumes) of ion-exchange buffer, and the fractions containing 6F-GGPP (fractions that stained purple on silica TLC plates with vanillin-sulfuric acid) were lyophilized to give a white solid (362.6 mg, 0.7 mmol, 58%) containing inorganic phosphate and probably some NH_4_HCO_3_. ^1^H NMR (CD_3_OD, 600 MHz) of 6F-GGPP: δ 1.59 (t, 3H, *J* = 2.8 Hz, CH_3_), 1.61 (d, 6H, *J* = 8.1 Hz, CH_3_), 1.67 (d, 3H, *J* = 4.6 Hz, CH_3_), 1.72 (s, 3H, CH_3_), 1.95 to 2.10 (m, 8H, CH_2_), 2.15 to 2.20 (m, 2H, CH_2_), 2.30 to 2.38 (m, 2H, CH_2_), 4.53 (t, 2H, *J* = 6.5 Hz, CH_2_), 5.11 (m, 2H, vinyl H), 5.46 (m, vinyl H).

### In vitro enzymatic assays of *Pc*TS1

The assays were conducted in a 100-μl mixture containing 50 mM tris-HCl buffer (pH 8.0), 15 mM MgCl_2_, 20 μM *Pc*TS1 enzyme, and 5 mM GGPP. The reaction was initiated by adding the enzyme and incubated for 3 hours at 37°C. To quench the reaction, 200 μl of ice-cold acetonitrile and 50 μl of saturated NaCl solution were added sequentially to induce phase separation. The upper organic layer was then collected for HPLC analysis. Chromatographic separation was achieved at 35°C and a flow rate of 1 ml/min using a 40-min solvent gradient system from 5 to 95% CH_3_CN in water, detailed as follows: 0 to 3 min at 5% CH_3_CN, 3- to 18-min ramping to 95% CH_3_CN, and maintaining 95% CH_3_CN in 18 to 40 min. The enzyme products were detected at 210 nm with a photodiode array detector. Similar assays were performed using 2*Z*-GGPP, GLPP, 1,1-D_2_-GGPP, or 6F-GGPP as substrates.

### Isolation of compounds **1** to **13**

*E. coli* BL21Gold(DE3) was used to express the plasmids containing CDF-MKI4 (see table S2 for more details) and pET28a-*Pc*TS1 and was cultured in 500 ml of LB supplemented with kanamycin (50 mg/liter) and streptomycin (50 mg/liter). After overnight incubation, 10-ml cultures were inoculated into 30× 1-liter flasks of fresh LB medium. When OD_600_ reached 1.5, IPTG (0.5 mM) and isoprenol (1.0 mM) were added. The cultures were then further incubated at 28°C and 220 rpm for an additional 18 hours. Subsequently, the cultures were centrifuged at 4000 rpm for 15 min, and the resulting pellet was extracted with methanol (MeOH). The organic extract was then partitioned with PE. The resulting PE solution was concentrated under reduced pressure, subjected to silica gel CC (200 to 300 mesh), and eluted with PE to obtain compound **1**. The isolation and purification of compounds germacrene B and **6** to **13** followed a similar procedure to the previous steps, with variations in the plasmids used. After purification on a silica gel column, compounds **6** to **13** underwent further purification using preparative HPLC. This involved a gradient elution program of acetonitrile (CH_3_CN) and water as the mobile phase (0 to 3 min, 5% CH_3_CN; 3 to 18 min, 5 to 95% CH_3_CN; 18 to 60 min, 95% CH_3_CN) at a flow rate of 20 ml/min using an Agilent Zorbax SB-C18 column (21.2 mm by 250 mm, 7 μm). For farnesol, after the MeOH extraction of bacterial cells, the resulting MeOH solution was concentrated under reduced pressure, subjected to silica gel CC (200 to 300 mesh), and eluted with a mixture of PE and EA in a ratio of 10:1 to yield farnesol. To obtain the enzyme reaction products (compounds **4** and **5**) of *Pc*TS1 and 1,1-D_2_-GGPP or 6F-GGPP, following a 50- or 300-ml scale incubation, the reaction solution was extracted with PE. The resulting PE solution was concentrated under reduced pressure and then subjected to preparative HPLC purification using the same gradient elution program as used for compounds **6** to **13**.

### GC-MS analysis

GC-MS analyses were conducted using an 8890 GC system (Agilent Technologies, Shanghai, China) equipped with a 7650 autosampler (Agilent Technologies, China) and coupled to a 5977C mass spectrometric detector (Agilent Technologies, DE, US). The GC instrument was configured with an HP-5 ms 30-m by 0.25-mm by 0.25-μm film capillary column (Agilent Technologies, US). A 1-μl sample was injected into the GC instrument with a 50:1 split ratio, and the inlet temperature was set at 250°C. Helium was used as the carrier gas with a constant flow rate of 1.0 ml/min. The elution gradient was controlled by an oven temperature program starting at 50°C, ramping up at 10°C/min to 260°C, resulting in a total run time of 21.0 min. The mass spectrometer operated in electron ionization positive mode at 70 eV with an ion source temperature of 250°C, a quadrupole temperature of 130°C, and a transfer line temperature of 250°C. Data acquisition was performed in full-scan mode over a mass range of 50 to 550 *m/z*. Mass spectrometric data analysis was conducted using MassHunter software (version 10.2, Agilent Technologies), with the NIST 23 database used for compound identification.

### TDDFT-ECD calculation

The torsional sampling method, Monte Carlo multiple minimum, and the OPLS_2005 force field were used in Macromodel 9.9.223 software to conduct conformational searches, applying an energy window of 21 kJ/mol. Conformers with more than 1% population underwent reoptimization at the B3LYP/6-311G(d,p) level, incorporating the IEFPCM solvent model for acetonitrile. The ECD spectra were derived from TDDFT calculations, maintaining consistency in the functional, basis set, and solvent model used for energy optimization. Last, the Boltzmann-averaged ECD spectra for compound **1** were generated using SpecDis1.62.

### Site-directed mutation

The synthesized *Pc*TS1 gene was used for site-directed mutagenesis, and overlapped polymerase chain reaction was conducted using TransStar FastPfu Fly DNA polymerase. The polymerase chain reaction products were then purified through gel extraction and inserted into a linearized pET28a vector via BamHI and HindIII digestion. After transforming these constructs into *E. coli* Turbo and confirming them through DNA sequencing, we obtained the desired plasmids. Only plasmids with correct sequences were selected for protein expression in *E. coli* BL21Gold(DE3), which was subsequently analyzed by HPLC. The primer sequences used are detailed in table S3.

### Crystallization, data collection, and structure determination of *Pc*TS1

Crystallization was performed using the sitting-drop vapor diffusion method. The crystals of *Pc*TS1 were grown under 0.2 M trilithium citrate and 20% polyethylene glycol 3350 at 4°C with Mg^2+^ and 2F-GGPP. These crystals were harvested for data collection at 100 K. All data were collected at SSRF and BL10U2, processed with HKL2000 (*42*). For phase determination of *Pc*TS1, molecular replacement was adopted using the structure of TS from *Eleutherobia rubra* CWA1 (PDB ID: 7S5L; 40% identity) as a starting model. The molecular replacement solution was outstanding, and automatic model building was performed with PHENIX (*43*). Additional model building was done manually with Coot (*44*) and refined with PHENIX. The final model of *Pc*TS1 containing residues 5 to 411 was refined to a 3.5 Å resolution with no ligands shown, as the occupancy of Mg^2+^ and 2F-GGPP is low; thus, the electron densities are notably weak. The statistics of the data collection and model refinement are summarized in table S10.

### MD simulations

MD simulations aim to solvate the protein and obtain a reasonable binding conformation of the ligand. Because of the considerable flexibility of the linear substrate (GGPP) and limitations in the accuracy in molecular force fields to describe the ligand, our MD simulations begin with the cyclic carbocation intermediate **D**^+^. The magnesium ion cluster was reconstructed by referring the crystal structure of *Aspergillus terreus* aristolochene synthase (PDB entry: 4KUX) (*45*), which features a well-coordinated magnesium cluster with the inorganic pyrophosphate (PP_i_) group. Subsequently, **D**^+^ was docked into the active pocket using AutoDock Vina software (*46*). The Amber FF99SB (*47*) force field was used for the protein, and the TIP3P (*48*) model was used for solvent water molecules. The restrained electrostatic potential (*49*) charges of the ligand were calculated at the HF/6-31G* level using the Gaussian 16 package. The force field parameters of ligands were generated from the AMBER GAFF force field (*50*). The MD simulations were carried out using the Amber20 package (*51*), and the periodic boundary condition with the cubic model was used to the system. The initial coordinates and topology file were generated by the tleap module in Amber20. Sodium ions were used to neutralize the system. Three steps of minimization were taken to relax the solvent molecules and protein-ligand complex. First, only water molecules in the systems were minimized and then side chains of residues, and last, all atoms were minimized. After minimization, the system was heated from 0 to 300 K gradually under the NVT ensemble, followed by another 100-ps NPT ensemble MD simulation for equilibration at a constant temperature of 300 K and a pressure of 1.0 atm. Afterward, a 50-ns NVT ensemble production MD simulation with a time step of 2 fs was performed for the system. During these simulations, general restraints were used to the magnesium coordination region and the ligand owing to the well-known poor treatment of metal-ligand coordination interaction and ligand conformation by the force field. Notably, all these restraints were removed during the subsequent QM/MM calculations. The SHAKE (*52*) algorithm was used for constraint hydrogens during MD simulations. A cutoff of 12 Å was set for both van der Waals and electrostatic interactions. After the system reached stability, a snapshot was selected to construct the initial structure for subsequent QM/MM simulations.

### QM/MM calculations

The QM/MM model was prepared by deleting the solvent beyond 30 Å from the C1 atom of the ligand on the basis of the protein-substrate complex equilibrated by MD simulations. Because of the restraints imposed in MD, we first subjected the system to a 5-ps unrestrained QM/MM MD process to alleviate potential tensions. The equilibrated structure was then used for subsequent potential energy scan (PES) processes with the reaction coordinate driving method (*53*, *54*). For the selection of the QM region, the carbocations, the three Mg^2+^ ions, and the PP_i_ group, along with the aromatic residues Phe93, F221, and Trp335, were included in the QM region during the QM/MM MD and PES calculations from **D**^+^ to product **1**. These residues were chosen because of their proximity to the intermediates and their potential role in stabilizing the carbocations. The Mg^2+^ cluster and PP_i_ group were removed from the QM region during the PES calculations from **A**^+^ to **C**^+^, as these rearrangements do not involve the participation of the PP_i_ group. For the definition of reaction coordinates, please refer to fig. S18. All atoms in the QM subsystem were treated by the M06-2X/6-31G(d) method (*55*, *56*), which is widely used in studies of cyclization reactions (*57*, *58*). The QM/MM boundary was treated by the improved pseudobond approach (*59*–*61*). The Amber FF99SB force field was used for all the remaining atoms, consistent with previous MD simulations. The spherical boundary condition was used to the system, with the atoms located more than 30 Å from the spherical center being fixed. A 12-Å cutoff was used for van der Waals interactions, while an 18-Å cutoff was used for electrostatic interactions. The system temperature was controlled by the Langevin thermostat method (*62*) at 300 K, and the Newton equation of motion was integrated by the Beeman algorithm (*63*) during the QM/MM MD process. All these QM/MM calculations were performed with the modified QChem (*64*) and Tinker (*65*) programs developed by Zhang’s group (www.nyu.edu/projects/yzhang/). In addition, the similar QM/MM protocol had also been used in our previous studies of other TSs (*66*–*68*).

### Binding energy calculation

To calculate the relative binding energies, we pairwise extracted the structures of the important residues and intermediates from the QM/MM scan trajectories. The residues were truncated between the α-carbon and the β-carbon and then saturated with hydrogen atoms. The relative binding energies were computed using Gaussian 16 with the same method used in the QM region of the QM/MM calculation. To avoid basis set superposition errors, we used the counterpoise correction method developed by Boys and Bernardi (*69*).

## Supplementary Materials

### This PDF file includes:

Compound structure elucidation Compound schemes Figs. S1 to S96 Tables S1 to S11 References
