# Supplementary material for: Mining coral-derived terpene synthases and mechanistic studies of the coral biflorane synthase
Source: Sci Adv. 2025 Feb 26;11(9):eadv0805. doi: 10.1126/sciadv.adv0805 (PMC11864185; doi:10.1126/sciadv.adv0805)
Supplement: Supplementary file 1 — Compound structure elucidation Compound schemes Figs. S1 to S96 Tables S1 to S11 References [file sciadv.adv0805_sm.pdf]

Science Advances
AAAS

Supplementary Materials for

Mining coral-derived terpene synthases and mechanistic studies of the coral biflorane synthase

Bao Chen *et al.*

Corresponding author: Baofu Xu, bfxu@simm.ac.cn; Chengyuan Wang, cywang@ips.ac.cn; Ruibo Wu, wurb3@mail.sysu.edu.cn; Yue-Wei Guo, ywguo@simm.ac.cn

Sci. Adv. **11**, eadv0805 (2025)
DOI: 10.1126/sciadv.adv0805

This PDF file includes:

Compound structure elucidation
Compound schemes
Figs. S1 to S96
Tables S1 to S11
References

Compound structure elucidation

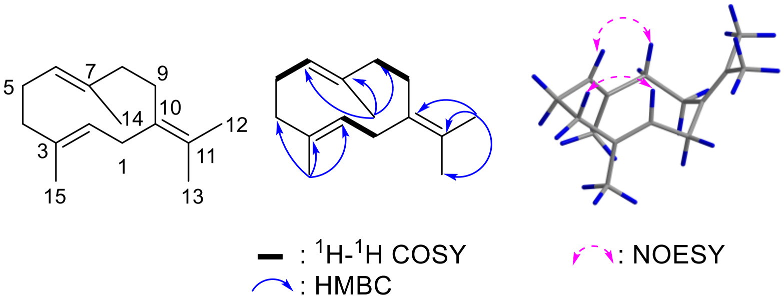

Germacrene B was readily identified by comparing its NMR spectroscopic data with those reported in the literature (*17*) further confirmed by 2D NMR correlations. ^{1}H NMR (C_6D_6, 400 MHz) δ 1.46 (3H, s, H-14), 1.48 (3H, s, H-15), 1.63 (3H, s, H-12), 1.67 (3H, s, H-13), 1.81 (1H, m, H-9a), 1.97 (1H, m, H-4a), 1.99 (1H, m, H-5a), 2.11 (1H, m, H-4b), 2.15 (2H, m, H-8a, H-8b), 2.25 (1H, m, H-5b), 2.51 (1H, m, H-9b), 2.52 (1H, m, H-1a), 2.98 (1H, m, H-1b), 4.66 (1H, d, J = 11.1, H-2), 4.76 (1H, d, J = 12.0, H-6); ^{13}C NMR (C_6D_6, 100 MHz) δ 136.9, 133.7, 131.6, 128.6, 126.9, 126.3, 41.0, 39.3, 34.0, 32.9, 26.2, 20.9, 20.5, 17.3, 16.4.

Farnesol was also identified by comparing its ^{1}H and ^{13}C NMR spectroscopic data with those reported in the literature (*18*). ^{1}H NMR ($CDCl_3$, 600 MHz) δ 1.62 (6H, s, CH_3), 1.70 (6H, s, CH_3), 1.98–2.16 (8H, m, CH_2), 4.17 (1H, d, J = 6.9 Hz, CH_2), 5.11 (1H, m, vinyl H), 5.13 (1H, m, vinyl H), 5.44 (1H, m, vinyl H); ^{13}C NMR ($CDCl_3$, 150 MHz) δ 140.0, 135.5, 131.5, 124.4, 123.9, 123.5, 59.5, 39.8, 39.7, 26.9, 26.4, 25.8, 17.8, 16.4, 16.1.

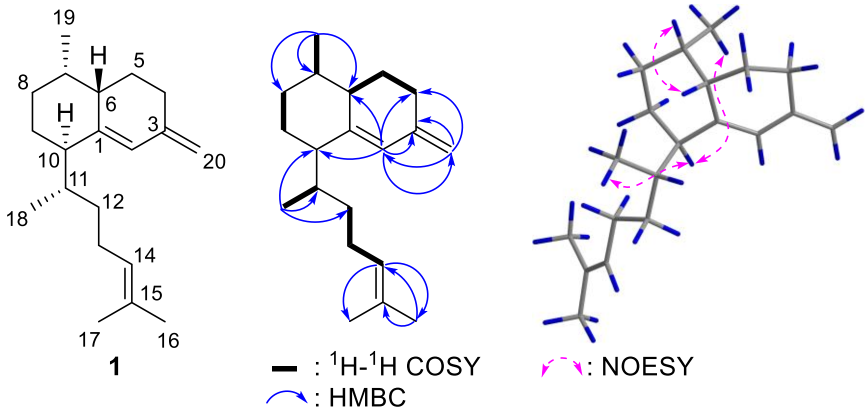

Compound **1** was obtained as a colorless oil, and its molecular formula was deduced to be $C_{20}H_{32}$ by GC-MS, which exhibited an M^{+} peak at m/z of 272.2, indicating 5 degrees of unsaturation. The ^{1}H NMR spectrum (Supplementary Table S5) showed four methyls at δ_H 1.69 (3H, s, H_3-16), δ_H 1.61 (3H, s, H_3-17), 0.93 (3H, d, J = 6.4 Hz, H_3-16), δ_H 0.91 (3H, d, J = 7.0 Hz, H_3-17), one terminal double bond at δ_H 4.71 (1H, s, H_2-20a), δ_H 4.66 (1H, s, H_2-20b), and two olefinic protons at δ_H 6.03 (1H, brs, H-2), δ_H 5.07 (1H, m, H-14), attributed to 2 trisubstituted double bonds. These olefinic bonds accounted for three degrees of unsaturation, a finding further confirmed by identifying six olefinic carbons (δ_C 153.5, 134.1, 132.2, 127.6, 124.8, 107.1) using ^{13}C NMR and HSQC spectra, suggesting the remaining two degrees of unsaturation are due to a bicyclic system. Significant HMBC correlations from H-2 (δ_H 6.03) to C-3 (δ_C 144.5)/C-4 (δ_C 29.6)/C-6 (δ_C 37.0)/C-10 (δ_C 49.8)/C-20 (δ_C 108.2), H-14 (δ_H 5.07) to C-16 (δ_C 25.9)/C-17 (δ_C 17.8), H_3-16 (δ_H

1.69) to C-14 (δ_C 125.3)/C-15 (δ_C 131.1), H_3-18 (δ_H 0.93) to C-10/C-11 (δ_C 31.8)/C-12 (δ_C 34.8), H_3-19 (δ_H 0.91) to C-6/C-7 (δ_C 35.3)/C-8 (δ_C 29.5), H_2-20 (δ_H 4.71, 4.66) to C-2/C-3/C-4, and the ^{1}H-^{1}H COSY spectrum from H_2-4 (δ_H 2.37, 2.20) to H-6 (δ_H 2.39), H_2-8 (δ_H 1.84, 1.32) to H_2-9 (δ_H 1.68), H_2-12 (δ_H 1.40, 0.94) to H-14, H_3-18 to H-11 (δ_H 1.73), H_3-19 to H-7 (δ_H 1.99) helped construct the biflorane skeleton. The planar structure was confirmed by comparing the ^{1}H and ^{13}C NMR data with those of model compound elisabethatriene (*15*), a precursor to pseudopterosins. The relative configuration was confirmed by NOESY experiment to be in agreement with elisabethatriene. The absolute configuration of **1** was identified by time-dependent density functional theory/electronic circular dichroism (TDDFT/ECD) calculations, with the Boltzmann-averaged ECD spectrum of (6*S*,7*S*,10*R*,11*S*)-**1** displaying an identical curve to the experimental one, confirming the (6*S*,7*S*,10*R*,11*S*) absolute configuration.
Compound **1**: colorless oil; $[\alpha]^{20}_{D}$ +49.3 (*c* 0.20, CH_3OH); For ^{1}H NMR ($CDCl_3$, 600 MHz) and ^{13}C NMR ($CDCl_3$, 150 MHz) spectral data, see Tables S5; For ^{1}H NMR (C_6D_6, 600 MHz) and ^{13}C NMR (C_6D_6, 150 MHz) spectral data, see Tables S6; GC-MS *m/z* 272.2.

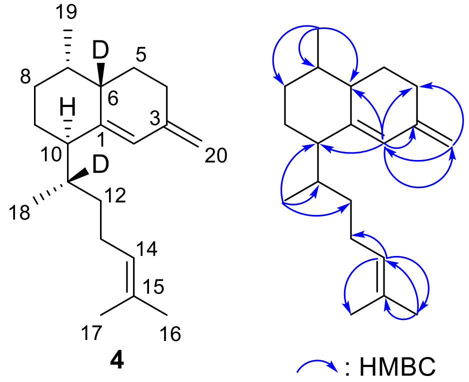

4

Compound **4** was identified as the 6,11-dideuterated derivative of compound **1**, as stated in the main text, further confirmed by HMBC correlations.
Compound **4**: colorless oil; $[\alpha]^{20}_{D}$ +22.7 (*c* 0.15, CH_3OH); For ^{1}H NMR ($CDCl_3$, 600 MHz) and ^{13}C NMR ($CDCl_3$, 150 MHz) spectral data, see Tables S5; GC-MS *m/z* 274.3.

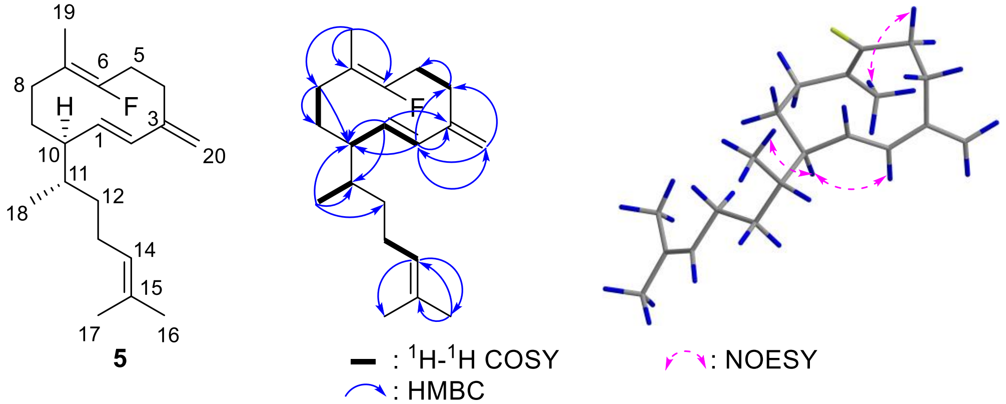

5

The structure of compound **5** was thoroughly explained in the main text and was further confirmed through 2D NMR analysis.
Compound **5**: colorless oil; $[\alpha]^{20}_{D}$ +44.0 (*c* 0.05, CH_3OH); For ^{1}H NMR (C_6D_6, 600 MHz) and ^{13}C NMR (C_6D_6, 150 MHz) spectral data, see Tables S7; GC-MS *m/z* 290.2.

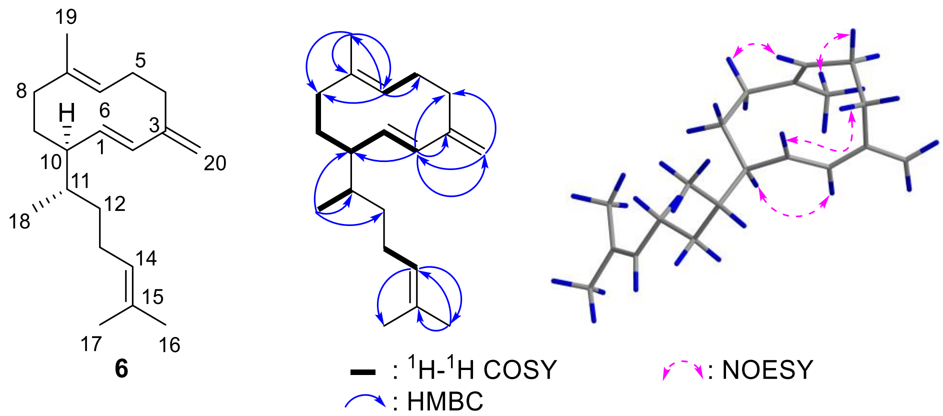

Compound **6** was obtained as a colorless oil. Its molecular formula, $C_{20}H_{32}$, was established by GC-MS showing an M^+ peak at *m*/*z* of 272.3, suggesting 5 degrees of unsaturation, the same as that of **1**. Its ^{13}C NMR and HSQC spectroscopic data (Table S7) implied the presence of eight sp^2 carbon atoms (δ_C 149.0, 136.4, 133.9, 133.6, 131.0, 129.9, 125.6, 109.4) at lower field, accounting for four degrees of unsaturation. Consequently, the nature of **6** was a monocyclic molecule. The clear ^{1}H-^{1}H COSY spectrum from H-2 (δ_H 5.78) to H_2-8 (δ_H 2.23), H_2-5 (δ_H 2.35, 1.96) to H-6 (δ_H 5.16) and the HMBC correlations from H_2-2 (δ_H 5.78) to C-3 (δ_C 149.0)/C-4 (δ_C 35.0)/C-10 (δ_C 52.4)/C-20 (δ_C 109.4), H-6 to C-5 (δ_C 29.7)/C-8 (δ_C 41.1)/C-19 (δ_C 16.1), H_3-18 to C-10/C-11 (δ_C 37.9)/C-12 (δ_C 34.0), H_3-19 to C-6 (δ_C 129.9)/C-7 (δ_C 133.9)/C-8, and H_2-20 (δ_H 4.95, 4.80) to C-2 (δ_C 136.4)/C-3/C-4, helped construct the 10 member ring (pictured below). Consequently, compound **6** was determined as the 1,10-cyclization product of GGPP. The configurations of C1/C2 and C6/C7 double bonds were assigned as all *E*, as deduced by the NOE correlations of H-1/H_2-4, H-2/H-10, H_2-5/H_3-19 and H-6/H_2-8, as well as by a large coupling constant ($^3J_{H-1,\ H-2}$ = 15.8 Hz).

Compound **6**: colorless oil; $[\alpha]_D^{20}$ +60.0 (*c* 0.18, CH_3OH); For ^{1}H NMR ($CDCl_3$, 600 MHz) and ^{13}C NMR ($CDCl_3$, 150 MHz) spectral data, see Tables S7; GC-MS *m*/*z* 272.2.

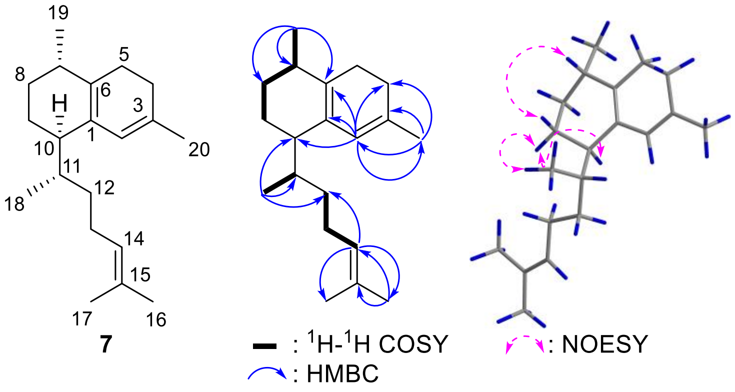

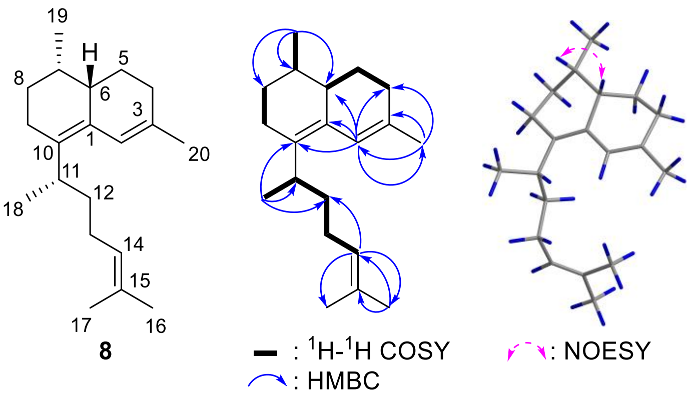

8

Compound **7** and **8** form a 2:1 ratio isomeric mixture. Due to the small quantity of the compounds, no further separation and purification were carried out. Compound **7** readily identified as isoelisabethatriene by comparing its NMR spectroscopic data (Table S8) with those reported in the literature (*8*, *70*) further confirmed by 2D NMR correlations. The NMR data of **8** were strongly reminiscent of those of the co-occurring diterpene isoelisabethatriene (**7**). A comparison of the ^{1}H and ^{13}C NMR data (Table S8) revealed that **8** differed from **7** only by the location of the double bond. The HMBC experiment showed the following correlations: H-2 to C-1/C-4/C-6/C-10/C-20, H_3-19 to C-6/C-7/C-8, H_3-18 to C-10/C-11/C-12, allowing the location of the double bond at $\Delta^{1,10}$ instead of $\Delta^{1,6}$ in compound **7**. Compound **8** was thus determined to be an analog of **7** with a double bond at $\Delta^{1,10}$.

Compound **7**: colorless oil; $[\alpha]^{20}_{D}$ +96.0 (*c* 0.05, CH_3OH); For ^{1}H NMR ($CDCl_3$, 600 MHz) and ^{13}C NMR ($CDCl_3$, 150 MHz) spectral data, see Tables S8; GC-MS *m/z* 272.2.

Compound **8**: colorless oil; $[\alpha]^{20}_{D}$ +96.0 (*c* 0.05, CH_3OH); For ^{1}H NMR ($CDCl_3$, 600 MHz) and ^{13}C NMR ($CDCl_3$, 150 MHz) spectral data, see Tables S8; GC-MS *m/z* 272.2.

Compound **9** was identified as being identical to the product obtained from the co-incubation of 2*Z*-GGPP and Bnd4 (compound **10** in the literature), by comparing its ^{1}H and ^{13}C NMR spectroscopic data (Figs. S69 and S70) to those reported in literature (*16*).

Compound **9**: colorless oil; $[\alpha]^{20}_{D}$ –27.3 (*c* 0.10, CH_3OH); ^{1}H NMR (C_6D_6, 600 MHz) δ 0.83 (3H, d, J = 6.9 Hz, CH_3-18), 1.57 (3H, s, CH_3-17), 1.65 (3H, s, CH_3-19), 1.67 (3H, s, CH_3-20), 1.70 (3H, s, CH_3-16), 5.23 (1H, m, CH-14), 5.61 (1H, brs, CH-2); ^{13}C NMR (C_6D_6, 150 MHz) δ 134.0, 131.0, 130.5, 125.6, 125.0, 124.2, 43.6, 39.7, 36.3, 32.8, 32.4, 31.9, 27.3, 26.7, 25.9, 23.8, 21.9, 18.7, 17.8, 14.2.

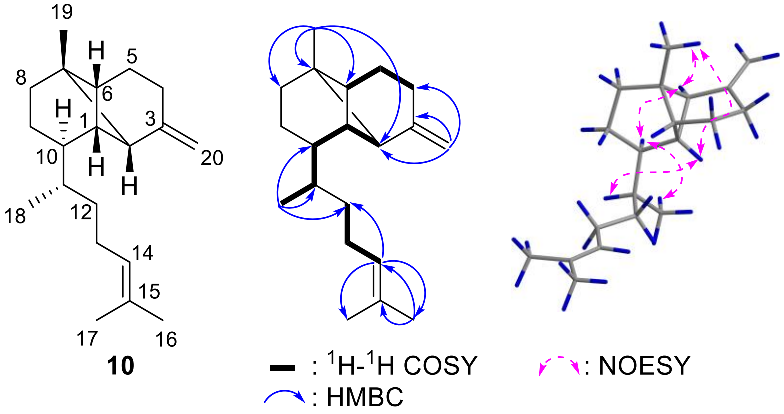

10

Compound **10** was also obtained as a colorless oil. Its molecular formula, $C_{20}H_{32}$, was determined by GC-MS, revealing an M^{+} peak at *m/z* of 272.3, indicating 5 degrees of unsaturation, consistent

with compound **1**. Analysis of its ^{13}C NMR and HSQC spectroscopic data (Table S9) suggested the presence of four sp^2 carbon atoms (δ_C 151.5, 131.0, 125.6, 106.7) at lower field, accounting for two degrees of unsaturation. Therefore, compound **10** was identified as a tricyclic molecule. Clear HMBC correlations from H_3-19 (δ_H 0.77) to C-2 (δ_C 60.7)/C-6 (δ_C 37.1)/C-7 (δ_C 42.9)/C-8 (δ_C 36.8), H_2-20 to C-2/C-3 (δ_C 151.5)/C-4 (δ_C 24.6), and ^1H-^1H COSY spectrum from H-2 (δ_H 2.15) to H_3-18 (δ_H 0.84), H_2-4 (δ_H 2.46, 2.22) to H-6 (δ_H 1.97) helped confirm a 2,7 cyclization after 1,6 cyclization to form **10**. This was consistent with the signal for H_3-19 in **10**, which exhibited a singlet instead of a doublet in **1**. The configuration of compound **10** was determined through NOE correlations and ring-closure mechanisms, as depicted in the figure above.

Compound **10**: colorless oil; $[\alpha]_D^{20}$ +24.8 (*c* 0.25, CH_3OH); For ^1H NMR (C_6D_6, 600 MHz) and ^{13}C NMR (C_6D_6, 150 MHz) spectral data, see Tables S9; GC-MS *m/z* 272.2.

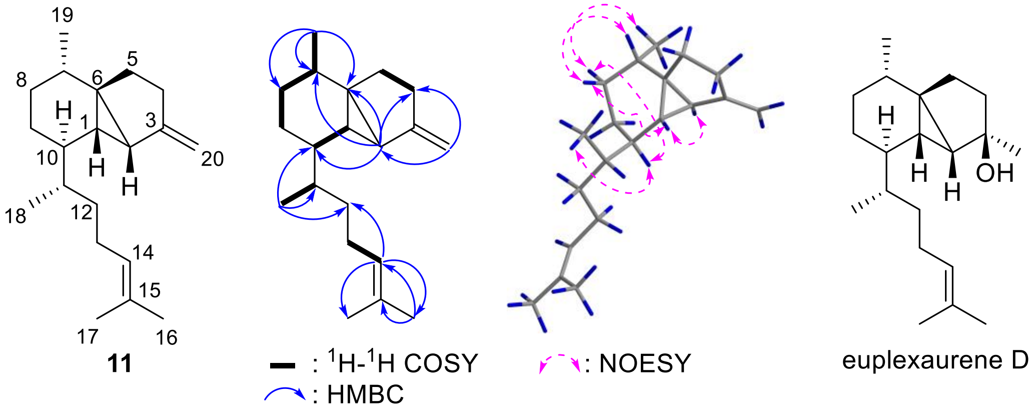

Compound **11** was isolated as a colorless oil, with a molecular formula of $C_{20}H_{32}$ determined via GC-MS, showing an *m/z* of 272.3. Its NMR data closely resembled those of euplexaurene D (*71*), a serrulatane terpenoid obtained from the South China Sea soft coral *Lemnalia* sp. Comparison of the NMR data between **11** and euplexaurene D (Table S9) revealed them to be analogs, differing only at the C-3 and C-20 positions due to the presence of typical terminal double bond signals (δ_C 154.6; δ_C 102.0, δ_H 5.00, 4.78), consistent with the observed molecular mass difference of 18 mass units. HMBC experiments showed correlations such as H-2 to C-4 (δ_C 29.4)/C-6 (δ_C 38.4)/C-7 (δ_C 31.5)/C-10 (δ_C 42.9), H_2-20 to C-2 (δ_C 36.4)/C-4 (δ_C 29.4), confirming the placement of the terminal double bond at C-3 and C-20. The configuration of compound **11** was determined to be the same as euplexaurene D through NOE correlations and ring-closure mechanisms, as illustrated in the figure above.

Compound **11**: colorless oil; $[\alpha]_D^{20}$ +116.0 (*c* 0.10, CH_3OH); For ^1H NMR (C_6D_6, 600 MHz) and ^{13}C NMR (C_6D_6, 150 MHz) spectral data, see Tables S9; GC-MS *m/z* 272.2.

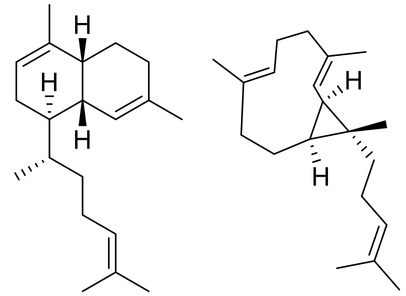

12 **13**

Compound **12** was readily identified as biflora-4,9,15-triene by comparing its NMR spectroscopic data with those reported in the literature (*72*). The configuration of compound **12** was determined through ring-closure mechanisms.

Compound **12**: colorless oil; $[\alpha]_D^{20}$ +42.0 (*c* 0.10, CH_3OH); ^1H NMR (C_6D_6, 600 MHz) δ 0.82 (3H, d, *J* = 6.8 Hz, CH_3-18), 1.59 (3H, s, CH_3-16), 1.67 (3H, s, CH_3-17), 1.69 (3H, s, CH_3-19), 1.69 (3H, s, CH_3-20), 5.09 (1H, m, CH-14), 5.41 (1H, brs, CH-8), 5.49 (1H, m, CH-2); ^{13}C NMR (C_6D_6, 150 MHz) δ 136.7, 134.5, 131.1, 125.2, 124.1, 121.6, 39.7, 39.0, 36.5, 35.9, 31.5, 31.0, 26.4, 25.9, 24.8, 24.1, 21.9, 17.8, 13.4.

Compound **13** was readily identified as (–)-(1*R*,10*S*,11*R*)-cneorubin Y by comparing its NMR spectroscopic data with those reported in the literature (*33*).

Compound **13**: colorless oil; $[\alpha]_D^{20}$ –28.0 (*c* 0.05, CH_3OH); ^1H NMR (C_6D_6, 600 MHz) δ 1.06 (3H, s, CH_3-18), 1.50 (3H, s, CH_3-19), 1.59 (3H, s, CH_3-16), 1.65 (3H, s, CH_3-20), 1.70 (3H, s, CH_3-17), 4.42 (1H, d, *J* = 11.2 Hz, CH-2), 4.81 (1H, m, CH-6), 5.25 (1H, m, CH-14); ^{13}C NMR (C_6D_6, 150 MHz) δ 140.5, 130.8, 128.5, 126.8, 125.4, 125.4, 43.9, 41.6, 37.7, 29.6, 27.3, 27.1, 26.5, 25.9, 25.6, 24.0, 21.1, 17.7, 16.8, 13.1.

Compound schemes from Materials and Methods

Synthesis of 6F-GGPP

Synthesis of (2*Z*,6*E*)-2-fluoro-3,7,11-trimethyldodeca-2,6,10-trien-1-ol

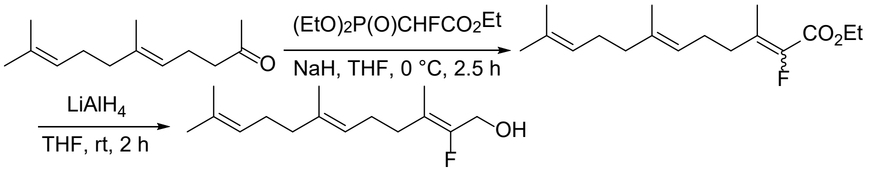

Synthesis of (2*Z*)-1-bromo-2-fluoro-3,7,11-trimethyldodeca-2,6,10-triene

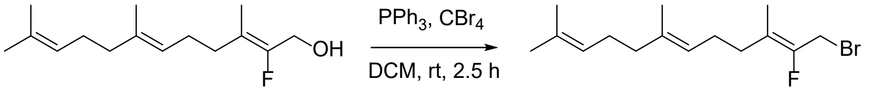

Synthesis of (6*Z*,10*E*)-6-fluoro-7,11,15-trimethyl-3-oxohexadeca-6,10,14-trienoic acid, ethyl ester

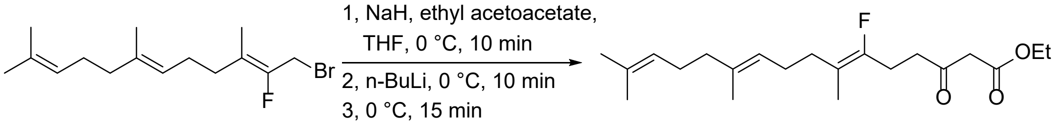

Synthesis of (2*Z*,6*Z*,10*E*)-3-(diisopropoxyphosphoryloxy)-6-fluoro-7,11,15-trimethylhexadeca-2,6,10,14-tetraenoic acid, ethyl ester

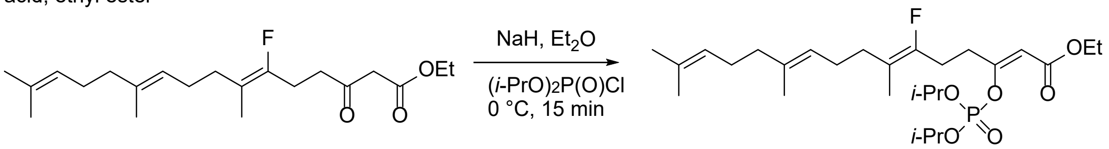

Synthesis of (2*E*/*Z*,6*Z*,10*E*)-6-fluoro-3,7,11,15-tetramethylhexadeca-2,6,10,14-tetraenoic acid, ethyl ester

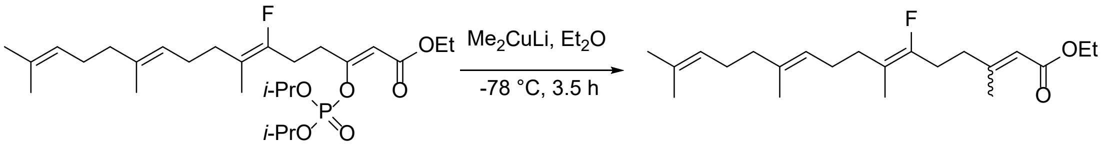

Synthesis of (2*E*,6*Z*,10*E*)-6-fluoro-3,7,11,15-tetramethylhexadeca-2,6,10,14-tetraen-1-ol

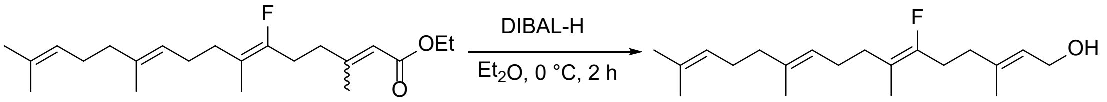

Synthesis of (2*E*,6*Z*,10*E*)-1-chloro-6-fluoro-3,7,11,15-tetramethylhexadeca-2,6,10,14-tetraene

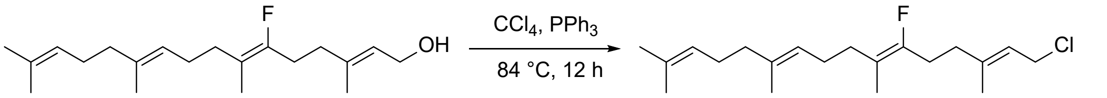

Synthesis of 6F-GGPP

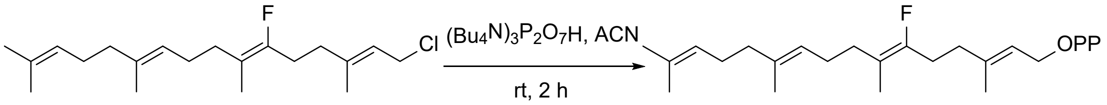

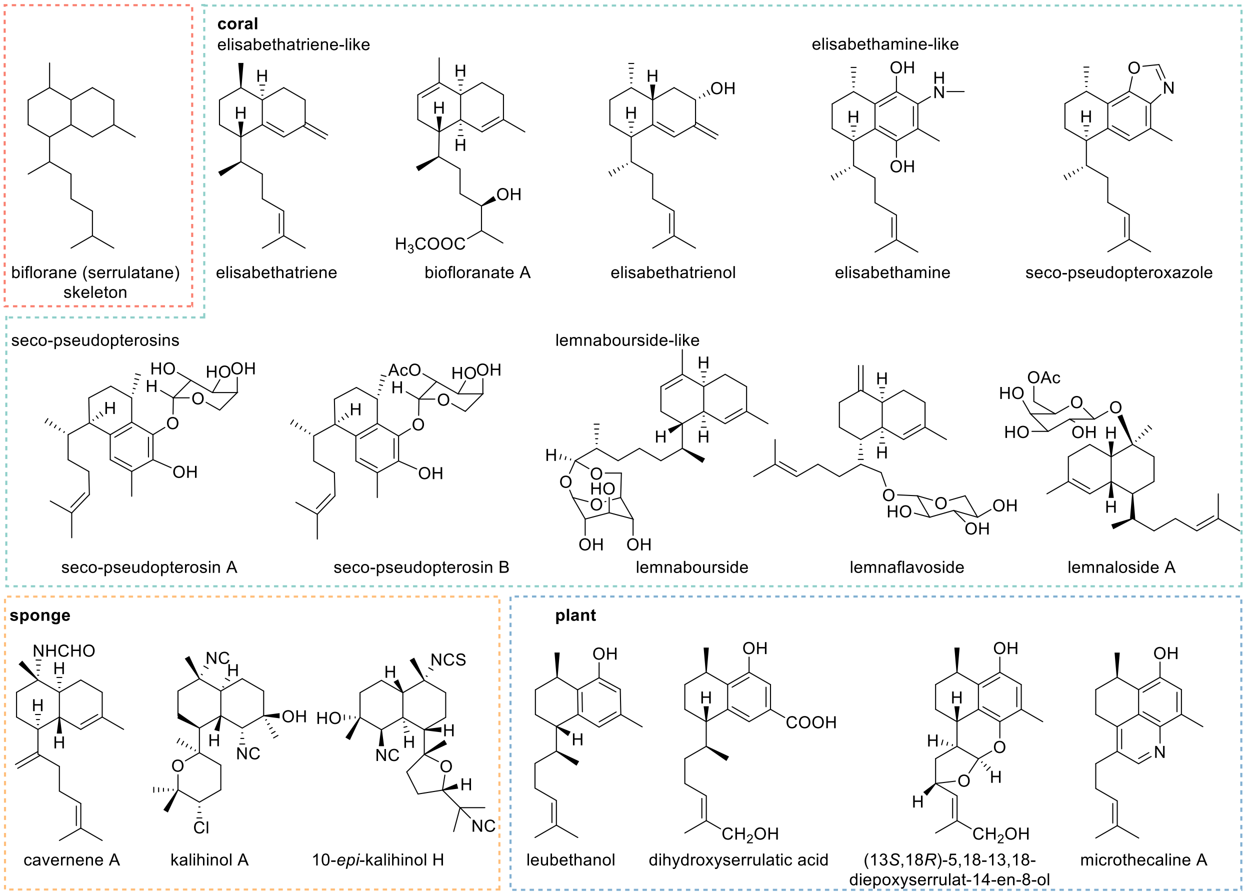

Fig. S1. Biflorane (serrulatane) diterpenoids.

Up to now, over 290 biflorane (also known as serrulatane) compounds have been identified (*5*). However, the use of either the biflorane or serrulatane naming systems presents significant disadvantages. When searching for biflorane, 45 compounds are found across 9 reports on natural products with biflorane skeletons. Similarly, using serrulatane as the search term yields 45 reports on compounds with serrulatane skeletons. In reality, both terms refer to the same molecular skeleton as shown above. The literature investigation reveals that bifloranes isolated from corals can be divided into four categories: compounds without nitrogen atoms and sugars (elisabethatriene-like compounds), nitrogenous compounds (elisabethamine-like compounds), diterpene glycosides with (seco-pseudopterosins) or without a benzene ring (lemnabourside-like compounds). Meanwhile, bifloranes derived from sponges feature functional groups such as cyano and isocyano, and those from plants exhibit varying degrees of oxidation.

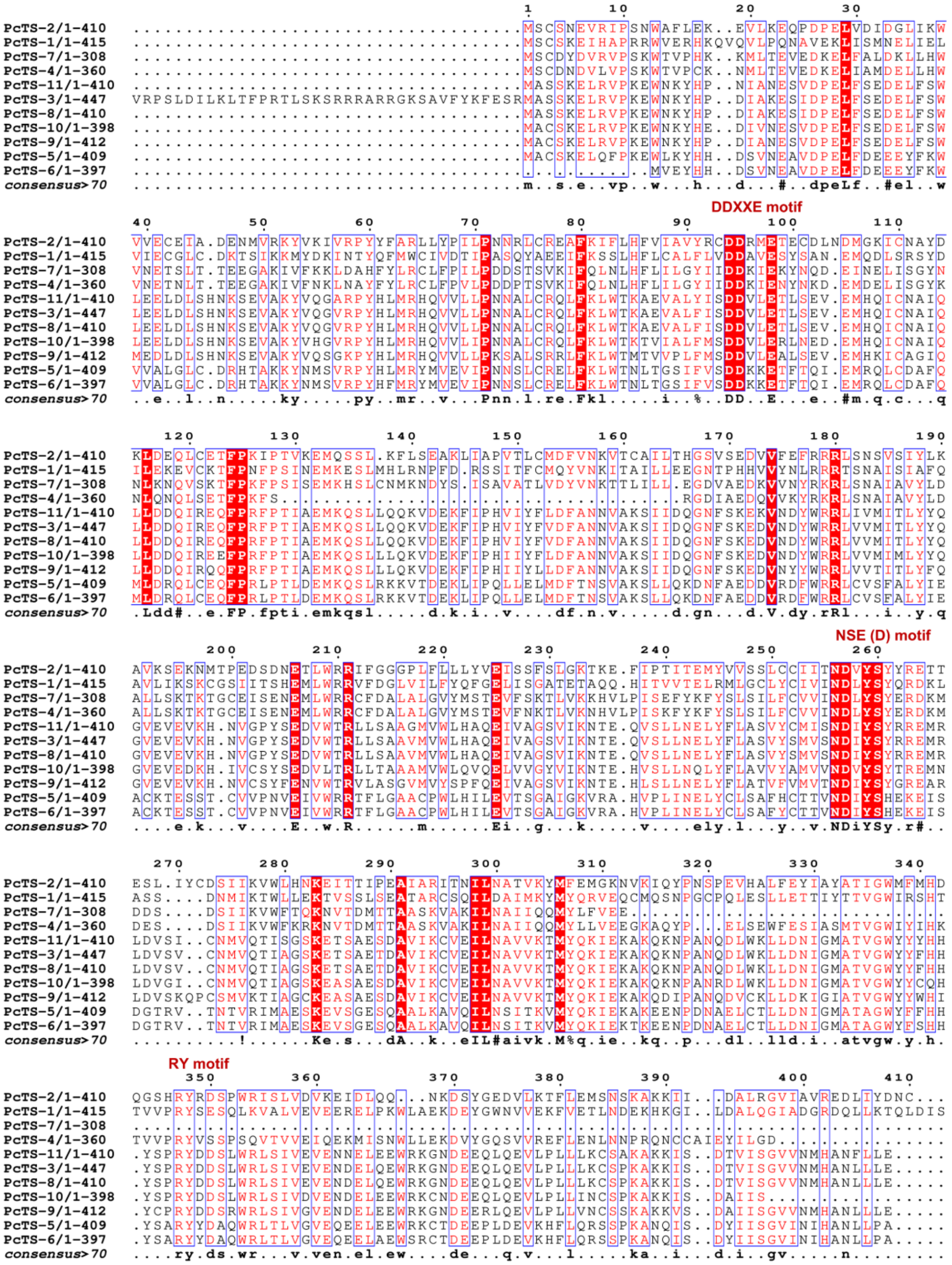

Fig. S2. Sequence alignment of TSs in *P. clavate* and their conserved type I TSs motifs.

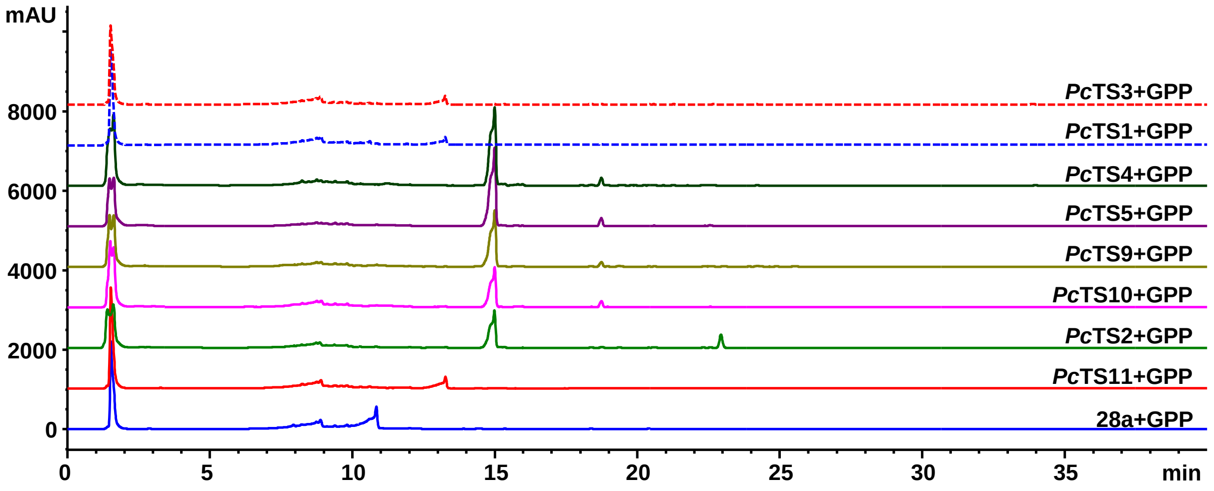

Fig. S3. Functional characterization of selected TSs utilizing previously constructed GPP overproduction system MKI2 (*16*).

The term "28a" shown in the figure refers to the "empty vector pET28a".

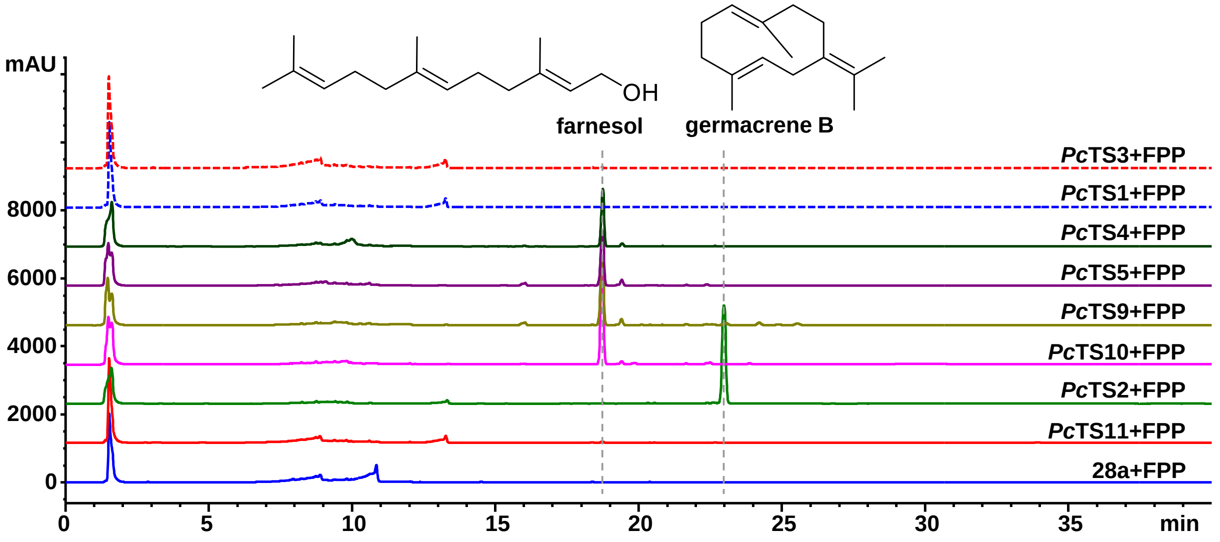

Fig. S4. Functional characterization of selected TSs utilizing previously constructed FPP overproduction system MKI3 (*16*).

The term "28a" shown in the figure refers to the "empty vector pET28a". *Pc*TS2 catalyzed the conversion of FPP to germacrene B, and four TSs (*Pc*TS4, *Pc*TS5, *Pc*TS9, *Pc*TS10) catalyzed the conversion of FPP to farnesol.

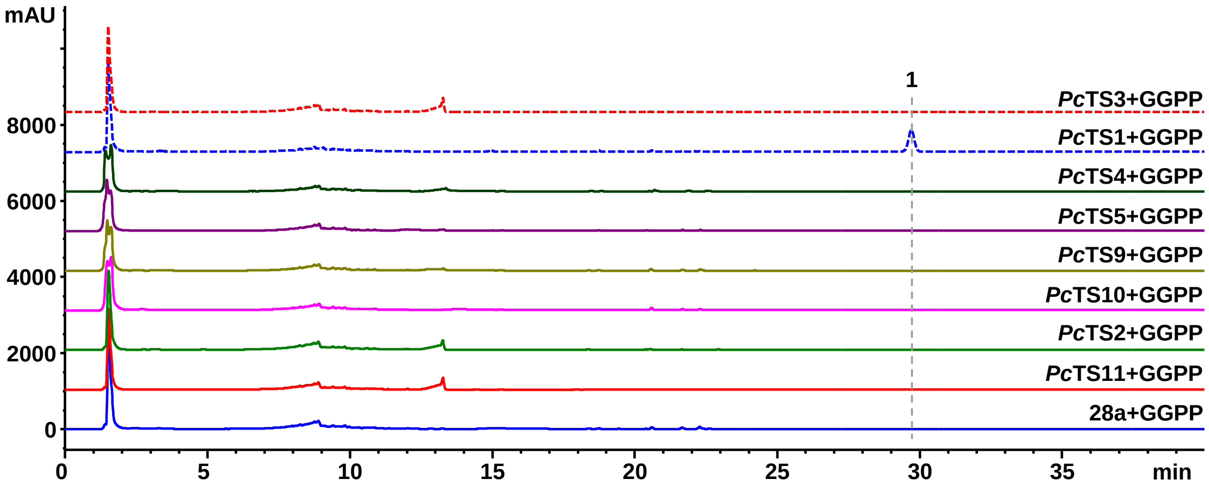

Fig. S5. Functional characterization of selected TSs utilizing previously constructed GGPP overproduction system MKI4 (*16*).

The term "28a" shown in the figure refers to the "empty vector pET28a". *Pc*TS1 catalyzed the conversion of GGPP to the biflorane compound **1**.

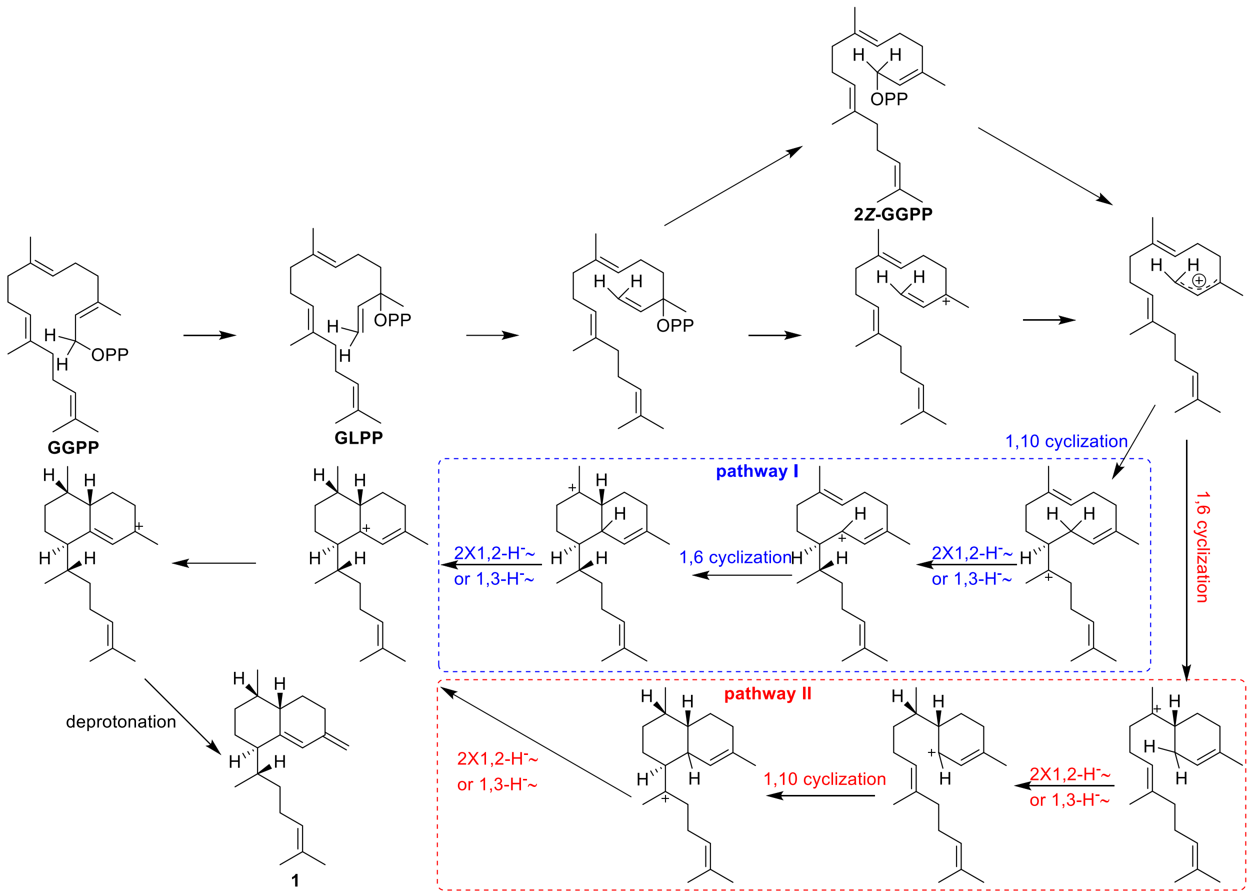

Fig. S6. Two plausible pathways for the formation of the 6,6-bicyclic biflorane skeleton.

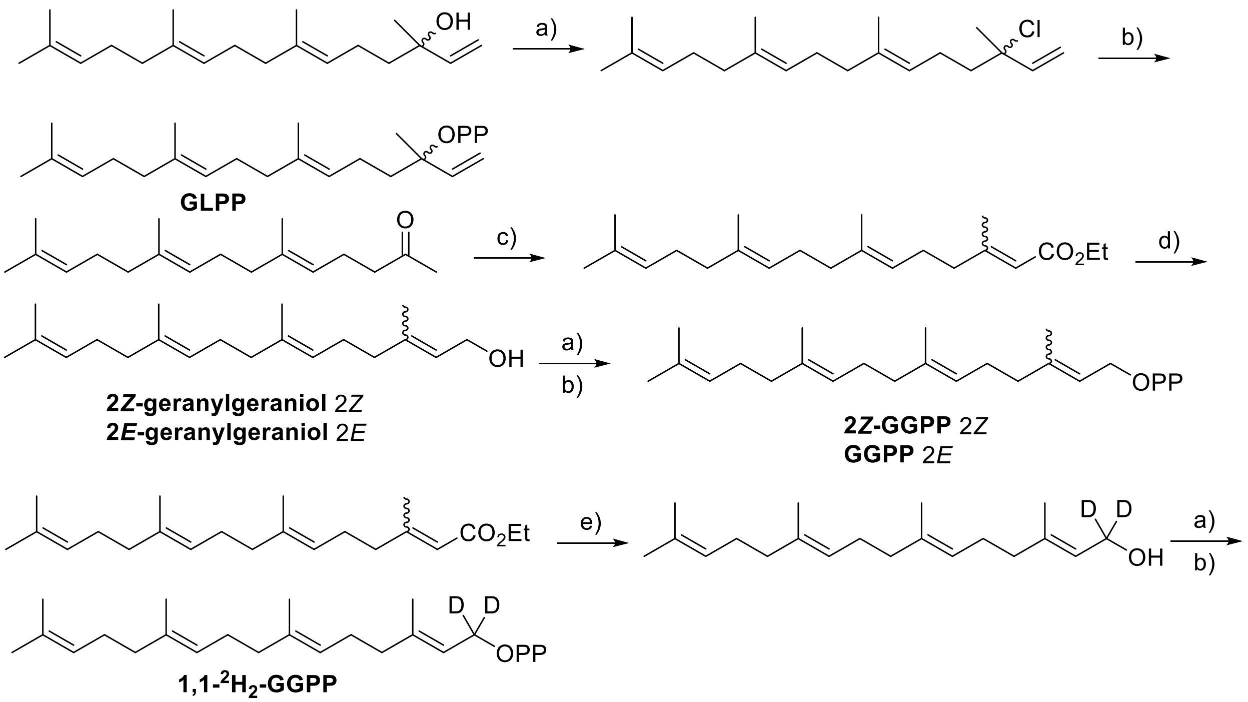

Fig. S7. Synthesis of GLPP, GGPP, 2*Z*-GGPP, and 1,1-D_2-GGPP (*16*).

Reaction conditions: a) CCl_4, PPh_3, 84 °C, 12 h; b) $(Bu_4N)_3P_2O_7H$, ACN, rt, 2 h; c) $(EtO)_2P(O)CH_2CO_2Et$, LDA, THF, -78 °C, 12 h; d) DIBAlH, Et_2O, 0 °C, 2 h; e) $LiAlD_4$, THF, 0 °C, 4 h.

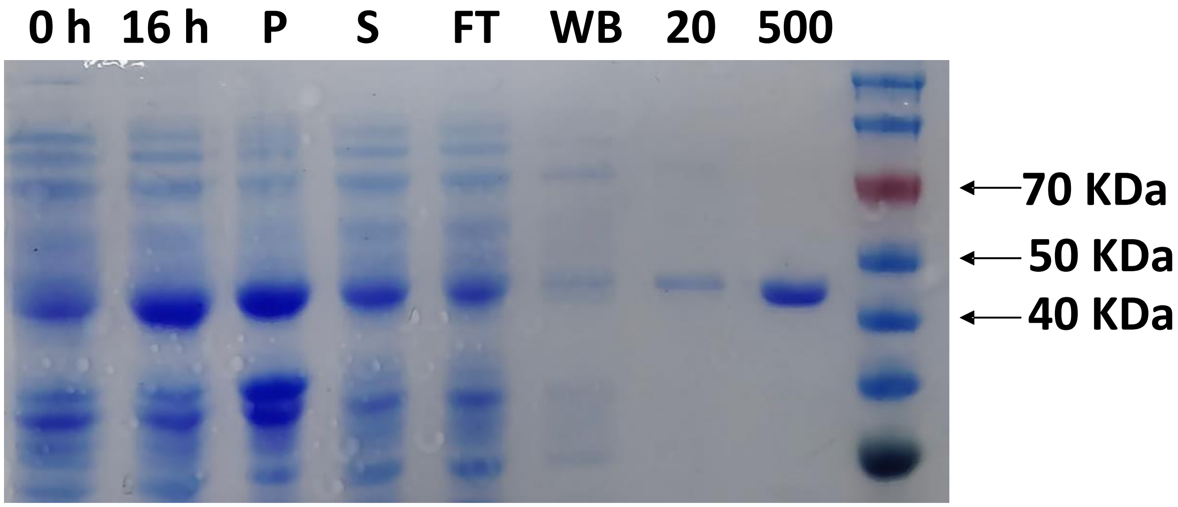

Fig. S8. The SDS-PAGE analysis of PcTS1 protein (49.8 kDa).

P: precipitate; S: supernatant; FT: flow through; WB: wash buffer; 20: 20 mM imidazole, 50 mM Tris-HCl, 150 mM NaCl; 500: 500 mM imidazole, 50 mM Tris-HCl, 150 mM NaCl.

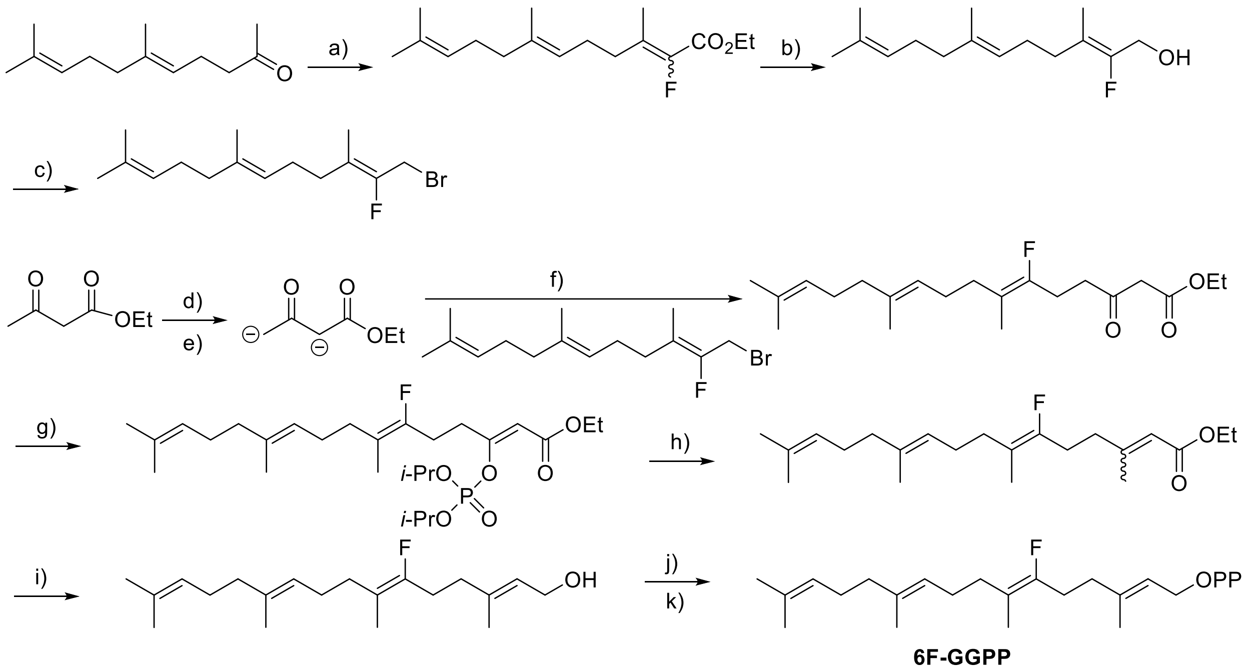

Fig. S9. Synthesis of 6F-GGPP.

Reaction conditions: a) $(EtO)_2P(O)CHFCO_2Et$, NaH, THF, 0 °C, 3.5 h; b) $LiAlH_4$, THF, rt, 24 h; c) PPh_3, CBr_4, DCM, rt, 2.5 h; d) NaH, THF, 0 °C, 10 min; e); n-BuLi, 0 °C, 10 min; f) 0 °C, 15 min; g) NaH, Et_2O, $(PhO)_2P(O)Cl$, 0 °C, 15 min; h) Me_2CuLi, Et_2O, -78 °C, 3.5 h; i) DIBAL-H, Et_2O, -78 °C, 2 h; j) CCl_4, PPh_3, 84 °C, 12 h; k) $(Bu_4N)_3P_2O_7H$, ACN, rt, 2 h.

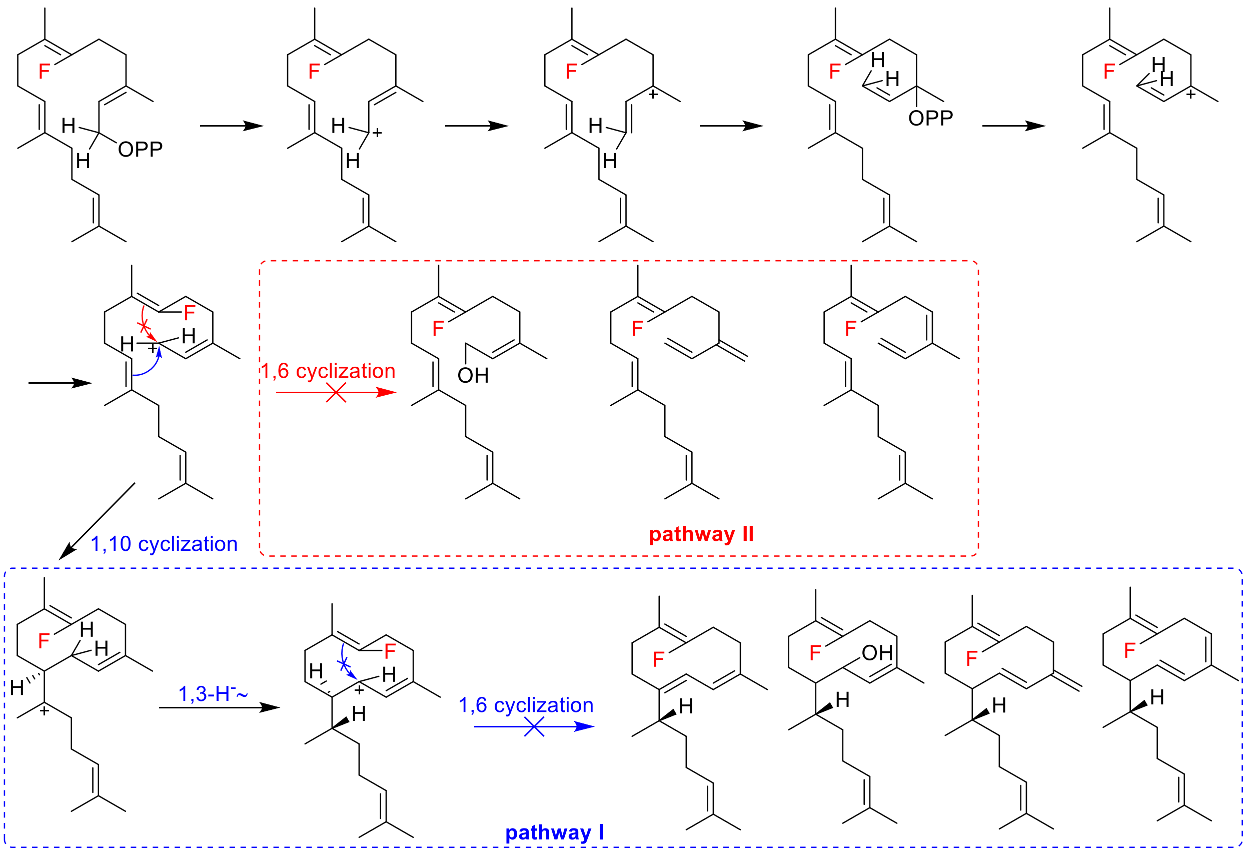

Fig. S10. Plausible pathways for incubation of *Pc*TS1 and 6F-GGPP in vitro and the possible intermediate shunt fluorinated products.

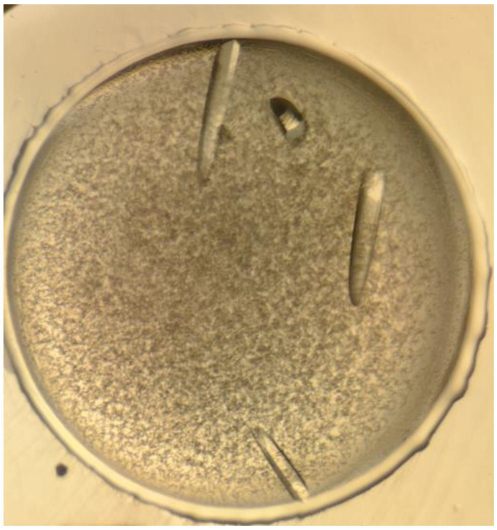

0.2 M Tri-lithium citrate, 20% PEG3350, 4 °C

Fig. S11. Protein crystallization conditions and crystallography of *Pc*TS1.

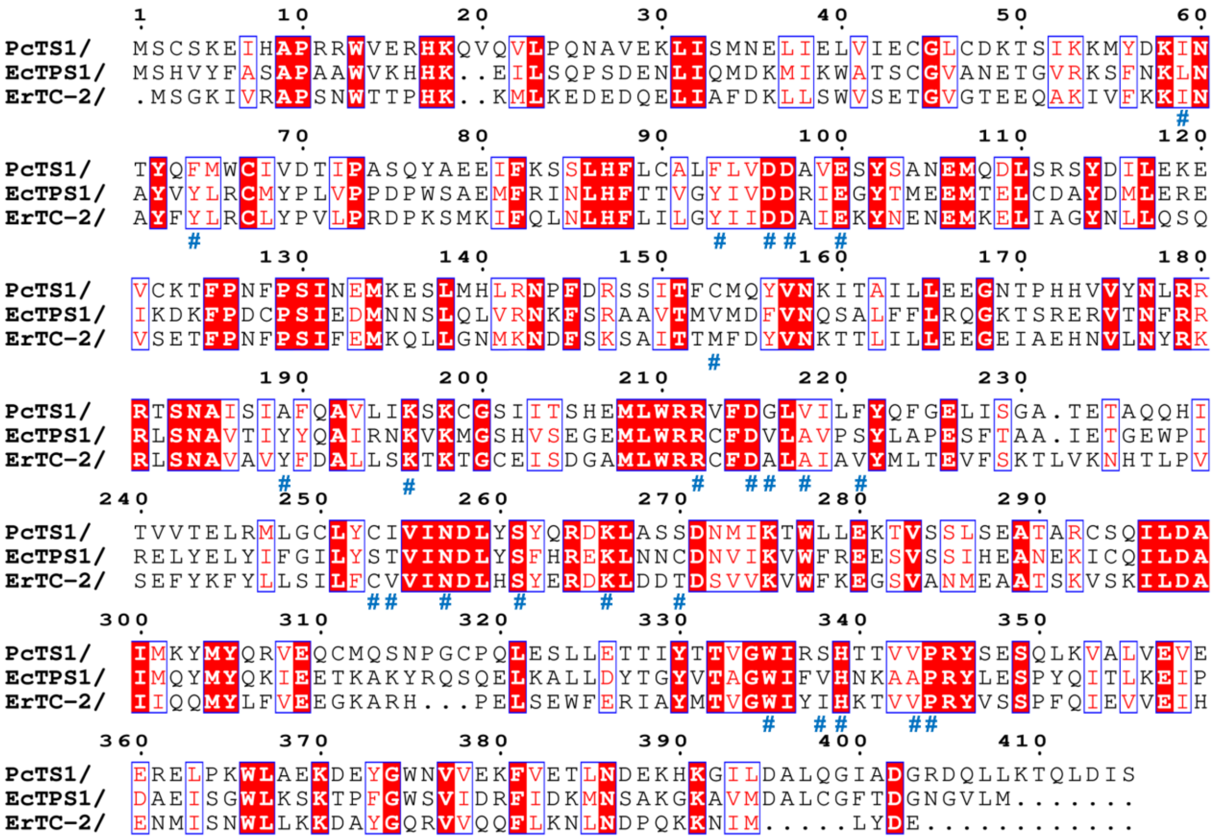

Fig. S12. Sequence alignment of *Pc*TS1 with *Ec*TPS1, and *Er*TC-2.

The key amino acid residues (# tag) of *Pc*TS1 were mutated to align with the amino acid positions in *Er*TC-2 and *Ec*TPS1.

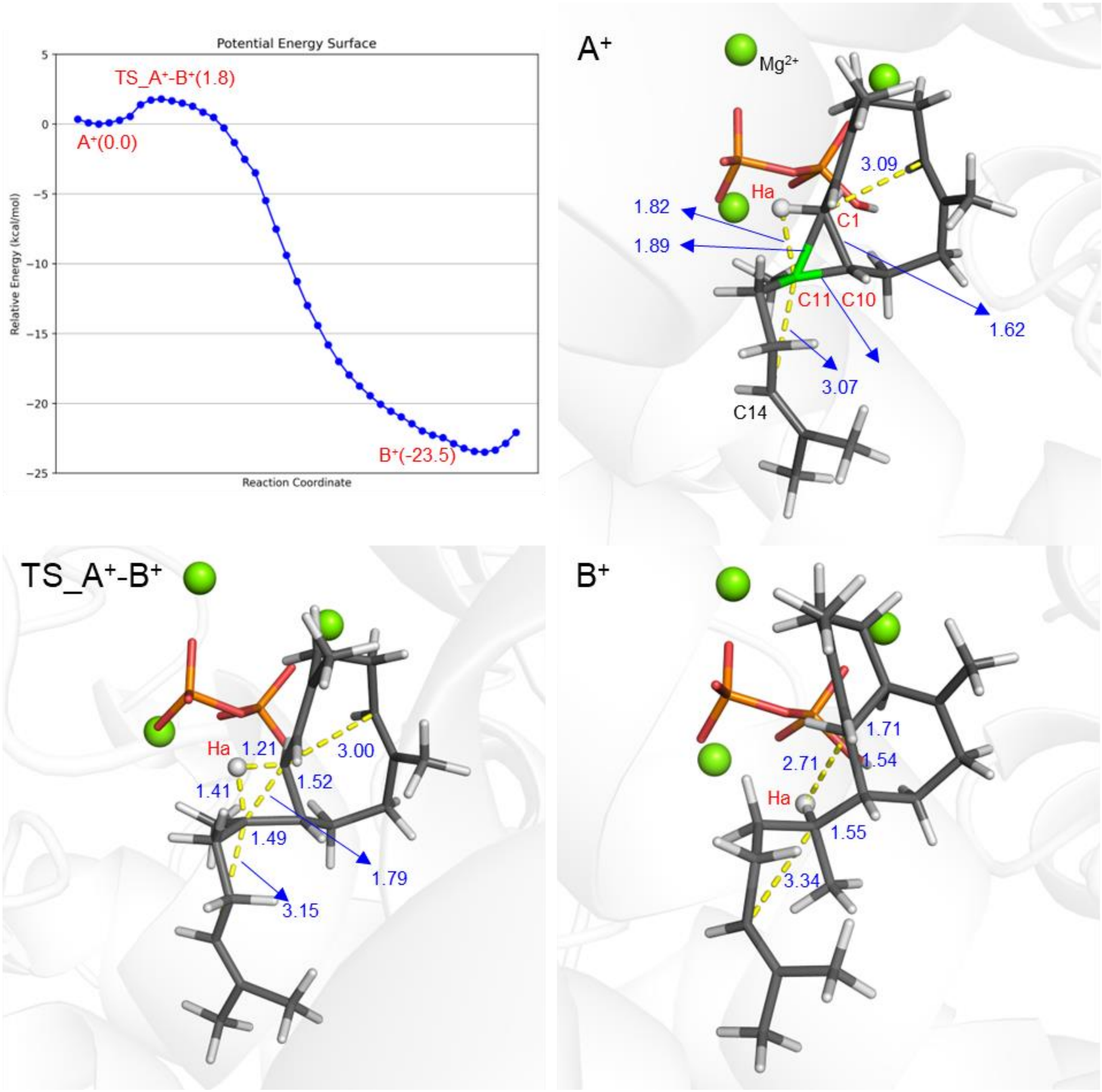

Fig. S13. The potential energy profile and key structures of intermediates A^+-B^+ in *Pc*TS1. The distances are given in Å.

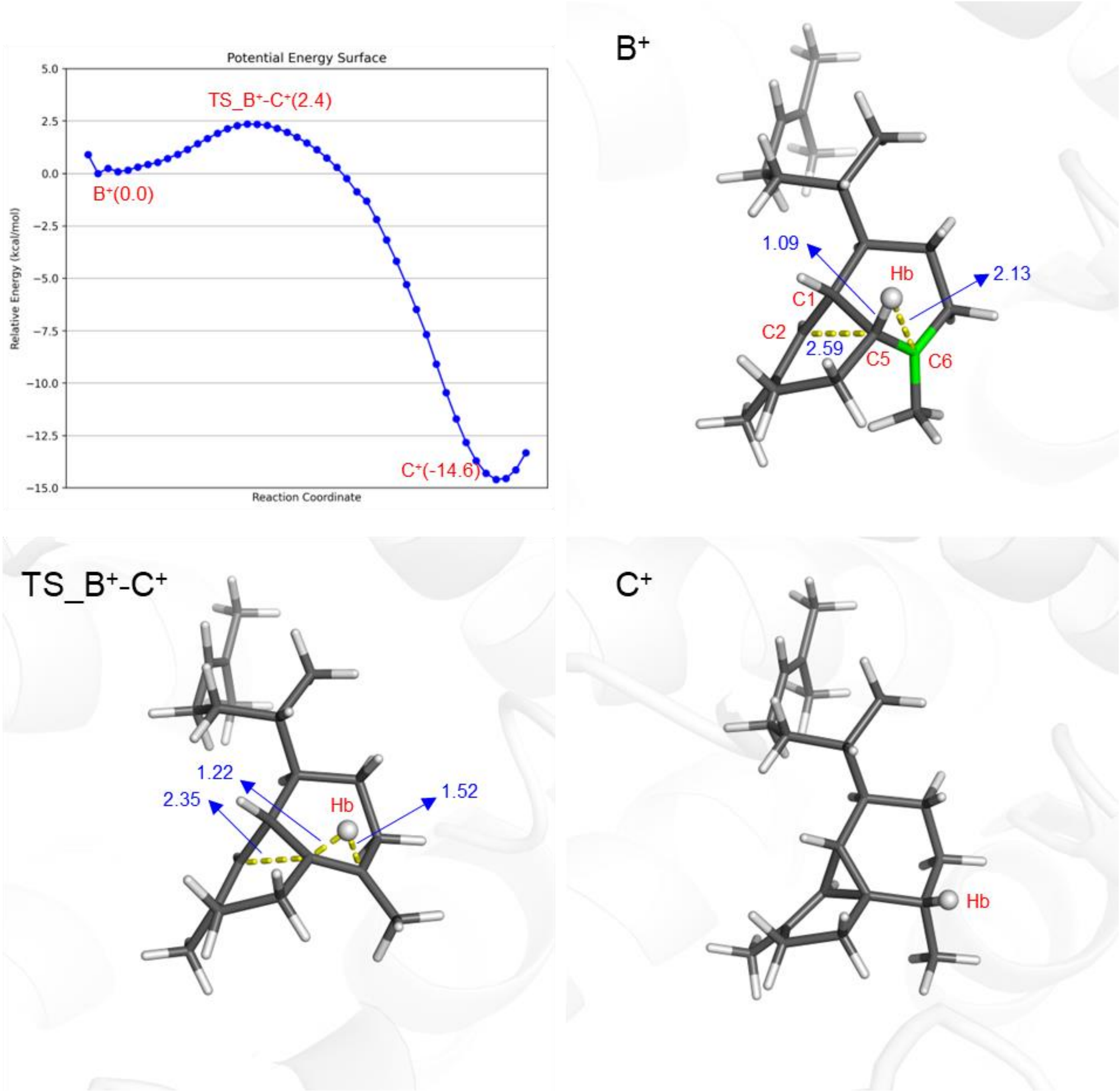

Fig. S14. The potential energy profile and key structures of intermediates B^+-C^+ in *Pc*TS1. The distances are given in Å.

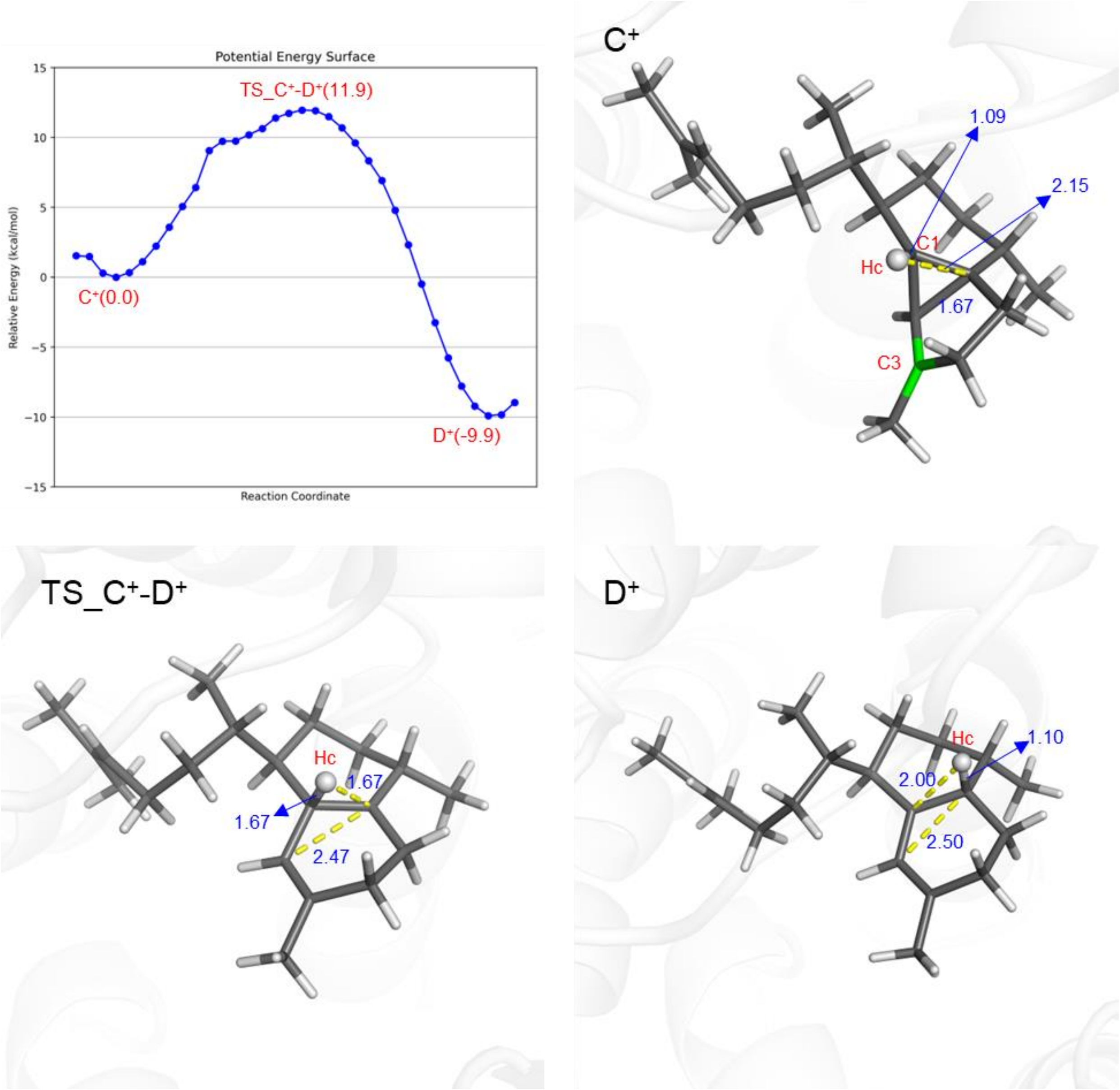

Fig. S15. The potential energy profile and key structures of intermediates C^{+}-D^{+} in *Pc*TS1. The distances are given in Å.

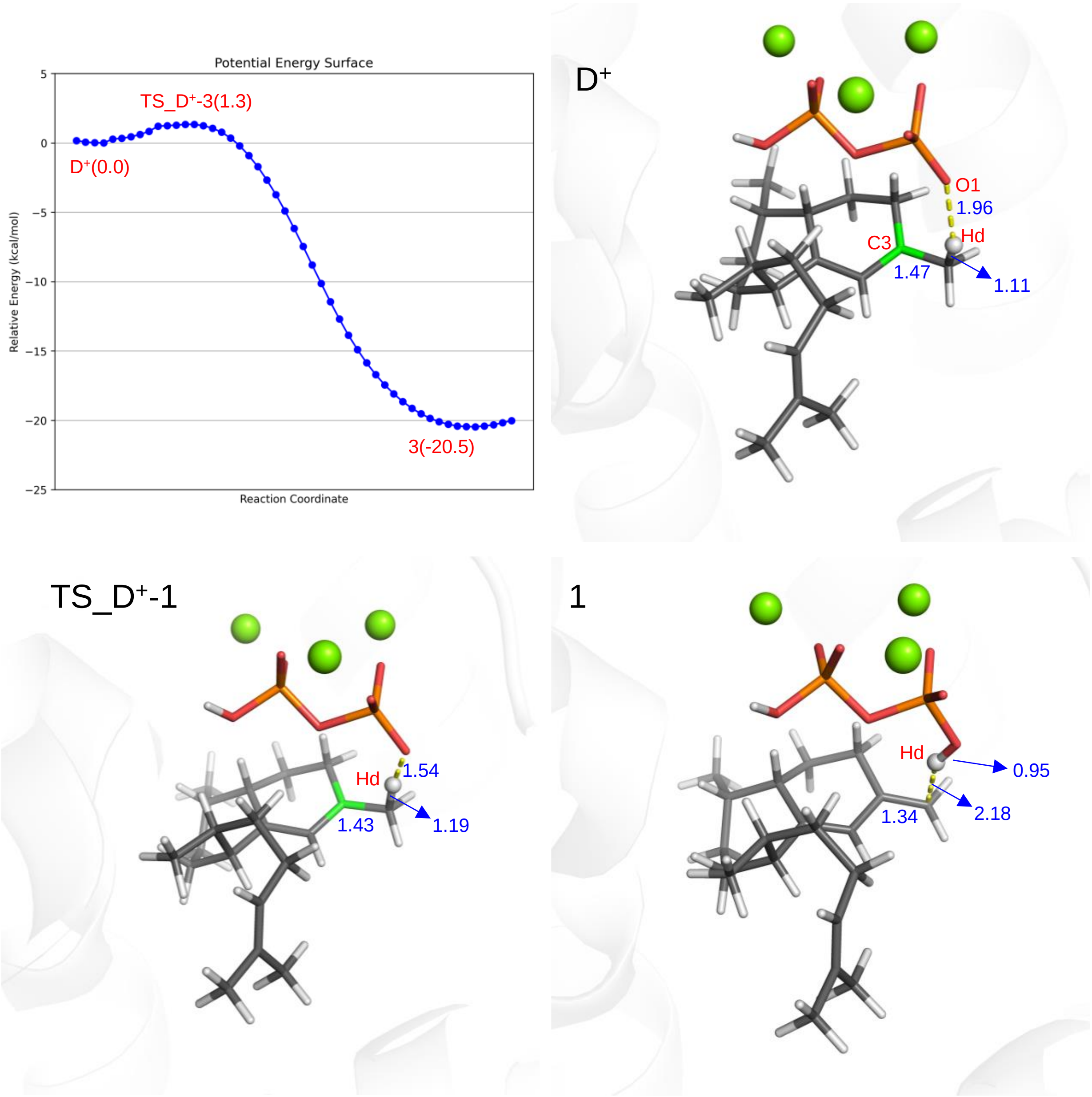

Fig. S16. The potential energy profile and key structures of intermediates D^{+}-1 in *Pc*TS1. The distances are given in Å.

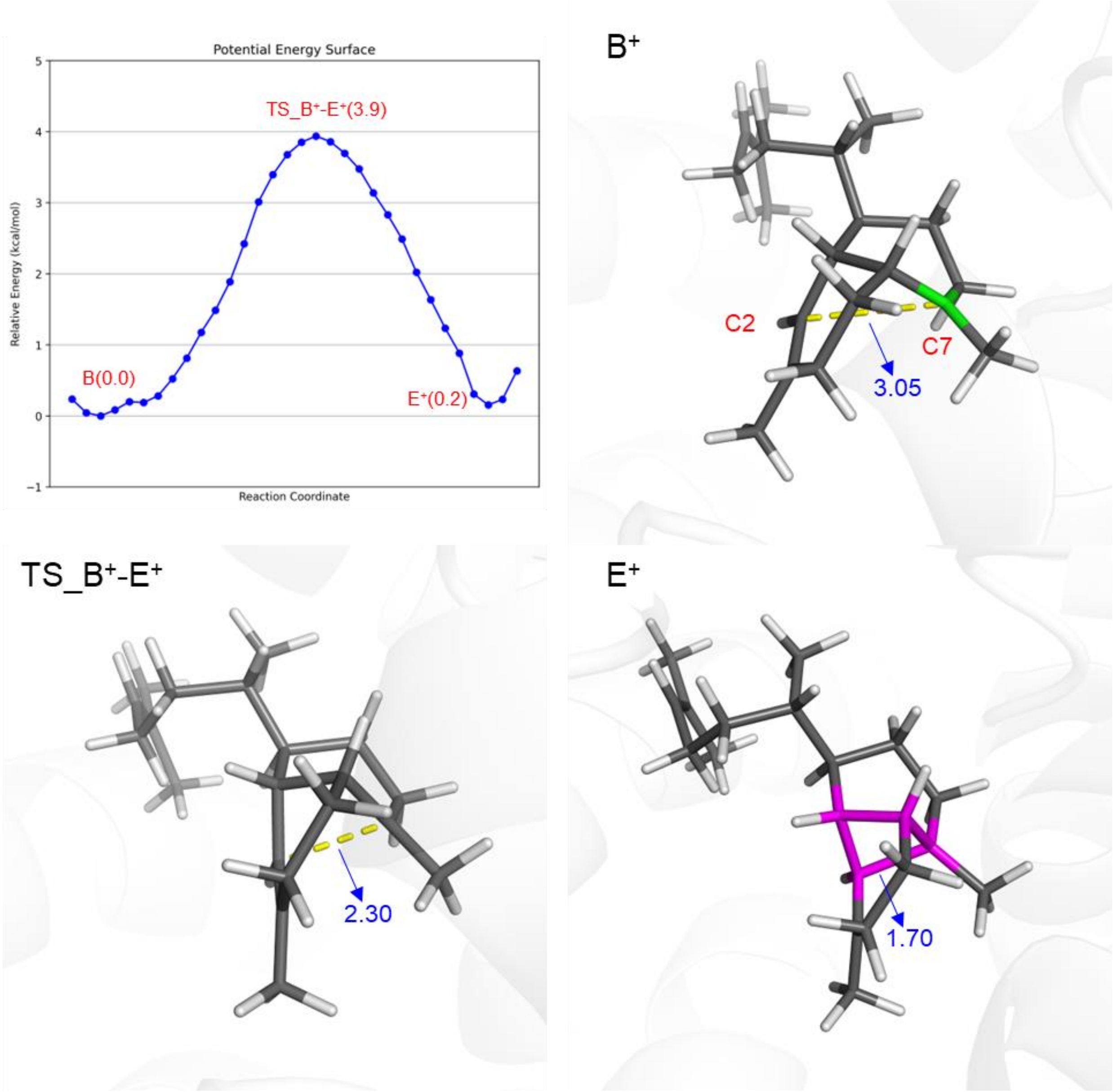

Fig. S17. The potential energy profile and key structures of intermediates B^+-E^+ in *Pc*TS1. The distances are given in Å.

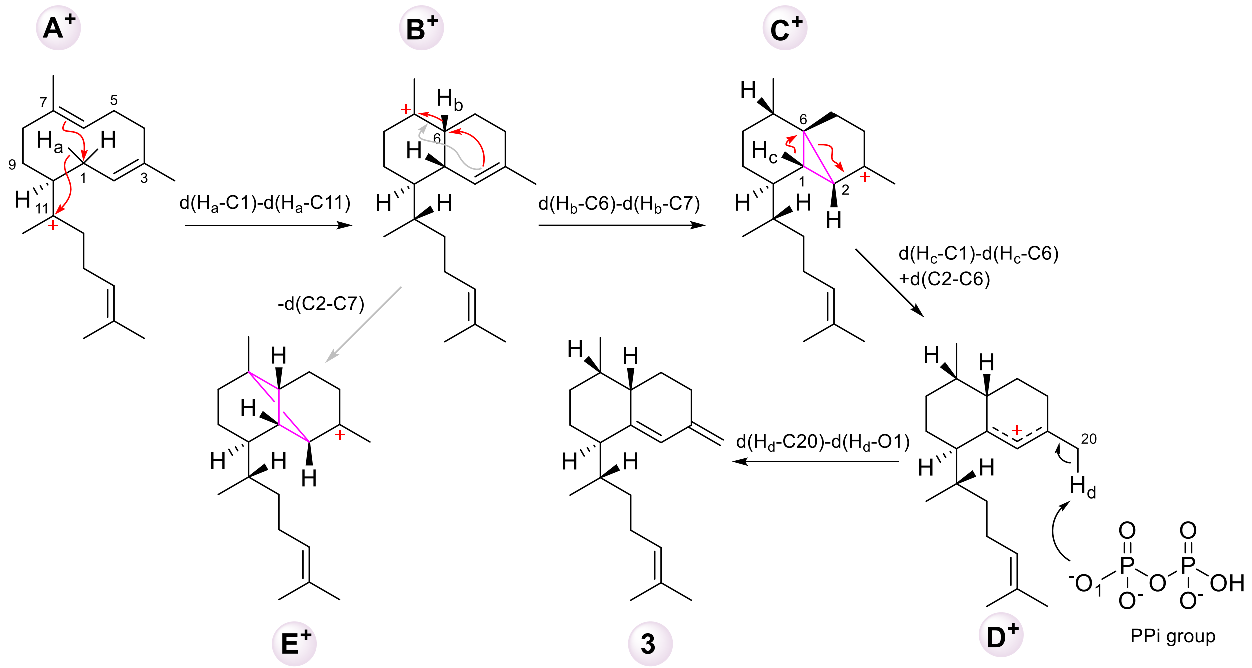

Fig. S18. The definition of reaction coordinates during the PES calculations.

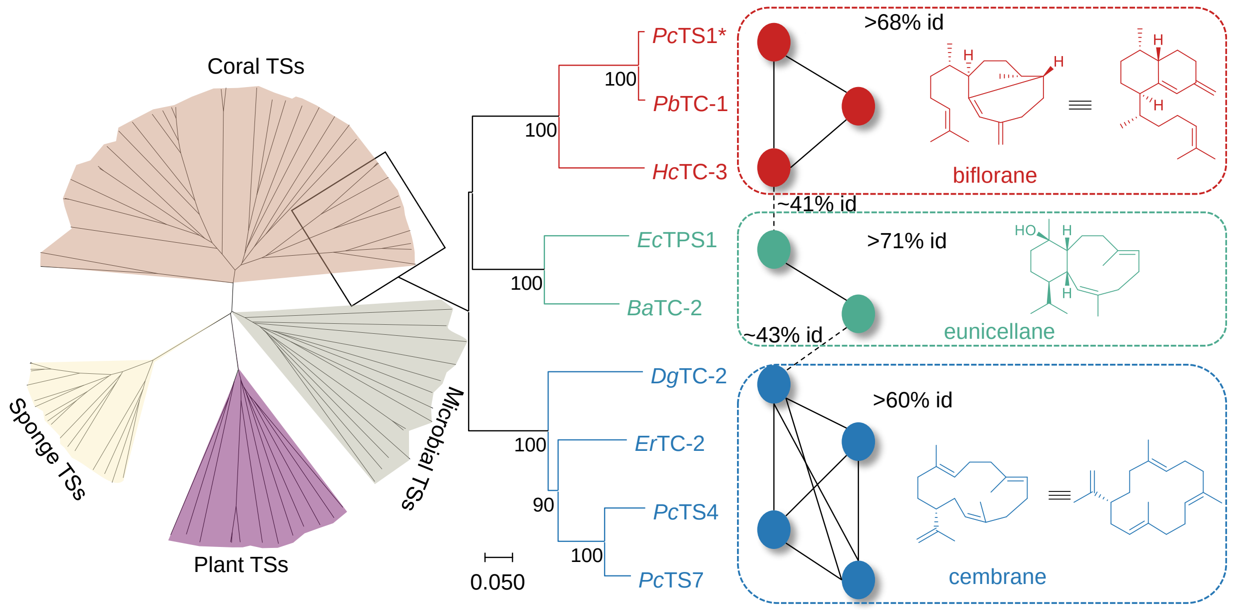

Fig. S19. Phylogenetic analysis of TSs currently identified from marine invertebrates.
Phylogenetic analysis of TSs currently identified from marine invertebrates using the neighbor-joining method. Some TSs from microorganisms and plants were added to enhance the completeness of the neighbor-joining tree, TSs responsible for the biosynthesis of biflorane, cembrane, and eunicellane skeletons exhibit close phylogenetic relationships, suggesting a potential evolutionary connection.

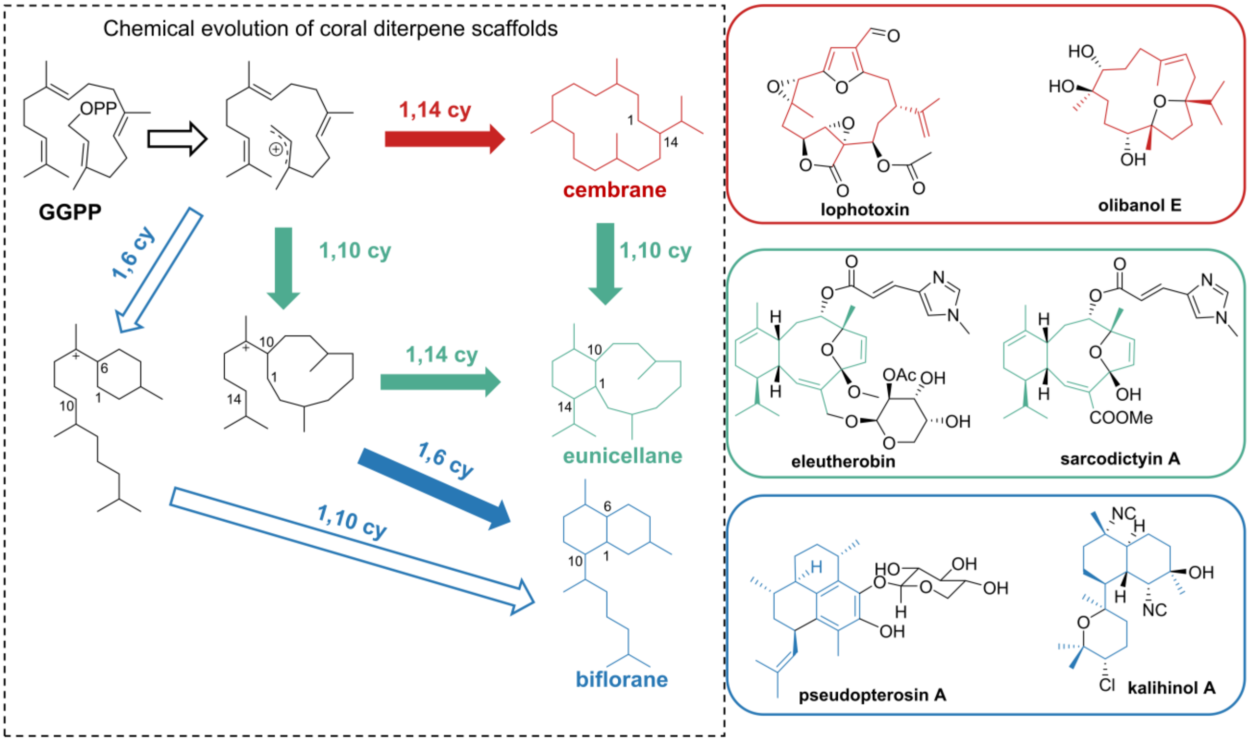

Fig. S20. Potential chemical evolution pathways of cembrane, eunicellane, and biflorane (serrulatane) diterpenoids, along with their associated bioactive compounds.

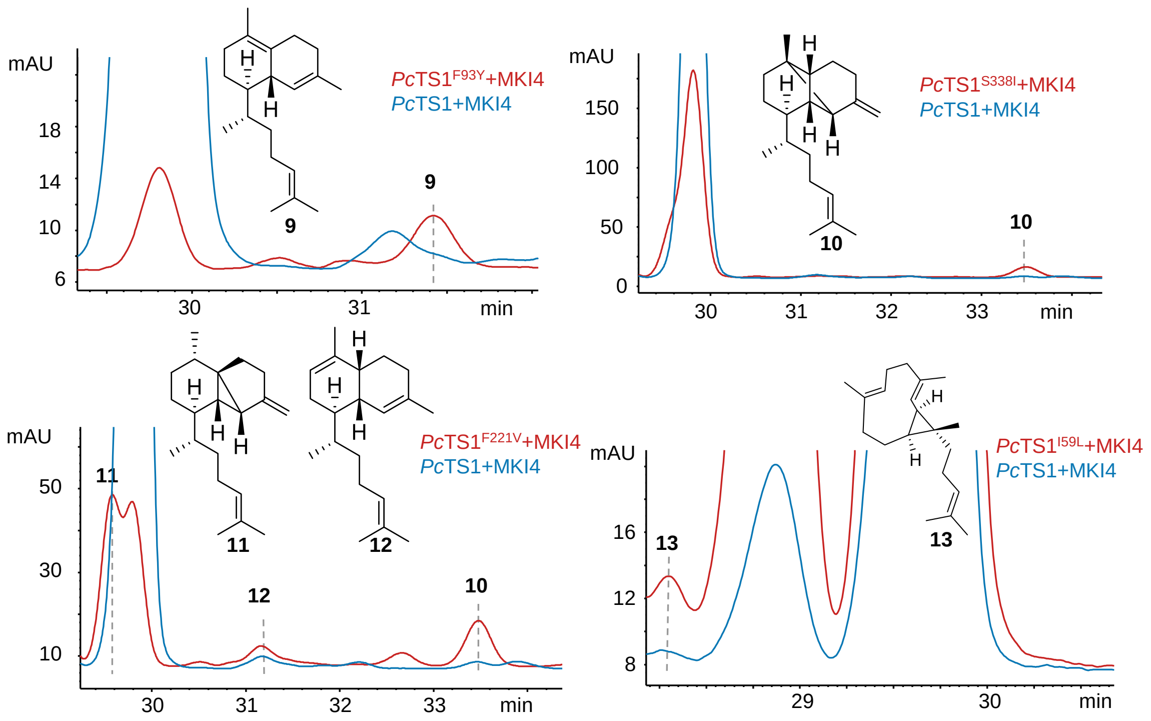

Fig. S21. HPLC analysis of fermentation broth of mutants *Pc*TS1^{F93Y}, *Pc*TS1^{S338I}, *Pc*TS1^{F221V}, and *Pc*TS1^{I59L}.

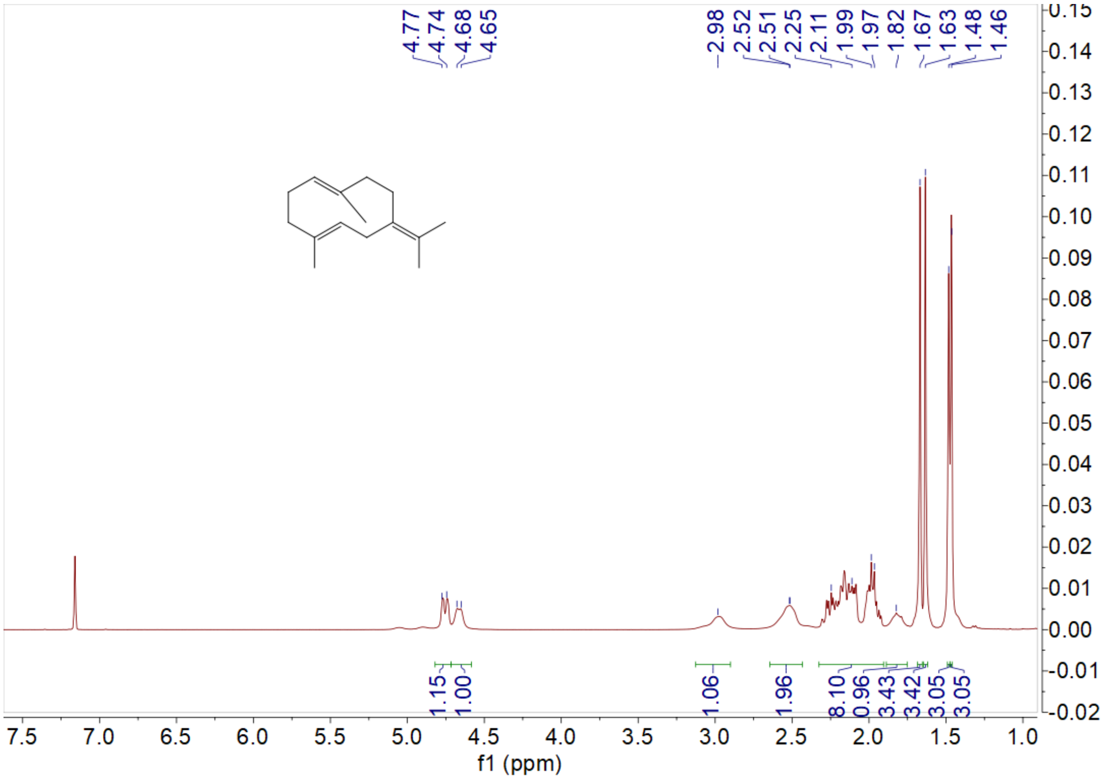

Fig. S22. ^{1}H NMR spectrum (400 MHz) of germacrene B in C_6D_6.

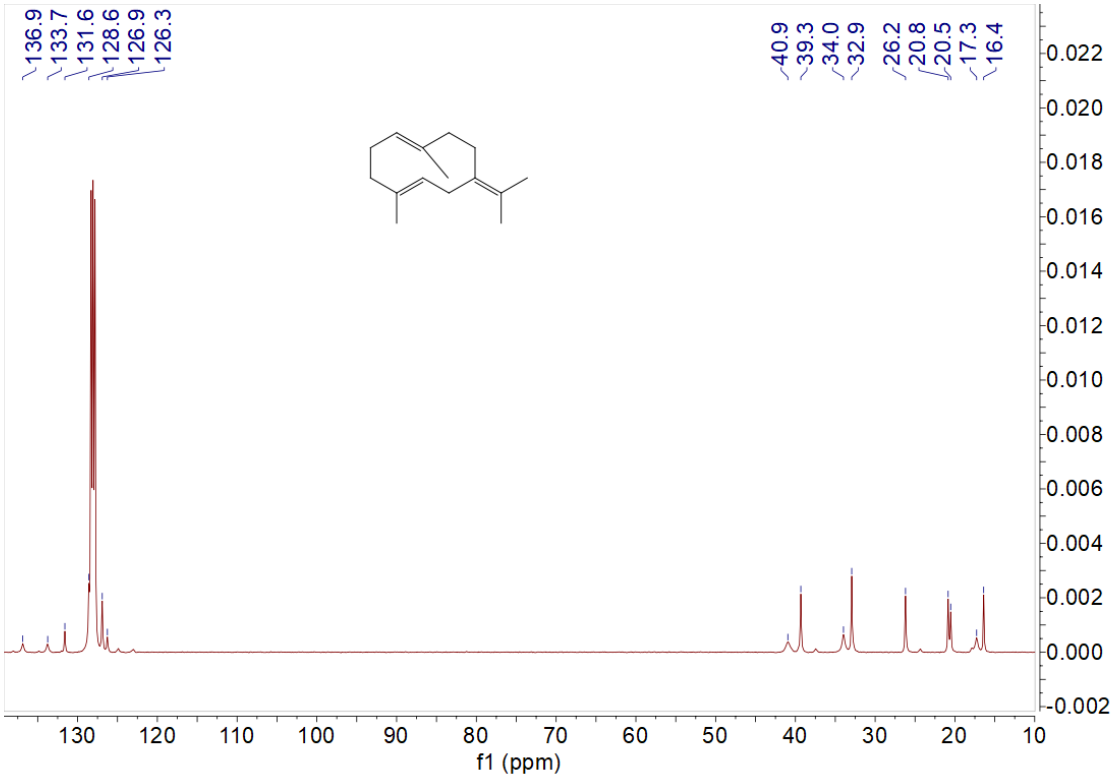

Fig. S23. ^{13}C NMR spectrum (100 MHz) of germacrene B in C_6D_6.

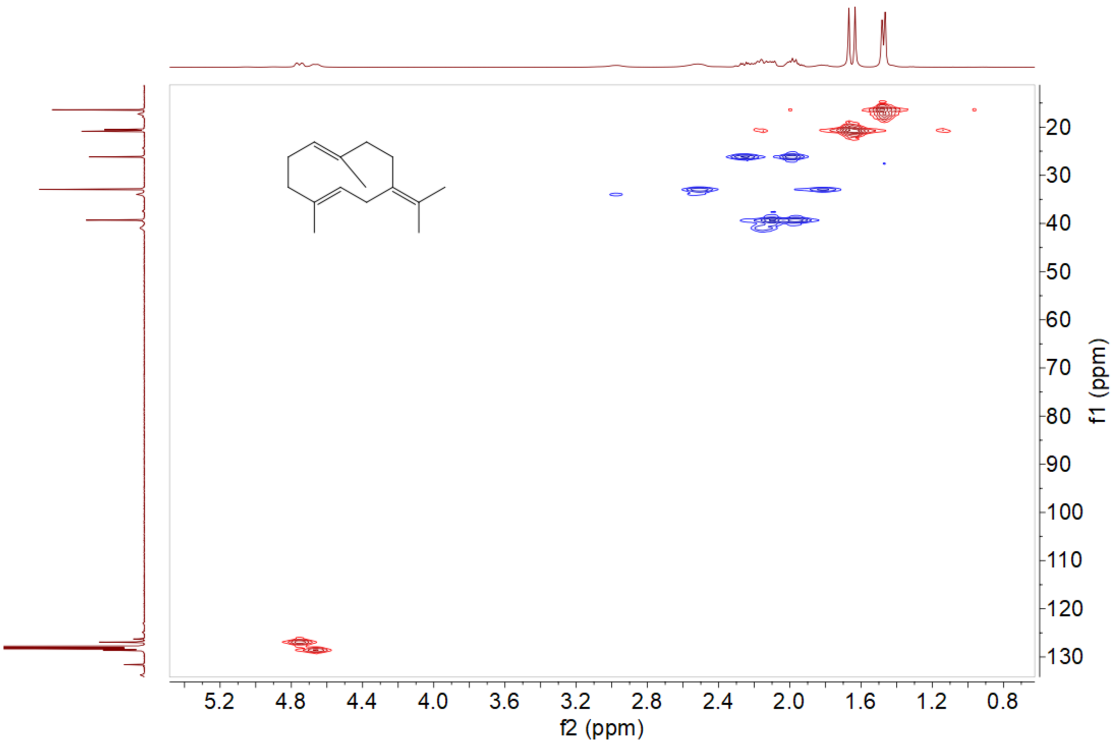

Fig. S24. HSQC NMR spectrum of germacrene B in C_6D_6.

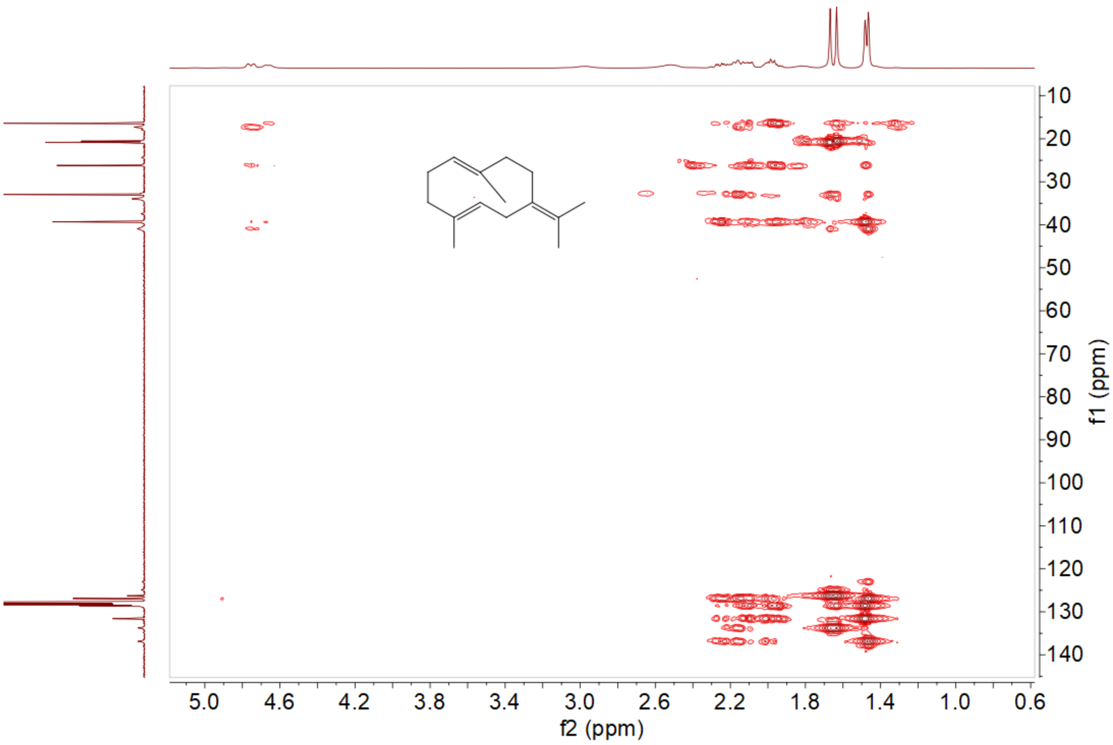

Fig. S25. HMBC NMR spectrum of germacrene B in C_6D_6.

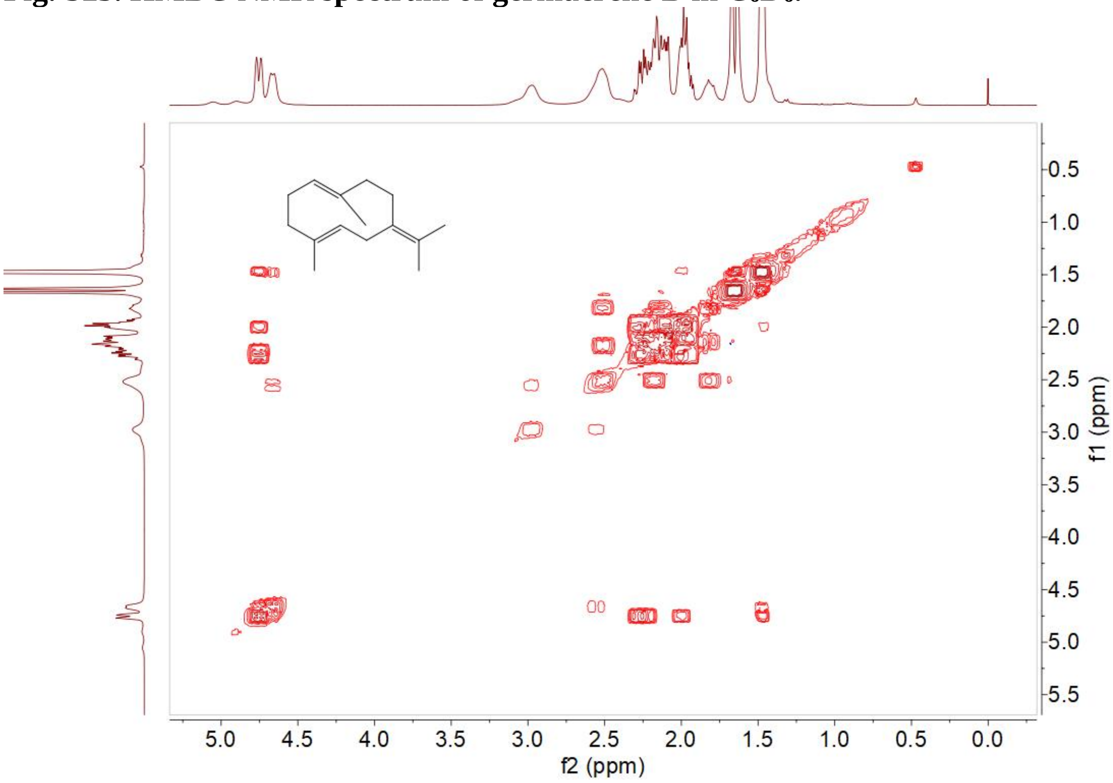

Fig. S26. ^1H-^1H COSY NMR spectrum of germacrene B in C_6D_6.

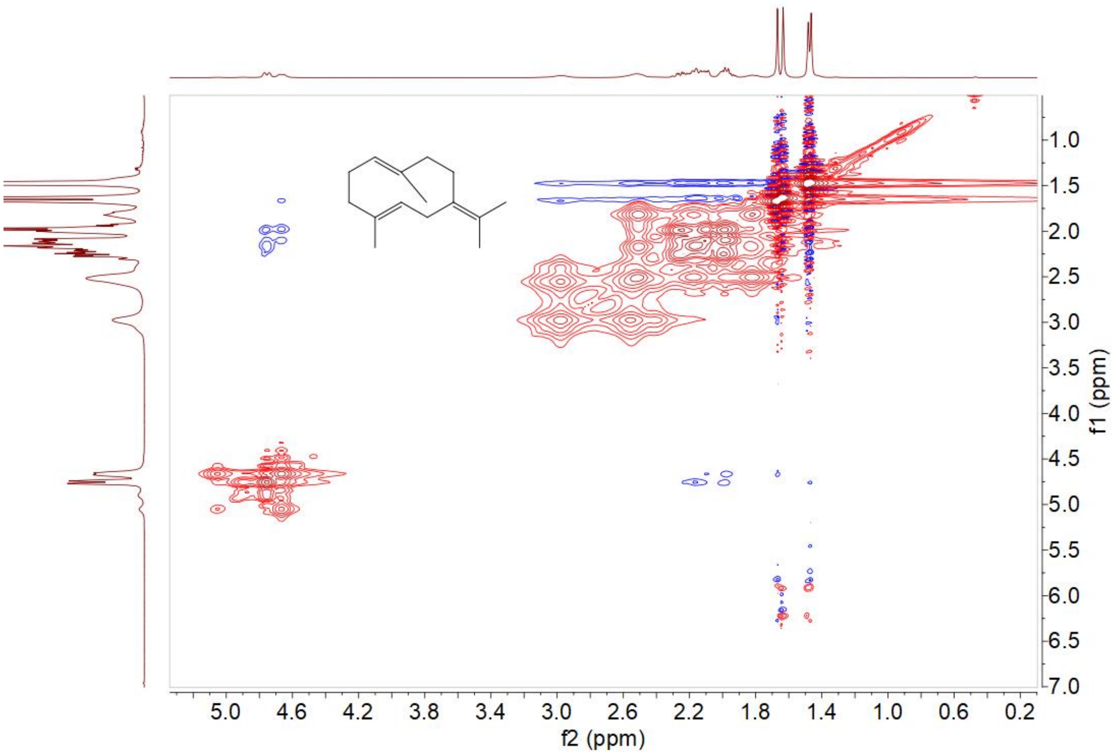

Fig. S27. NOESY NMR spectrum of germacrene B in C_6D_6.

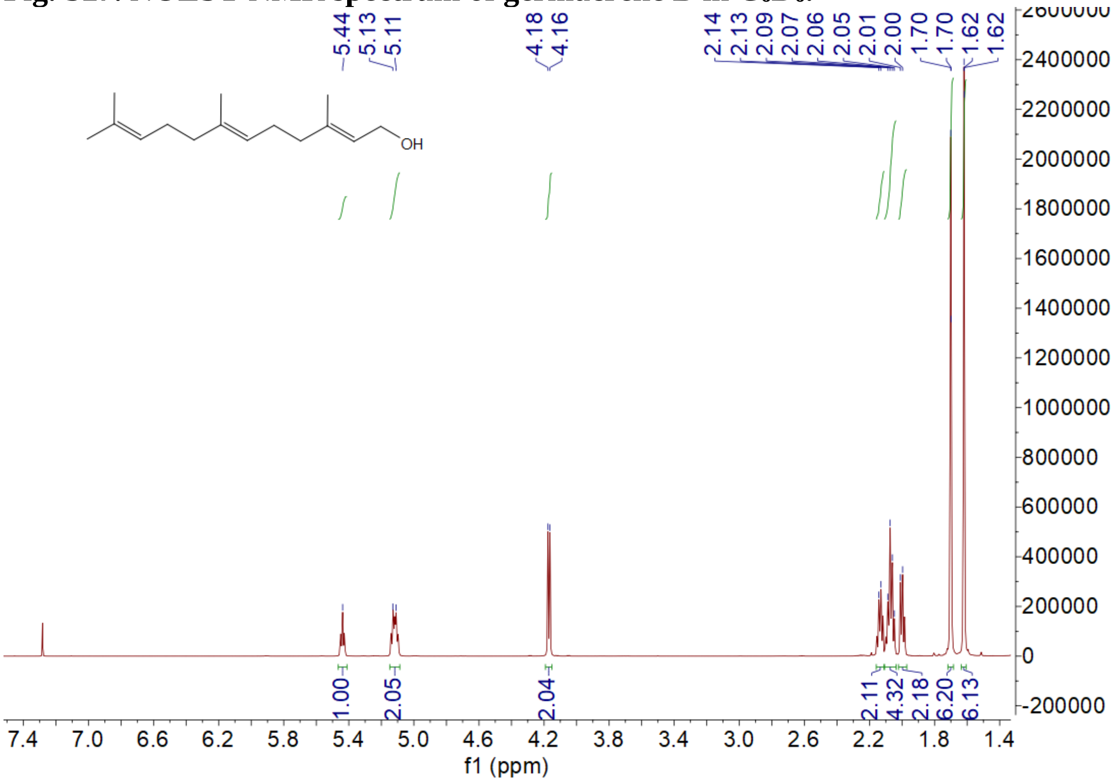

Fig. S28. ^{1}H NMR spectrum (600 MHz) of farnesol in $CDCl_3$.

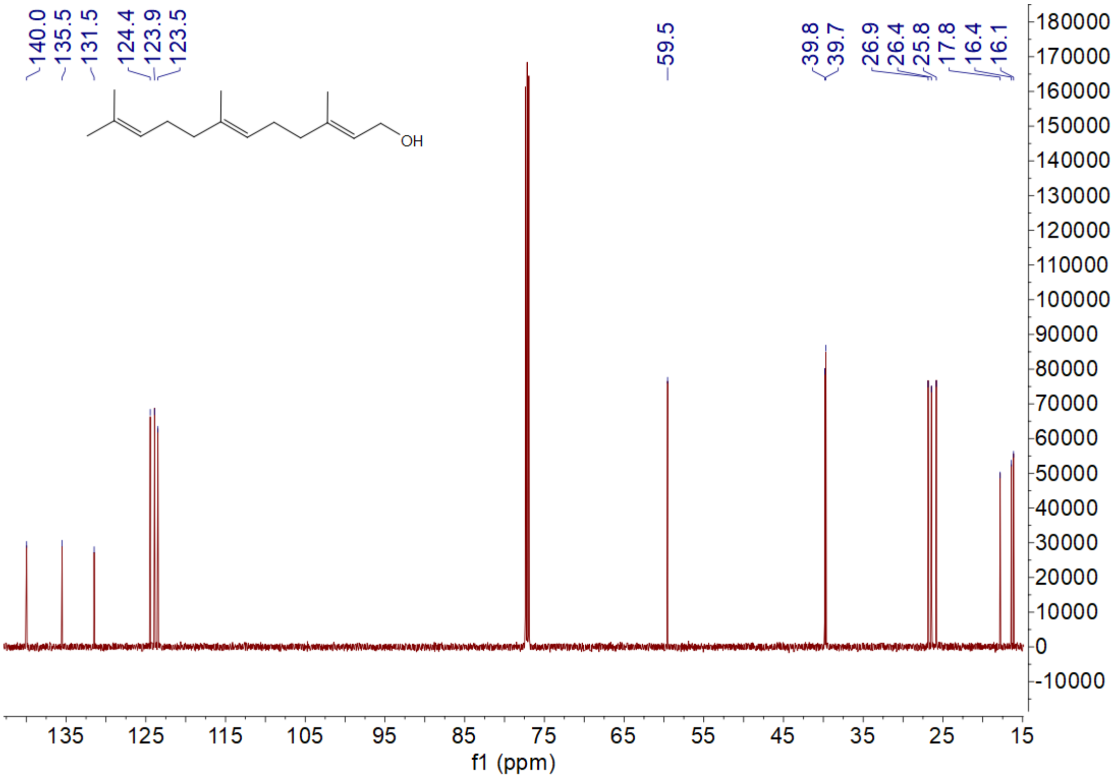

Fig. S29. ^{13}C NMR spectrum (150 MHz) of farnesol in $CDCl_3$.

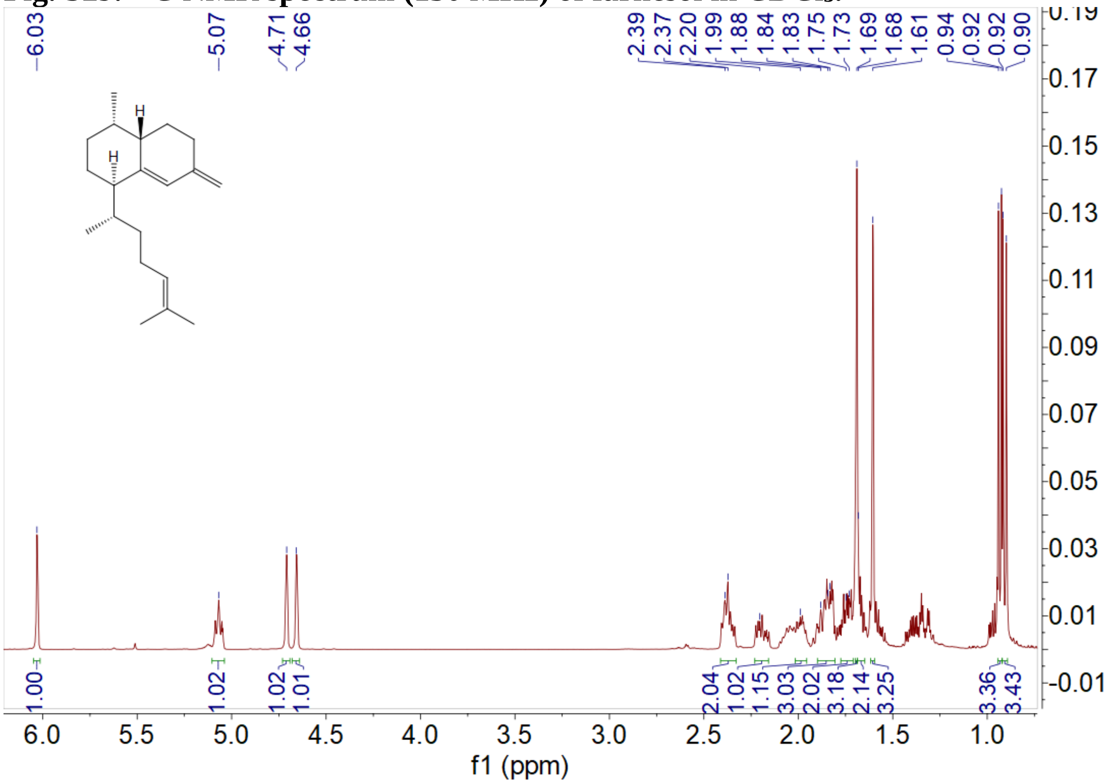

Fig. S30. ^{1}H NMR spectrum (400 MHz) of elisabethatriene (1) in $CDCl_3$.

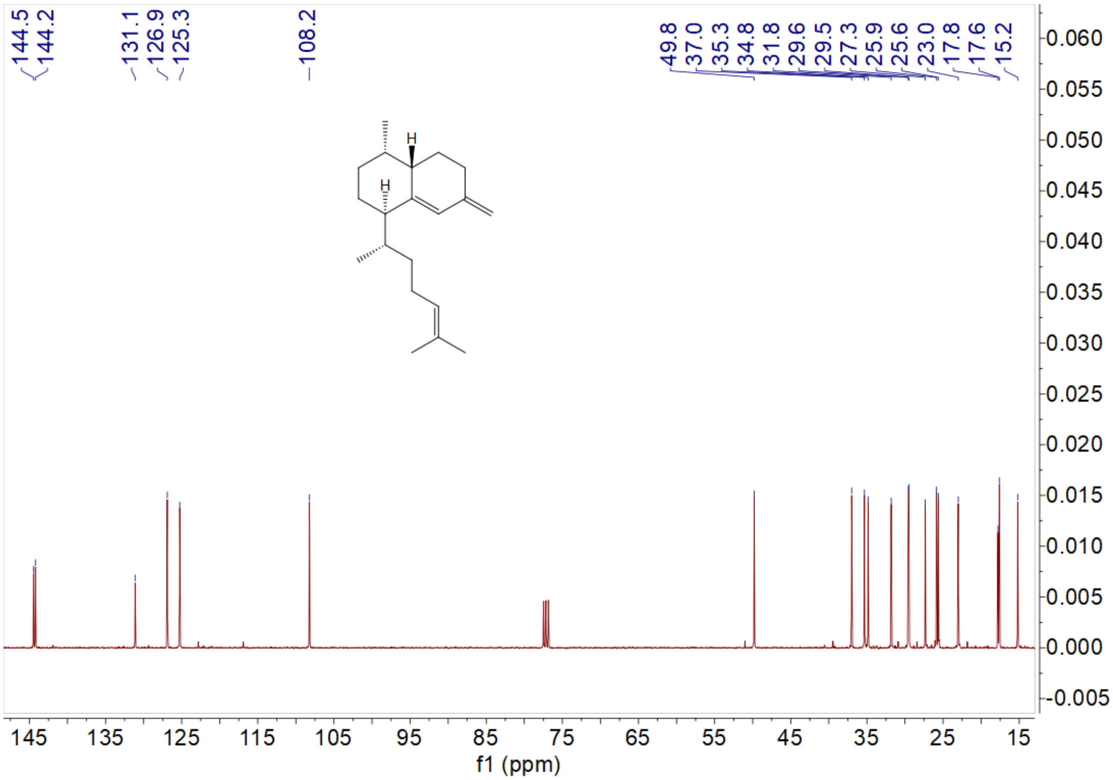

Fig. S31. ^{13}C NMR spectrum (100 MHz) of elisabethatriene (1) in $CDCl_3$.

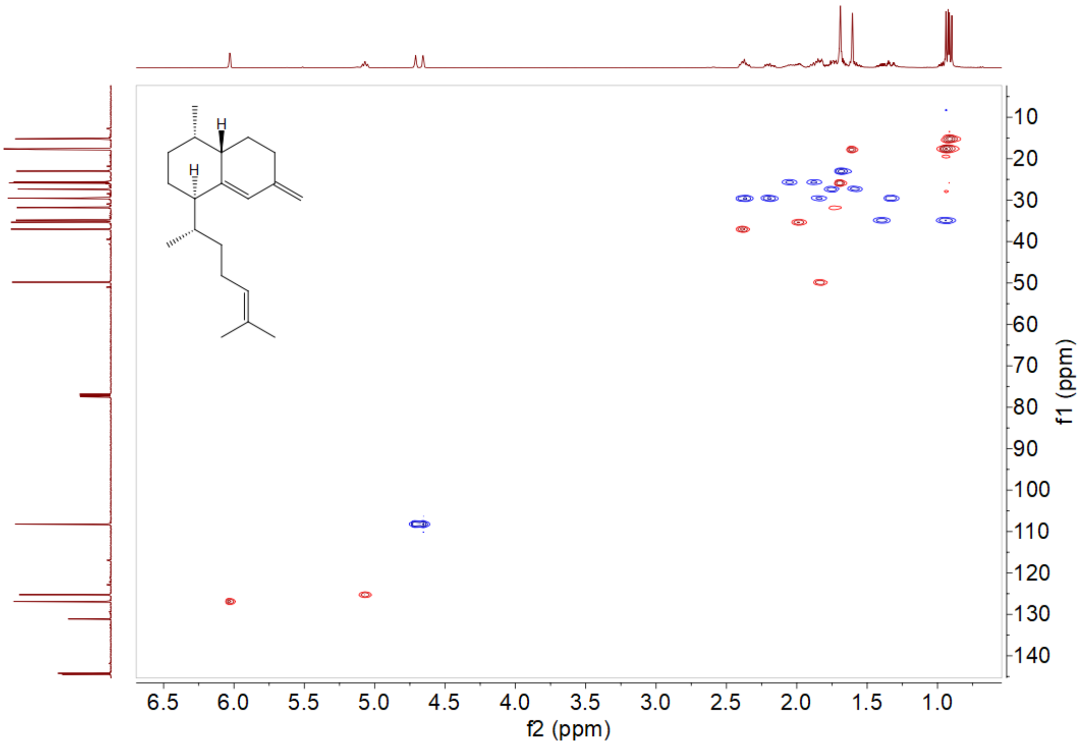

Fig. S32. HSQC NMR spectrum of elisabethatriene (1) in $CDCl_3$.

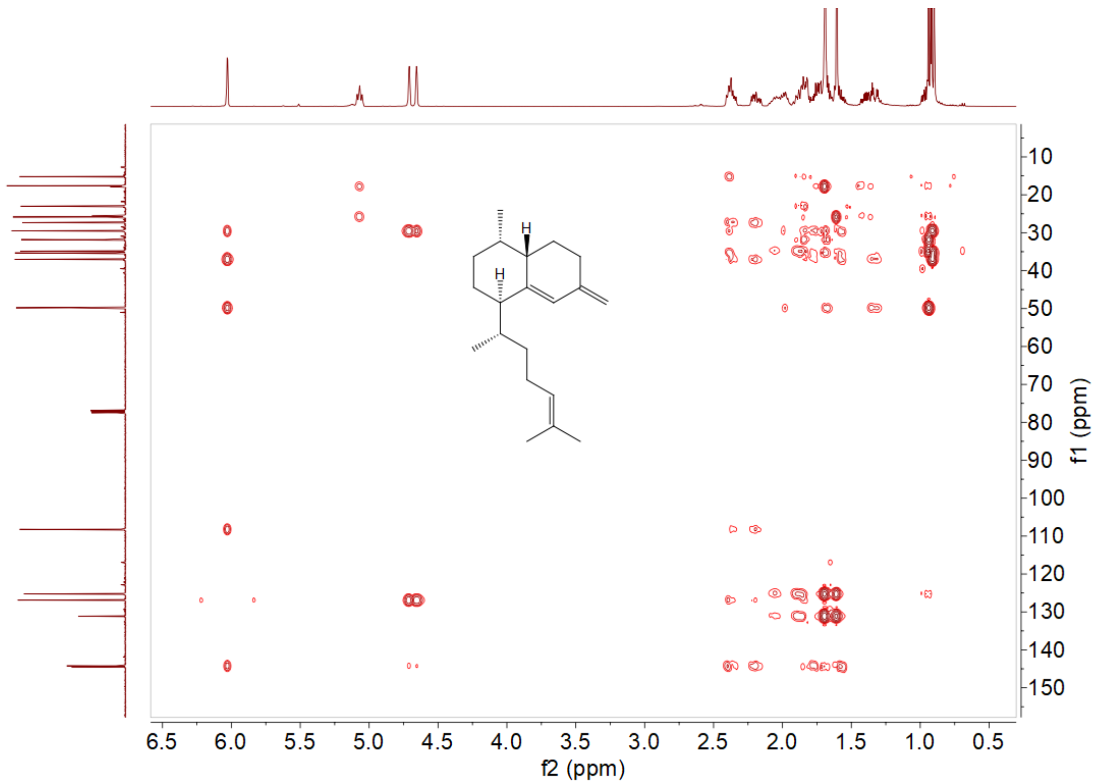

Fig. S33. HMBC NMR spectrum of elisabethatriene (1) in $CDCl_3$.

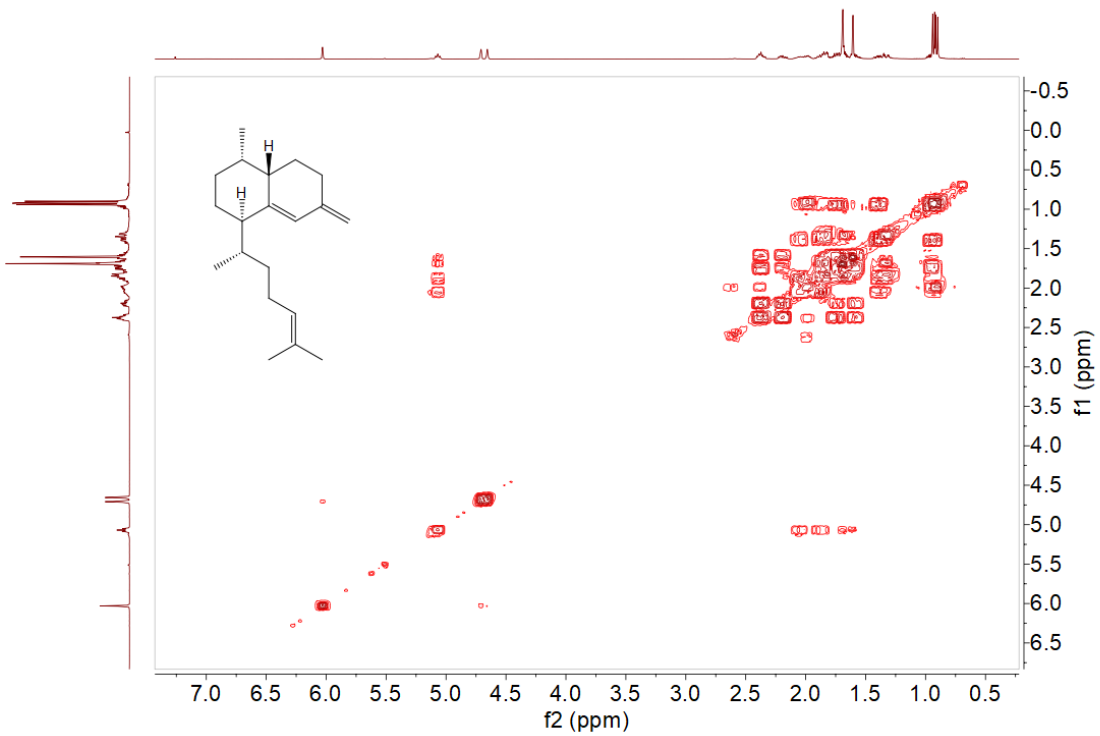

Fig. S34. ^1H-^1H COSY NMR spectrum of elisabethatriene (1) in $CDCl_3$.

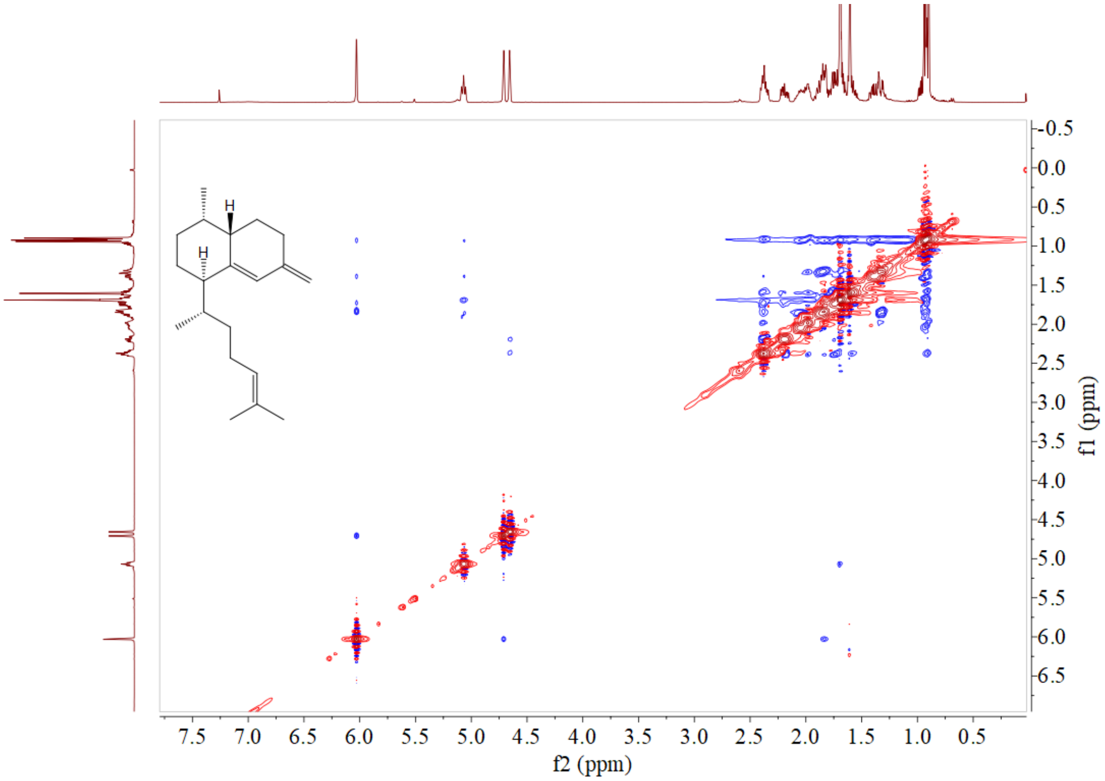

Fig. S35. NOESY NMR spectrum of elisabethatriene (1) in $CDCl_3$.

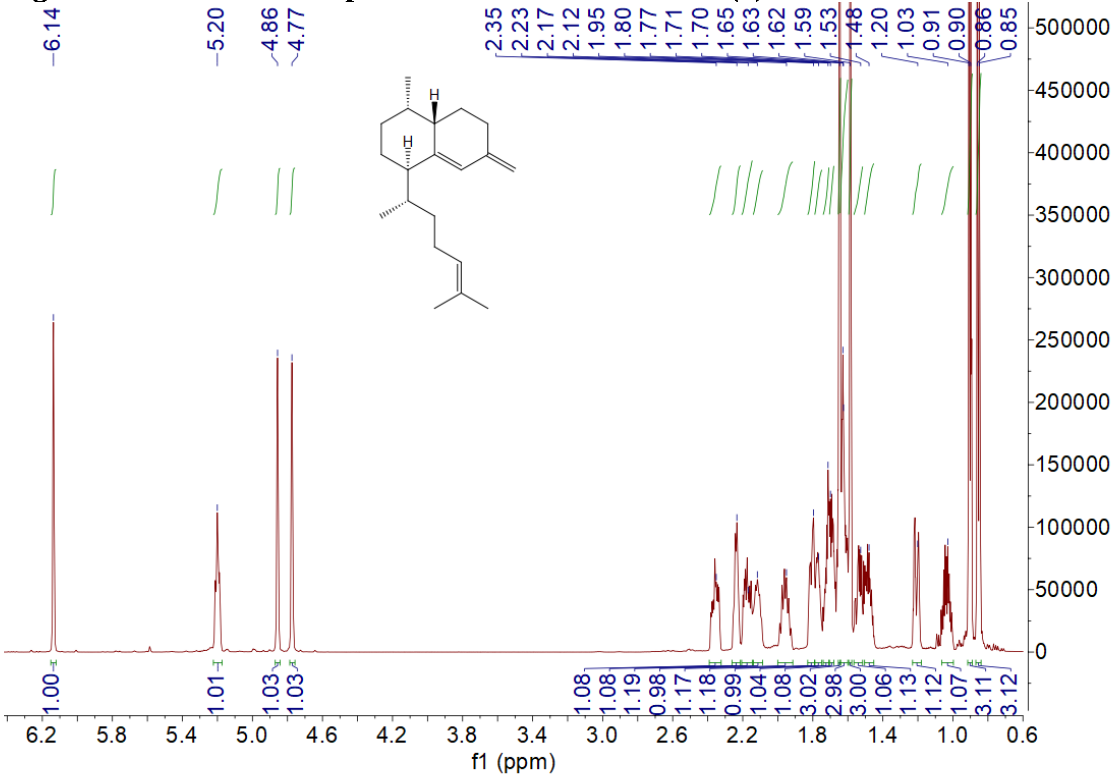

Fig. S36. ^{1}H NMR spectrum (400 MHz) of elisabethatriene (1) in C_6D_6.

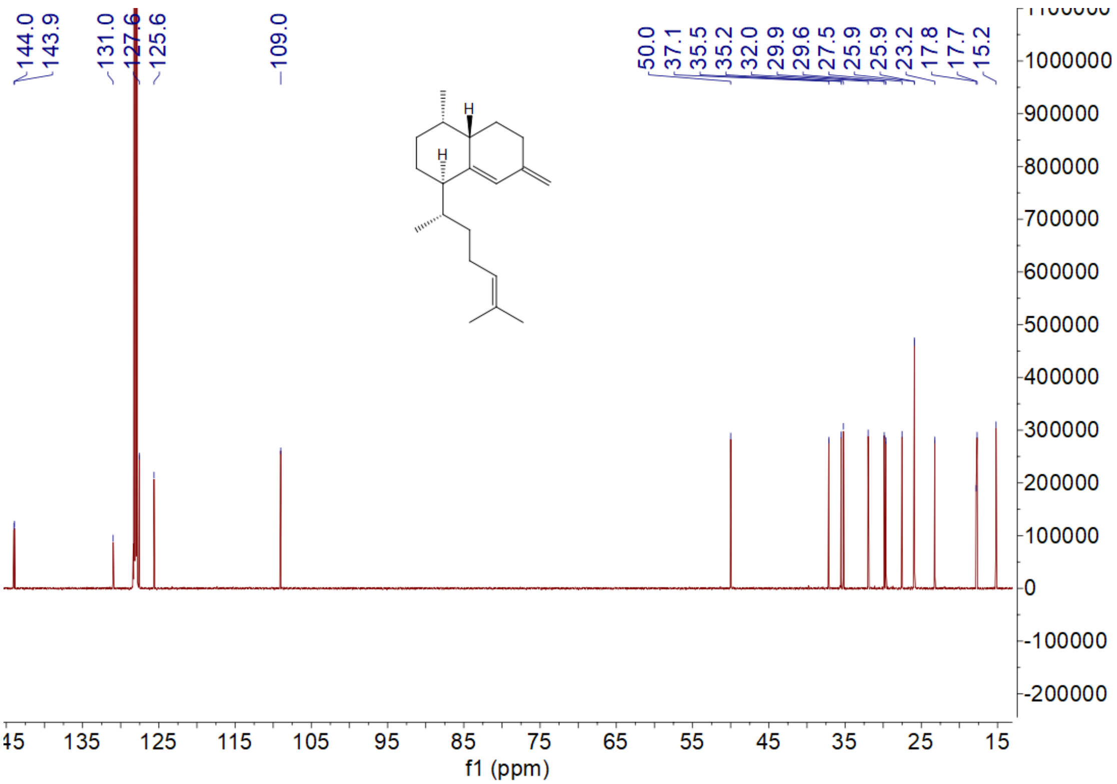

Fig. S37. ^{13}C NMR spectrum (100 MHz) of elisabethatriene (1) in C_6D_6.

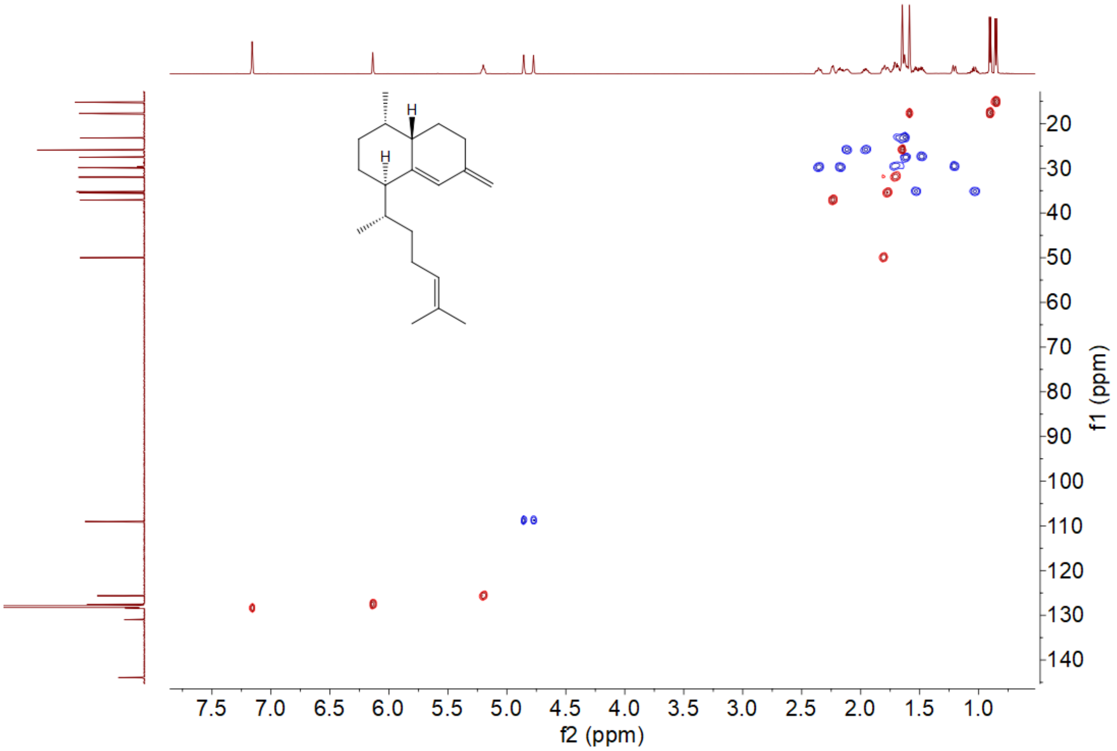

Fig. S38. HSQC NMR spectrum of elisabethatriene (1) in C_6D_6.

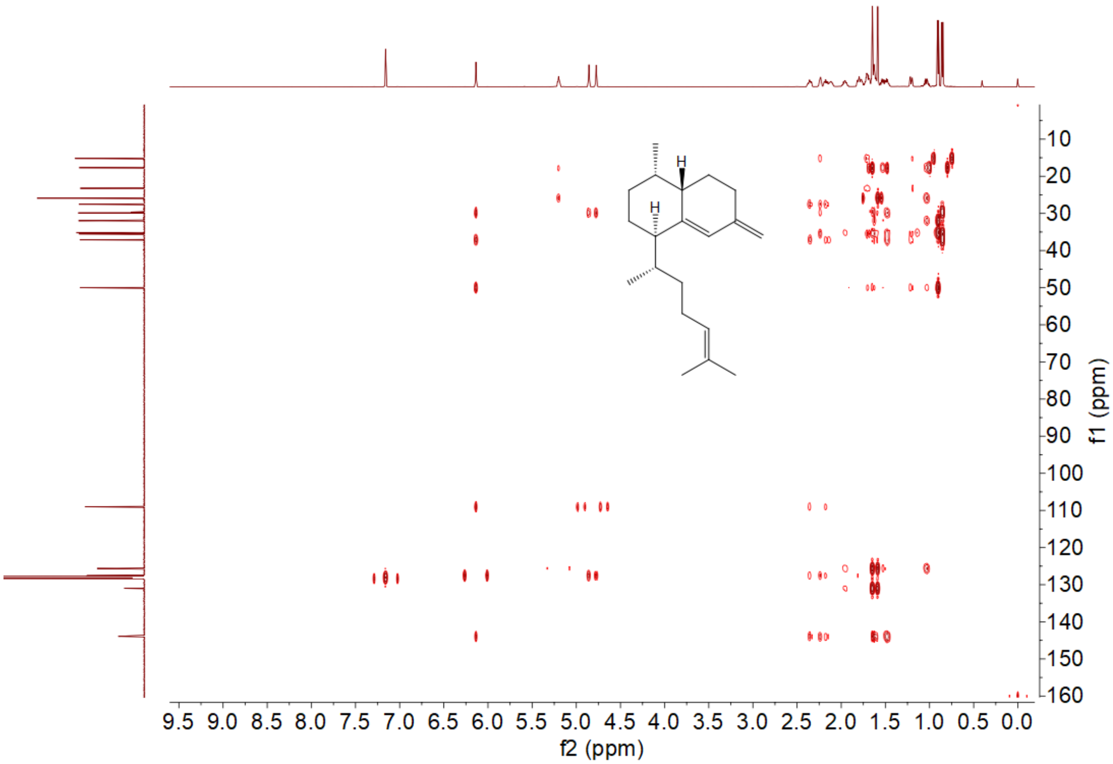

Fig. S39. HMBC NMR spectrum of elisabethatriene (1) in C_6D_6.

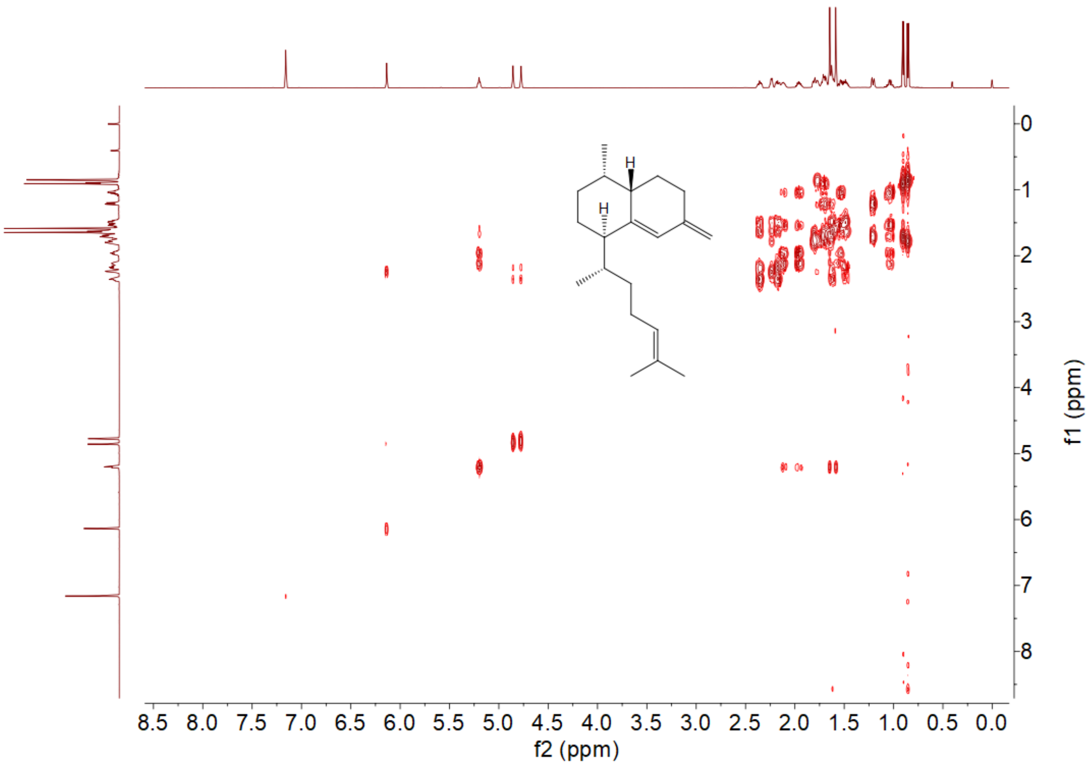

Fig. S40. ^{1}H-^{1}H COSY NMR spectrum of elisabethatriene (1) in C_6D_6.

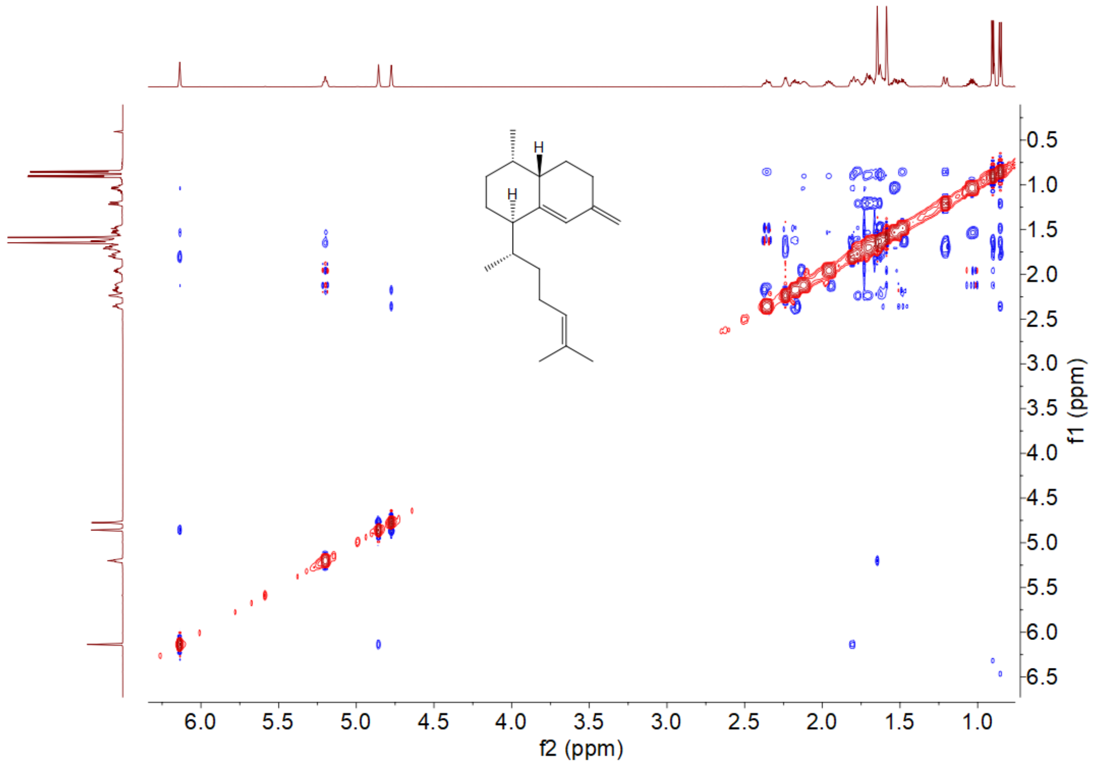

Fig. S41. NOESY NMR spectrum of elisabethatriene (1) in C_6D_6.

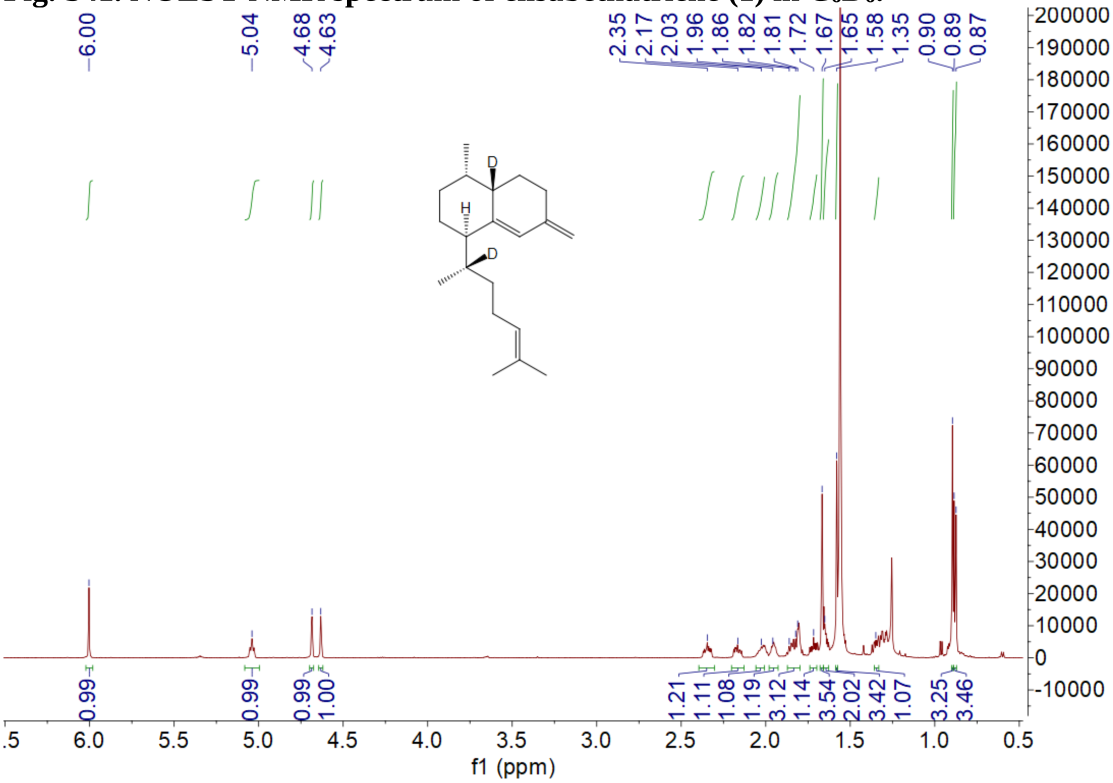

Fig. S42. ^{1}H NMR spectrum (600 MHz) of 4 in $CDCl_3$.

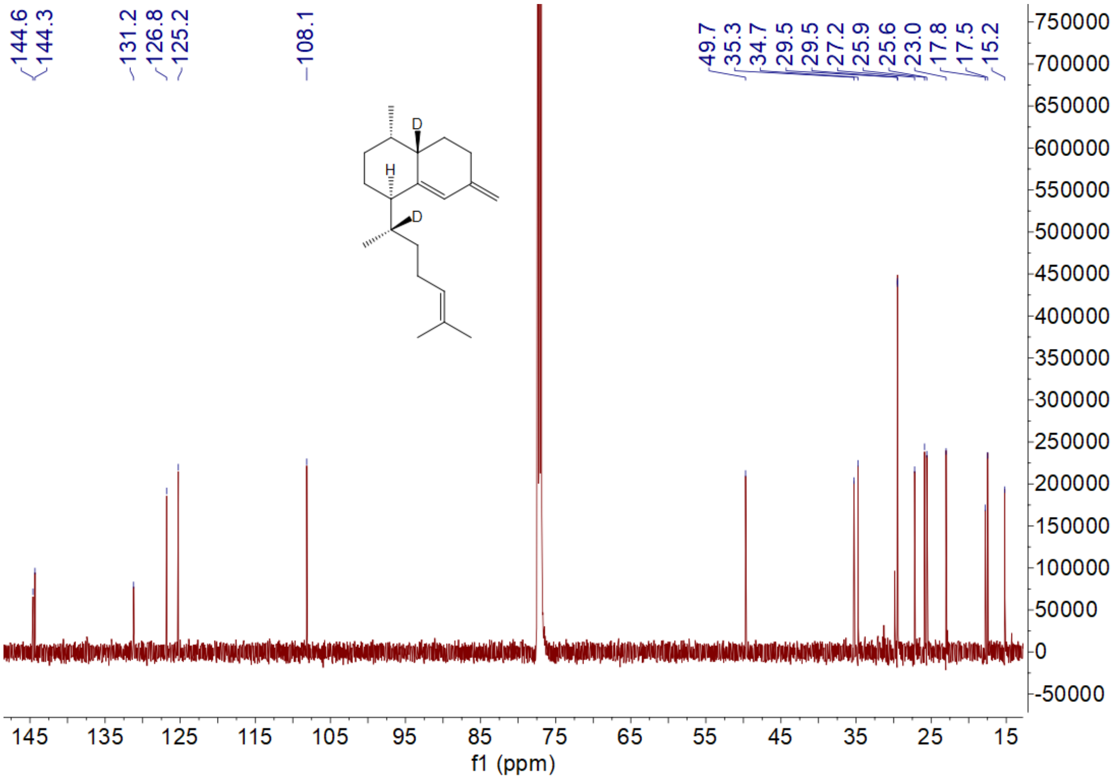

Fig. S43. ^{13}C NMR spectrum (150 MHz) of 4 in CDCl$_3$.

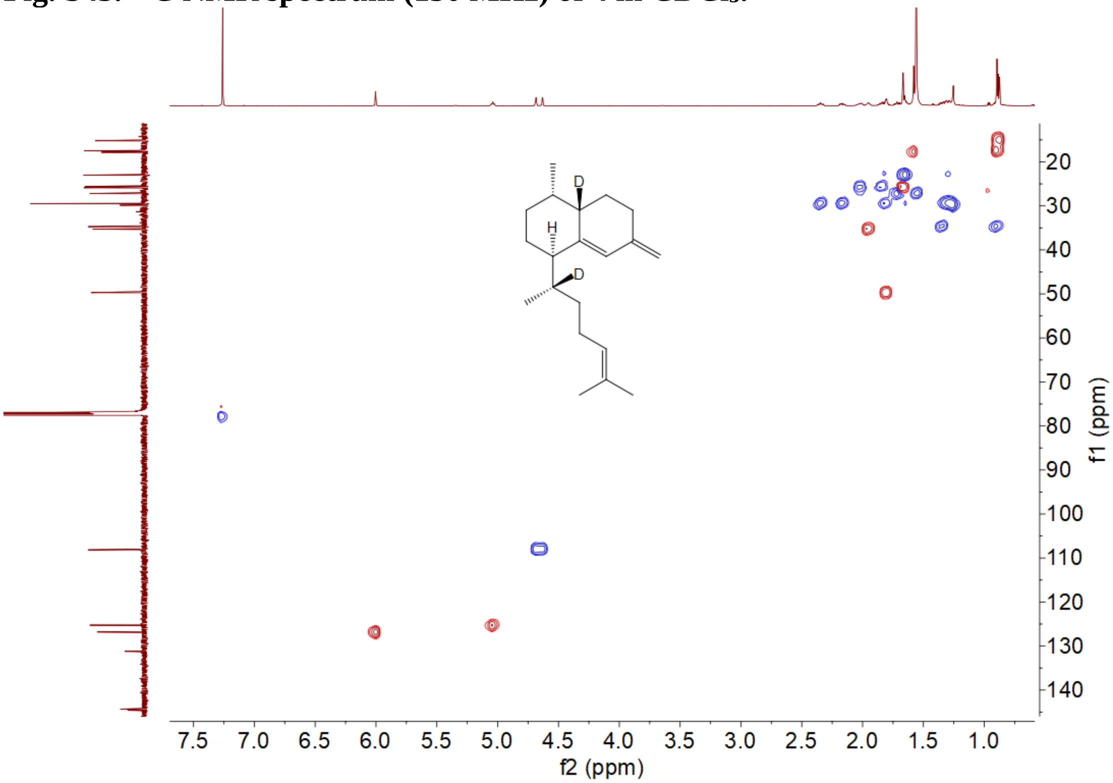

Fig. S44. HSQC NMR spectrum of 4 in CDCl$_3$.

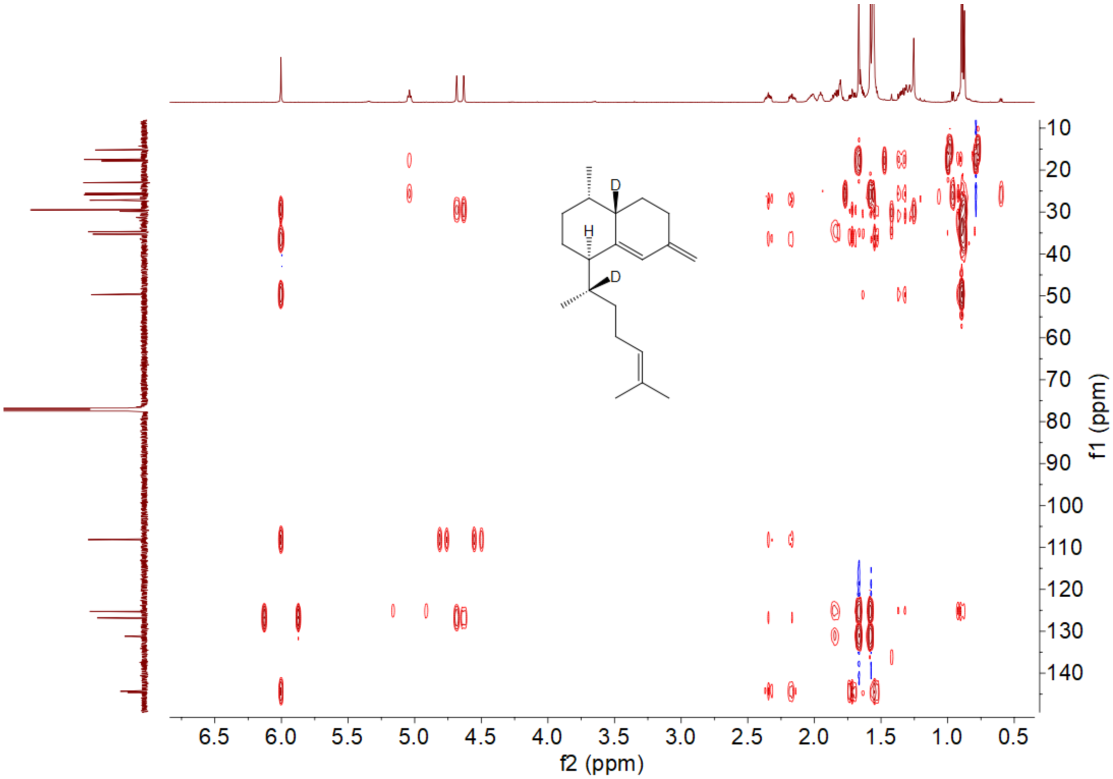

Fig. S45. HMBC NMR spectrum of 4 in $CDCl_3$.

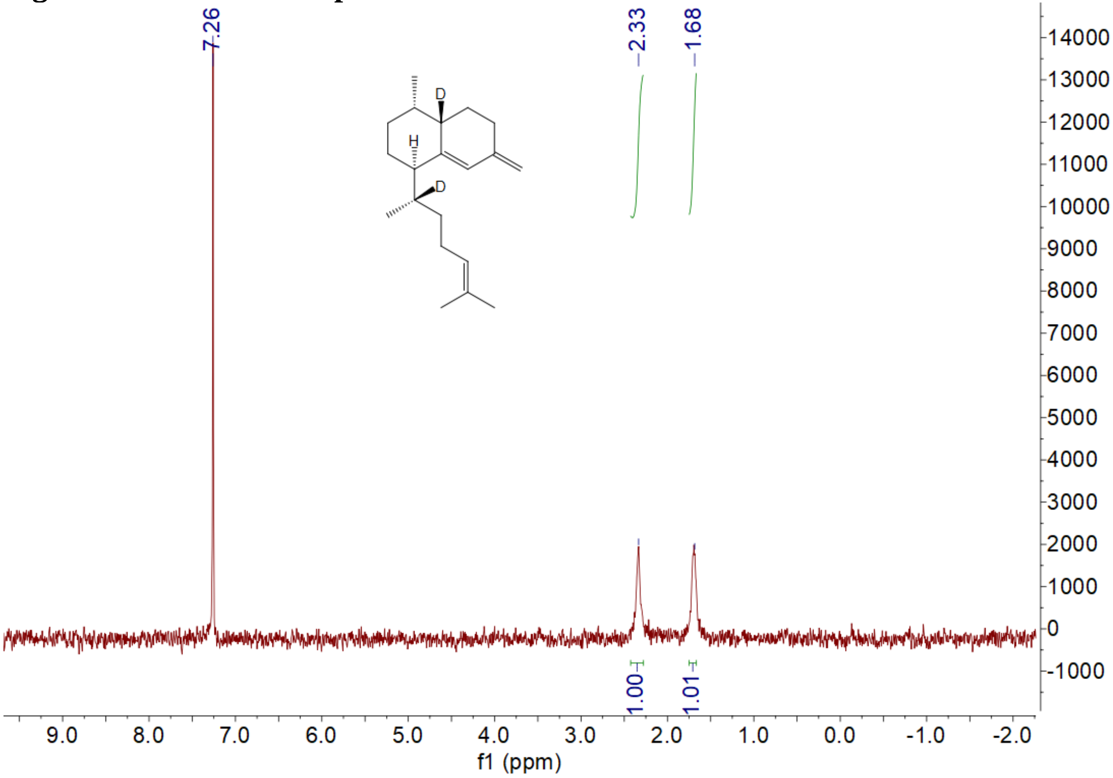

Fig. S46. Deuterium NMR spectrum of 4 in $CDCl_3$.

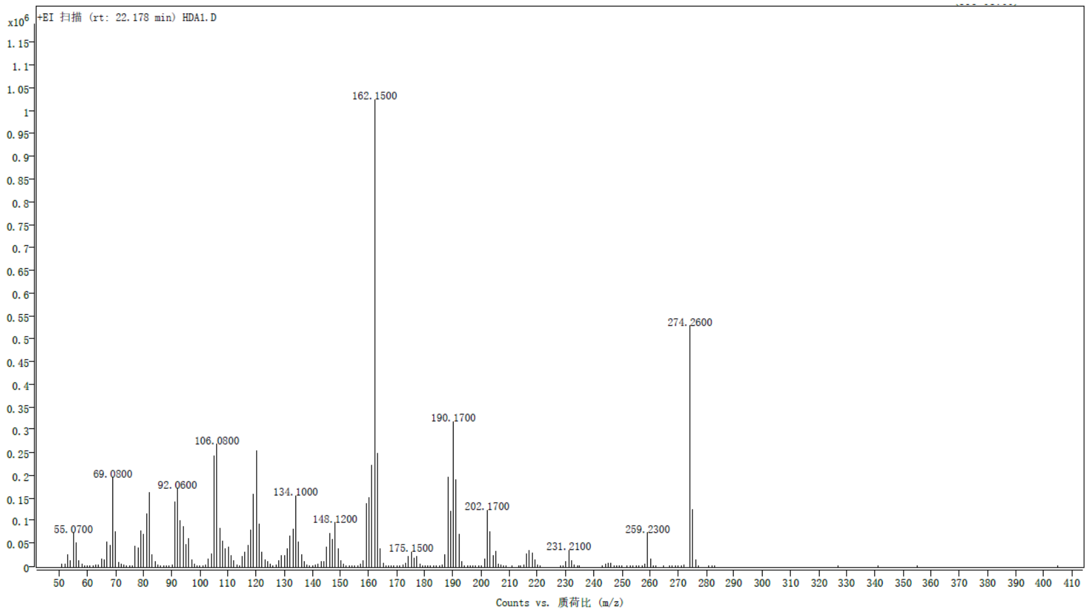

Fig. S47. The GC-MS spectra of 4.

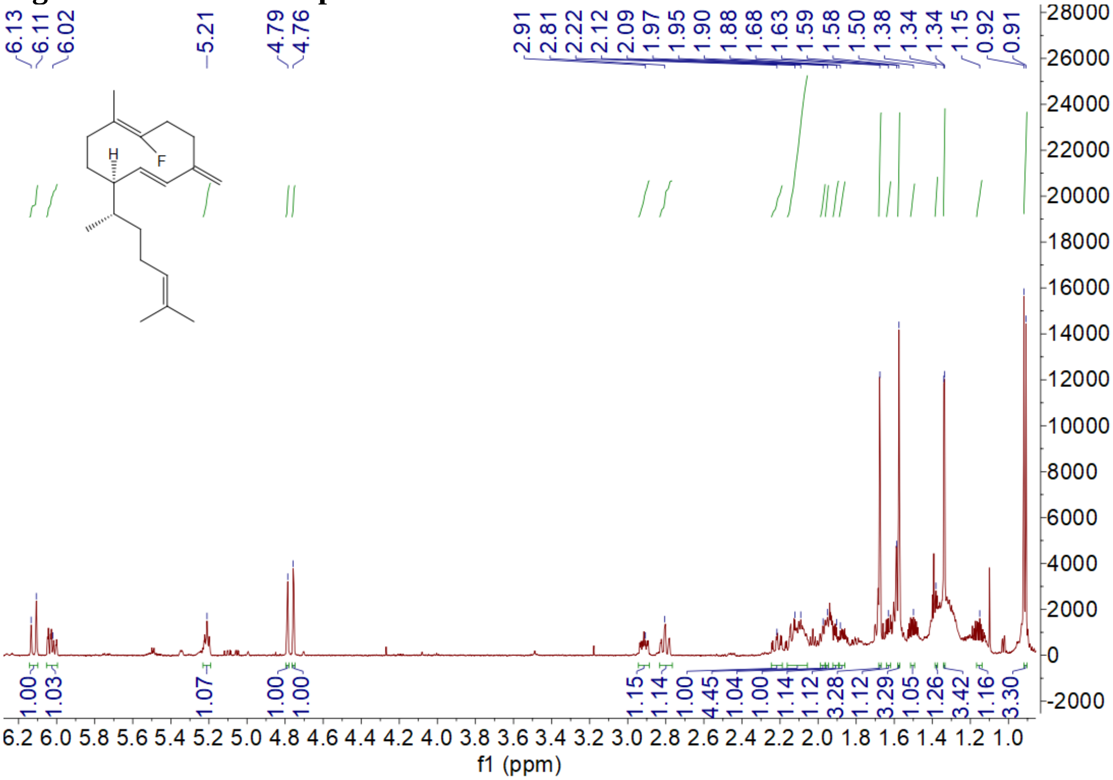

Fig. S48. ^{1}H NMR spectrum (600 MHz) of 5 in C_6D_6.

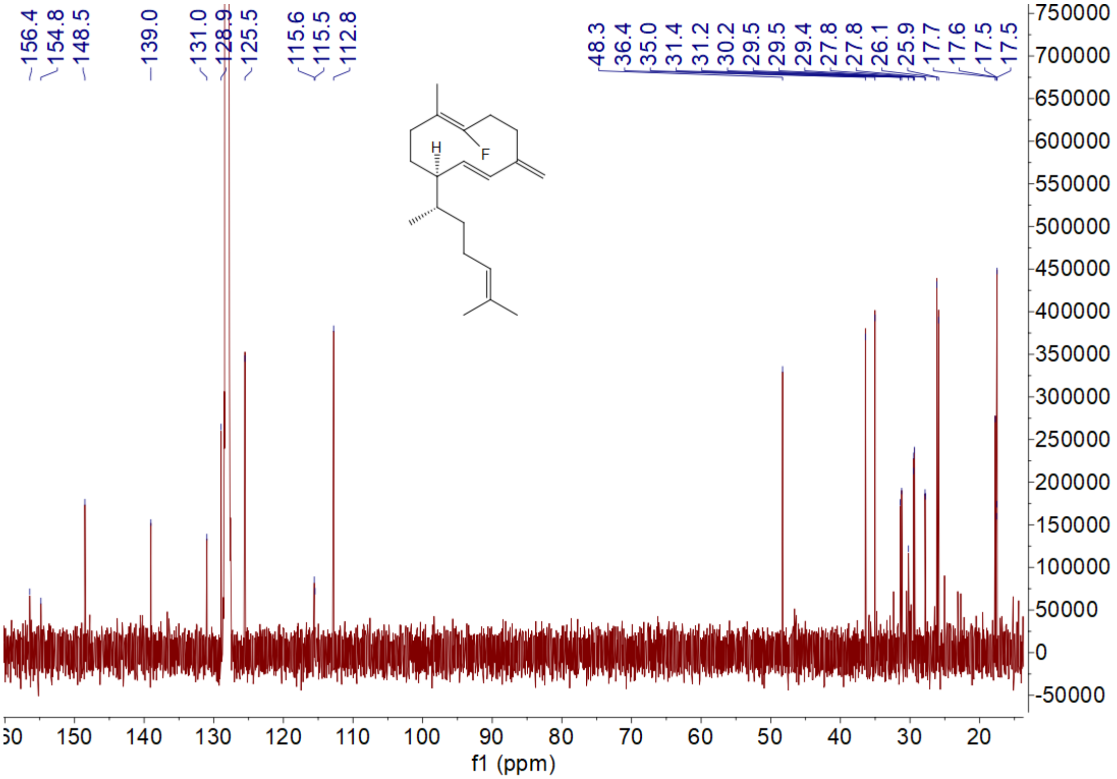

Fig. S49. ^{13}C NMR spectrum (150 MHz) of 5 in C_6D_6.

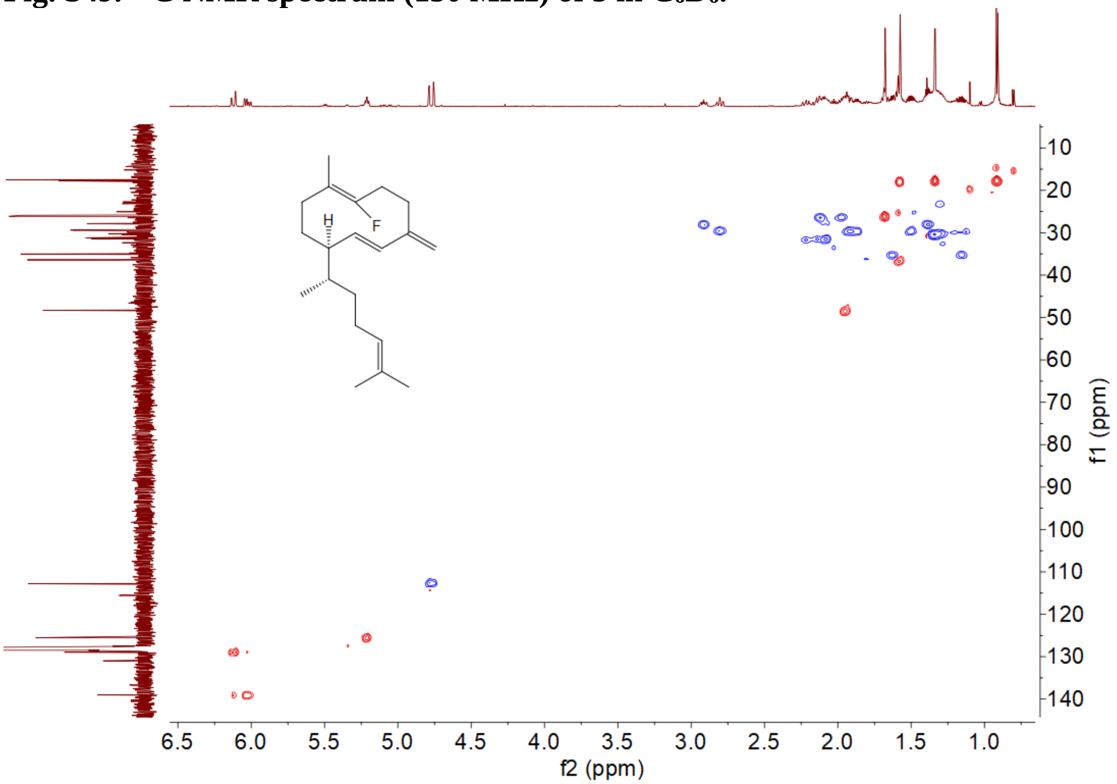

Fig. S50. HSQC NMR spectrum of 5 in C_6D_6.

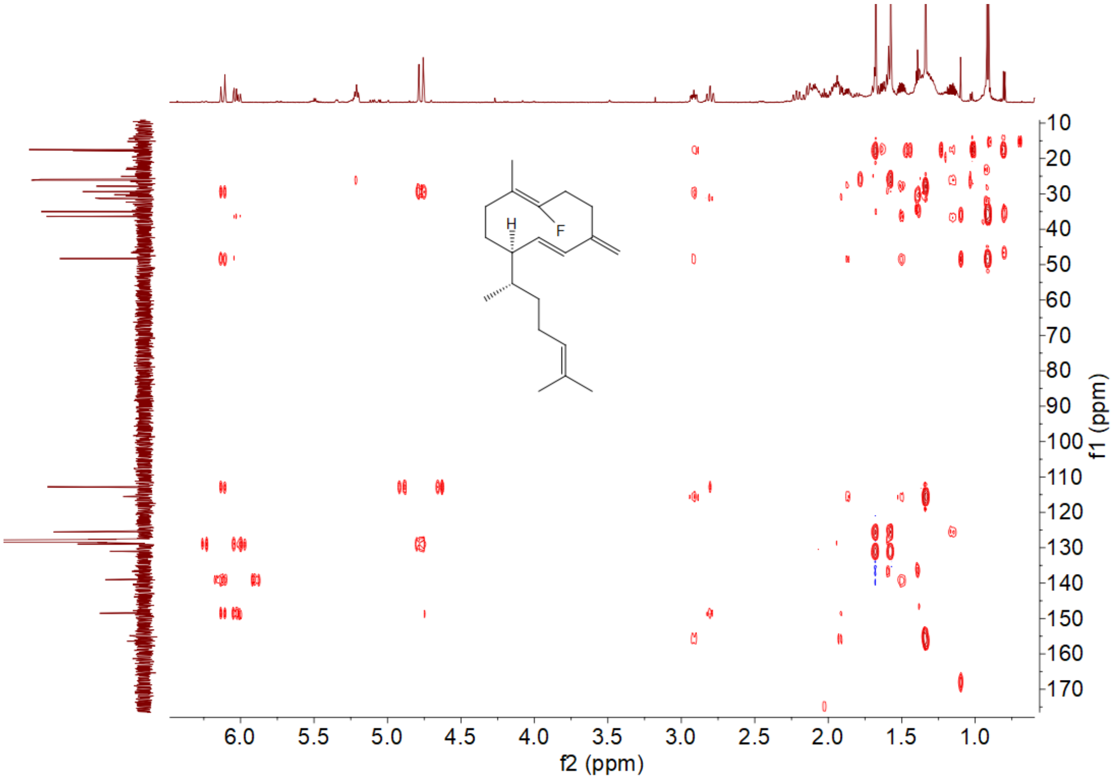

Fig. S51. HMBC NMR spectrum of 5 in C_6D_6.

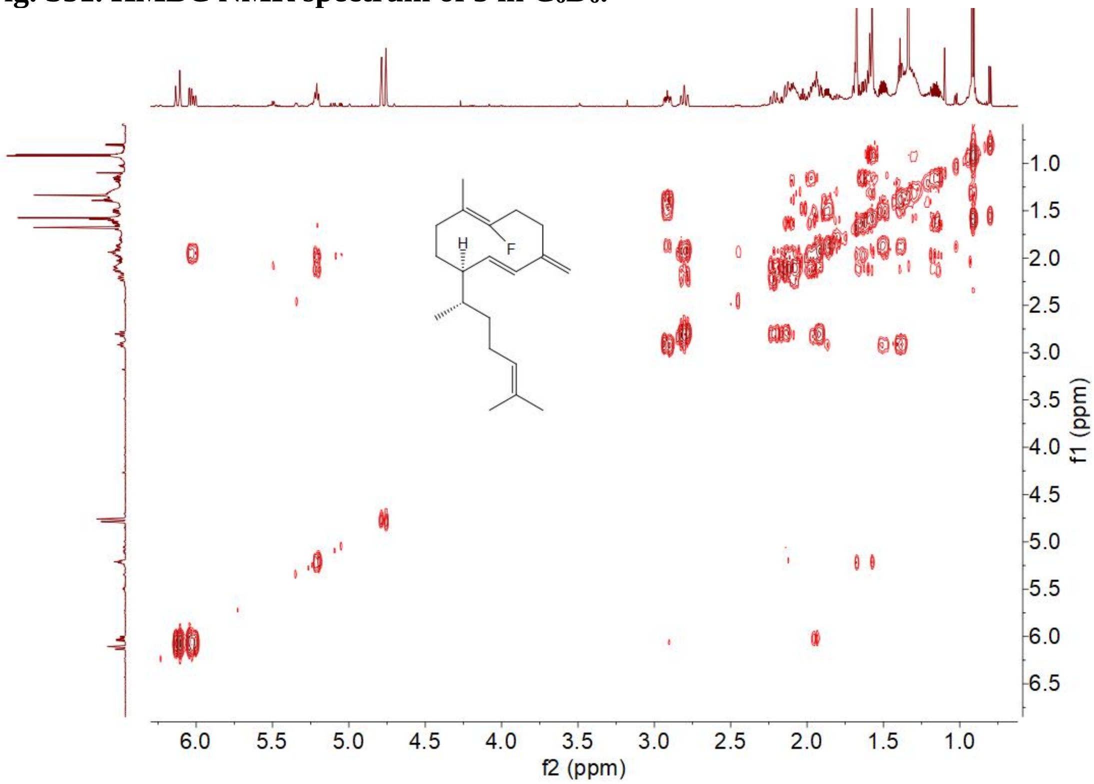

Fig. S52. ^1H-^1H COSY NMR spectrum of 5 in C_6D_6.

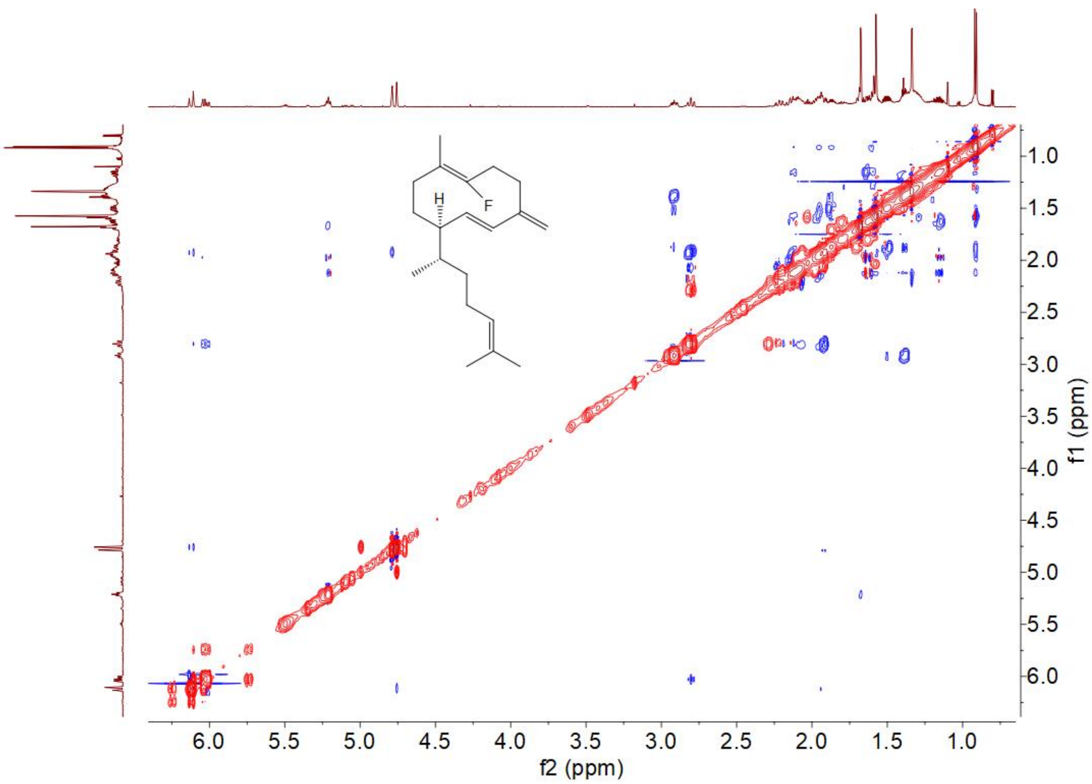

Fig. S53. NOESY NMR spectrum of 5 in C_6D_6.

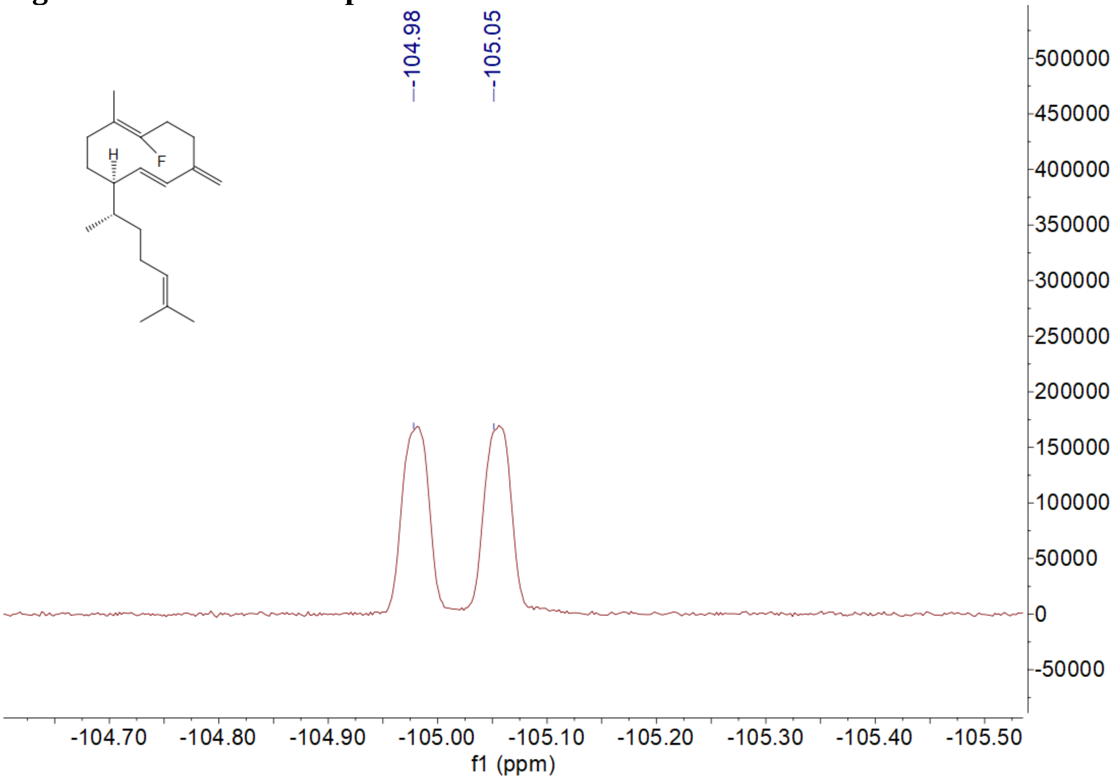

Fig. S54. ^{19}F NMR spectrum (600 MHz) of 5 in C_6D_6.

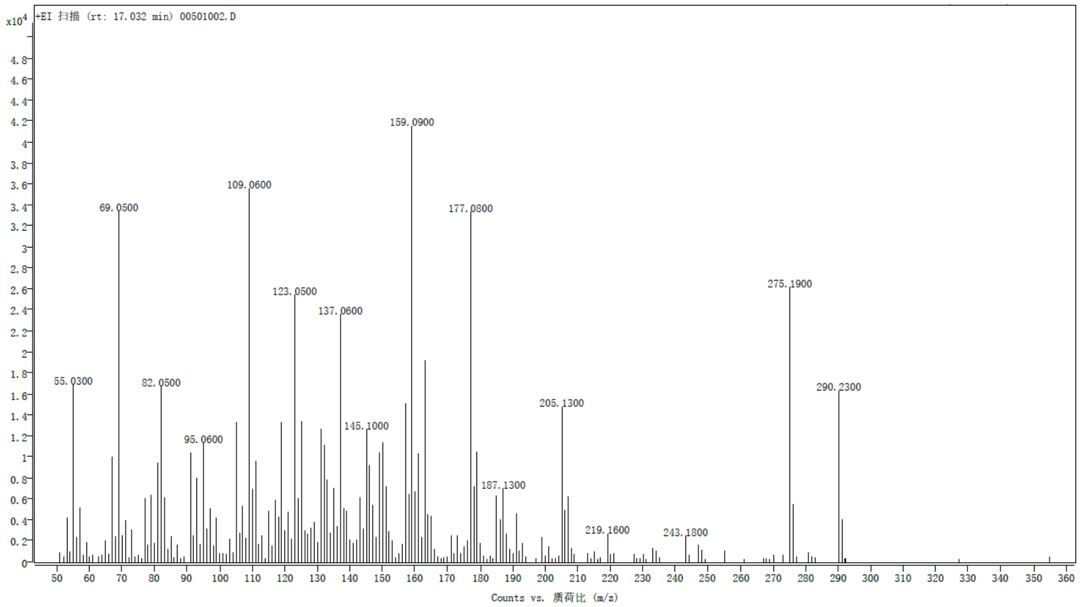

Fig. S55. The GC-MS spectra of 5.

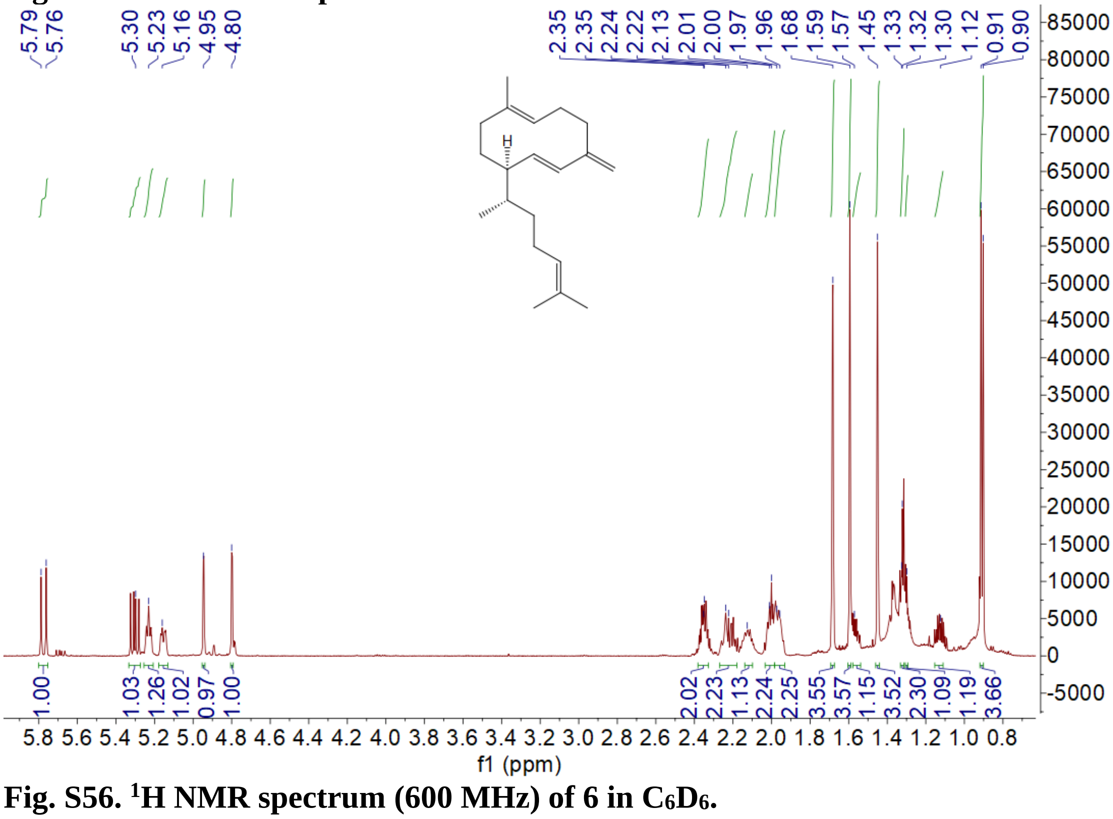

Fig. S56. [1]**H NMR spectrum (600 MHz) of 6 in C_6D_6.**

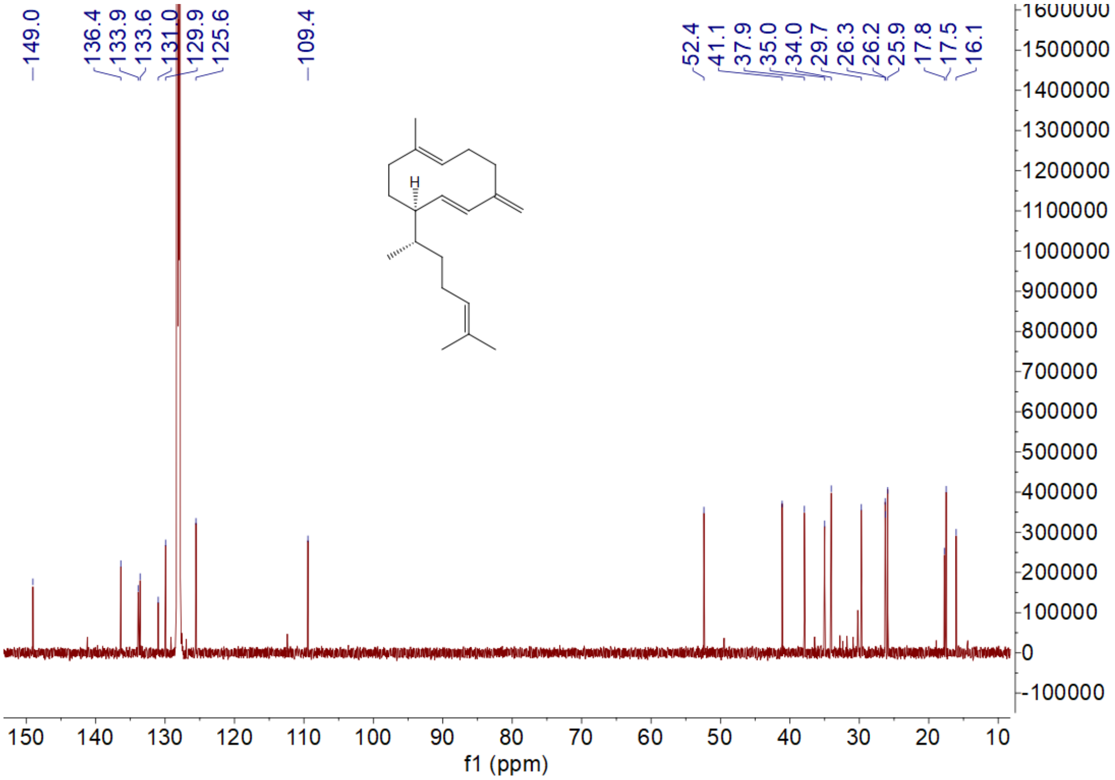

Fig. S57. ^{13}C NMR spectrum (150 MHz) of 6 in C_6D_6.

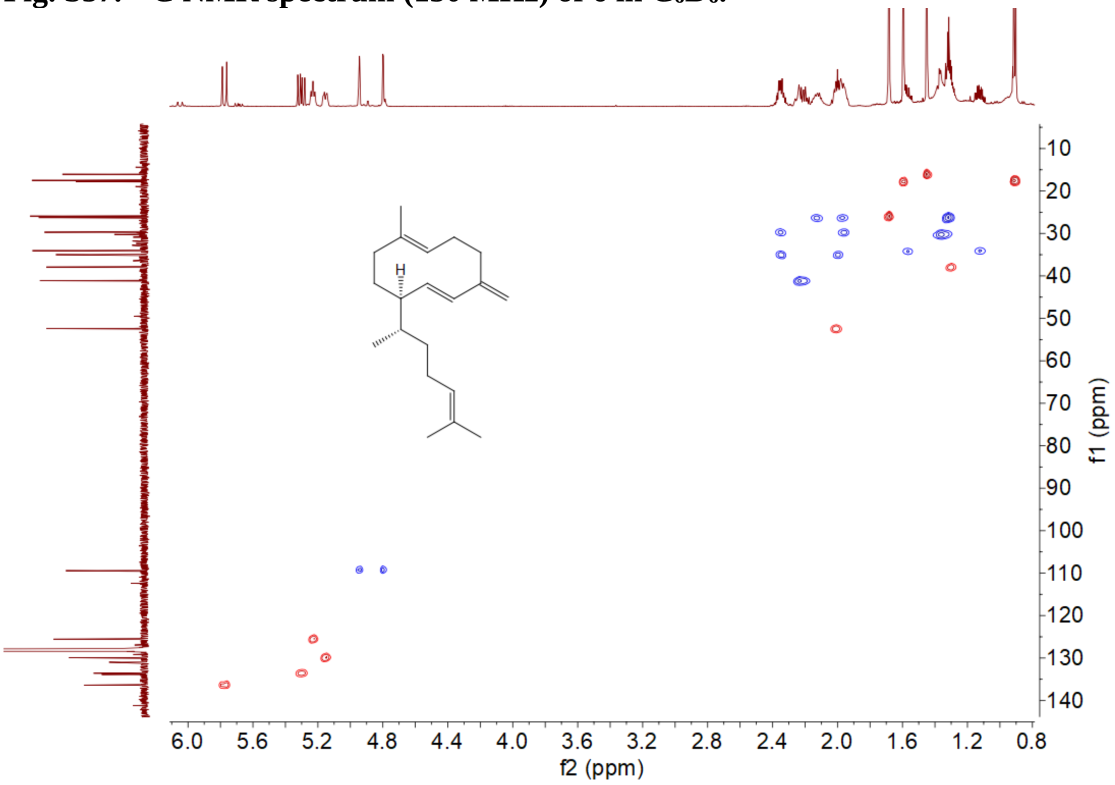

Fig. S58. HSQC NMR spectrum of 6 in C_6D_6.

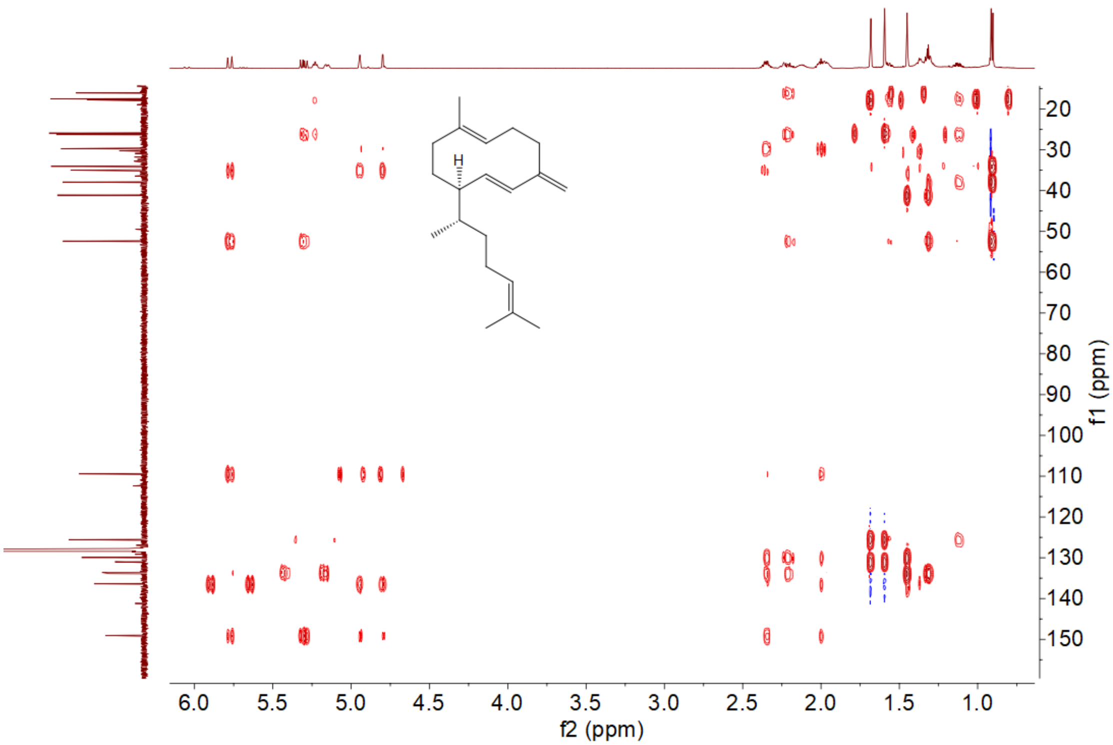

Fig. S59. HMBC NMR spectrum of 6 in C_6D_6.

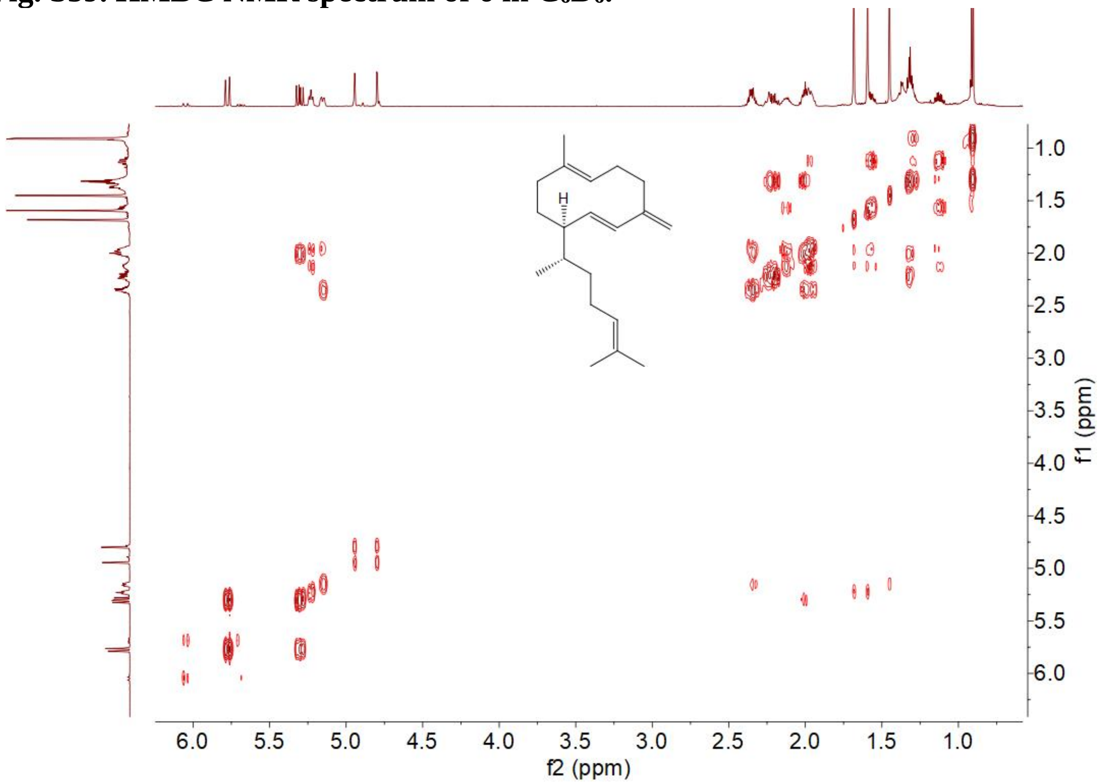

Fig. S60. ^1H-^1H COSY NMR spectrum of 6 in C_6D_6.

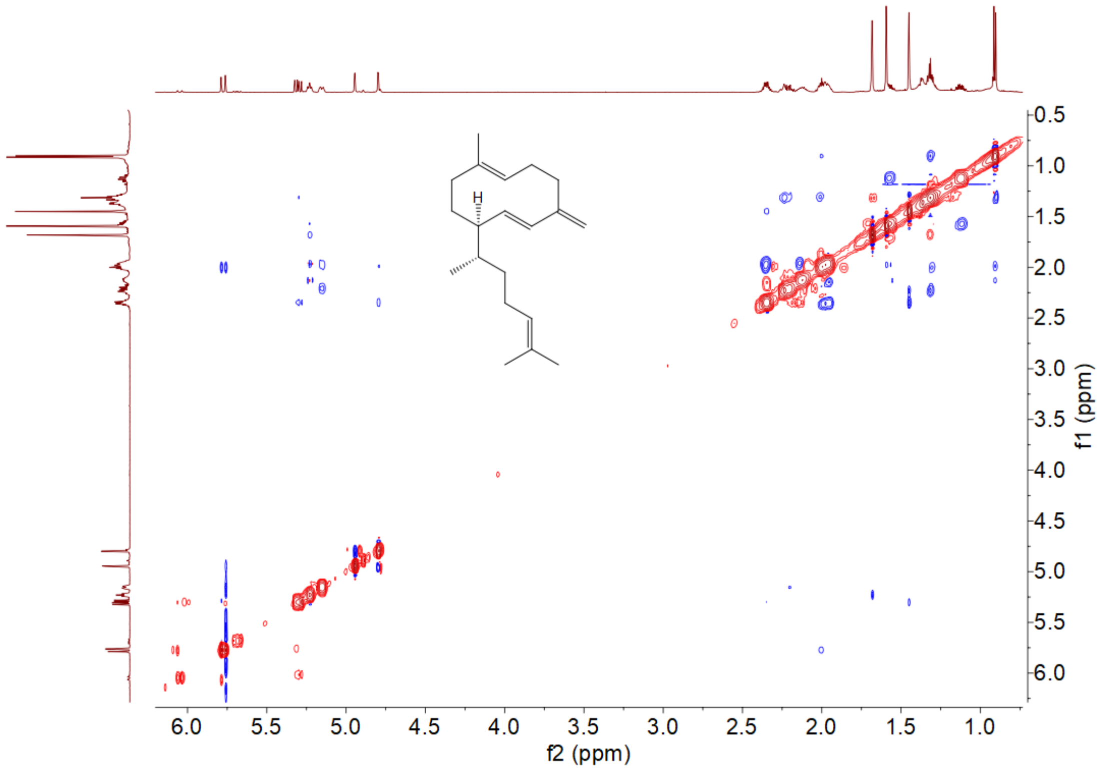

Fig. S61. NOESY NMR spectrum of 6 in C_6D_6.

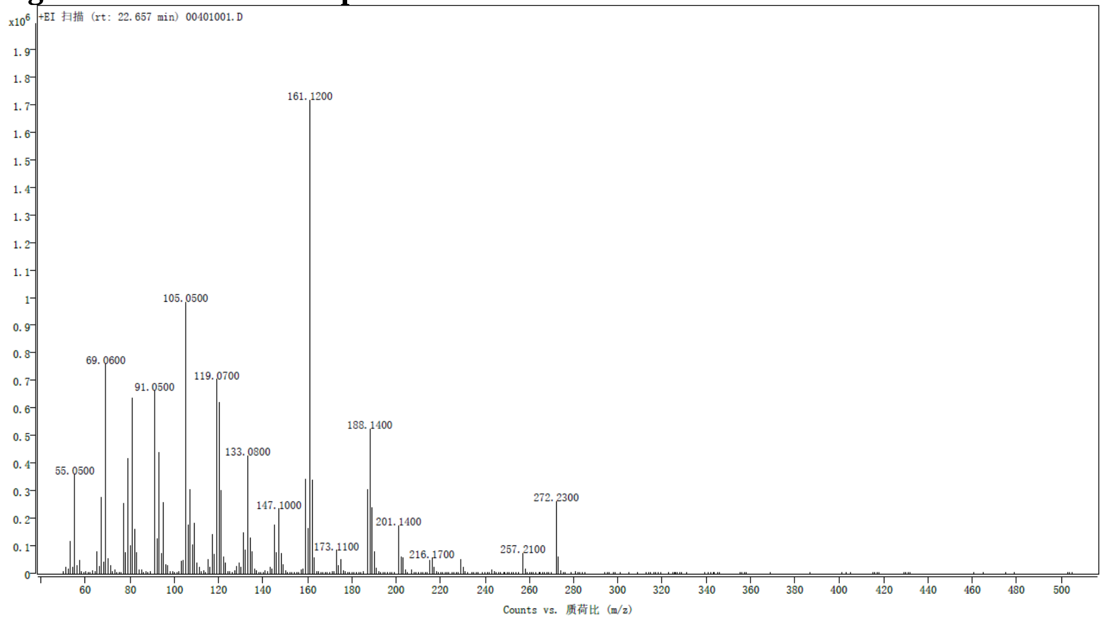

Fig. S62. The GC-MS spectra of 6.

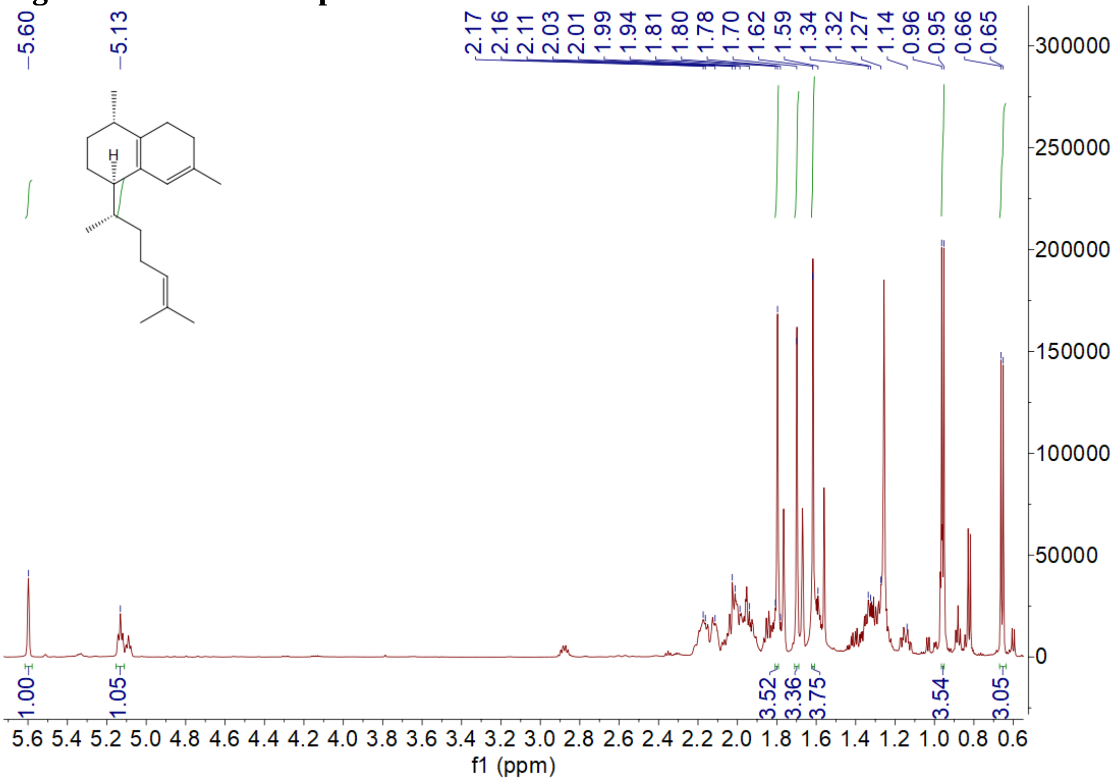

Fig. S63. ^{1}H NMR spectrum (600 MHz) of 7 in $CDCl_3$.

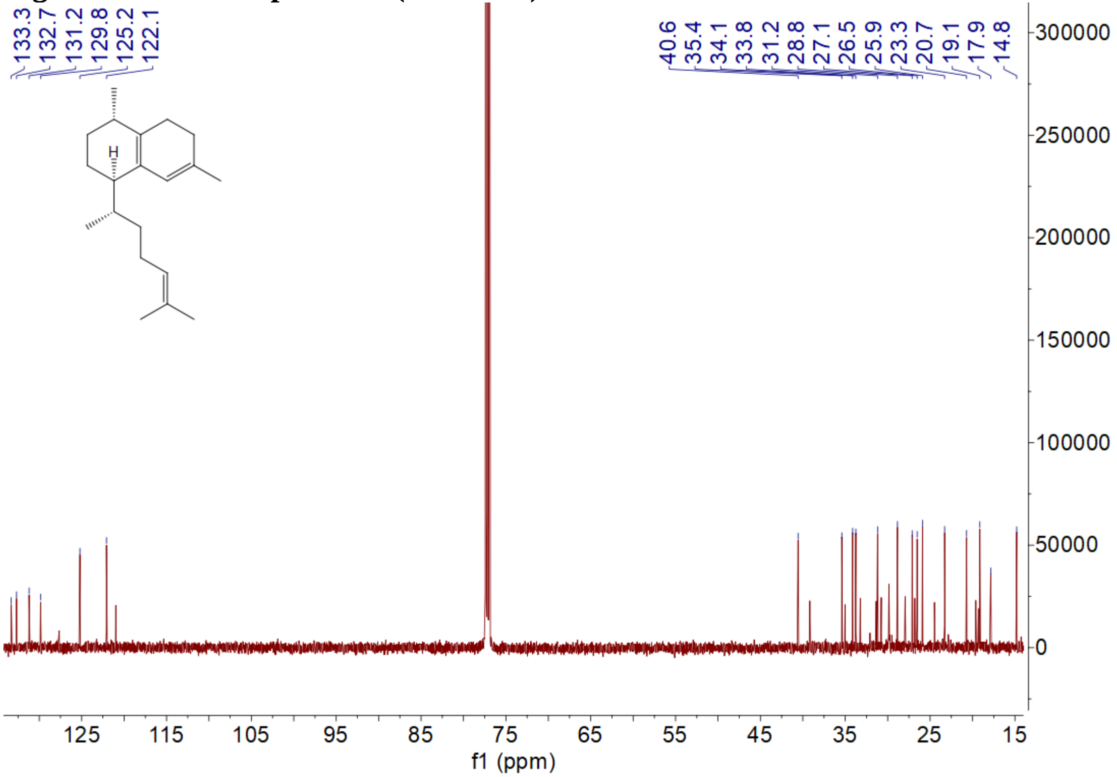

Fig. S64. ^{13}C NMR spectrum (150 MHz) of 7 in $CDCl_3$.

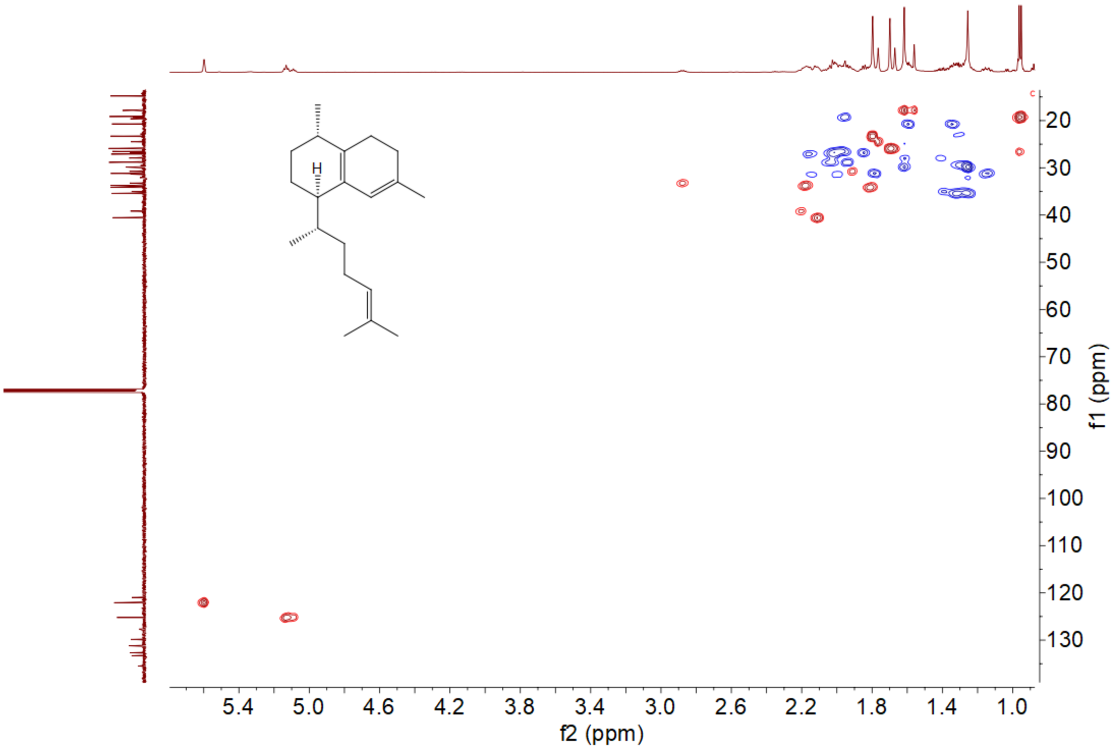

Fig. S65. HSQC NMR spectrum of 7 in $CDCl_3$.

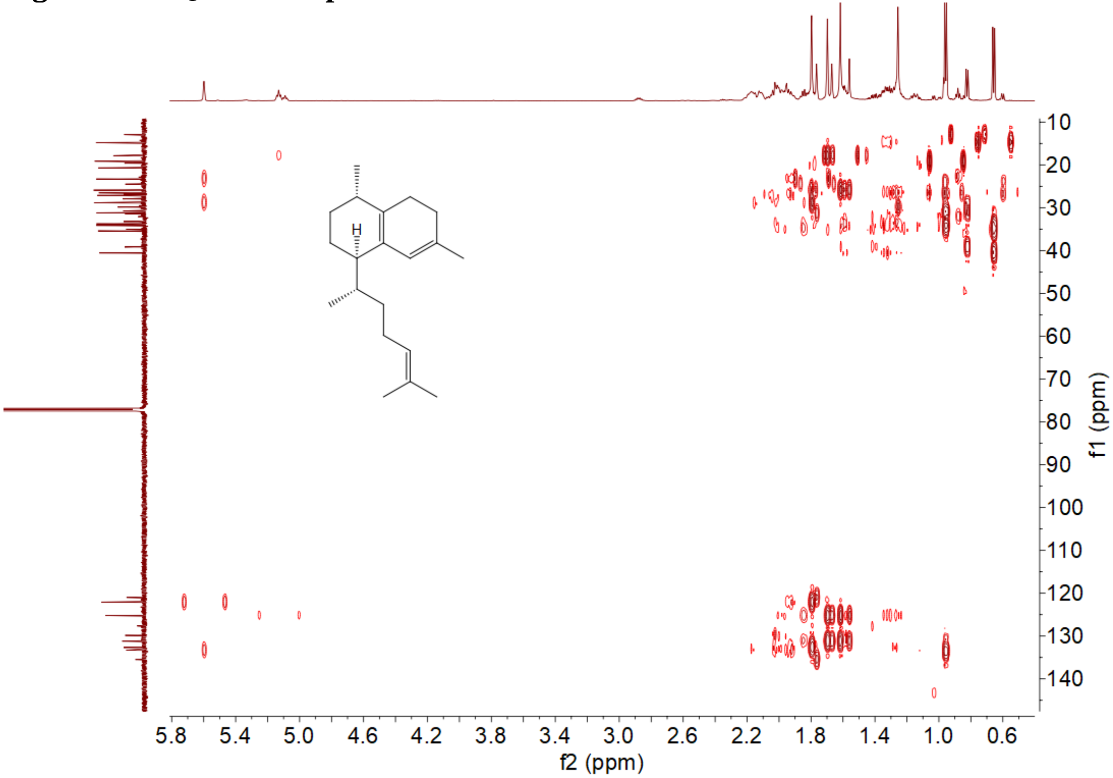

Fig. S66. HMBC NMR spectrum of 7 in $CDCl_3$.

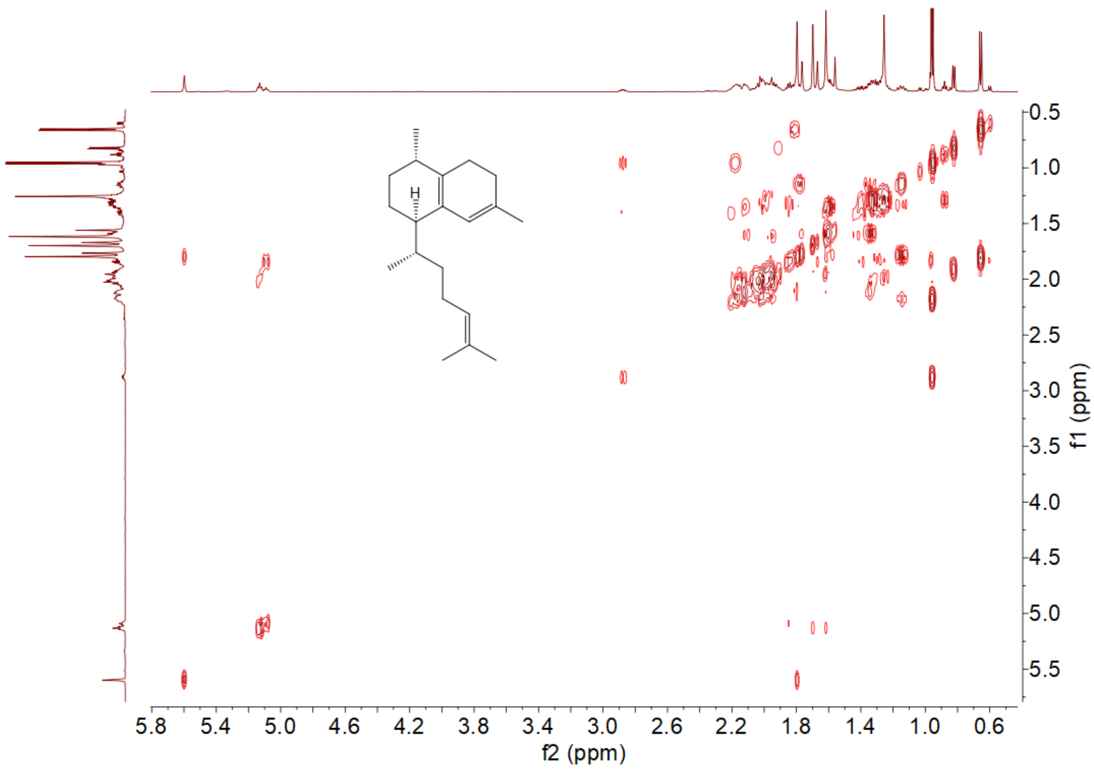

Fig. S67. ^{1}H-^{1}H COSY NMR spectrum of 7 in $CDCl_3$.

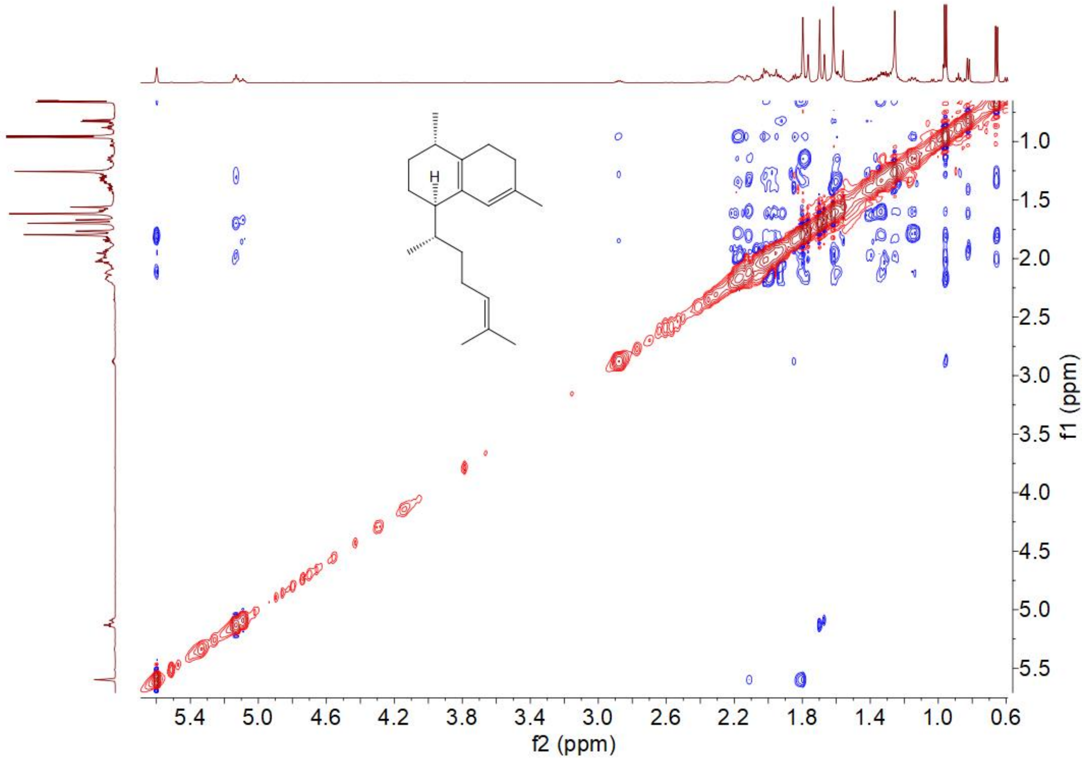

Fig. S68. NOESY NMR spectrum of 7 in $CDCl_3$.

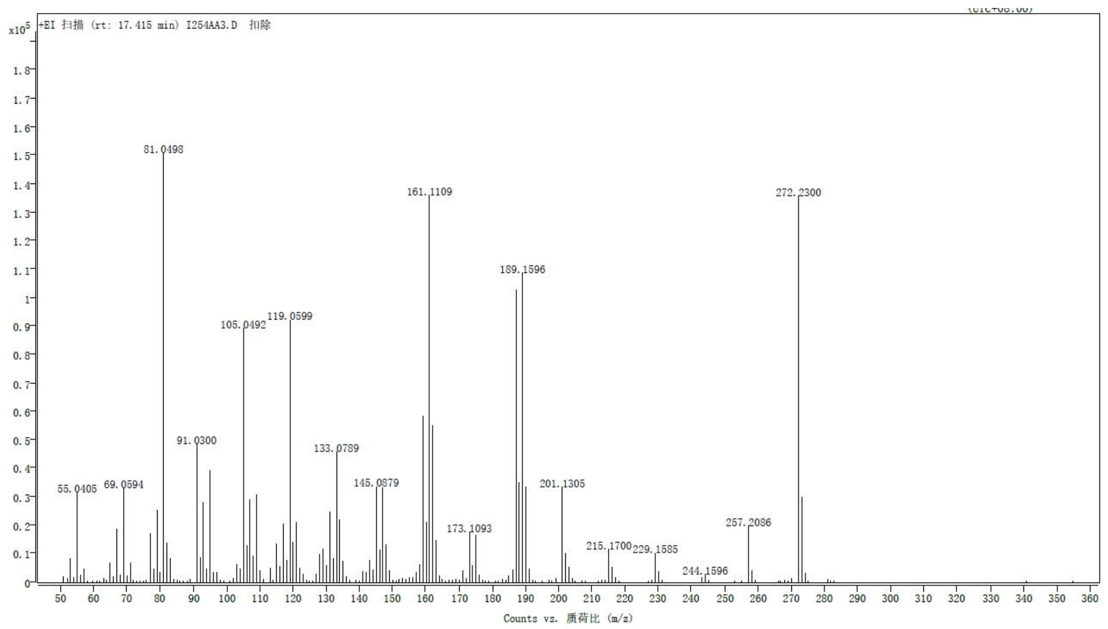

Fig. S69. The GC-MS spectra of 7.

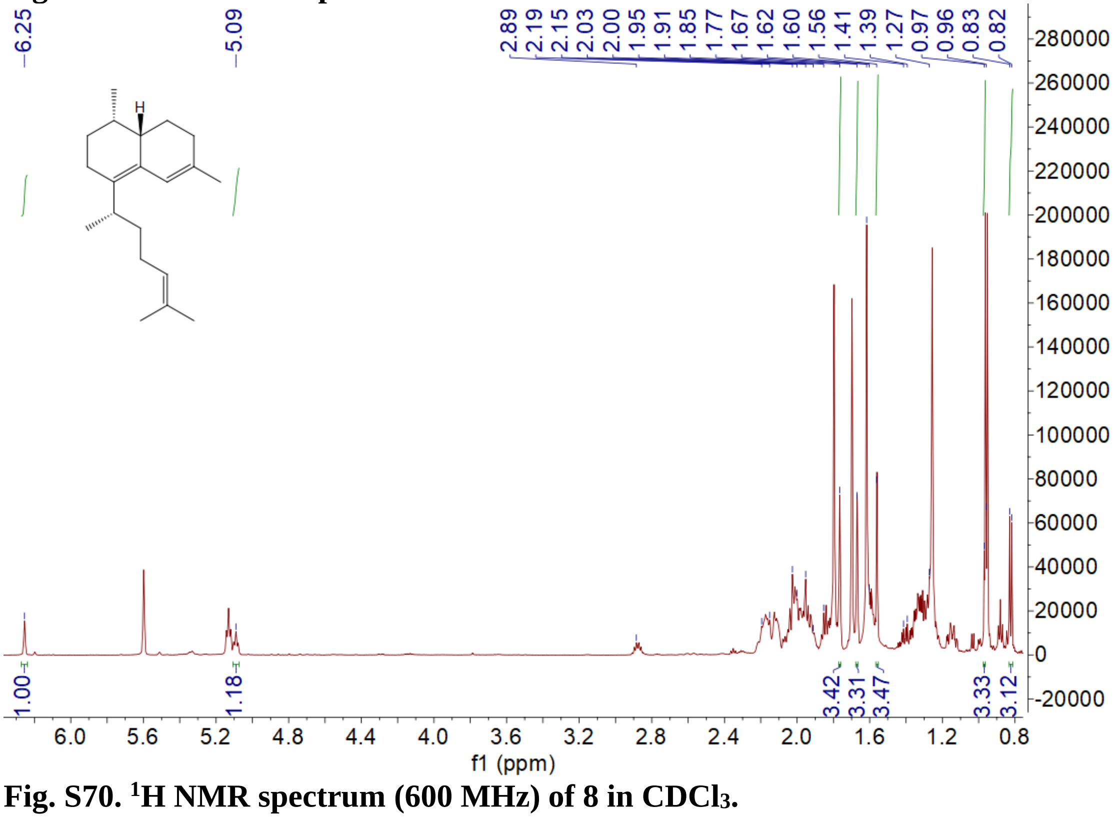

Fig. S70. ^{1}H NMR spectrum (600 MHz) of 8 in $CDCl_3$.

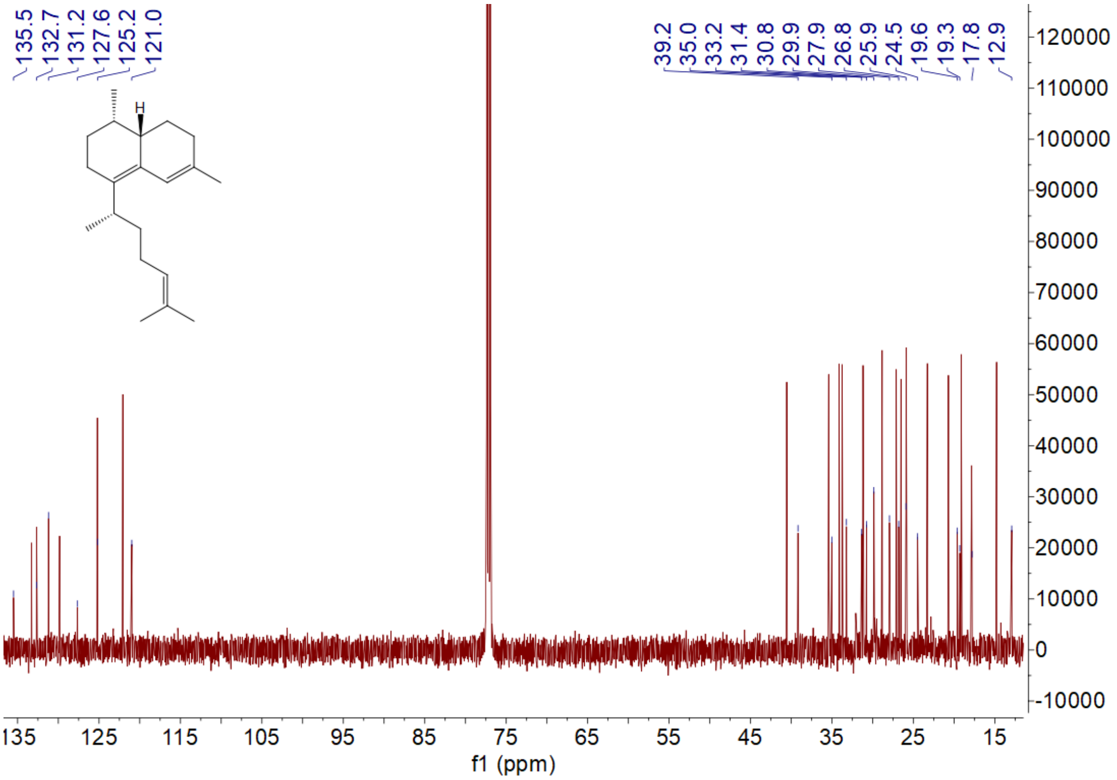

Fig. S71. ^{13}**C NMR spectrum (150 MHz) of 8 in** $CDCl_3$**.**

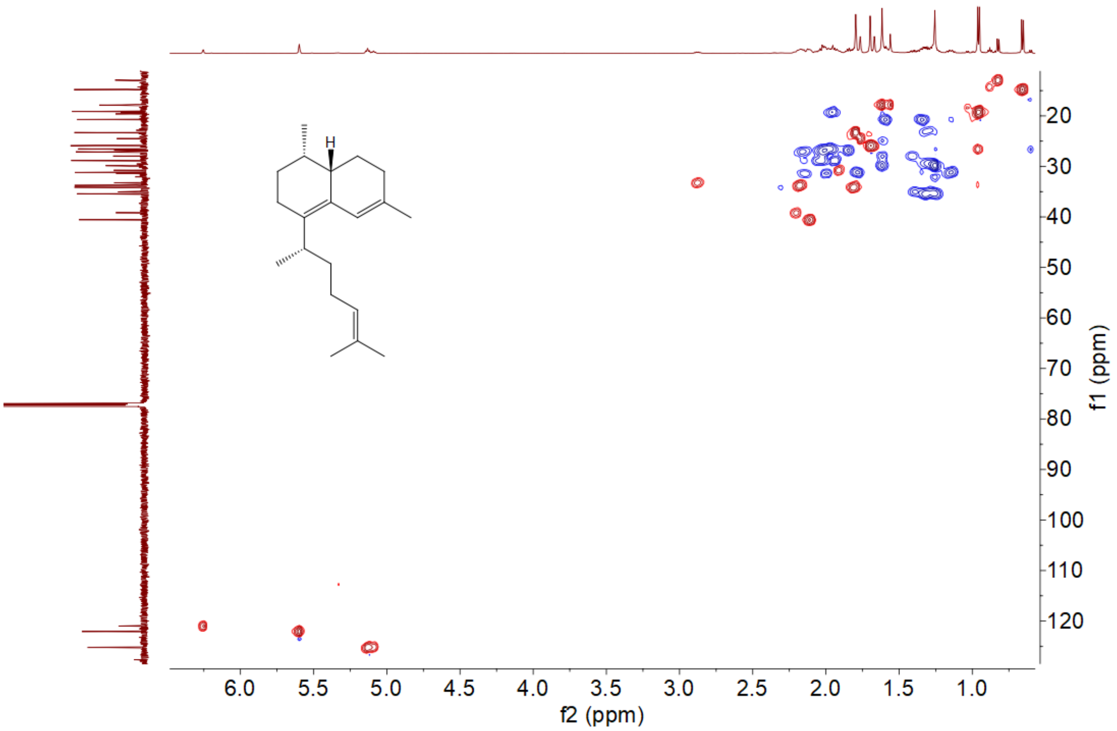

Fig. S72. HSQC NMR spectrum of 8 in $CDCl_3$**.**

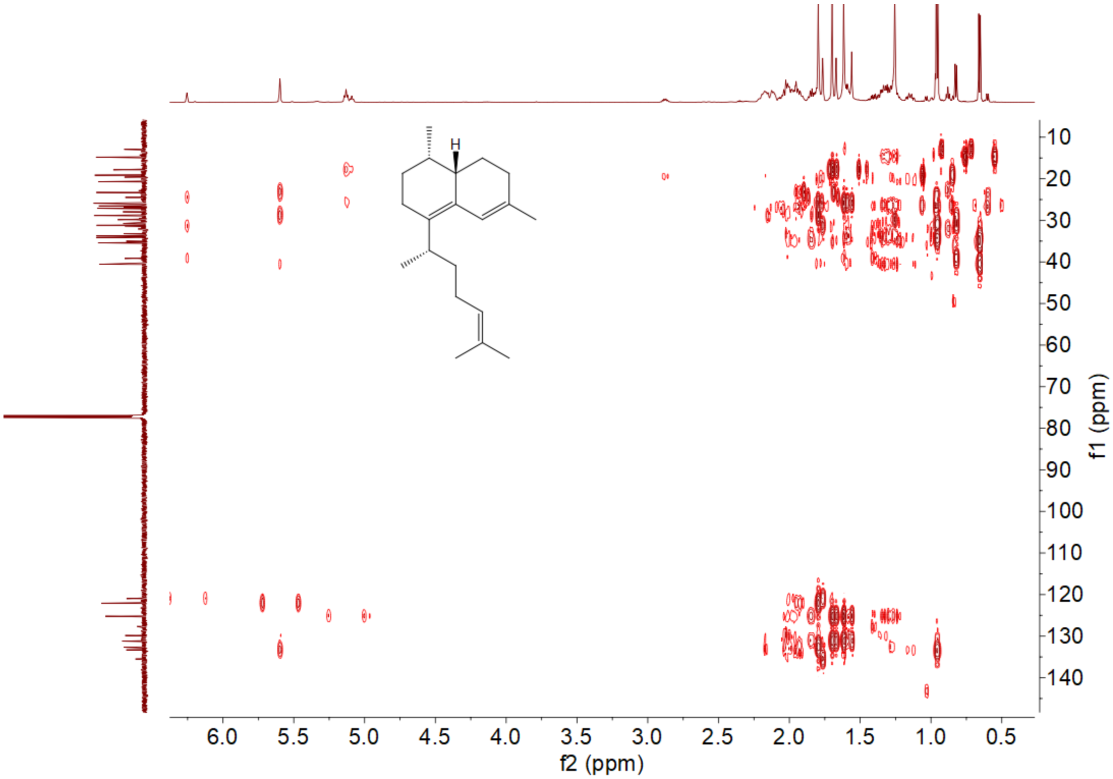

Fig. S73. HMBC NMR spectrum of 8 in $CDCl_3$.

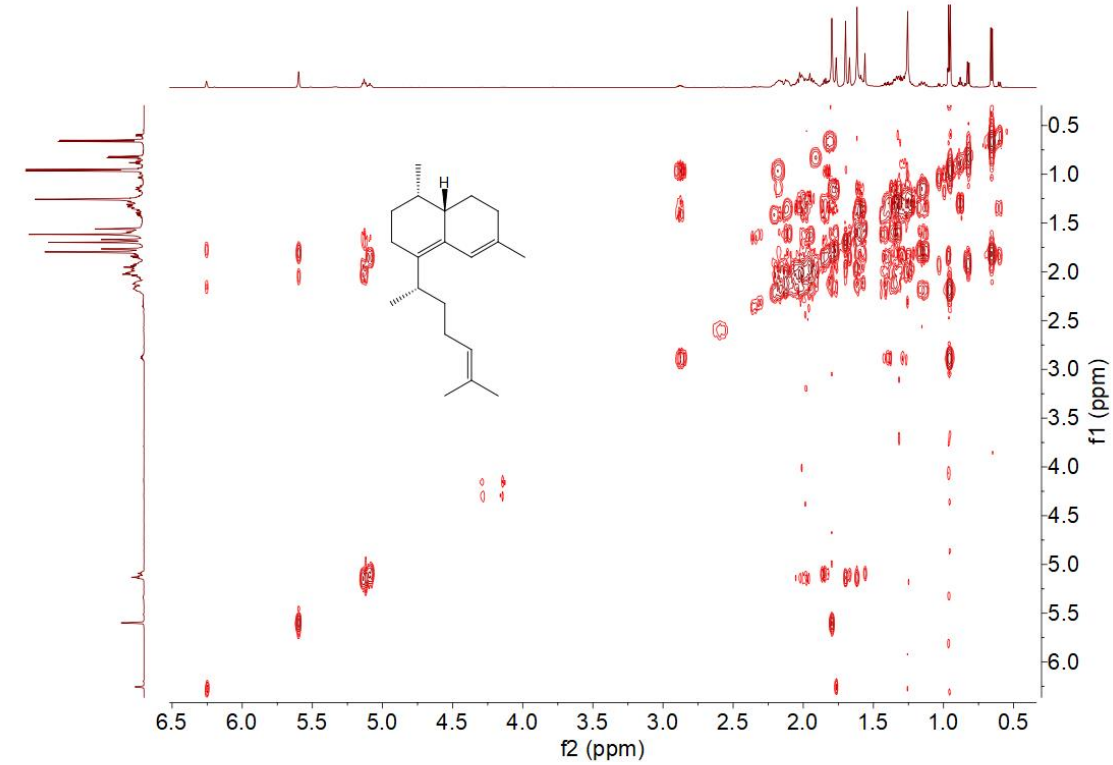

Fig. S74. ^1H-^1H COSY NMR spectrum of 8 in $CDCl_3$.

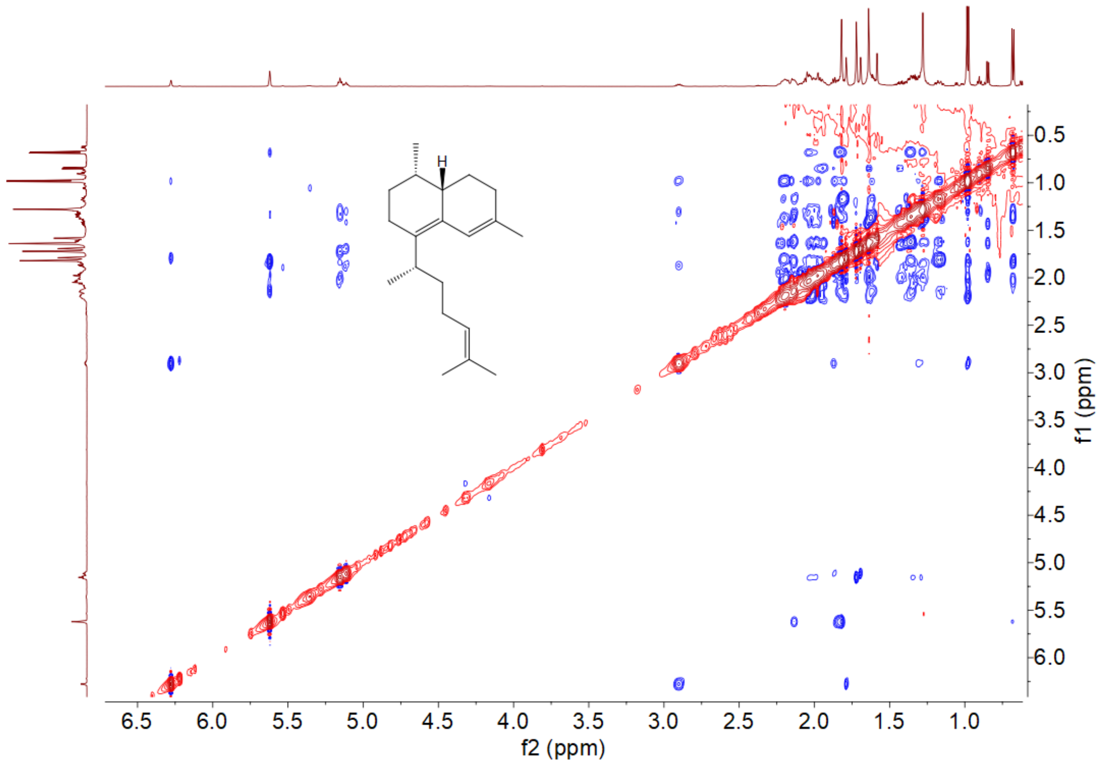

Fig. S75. NOESY NMR spectrum of 8 in CDCl$_3$.

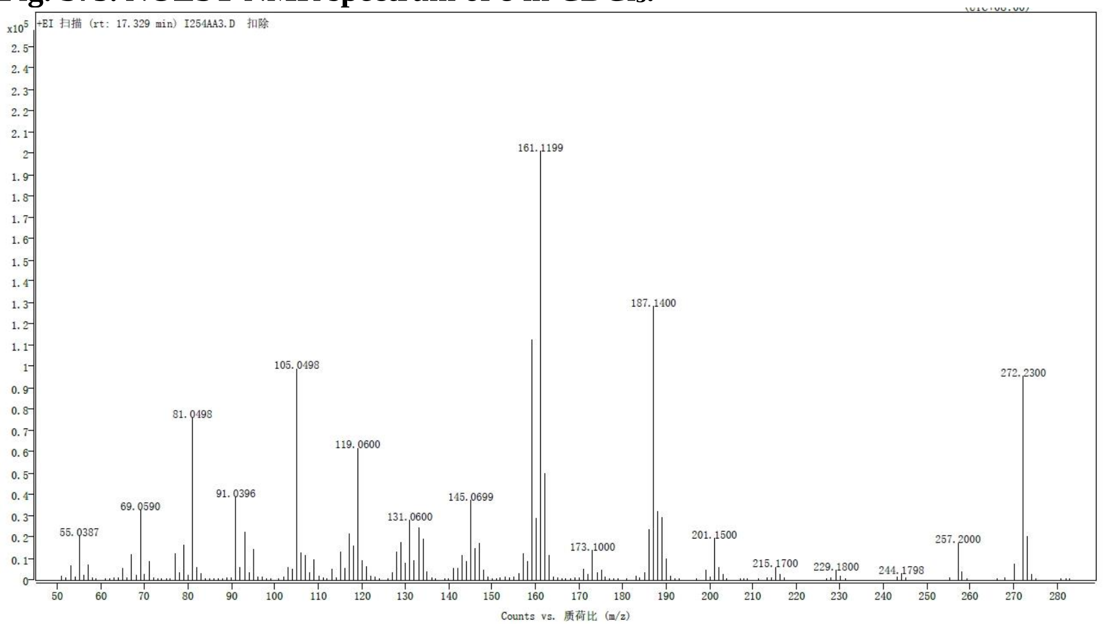

Fig. S76. The GC-MS spectra of 8.

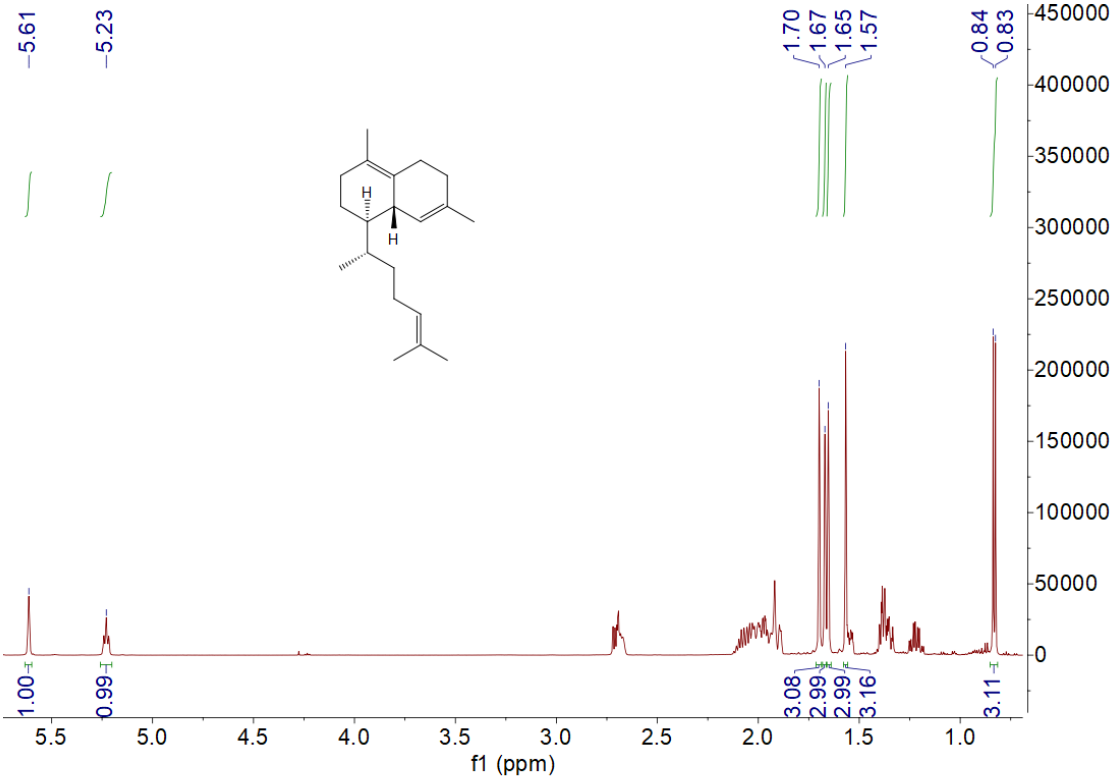

Fig. S77. ^{1}H **NMR spectrum (600 MHz) of 9 in** C_6D_6**.**

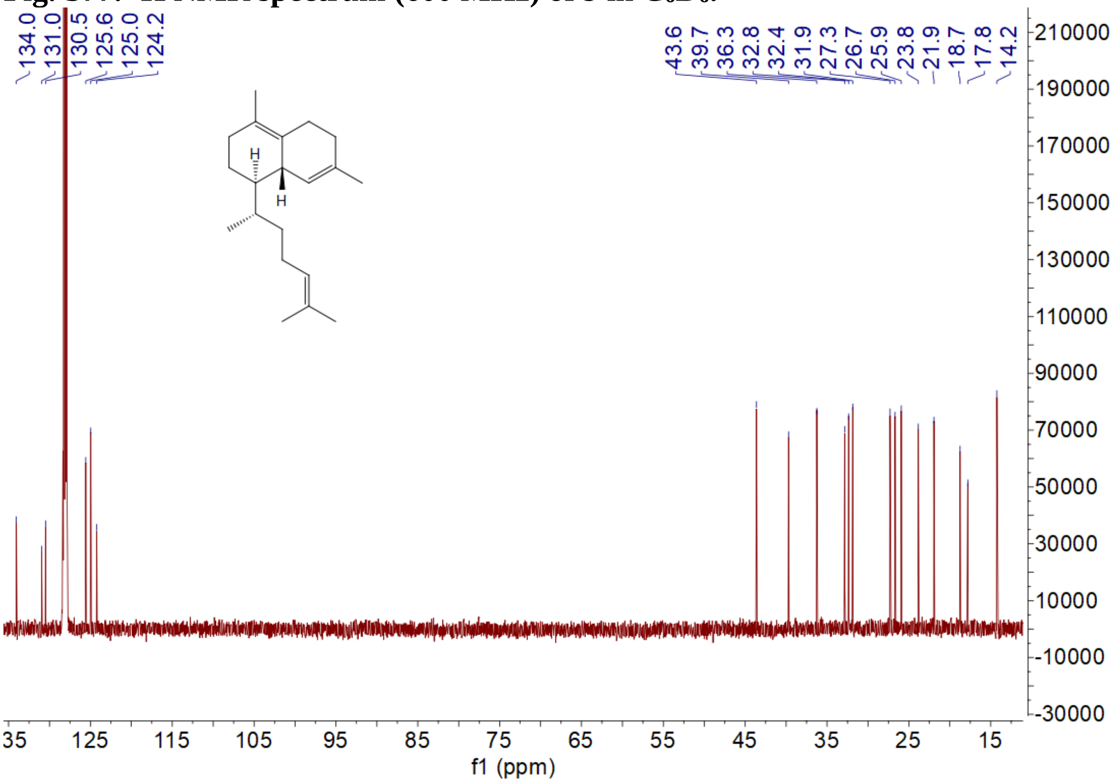

Fig. S78. ^{13}C **NMR spectrum (150 MHz) of 9 in** C_6D_6**.**

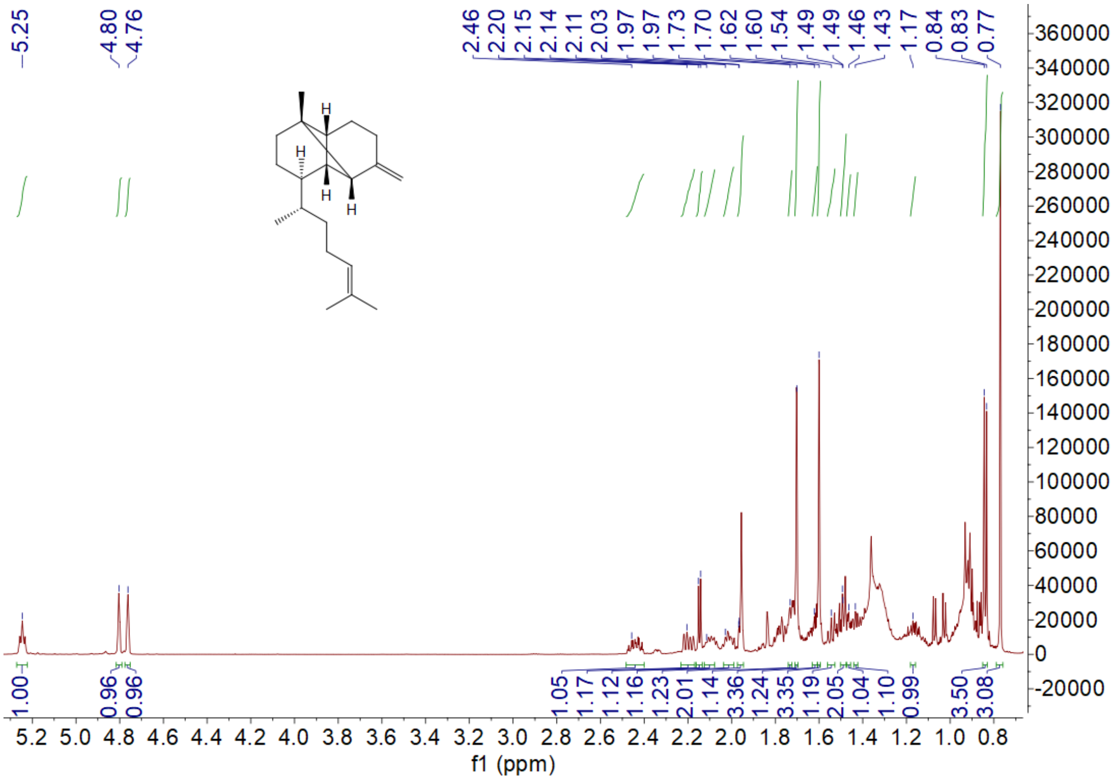

Fig. S79. ^{1}**H NMR spectrum (600 MHz) of 10 in** C_6D_6**.**

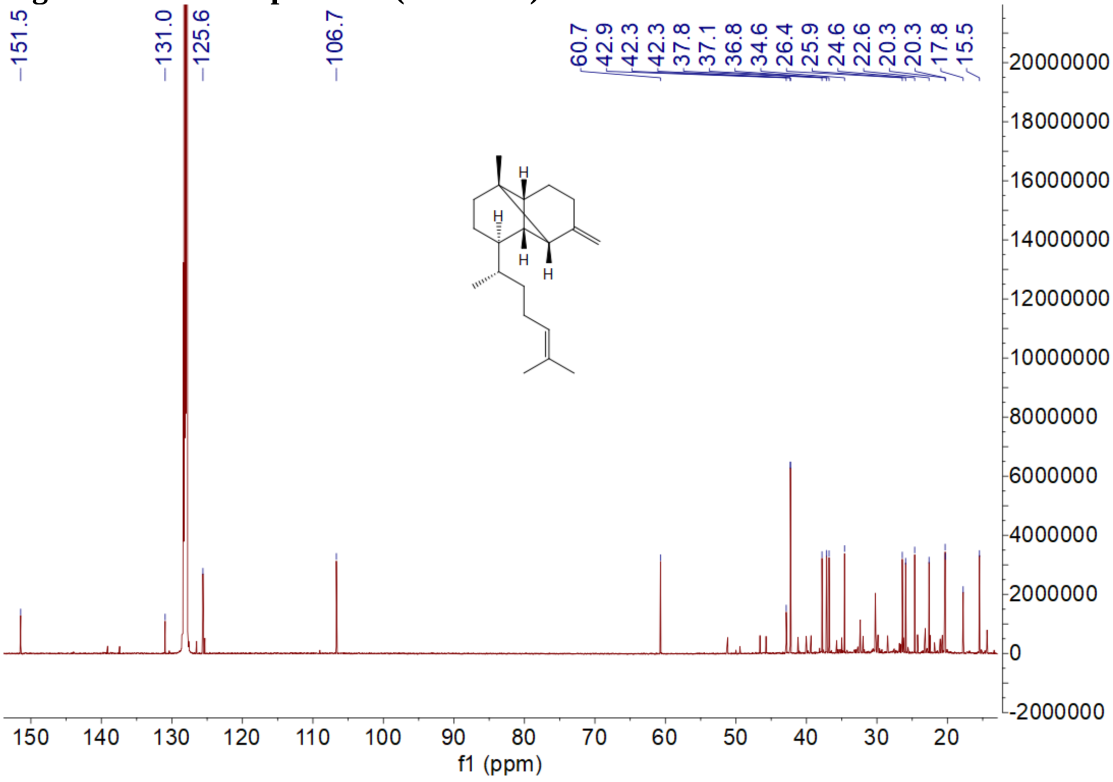

Fig. S80. ^{13}**C NMR spectrum (150 MHz) of 10 in** C_6D_6**.**

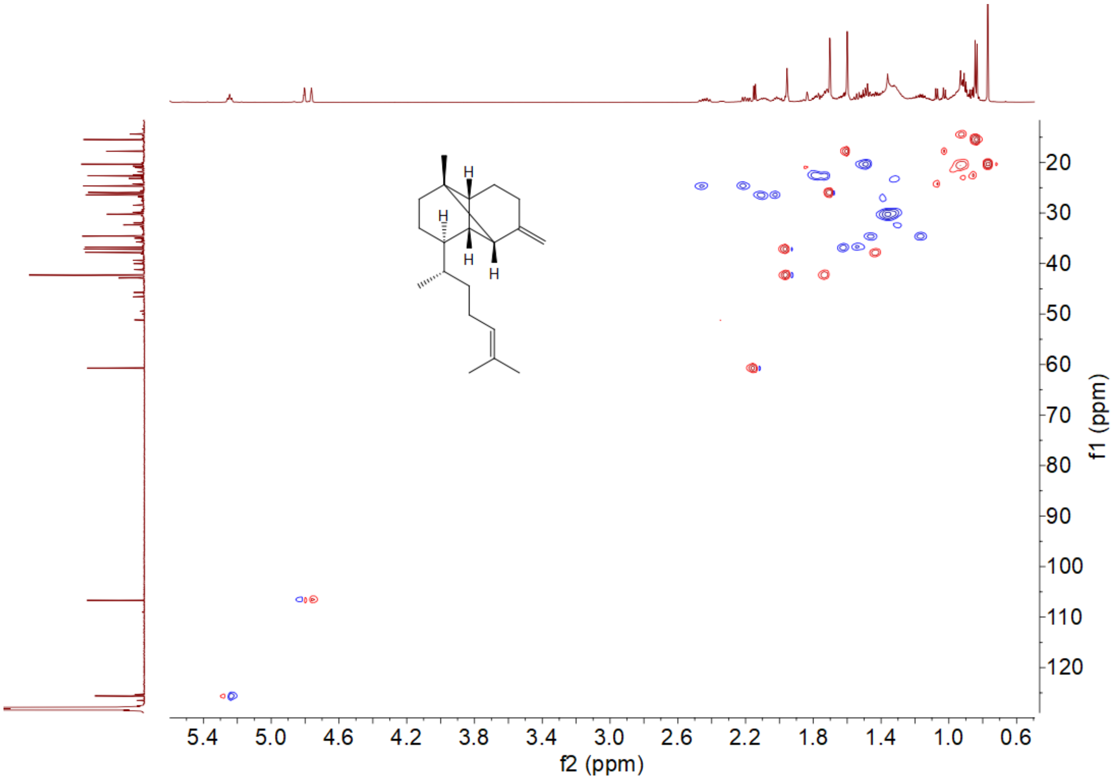

Fig. S81. HSQC NMR spectrum of 10 in C_6D_6.

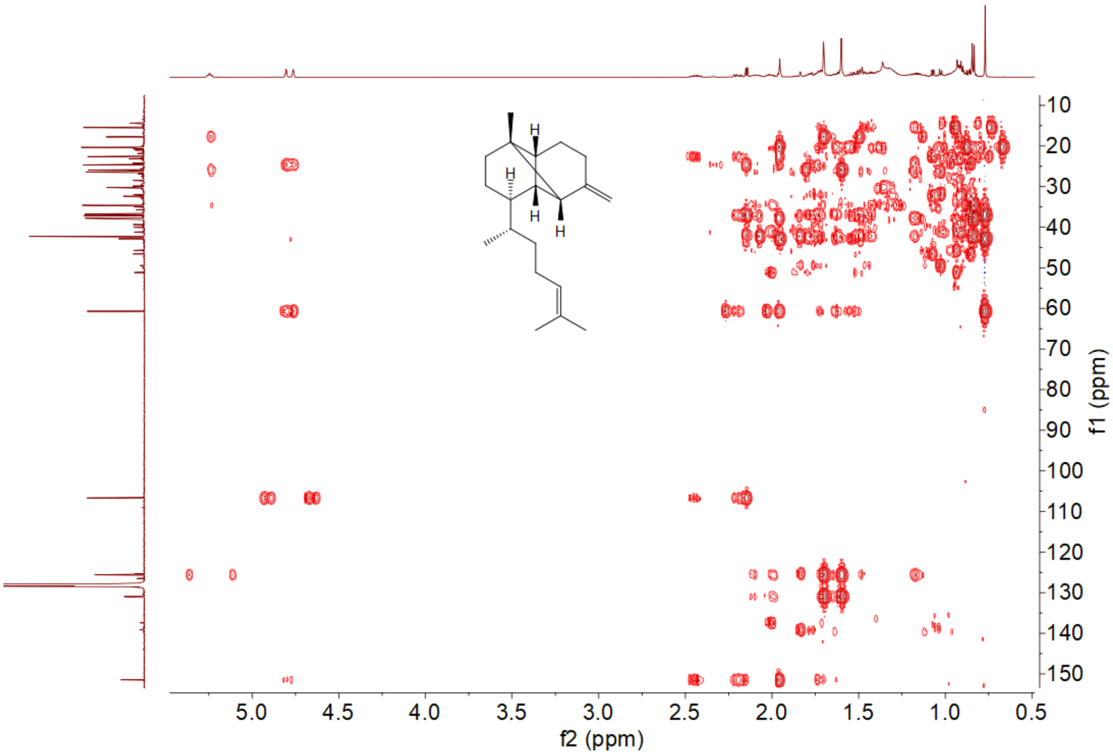

Fig. S82. HMBC NMR spectrum of 10 in C_6D_6.

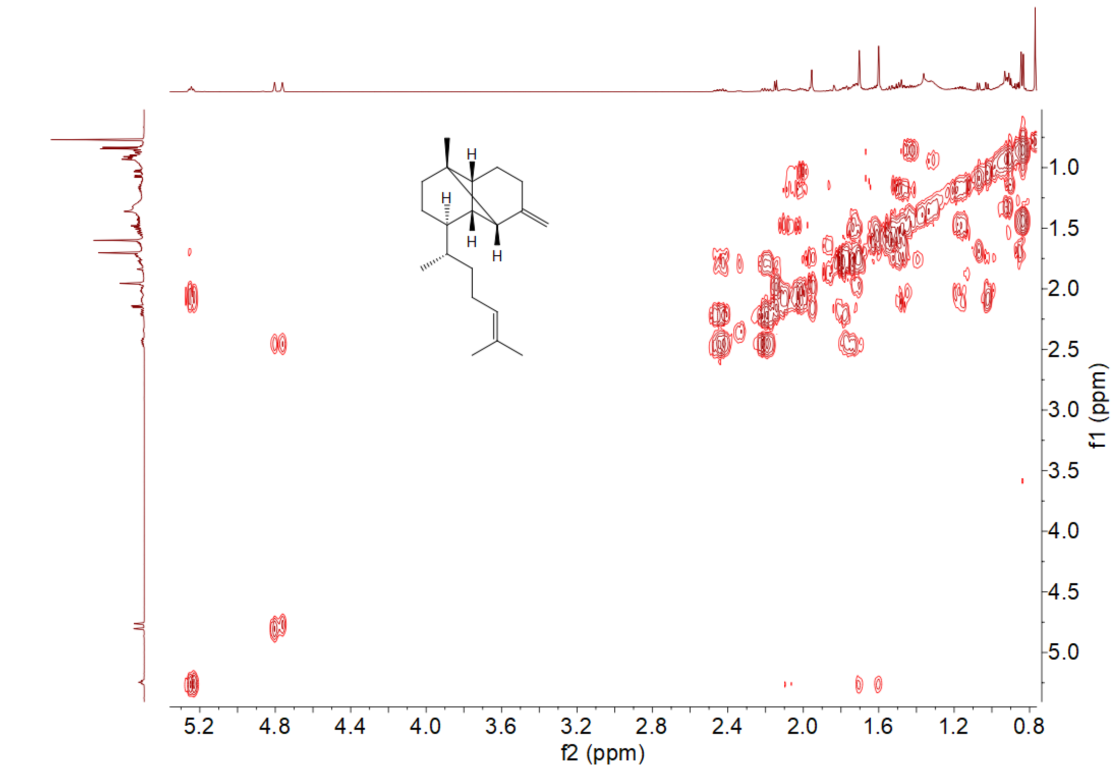

Fig. S83. ^{1}H-^{1}H COSY NMR spectrum of 10 in C_6D_6.

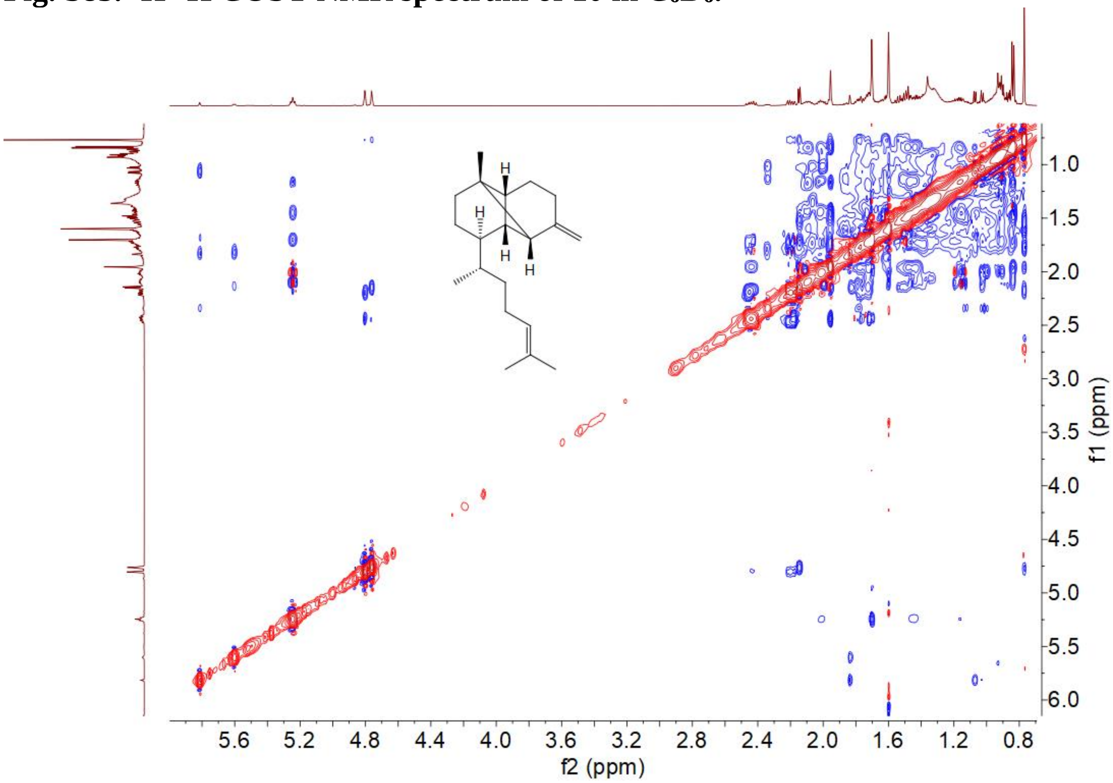

Fig. S84. NOESY NMR spectrum of 10 in C_6D_6.

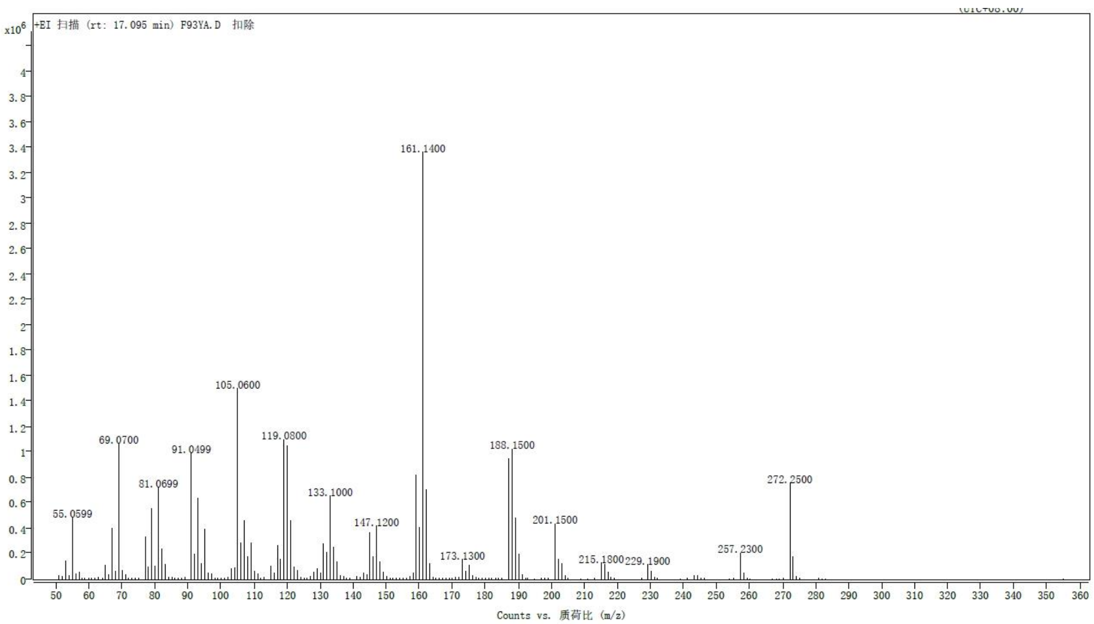

Fig. S85. The GC-MS spectra of 10.

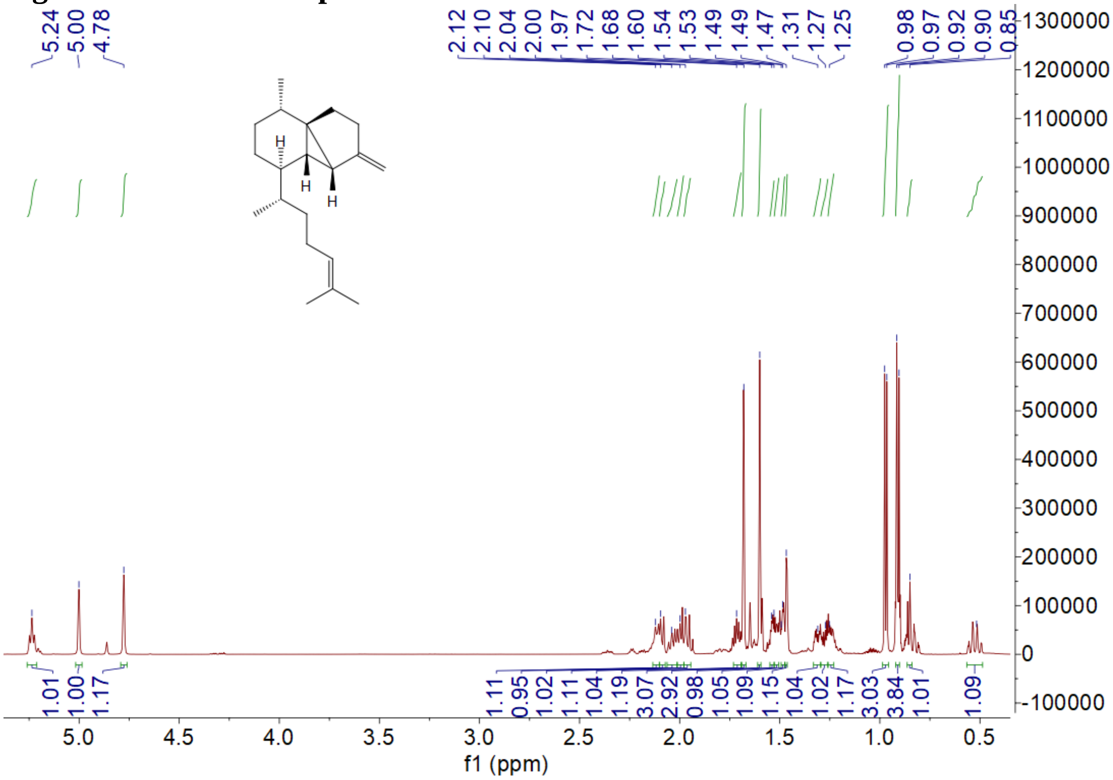

Fig. S86. ^{1}H NMR spectrum (600 MHz) of 11 in C_6D_6.

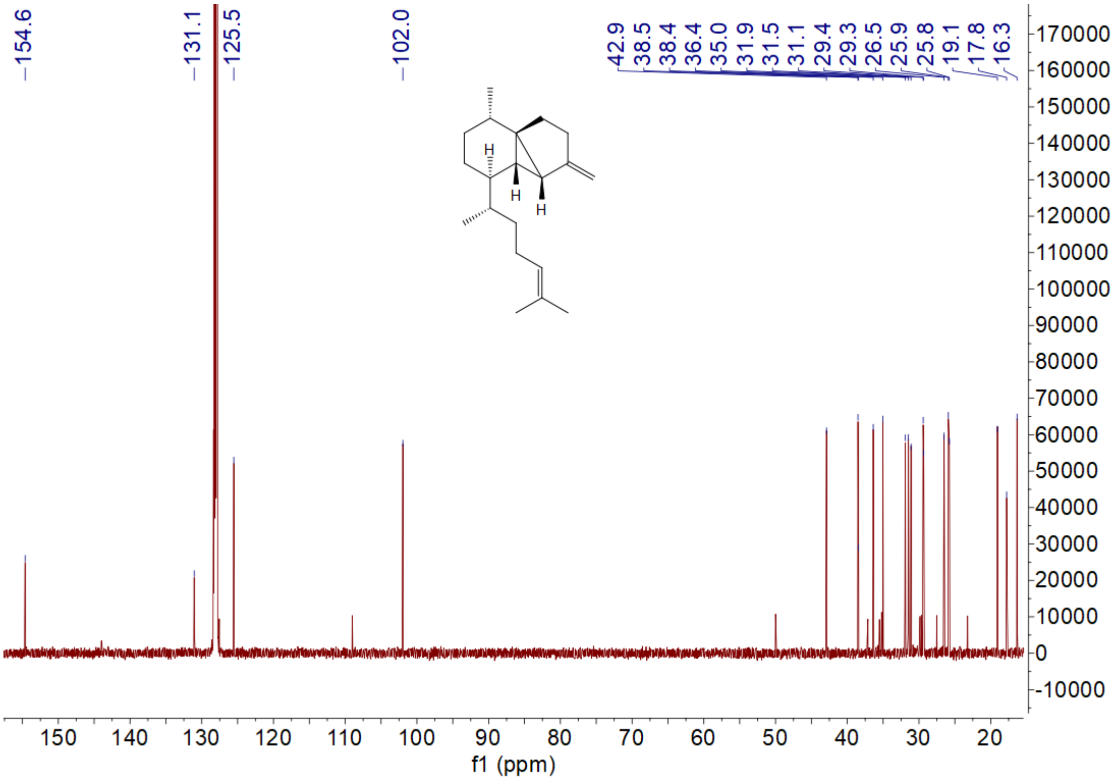

Fig. S87. ^{13}C NMR spectrum (150 MHz) of 11 in C_6D_6.

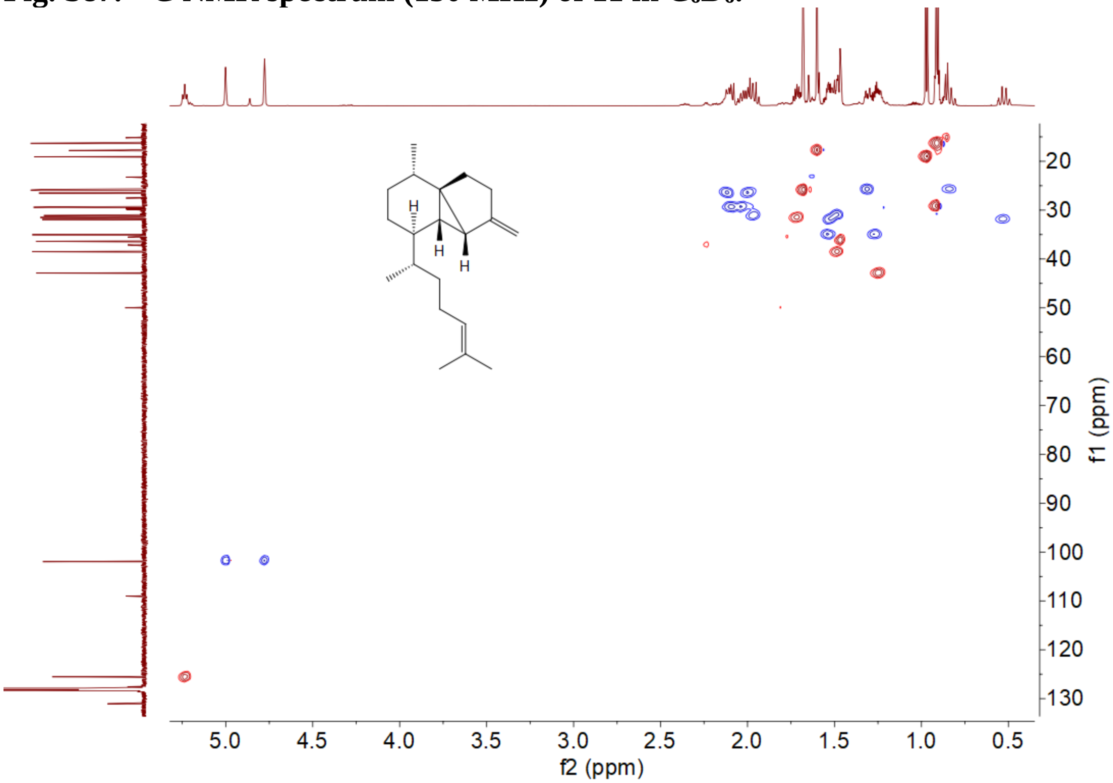

Fig. S88. HSQC NMR spectrum of 11 in C_6D_6.

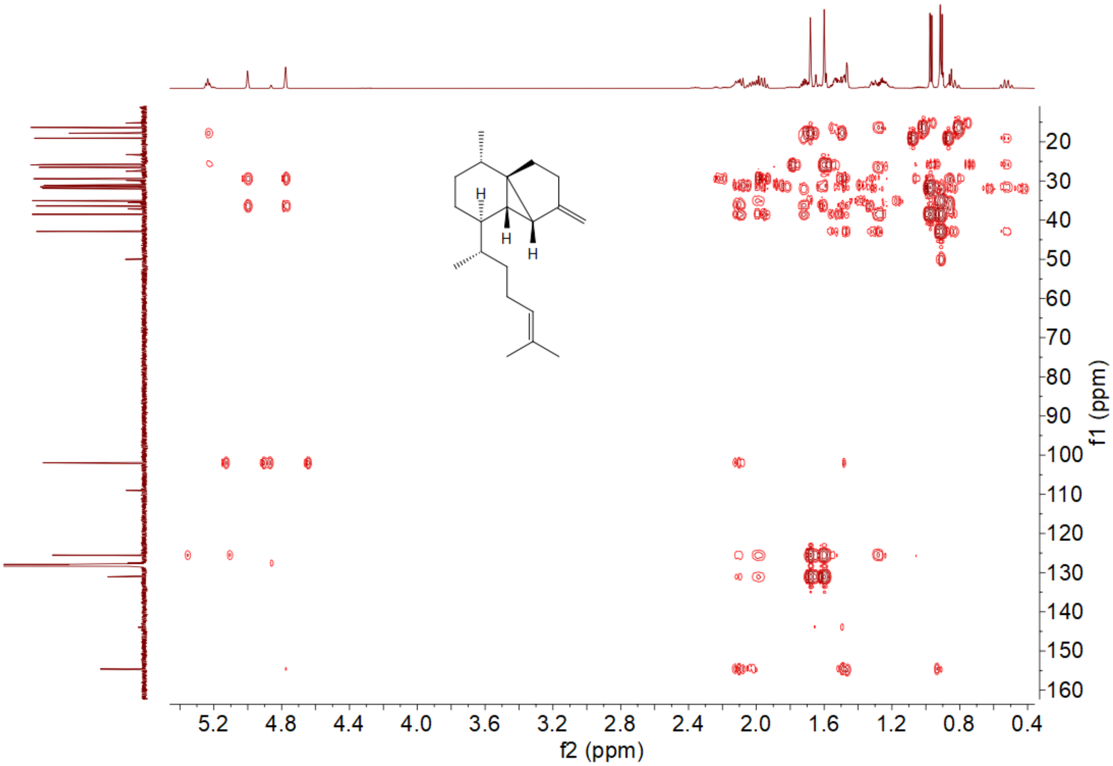

Fig. S89. HMBC NMR spectrum of 11 in C_6D_6.

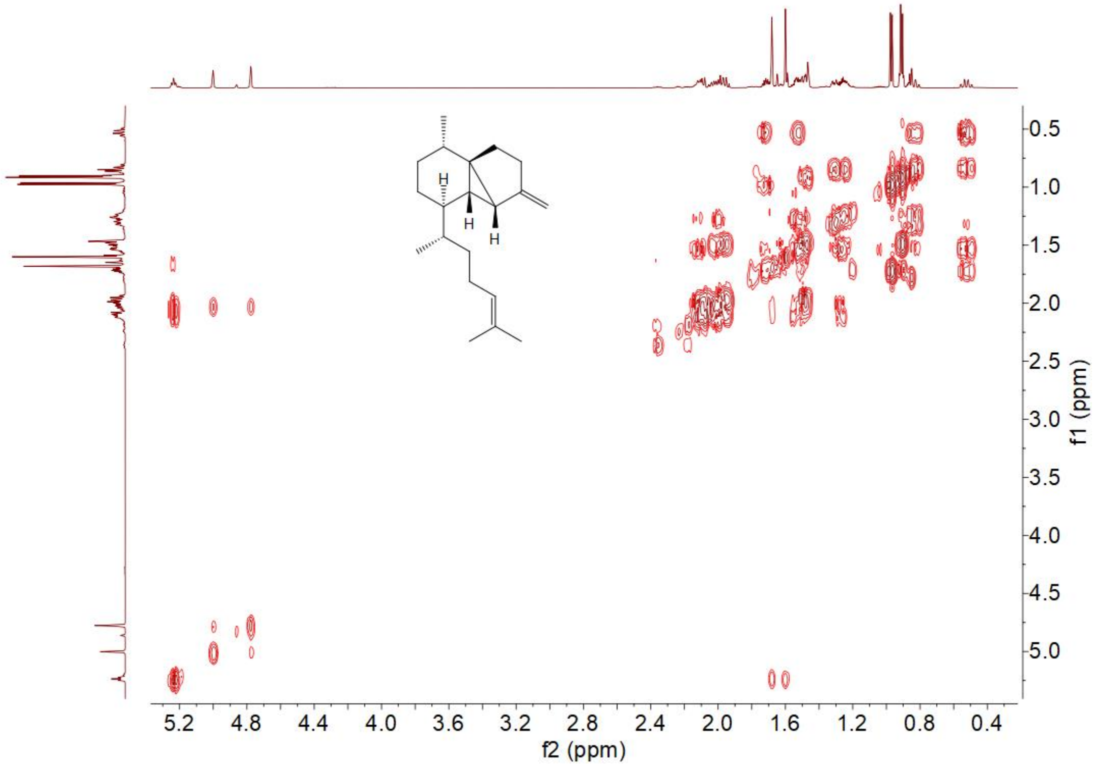

Fig. S90. ^1H-^1H COSY NMR spectrum of 11 in C_6D_6.

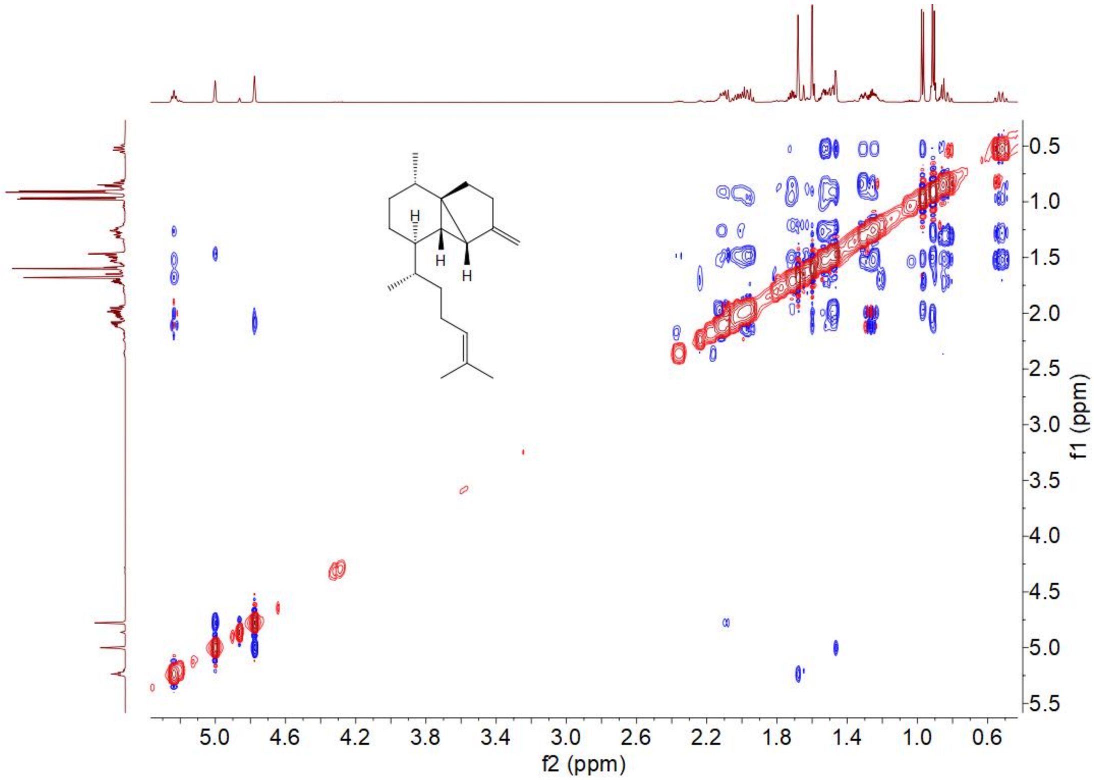

Fig. S91. NOESY NMR spectrum of 11 in C_6D_6.

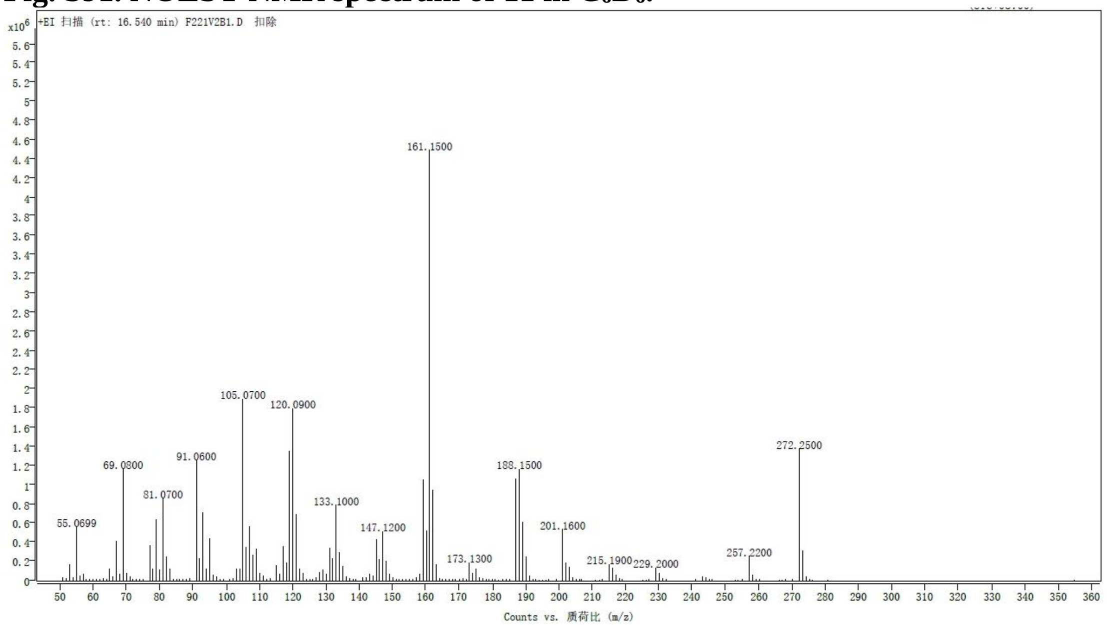

Fig. S92. The GC-MS spectra of 11.

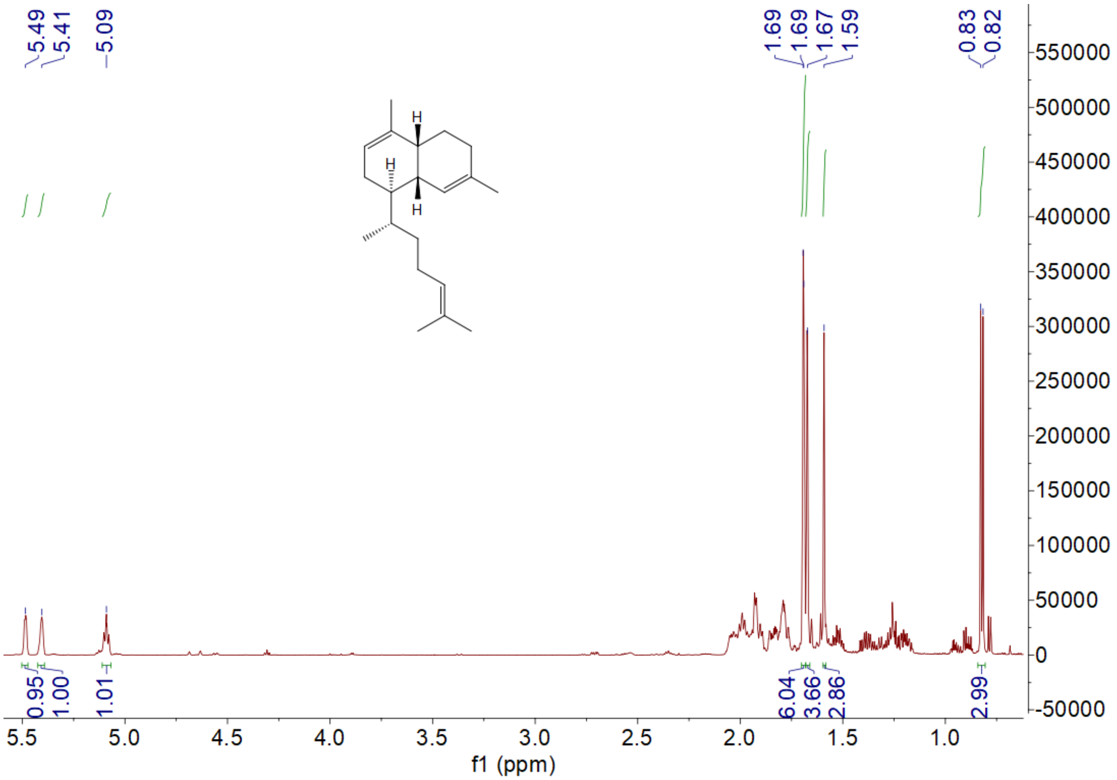

Fig. S93. ^{1}H NMR spectrum (600 MHz) of 12 in C_6D_6.

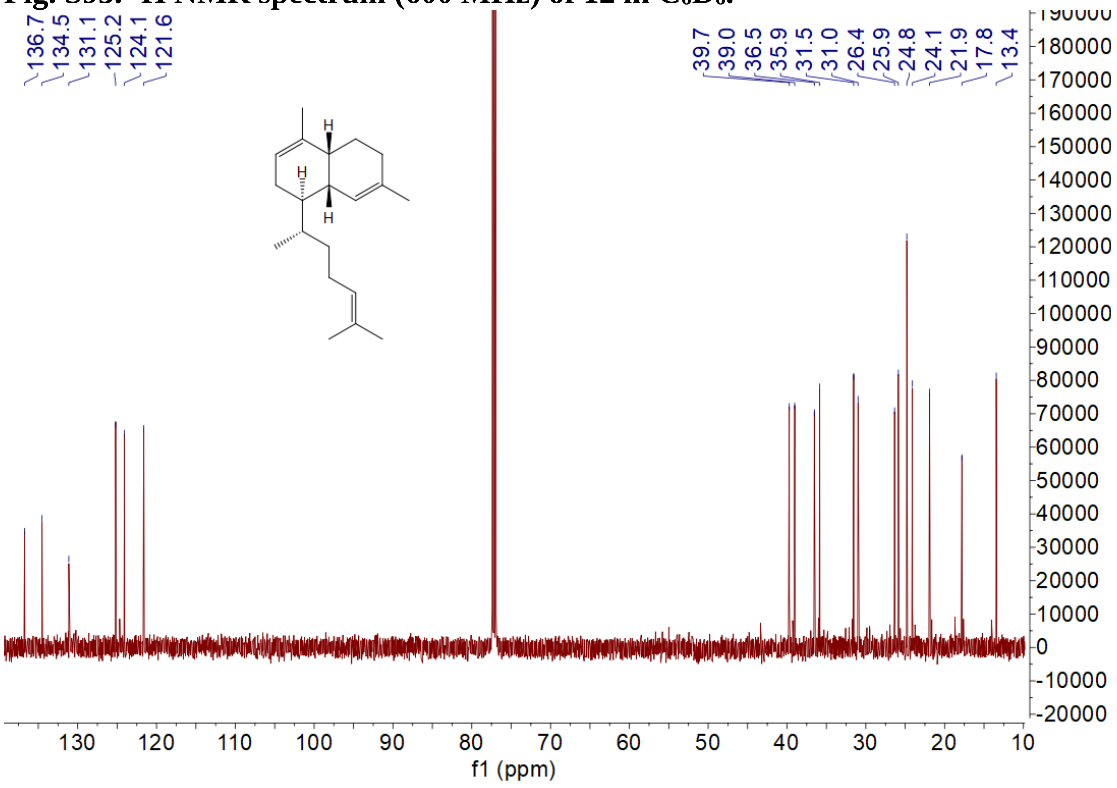

Fig. S94. ^{13}C NMR spectrum (150 MHz) of 12 in C_6D_6.

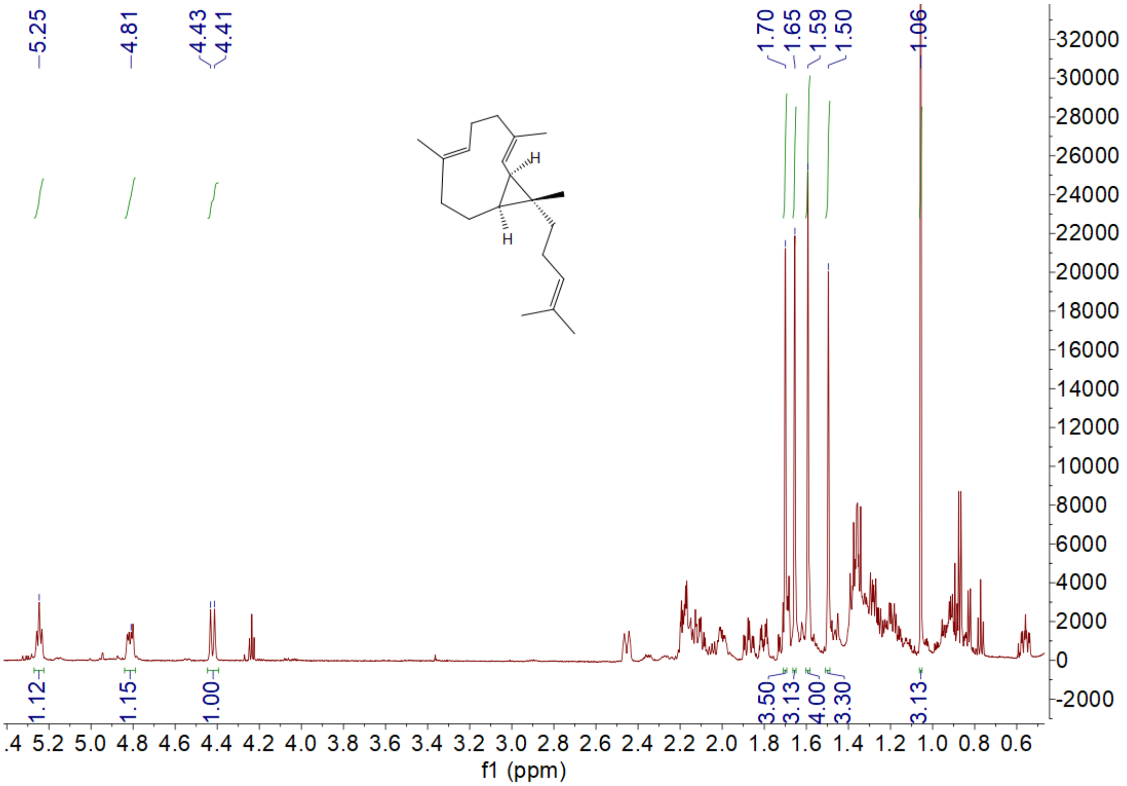

Fig. S95. ^{1}H NMR spectrum (600 MHz) of 13 in C_6D_6.

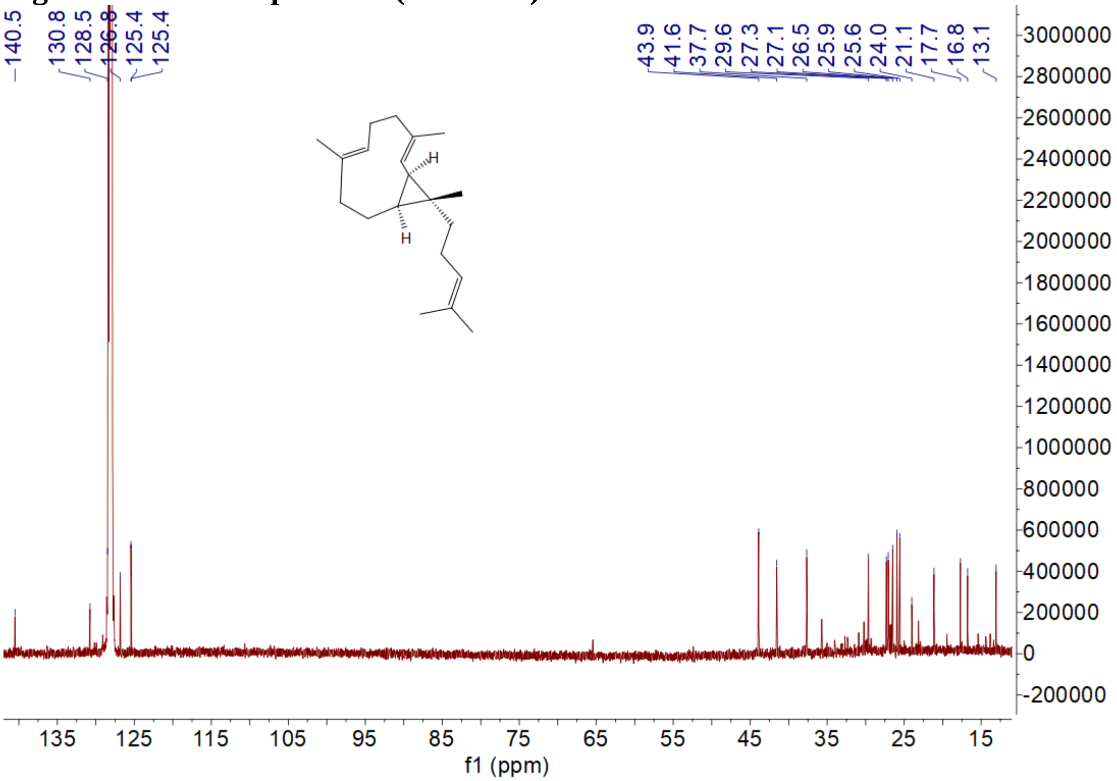

Fig. S96. ^{13}C NMR spectrum (150 MHz) of 13 in C_6D_6.

Table S1. Strains and plasmids used in this study.

Strain	Description	Source
E. coli Turbo	Host for general cloning	Shanghai Weidi Biotechnology
E. coli BL21 Gold (DE3)	Host for high-level protein production	Shanghai Weidi Biotechnology

Table S2. Plasmids used in this study.

Plasmid	Description	Source (Reference)
CDF-MKI2	Kinase-based system constructed by L.L. for GPP production (The "MKI2" encodes for four genes responsible for converting isoprenol into GPP. "MKI" expresses three genes that convert isoprenol into DMAPP, where "MKI" is an abbreviation for these three genes. “M” refers to a kinase, hydroxyethylthiazole kinase (ThiM) from *E. coli*; “K” refers to a kinase, isopentenyl phosphate kinase (ipk) from *Arabidopsis thaliana*; “I” refers to isopentenyl diphosphate isomerase (idi) from *E. coli*. All genes are under a single T7 promoter-lacO transcription/regulation module. Ribosome binding sites (rbs) were included before each gene to ensure maximum translation. The "2" signifies the capability of the recombinant strain to produce two times the isoprene units. In another word, a GPP synthase has been introduced in the “MKI” system for generating C10 polyprenyl pyrophosphate substrate.)	(*16*)
CDF-MKI3	Kinase-based system constructed by L.L. for FPP production (The "MKI3" encodes for four genes responsible for converting isoprenol into FPP, with "MKI" denoting an abbreviation for the three genes previously mentioned. The "3" signifies the capability of the recombinant strain to produce three times the isoprene units. In another word, a FPP synthase has been introduced in the “MKI” system for generating C15 polyprenyl pyrophosphate substrate.)	(*16*)
CDF-MKI4	Kinase-based system for GGPP production (gift from *Prof*. Jeffrey D. Rudolf. Previously constructed by B.X.)	(*16*)
pET28a	General plasmid for cloning and protein production	Shanghai Weidi Biotechnology
pET28a-*Pc*TS1-I59A	pET28a harboring *Pc*TS1 (I59A)	This study

pET28a-*Pc*TS1-I59L	pET28a harboring *Pc*TS1 (I59L)	This study
pET28a-*Pc*TS1-F64A	pET28a harboring *Pc*TS1 (F64A)	This study
pET28a-*Pc*TS1-F64Y	pET28a harboring *Pc*TS1 (F64Y)	This study
pET28a-*Pc*TS1-F93A	pET28a harboring *Pc*TS1 (F93A)	This study
pET28a-*Pc*TS1-F93Y	pET28a harboring *Pc*TS1 (F93Y)	This study
pET28a-*Pc*TS1-D96A	pET28a harboring *Pc*TS1 (D96A)	This study
pET28a-*Pc*TS1-D97A	pET28a harboring *Pc*TS1 (D97A)	This study
pET28a-*Pc*TS1-E100A	pET28a harboring *Pc*TS1 (E100A)	This study
pET28a-*Pc*TS1-C153M	pET28a harboring *Pc*TS1 (C153M)	This study
pET28a-*Pc*TS1-C153V	pET28a harboring *Pc*TS1 (C153V)	This study
pET28a-*Pc*TS1-C153A	pET28a harboring *Pc*TS1 (C153A)	This study
pET28a-*Pc*TS1-A189F	pET28a harboring *Pc*TS1 (A189F)	This study
pET28a-*Pc*TS1-A189Y	pET28a harboring *Pc*TS1 (A189Y)	This study
pET28a-*Pc*TS1-K196A	pET28a harboring *Pc*TS1 (K196A)	This study
pET28a-*Pc*TS1-K196D	pET28a harboring *Pc*TS1 (K196D)	This study
pET28a-*Pc*TS1-K196E	pET28a harboring *Pc*TS1 (K196E)	This study
pET28a-*Pc*TS1-K196R	pET28a harboring *Pc*TS1 (K196R)	This study
pET28a-*Pc*TS1-K196T	pET28a harboring *Pc*TS1 (K196T)	This study
pET28a-*Pc*TS1-R212A	pET28a harboring *Pc*TS1 (R212A)	This study
pET28a-*Pc*TS1-D215A	pET28a harboring *Pc*TS1 (D215A)	This study
pET28a-*Pc*TS1-G216F	pET28a harboring *Pc*TS1 (G216F)	This study
pET28a-*Pc*TS1-G216Y	pET28a harboring *Pc*TS1 (G216Y)	This study
pET28a-*Pc*TS1-G216A	pET28a harboring *Pc*TS1 (G216A)	This study
pET28a-*Pc*TS1-G216V	pET28a harboring *Pc*TS1 (G216V)	This study
pET28a-*Pc*TS1-V218A	pET28a harboring *Pc*TS1 (V218A)	This study
pET28a-*Pc*TS1-F221A	pET28a harboring *Pc*TS1 (F221A)	This study
pET28a-*Pc*TS1-F221Y	pET28a harboring *Pc*TS1 (F221Y)	This study
pET28a-*Pc*TS1-F221V	pET28a harboring *Pc*TS1 (F221V)	This study
pET28a-*Pc*TS1-F221S	pET28a harboring *Pc*TS1 (F221S)	This study
pET28a-*Pc*TS1-C253A	pET28a harboring *Pc*TS1 (C253A)	This study
pET28a-*Pc*TS1-C253M	pET28a harboring *Pc*TS1 (C253M)	This study
pET28a-*Pc*TS1-C253V	pET28a harboring *Pc*TS1 (C253V)	This study
pET28a-*Pc*TS1-C253S	pET28a harboring *Pc*TS1 (C253S)	This study
pET28a-*Pc*TS1-I254A	pET28a harboring *Pc*TS1 (I254A)	This study
pET28a-*Pc*TS1-I254M	pET28a harboring *Pc*TS1 (I254M)	This study
pET28a-*Pc*TS1-I254T	pET28a harboring *Pc*TS1 (I254T)	This study
pET28a-*Pc*TS1-I254V	pET28a harboring *Pc*TS1 (I254V)	This study
pET28a-*Pc*TS1-N257A	pET28a harboring *Pc*TS1 (N257A)	This study
pET28a-*Pc*TS1-S261A	pET28a harboring *Pc*TS1 (S261A)	This study
pET28a-*Pc*TS1-K266A	pET28a harboring *Pc*TS1 (K266A)	This study
pET28a-*Pc*TS1-S270A	pET28a harboring *Pc*TS1 (S270A)	This study
pET28a-*Pc*TS1-S270T	pET28a harboring *Pc*TS1 (S270T)	This study
pET28a-*Pc*TS1-S270C	pET28a harboring *Pc*TS1 (S270C)	This study
pET28a-*Pc*TS1-W335A	pET28a harboring *Pc*TS1 (W335A)	This study
pET28a-*Pc*TS1-S338A	pET28a harboring *Pc*TS1 (S338A)	This study
pET28a-*Pc*TS1-S338W	pET28a harboring *Pc*TS1 (S338W)	This study

pET28a-*Pc*TS1-S338I	pET28a harboring *Pc*TS1 (S338I)	This study
pET28a-*Pc*TS1-S338V	pET28a harboring *Pc*TS1 (S338V)	This study
pET28a-*Pc*TS1-H339A	pET28a harboring *Pc*TS1 (H339A)	This study
pET28a-*Pc*TS1-H339W	pET28a harboring *Pc*TS1 (H339W)	This study
pET28a-*Pc*TS1-V343A	pET28a harboring *Pc*TS1 (V343A)	This study
pET28a-*Pc*TS1-P344A	pET28a harboring *Pc*TS1 (P344A)	This study

Table S3. Primer sequences used in this study.

Primer	Nucleotide Sequence (5´–3´)	Purpose
28a-*Pc*TS1-F	CAGCAAATGGGTCGCGGATCCATGAGCTGCAGCAAAGAAAT	*Pc*TS1 mutant amplification for expression in *E. coli*
28a-*Pc*TS1-R	TCGAGTGCGGCCGCAAGCTTTTAGCTAATATCCAGCTGCG	
28a-HXL7I59A-F	TAAAAAAATGTATGATAAAGCTAACACCTATCAGTTTATGTGG	*Pc*TS1 mutagenesis for I59A
28a-HXL7I59A-R	TAAACTGATAGGTGTTAGCTTTATCATACATTTTTTTAATGC	
28a-HXL7I59L-F	TAAAAAAATGTATGATAAACTGAACACCTATCAGTTTATGTGG	*Pc*TS1 mutagenesis for I59L
28a-HXL7I59L-R	TAAACTGATAGGTGTTCAGTTTATCATACATTTTTTTAATGC	
28a-HXL7F64A-F	TAAAATTAACACCTATCAGGCTATGTGGTGCATTGTGGAT	*Pc*TS1 mutagenesis for F64A
28a-HXL7F64A-R	CAATGCACCACATAGCCTGATAGGTGTTAATTTTATCATA	
28a-HXL7F64Y-F	TAAAATTAACACCTATCAGTATATGTGGTGCATTGTGGAT	*Pc*TS1 mutagenesis for F64Y
28a-HXL7F64Y-R	CAATGCACCACATATACTGATAGGTGTTAATTTTATCATA	
28a-HXL7F93A-F	GCATTTTCTGTGCGCGCTGGCTCTGGTGGATGATGCGGTG	*Pc*TS1 mutagenesis for F93A
28a-HXL7F93A-R	GCATCATCCACCAGAGCCAGCGCGCACAGAAAATGCAGGC	
28a-HXL7F93Y-F	GCATTTTCTGTGCGCGCTGTATCTGGTGGATGATGCGGTG	*Pc*TS1 mutagenesis for F93Y
28a-HXL7F93Y-R	GCATCATCCACCAGATACAGCGCGCACAGAAAATGCAGGC	
28a-HXL7D96A-F	GCGCGCTGTTTCTGGTGGCTGATGCGGTGGAAAGCTATAG	*Pc*TS1 mutagenesis for D96A
28a-HXL7D96A-R	GCTTTCCACCGCATCAGCCACCAGAAACAGCGCGCACAG	
28a-HXL7D97A-F	GCGCTGTTTCTGGTGGATGCTGCGGTGGAAAGCTATAGCG	*Pc*TS1 mutagenesis for D97A
28a-HXL7D97A-R	TAGCTTTCCACCGCAGCATCCACCAGAAACAGCGCGCACAG	
28a-HXL7E100A-F	CTGGTGGATGATGCGGTGGCAAGCTATAGCGCGAACGAAAT	*Pc*TS1 mutagenesis for E100A
28a-HXL7E100A-R	GTTCGCGCTATAGCTTGCCACCGCATCATCCACCAGAAAC	
28a-HXL7C153A-F	GCAGCAGCATTACCTTTGCCATGCAGTATGTGAACAAAAT	*Pc*TS1 mutagenesis for C153A
28a-HXL7C153A-R	TCACATACTGCATGGCAAAGGTAATGCTGCTGCGATCAAA	

28a-HXL7C153M-F	GCAGCAGCATTACCTTTATGATGCAGTATGTGAACAAAAT	*Pc*TS1 mutagenesis for C153M
28a-HXL7C153M-R	TCACATACTGCATCATAAAGGTAATGCTGCTGCGATCAAA	
28a-HXL7C153V-F	GCAGCAGCATTACCTTTGTGATGCAGTATGTGAACAAAAT	*Pc*TS1 mutagenesis for C153V
28a-HXL7C153V-R	TCACATACTGCATCACAAAGGTAATGCTGCTGCGATCAAA	
28a-HXL7A189F-F	GAGCAACGCGATTAGCATTTTCTTTCAAGCGGTGCTGATT	*Pc*TS1 mutagenesis for A189F
28a-HXL7A189F-R	GCACCGCTTGAAAGAAAATGCTAATCGCGTTGCTCGTGCG	
28a-HXL7A189Y-F	AGCAACGCGATTAGCATTTACTTTCAAGCGGTGCTGATT	*Pc*TS1 mutagenesis for A189Y
28a-HXL7A189Y-R	GCACCGCTTGAAAGTAAATGCTAATCGCGTTGCTCGTGCG	
28a-HXL7K196A-F	CAAGCGGTGCTGATTGCAAGCAAATGCGGCAGCATTATTAC	*Pc*TS1 mutagenesis for K196A
28a-HXL7K196A-R	GCTGCCGCATTTGCTTGCAATCAGCACCGCTTGAAACGC	
28a-HXL7K196D-F	CAAGCGGTGCTGATTGACAGCAAATGCGGCAGCATTATTAC	*Pc*TS1 mutagenesis for K196D
28a-HXL7K196D-R	GCTGCCGCATTTGCTGTCAATCAGCACCGCTTGAAACGC	
28a-HXL7K196E-F	CAAGCGGTGCTGATTGAAAGCAAATGCGGCAGCATTATTAC	*Pc*TS1 mutagenesis for K196E
28a-HXL7K196E-R	GCTGCCGCATTTGCTTTCAATCAGCACCGCTTGAAACGC	
28a-HXL7K196R-F	CAAGCGGTGCTGATTAGAAGCAAATGCGGCAGCATTATTAC	*Pc*TS1 mutagenesis for K196R
28a-HXL7K196R-R	GCTGCCGCATTTGCTTCTAATCAGCACCGCTTGAAACGC	
28a-HXL7K196T-F	CAAGCGGTGCTGATTACAAGCAAATGCGGCAGCATTATTAC	*Pc*TS1 mutagenesis for K196T
28a-HXL7K196T-R	GCTGCCGCATTTGCTTGTAATCAGCACCGCTTGAAACGC	
28a-HXL7R212A-F	TGAAATGCTGTGGCGCGCCGTGTTTGATGGCCTGGTGATT	*Pc*TS1 mutagenesis for R212A
28a-HXL7R212A-R	GGCCATCAAACACGGCGCGCCACAGCATTTCATGGCTCG	
28a-HXL7D215A-F	GTGGCGCCGCGTGTTTGCTGGCCTGGTGATTCTGTTTTAT	*Pc*TS1 mutagenesis for D215A
28a-HXL7D215A-R	AGAATCACCAGGCCAGCAAACACGCGGCGCCACAGCATTTC	
28a-HXL7G216F-F	GTGGCGCCGCGTGTTTGATTTCCTGGTGATTCTGTTTTAT	*Pc*TS1 mutagenesis for G216F

28a-HXL7G216F-R	AGAATCACCAGGAAATCAAACACGCGGCGCCACAGCATTTC	
28a-HXL7G216Y-F	GTGGCGCCGCGTGTTTGATTACCTGGTGATTCTGTTTTAT	*Pc*TS1 mutagenesis for G216Y
28a-HXL7G216Y-R	AGAATCACCAGGTAATCAAACACGCGGCGCCACAGCATTTC	
28a-HXL7G216A-F	GTGGCGCCGCGTGTTTGATGCGCTGGTGATTCTGTTTTAT	*Pc*TS1 mutagenesis for G216A
28a-HXL7G216A-R	AGAATCACCAGCGCATCAAACACGCGGCGCCACAGCATTTC	
28a-HXL7G216V-F	GTGGCGCCGCGTGTTTGATGTGCTGGTGATTCTGTTTTAT	*Pc*TS1 mutagenesis for G216V
28a-HXL7G216V-R	AGAATCACCAGCACATCAAACACGCGGCGCCACAGCATTTC	
28a-HXL7V218A-F	GCGTGTTTGATGGCCTGGCGATTCTGTTTTATCAGTTTGG	*Pc*TS1 mutagenesis for V218A
28a-HXL7V218A-R	CTGATAAAACAGAATCGCCAGGCCATCAAACACGCGGCGC	
28a-HXL7F221A-F	TGGCCTGGTGATTCTGGCTTATCAGTTTGGCGAACTGATT	*Pc*TS1 mutagenesis for F221A
28a-HXL7F221A-R	CGCCAAACTGATAAGCCAGAATCACCAGGCCATCAAACAC	
28a-HXL7F221Y-F	TGGCCTGGTGATTCTGTATTATCAGTTTGGCGAACTGATT	*Pc*TS1 mutagenesis for F221Y
28a-HXL7F221Y-R	CGCCAAACTGATAATACAGAATCACCAGGCCATCAAACAC	
28a-HXL7F221V-F	TGGCCTGGTGATTCTGGTGTATCAGTTTGGCGAACTGATT	*Pc*TS1 mutagenesis for F221V
28a-HXL7F221V-R	CGCCAAACTGATACACCAGAATCACCAGGCCATCAAACAC	
28a-HXL7F221S-F	TGGCCTGGTGATTCTGAGCTATCAGTTTGGCGAACTGATT	*Pc*TS1 mutagenesis for F221S
28a-HXL7F221S-R	CGCCAAACTGATAGCTCAGAATCACCAGGCCATCAAACAC	
28a-HXL7C253A-F	TGCTGGGCTGCCTGTATGCCATTGTGATTAACGATCTGT	*Pc*TS1 mutagenesis for C253A
28a-HXL7C253A-R	GATCGTTAATCACAATGGCATACAGGCAGCCCAGCATGCG	
28a-HXL7C253M-F	TGCTGGGCTGCCTGTATATGATTGTGATTAACGATCTGT	*Pc*TS1 mutagenesis for C253M
28a-HXL7C253M-R	GATCGTTAATCACAATCATATACAGGCAGCCCAGCATGCG	
28a-HXL7C253V-F	TGCTGGGCTGCCTGTATGTCATTGTGATTAACGATCTGT	*Pc*TS1 mutagenesis for C253V
28a-HXL7C253V-R	GATCGTTAATCACAATGACATACAGGCAGCCCAGCATGCG	

28a-HXL7C253S-F	TGCTGGGCTGCCTGTATAGCATTGTG ATTAACGATCTGT	*Pc*TS1 mutagenesis for C253S
28a-HXL7C253S-R	GATCGTTAATCACAATGCTATACAGG CAGCCCAGCATGCG	
28a-HXL7I254A-F	GGGCTGCCTGTATTGCGCTGTGATTA ACGATCTGTATAGC	*Pc*TS1 mutagenesis for I254A
28a-HXL7I254A-R	CAGATCGTTAATCACAGCGCAATAC AGGCAGCCCAGCATG	
28a-HXL7I254M-F	GGGCTGCCTGTATTGCATGGTGATTA ACGATCTGTATAGC	*Pc*TS1 mutagenesis for I254M
28a-HXL7I254M-R	CAGATCGTTAATCACCATGCAATACA GGCAGCCCAGCATG	
28a-HXL7I254V-F	GGGCTGCCTGTATTGCGTTGTGATTA ACGATCTGTATAGC	*Pc*TS1 mutagenesis for I254V
28a-HXL7I254V-R	CAGATCGTTAATCACAACGCAATAC AGGCAGCCCAGCATG	
28a-*Pc*TS1I254T-F	GGGCTGCCTGTATTGCACCGTGATTA ACGATCTGTATAGC	*Pc*TS1 mutagenesis for I254T
28a-*Pc*TS1I254T-R	CAGATCGTTAATCACGGTGCAATAC AGGCAGCCCAGCATG	
28a-HXL7N257A-F	TGTATTGCATTGTGATTGCCGATCTG TATAGCTATCAGCG	*Pc*TS1 mutagenesis for N257A
28a-HXL7N257A-R	GCTATACAGATCGGCAATCACAATG CAATACAGGCAGCCC	
28a-HXL7S261A-F	GTGATTAACGATCTGTATGCCTATCA GCGCGATAAACTGG	*Pc*TS1 mutagenesis for S261A
28a-HXL7S261A-R	ATCGCGCTGATAGGCATACAGATCG TTAATCACAATGCAAT	
28a-HXL7K266A-F	TAGCTATCAGCGCGATGCACTGGCG AGCAGCGATAACATG	*Pc*TS1 mutagenesis for K266A
28a-HXL7K266A-R	CGCTGCTCGCCAGTGCATCGCGCTGA TAGCTATACAGATCG	
28a-HXL7S270A-F	GCGATAAACTGGCGAGCGCCGATAA CATGATTAAAACCTG	*Pc*TS1 mutagenesis for S270A
28a-HXL7S270A-R	GTTTTAATCATGTTATCGGCGCTCGC CAGTTTATCGCGCT	
28a-HXL7S270T-F	GCGATAAACTGGCGAGCACCGATAA CATGATTAAAACCTG	*Pc*TS1 mutagenesis for S270T
28a-HXL7S270T-R	GTTTTAATCATGTTATCGGTGCTCGC CAGTTTATCGCGCT	
28a-HXL7S270C-F	GCGATAAACTGGCGAGCTGCGATAA CATGATTAAAACCTG	*Pc*TS1 mutagenesis for S270C
28a-HXL7S270C-R	GTTTTAATCATGTTATCGCAGCTCGC CAGTTTATCGCGCT	
28a-HXL7W335A-F	CATTTACACCACCGTTGGCGCGATTC GCAGCCATACCACCG	*Pc*TS1 mutagenesis for W335A

28a-HXL7W335A-R	GTATGGCTGCGAATCGCGCCAACGG TGGTGTAAATGGTGG	
28a-HXL7S338A-F	CCGTTGGCTGGATTCGCGCCCATACC ACCGTTGTGCCGCG	*Pc*TS1 mutagenesis for S338A
28a-HXL7S338A-R	ACAACGGTGGTATGGGCGCGAATCC AGCCAACGGTGGTG	
28a-HXL7S338W-F	CCGTTGGCTGGATTCGCTGGCATACC ACCGTTGTGCCGCG	*Pc*TS1 mutagenesis for S338W
28a-HXL7S338W-R	ACAACGGTGGTATGCCAGCGAATCC AGCCAACGGTGGTG	
28a-HXL7S338I-F	CCGTTGGCTGGATTCGCATTCATACC ACCGTTGTGCCGCG	*Pc*TS1 mutagenesis for S338I
28a-HXL7S338I-R	ACAACGGTGGTATGAATGCGAATCC AGCCAACGGTGGTG	
28a-HXL7S338V-F	CCGTTGGCTGGATTCGCGTGCATACC ACCGTTGTGCCGCG	*Pc*TS1 mutagenesis for S338V
28a-HXL7S338V-R	ACAACGGTGGTATGCACGCGAATCC AGCCAACGGTGGTG	
28a-HXL7H339A-F	TGGCTGGATTCGCAGCGCTACCACCG TTGTGCCGCGCTAT	*Pc*TS1 mutagenesis for H339A
28a-HXL7H339A-R	GGCACAACGGTGGTAGCGCTGCGAA TCCAGCCAACGGTGG	
28a-HXL7H339W-F	TGGCTGGATTCGCAGCTGGACCACC GTTGTGCCGCGCTAT	*Pc*TS1 mutagenesis for H339W
28a-HXL7H339W-R	GGCACAACGGTGGTCCAGCTGCGAA TCCAGCCAACGGTGG	
28a-HXL7V343A-F	CAGCCATACCACCGTTGCGCCGCGCT ATAGCGAAAGTCAGC	*Pc*TS1 mutagenesis for V343A
28a-HXL7V343A-R	CTTTCGCTATAGCGCGGCGCAACGGT GGTATGGCTGCGAATC	
28a-HXL7P344A-F	CAGCCATACCACCGTTGTGGCGCGCT ATAGCGAAAGTCAGC	*Pc*TS1 mutagenesis for P344A
28a-HXL7P344A-R	CTTTCGCTATAGCGCGCCACAACGGT GGTATGGCTGCGAATC	

Table S4. Protein sequence of selected TSs in *P. clavata*.

*Pc*TS-1 (Uniprot No.: A0A6S7HXL7):
MSCSKEIHAPRRWVERHKQVQVLPQNAVEKLISMNELIELVIECGLCDKTSIKKMYDK<br>INTYQFMWCIVDTIPASQYAEEIFKSSLHFLCALFLVDDAVESYSANEMQDLSRSYDIL<br>EKEVCKTFPNFPSINEMKESLMHLRNPFDRSSITFCMQYVNKITAILLEEGNTPHHVVY<br>NLRRRTSNAISIAFQAVLIKSKCGSIITSHEMLWRRVFDGLVILFYQFGELISGATETAQ<br>QHITVVTELRMLGCLYCIVINDLYSYQRDKLASSDNMIKTWLLEKTVSSLSEATARCS<br>QILDAIMKYMYQRVEQCMQSNPGCPQLESLLETTIYTTVGWIRSHTTVVPRYSESQLK<br>VALVEVEERELPKWLAEKDEYGWNVVEKFVETLNDEKHKGILDALQGIADGRDQLL<br>KTQLDIS
*Pc*TS-2 (Uniprot No.: A0A7D9ETX6):
MSCSNEVRIPSNWAFLEKEVLKEQPDPELVDIDGLIKWVVECEIADENMVRKYVKIVR<br>PYYFARLLYPILPNNRLCREAFKIFLHFVIAVYRCDDRMETECDLNDMGKICNAYDKL<br>DEQLCETFPKIPTVKEMQSSLKFLSEAKLIAPVTLCMDFVNKVTCAILTHGSVSEDVVF<br>EFRRRLSNSVSIYLKAVKSEKNMTPEDSDNETLWRRIFGGGPLFLLLYVEISSFSLGKT<br>KEFIPTITEMYVVSSLCCIITNDVYSYYRETTESLIYCDSIIKVWLHNKEITTIPEAIARIT<br>NILNATVKYMFEMGKNVKIQYPNSPEVHALFEYIAYATIGWMFMHDQGSHRYRDSP<br>WRISLVDVKEIDLQQNKDSYGEDVLKTFLEMSNSKAKKIIDALRGVIAVREDLIYDNC
*Pc*TS-3 (Uniprot No.: A0A6S7GFJ4):
VRPSLDILKLTFPRTLSKSRRRARRGKSAVFYKFESRMASSKELRVPKEWNKYHPDIA<br>KESIDPELFSEDELFSWLEELDLSHNKSEVAKYVQGVRPYHLMRHQVVLLPNNALCR<br>QLFKLWTKAEVALFISDDVLETLSEVEMHQICNAIQLLDDQIREQFPRFPTIAEMKQSL<br>LQQKVDEKFIPHVIYFVDFANNVAKSIIDQGNFSKEDVNDYWRRLVVMITLYYQGVE<br>VEVKHNVGPYSEDVWTRLLSAAVMVWLHAQEIVAGSVIKNTEQVSLLNELYFLASV<br>YSMVSNDIYSYRREMRLDVSVCNMVQTIAGSKETSAETDAVIKCVEILNAVVKTMYQ<br>KIEKAKQKNPANQDLWKLLDNIGMATVGWYYFHHYSPRYDDSLWRLSIVEVENDEL<br>QEWRKGNDEEQLQEVLPLLLKCSPKAKKISDTVISGVVNMHANLLLE
*Pc*TS-4 (Uniprot No.: A0A6S7HZJ2):
MSCDNDVLVPSKWTVPCKNMLTEVEDKELIAMDELLHWVNETNLTTEEGAKIVFNK<br>LNAYFYLRCLFPVLPDDPTSVKIFQLNLHFLILGYIIDDKIENYNKDEMDELISGYKNLQ<br>NQLSETFPKFSRGDIAEDQVVKYRKRLSNAIAVYLDALLSKTKTGCEISENEMLWRRC<br>FDALALGVYMSTEVFNKTLVKNHVLPISKFYKFYSLSILFCVVINDLYSYERDKMDES<br>DSIIKVWFKRKNVTDMTTAASKVAKILNAIIQQMYLLVEEGKAQYPELSEWFESIASM<br>TVGWIYIHKTVVPRYVSSPSQVTVVEIQEKMISNWLLEKDVYGQSVVREFLENLNNPR<br>QNCCAIEYILGD
*Pc*TS-5 (Uniprot No.: A0A6S7IQD1):
MACSKELQFPKEWLKYHHDSVNEAVDPELFDEEEYFKWVVALGLCDRHTAKKYNM<br>SVRPYHLMRYMVEVIPNNSLCRELFKLWTNLTGSIFVSDDKKETFTQIEMRQLCDAFQ<br>MLDRQLCEQFPRLPTLDEMKQSLRKKVTDEKLIPQLLELMDFTNSVAKSLLQKDNFA<br>EDDVRDFWRRLCVSFALYIEACKTESSTCVVPNVEIVWRRTFLGAACPWLHILEVTSG<br>AIGKVRAHVPLINELYCLSAFHCTTVNDIYSHEKEISDGTRVTNTVRIMAESKEVSGES<br>QAALKAVQILNSITKVMYQKIEKAKEENPDNAELCTLLDNIGMATAGWYFFHHYSAR<br>YYDAQWRLTLVGVEQEELEEWRKCTDEEPLDEVKHFLQRSSPKANQISDYIISGVINIH<br>ANLLPA
*Pc*TS-6 (Uniprot No.: A0A6S7IRN7):

MVEYHHDSVNEAVDPELFDEEEYFKWVVALGLCDRHTAKKYNMSVRPYHFMRYM
VEVIPNNSLCRELFKLWTNLTGSIFVSDDKKETFTQIEMRQLCDAFQMLDRQLCEQFP
RLPTLDEMKQSLRKKVTDEKLIPQLLELMDFTNSVAKSLLQKDNFAEDDVRDFWRRL
CVSFALYIEACKTESSTCVVPNVEIVWRRTFLGAACPWLHILEVTSGAIGKVRAHVPLI
NELYCLSAFYCTTVNDIYSHEKEISDGTRVTNTVRIMAESKEVSGESQAALKAVQILNS
ITKVMYQKIEKTKEENPDNAELCTLLDNIGMATAGWYFSHHYSARYYDAQWRLTLV
GVEQEELAEWSRCTDEEPLDEVKHFLQRSSPKANQISDYIISGVINIHANLLPA

*Pc*TS-7 (Uniprot No.: A0A6S7JUP8):

MSCDYDVRVPSKWTVPHKKMLTEVEDKELFALDKLLHWVNETSLTTEEGAKIVFKK
LDAHFYLRCLFPILPDDSTSVKIFQLNLHFLILGYIIDDKIEKYNQDEINELISGYNNLKN
QVSKTFPKFPSISEMKHSLCNMKNDYSISAVATLVDYVNKTTLILLEGDVAEDKVVNY
RKRLSNAIAVYLDALLSKTKTGCEISENEMLWRRCFDALALGVYMSTEVFSKTLVKK
HVLPISEFYKFYSLSILFCVVINDLYSYERDKMDDSDSIIKVWFTQKNVTDMTTAASKV
AKILNAIIQQMYLFVEE

*Pc*TS-8 (Uniprot No.: A0A6S7GM34):

MASSKELRVPKEWNKYHPDIAKESIDPELFSEDELFSWLEELDLSHNKSEVAKYVQGV
RPYHLMRHQVVLLPNNALCRQLFKLWTKAEVALFISDDVLETLSEVEMHQICNAIQL
LDDQIREQFPRFPTIAEMKQSLLQQKVDEKFIPHVIYFVDFANNVAKSIIDQGNFSKED
VNDYWRRLVVMITLYYQGVEVEVKHNVGPYSEDVWTRLLSAAVMVWLHAQEIVAG
SVIKNTEQVSLLNELYFLASVYSMVSNDIYSYRREMRLDVSVCNMVQTIAGSKETSAE
TDAVIKCVEILNAVVKTMYQKIEKAKQKNPANQDLWKLLDNIGMATVGWYYFHHY
SPRYDDSLWRLSIVEVENDELQEWRKGNDEEQLQEVLPLLLKCSPKAKKISDTVISGV
VNMHANLLLE

*Pc*TS-9 (Uniprot No.: A0A7D9JFW3):

MACSKELRVPKEWNKYHPDIANESVDPELFSEDELFSWMEDLDLSHNKSEVAKYVQS
GKPYHLMRHQVVLLPKSALSRRLFKLWTMTVVPLFMSDDVLEALSEVEMHKICAGIQ
LLDDQIRQQFPRFPTIAEMKQSLLLQKVDEKFIPHIIYLLDFANNVAKSIIDQGNFSKED
VNYYWRRLVVTITLYFQGVEVEVKHNVCSYFENVWTRVLASGVMVYSPFQEIVAGS
VIKNTEHLSLLNELYFLATVFVMVTNDIYSYGREARLDVSKQPCSMVKTIAGCKEASA
ESDAVIKCVEILNAVVKTMYQKIEKAKQDIPANQDVCKLLDKIGIATVGWYYWHIYC
PRYDDSRWRLSIVGVENDELEEWRKGNDEERLQEVLPLLVNCSSKAKKVSDAIISGVV
NMHANLLLE

*Pc*TS-10 (Uniprot No.: A0A7D9M5R7):

MACSKELRVPKEWNKYHEDIVNESVDPELFSEDELFSWLEELDLSHNKSEVAKYVHG
VRPYHLMRHQVVLIPNNALCRQLFKLWTKTVIALFMSDDVLERLNEDEMHQICNAIQ
LLDDQIREEFPRFPTIAEMKQSLLLQKVDEKFIPHIIYFLDFANNVAKSIIDQGNFSKEDV
NDYWRRLVVMIMLYYQGVEVEDKHIVCSYSEDVLTRLLTAAAMVWLQVQELVVGY
VIKNTEHVSLLNQLYFLAVVYAMVVNDVYSYTREMNLDVGICNMVQTIAGSKEASA
ESDAVIKCVEILNAVVKTMYQKIEKAKQKNPANRDLWKLLDNIGMATVGWYYCQH
YSPRYDDSLWRLSIVDVENDELEEWRKCNDEEQLQEVLPLLINCSPKAKKISDAIIS

*Pc*TS-11 (Uniprot No.: A0A7D9EWQ8):

MASSKELRVPKEWNKYHPNIANESVDPELFSEDELFSWLEELDLSHNKSEVAKYVQG
ARPYHLMRHQVVLLPNNALCRQLFKLWTKAEVALYISDDVLETLSEVEMHQICNAIQ
LLDDQIREQFPRFPTIAEMKQSLLQQKVDEKFIPHVIYFLDFANNVAKSIIDQGNFSKEK
VNDYWRRLIVMITLYYQGVEVEVKHNVGPYSEDVWTRLLSAAGMVWLHAQEIVAG
SVIKNTEQVSLLNELYFLASVYCMISNDIYSYRREMRLDVSICNMVQTISGSKETSAES

DAVIKCVEILNAVVKTMYQKIEKAKQKNPANQDLWKLLDNIGMATVGWYYYHHYS
PRYDDSLWRLSIVEVENNELEEWRKGNDEEQLQEVLPLLLKCSAKAKKISDTVISGVV
NMHANFLLE

Table S5. ^1H (600 MHz) and ^{13}C NMR (150 MHz) data of 1 and 4 in $CDCl_3$.

NO.	1		4	
	δ_H, mult. (J, Hz)	δ_C (mult.)	δ_H, mult. (J, Hz)	δ_C (mult.)
1		144.2, C		144.3, C
2	6.03, brs	126.9, CH	6.00, s	126.8, CH
3		144.5, C		144.6, C
4a	2.37, m	29.6, CH_2	2.35, m	29.5, CH_2
4b	2.20, m		2.17, m	
5a	1.75, m	27.3, CH_2	1.72, m	27.2, CH_2
5b	1.59, m		1.55, m	
6	2.39, m	37.0, CH		36.5, CD
7	1.99, m	35.3, CH	1.96, m	35.3, CH
8a	1.84, m	29.5, CH_2	1.82, m	29.5, CH_2
8b	1.32, m		1.28, m	
9a	1.68, m	23.0, CH_2	1.65, m	23.0, CH_2
9b	1.68, m		1.65, m	
10	1.83, m	49.8, CH	1.81, m	49.7, CH
11	1.73, m	31.8, CH		31.3, CD
12a	1.40, m	34.8, CH_2	1.35, m	34.7, CH_2
12b	0.94, m		0.91, m	
13a	2.05, m	25.6, CH_2	2.02, m	25.6, CH_2
13b	1.88, m		1.86, m	
14	5.07, m	125.3, CH	5.04, m	125.2, CH
15		131.1, C		131.2, C
16	1.69, s	25.9, CH_3	1.67, s	25.9, CH_3
17	1.61, s	17.8, CH_3	1.58, s	17.8, CH_3
18	0.93, d (6.4)	17.6, CH_3	0.90, s	17.5, CH_3
19	0.91, d (7.0)	15.2, CH_3	0.88, d (7.0)	15.2, CH_3
20a	4.71, s	108.2, CH_2	4.68, s	108.1, CH_2
20b	4.66, s		4.63, s	

Table S6. ^{1}H (600 MHz) and ^{13}C NMR (150 MHz) data of 1 and elisabethatriene (*15*) in C_6D_6.

**No.**	**1**		Elisabethatriene (*15*)	
	δ_H (mult, *J* in Hz)	δ_C (mult)	δ_H (mult, *J* in Hz)	δ_C (mult)
1		144.0, C		144.0, C
2	6.14, s	127.6, CH	6.14, s	127.5, CH
3		143.9, C		143.9, C
4a	2.35, m	29.9, CH_2	2.36, ddd (14.1, 9.1, 4.6)	29.8, CH_2
4b	2.17, m		2.21-2.15, m	
5a	1.62, m	27.5, CH_2	1.63-1.58, m	27.5, CH_2
5b	1.48, m		1.53-1.44, m	
6	2.23, m	37.1, CH	2.24, q (6.0)	37.1, CH
7	1.77, m	35.5, CH	1.79-1.75, m	35.5, CH
8a	1.71, m	29.6, CH_2	1.75-1.67, m	29.6, CH_2
8b	1.20, m		1.21, ddt (12.4, 3.1, 3.1)	
9a	1.63, m	23.2, CH_2	1.67-1.69, m	23.2, CH_2
9b	1.63, m		1.67-1.69, m	
10	1.80, m	50.0, CH	1.83-1.79, m	50.0, CH
11	1.70, m	32.0, CH	1.74-1.70, m	31.9, CH
12a	1.53, m	35.2, CH_2	1.56-1.52, m	35.2, CH_2
12b	1.03, m		1.04 ddt (13.2, 9.4, 4.9)	
13a	2.12, m	25.9, CH_2	2.15-2.09 (m)	25.9, CH_2
13b	1.95, m		2.00-1.90 (m)	
14	5.20, m	125.6, CH	5.12, ddsept (7.1, 7.1, 1.5)	125.6, CH
15		131.0, C		131.0, C
16	1.65, s	25.9, CH_3	1.65, s	25.9, CH_3
17	1.59, s	17.8, CH_3	1.59, s	17.8, CH_3
18	0.90, d (6.4)	17.7, CH_3	0.90, d (6.6)	17.7, CH_3
19	0.85, d (7.0)	15.2, CH_3	0.86, s	15.2, CH_3
20a	4.86, s	109.0, CH_2	4.86, s	109.0, CH_2
20b	4.78, s		4.78, s	

Table S7. ^{1}H (600 MHz) and ^{13}C NMR (150 MHz) data of 5 and 6 in C_6D_6.

**No.**	δ_H (mult, *J* in Hz)	δ_C (mult)	δ_H (mult, *J* in Hz)	δ_C (mult)
1	6.02, ddd (16.3, 9.5, 2.3)	139.0, C	5.30, dd (15.8, 10.0)	133.6, CH
2	6.12, d (16.3)	128.9, CH	5.78, d (15.8)	136.4, CH
3		148.5, C		149.0, C
4a	2.81, m	29.4, CH_2, $J_{C, F}$, d (1.9)	2.35, m	35.0, CH_2
4b	1.90, m		2.00, m	
5a	2.22, m	31.3, CH_2, $J_{C, F}$, d (30.4)	2.35, m	29.7, CH_2
5b	2.09, m		1.96, m	
6		155.6, CF, $J_{C, F}$, d (242.3)	5.16, m	129.9, CH
7		115.5, C, $J_{C, F}$, d (16.1)		133.9, C
8a	2.91, m	27.8, CH_2, $J_{C, F}$, d (6.7)	2.23, m	41.1, CH_2
8b	1.38, m		2.23, m	
9a	1.88, m	29.5, CH_2, $J_{C, F}$, d (2.7)	1.32, m	26.2, CH_2
9b	1.50, m		1.32, m	
10	1.95, m	48.3, CH	2.01, m	52.4, CH
11	1.58, m	36.4, CH	1.30, m	37.9, CH
12a	1.63, m	35.0, CH_2	1.57, m	34.0, CH_2
12b	1.15, m		1.12, m	
13a	2.12, m	26.1, CH_2	2.13, m	26.3, CH_2
13b	1.97, m		1.97, m	
14	5.21, m	125.5, CH	5.23, m	125.6, CH
15		131.0, C		131.0, C
16	1.68, s	25.9, CH_3	1.68, s	25.9, CH_3
17	1.58, brs	17.7, CH_3	1.59, s	17.8, CH_3
18	0.91, d (6.7)	17.5, CH_3	0.91, d (6.7)	17.5, CH_3
19	1.34, d (2.4)	17.5, CH_3, $J_{C, F}$, d (2.7)	1.45, s	16.1, CH_3
20a	4.79, brs	112.8, CH_2	4.95, s	109.4, CH_2
20b	4.76, brs		4.80, s	

Table S8. ^{1}H (600 MHz) and ^{13}C NMR (150 MHz) data of 7 and 8 in $CDCl_3$.

No.	7		8	
	δ_H (mult, *J* in Hz)	δ_C (mult)	δ_H (mult, *J* in Hz)	δ_C (mult)
1		129.8, C		127.6, C
2	5.60, s	122.1, CH	6.25, s	121.0, CH
3		132.7, C		135.5, C
4a	2.03, m	28.8, CH_2	2.15, m	31.4, CH_2
4b	1.94, m		2.00, m	
5a	2.16, m	27.1, CH_2	1.61, m	27.9, CH_2
5b	2.01, m		1.41, m	
6		133.3, C	2.20, m	39.2, CH
7	2.18, m	33.8, CH	1.91, m	30.8, CH
8a	1.78, m	31.2, CH_2	1.62, m	29.9, CH_2
8b	1.14, m		1.62, m	
9a	1.59, m	20.7, CH_2	1.95, m	19.3, CH_2
9b	1.34, m		1.95, m	
10	2.11, m	40.6, CH		132.7, C
11	1.81, m	34.1, CH	2.89, m	33.2, CH
12a	1.32, m	35.4, CH_2	1.39, m	35.0, CH_2
12b	1.27, m		1.27, m	
13a	1.99, m	26.5, CH_2	2.02, m	26.8, CH_2
13b	1.99, m		1.85, m	
14	5.13, m	125.2, CH	5.09, m	125.2, CH
15		131.2, C		131.2, C
16	1.70, s	25.9, CH_3	1.67, s	25.9, CH_3
17	1.62, s	17.9, CH_3	1.56, s	17.8, CH_3
18	0.66, d (6.9)	14.8, CH_3	0.96, d (6.4)	19.6, CH_3
19	0.96, d (7.0)	19.1, CH_3	0.82, d (7.0)	12.9, CH_3
20	1.80, s	23.3, CH_3	1.77, s	24.5, CH_3

Table S9. ^{1}H (600 MHz) and ^{13}C NMR (150 MHz) data of 10 and 11 in C_6D_6.

**No**.	δ_H (mult, *J* in Hz)	δ_C (mult)	δ_H (mult, *J* in Hz)	δ_C (mult)
1	1.97, m	42.3, CH	0.92, m	29.3, CH
2	2.15, d (5.9)	60.7, CH	1.47, d (2.8)	36.4, CH
3		151.5, C		154.6, C
4a	2.46, m	24.6, CH_2	2.10, m	29.4, CH_2
4b	2.22, m		2.04, m	
5a	1.78, m	22.6, CH_2	1.97, m	31.1, CH_2
5b	1.74, m		1.49, m	
6	1.97, m	37.1, CH		38.4, C
7		42.9, C	1.72, m	31.5, CH
8a	1.62, m	36.8, CH_2	1.53, m	31.9, CH_2
8b	1.54, m		0.52, m	
9a	1.49, m	20.3, CH_2	1.31, m	25.8, CH_2
9b	1.49, m		0.85, m	
10	1.73, m	42.3, CH	1.25, m	42.9, CH
11	1.43, m	37.8, CH	1.49, m	38.5, CH
12a	1.46, m	34.6, CH_2	1.54, m	35.0, CH_2
12b	1.17, m		1.27, m	
13a	2.11, m	26.4, CH_2	2.12, m	26.5, CH_2
13b	2.03, m		2.00, m	
14	5.25, m	125.6, CH	5.24, m	125.5, CH
15		131.0, C		131.1, C
16	1.70, s	25.9, CH_3	1.68, s	25.9, CH_3
17	1.60, s	17.8, CH_3	1.60, s	17.8, CH_3
18	0.84, d (6.8)	15.5, CH_3	0.91, d (6.7)	16.3, CH_3
19	0.77, s	20.3, CH_3	0.97, d (6.5)	19.1, CH_3
20a	4.80, brs	106.7, CH_2	5.00, brs	102.0, CH_2
20b	4.76, brs		4.78, brs	

Table S10. Crystallographic data collection and refinement statistics.

	*Pc*TS1
**Data collection**	
Space group	P3221
Cell dimensions	
a, b, c (Å)	85.2837 85.2837 106.137
α, β, γ (°)	90 90 120
Resolution (Å)	73.86 - 3.501 (3.626 - 3.501)
Rmerge	0.811 (2.813)
*R*meas	0.8361 (2.915)
Rpim	0.201 (0.7578)
I / σ*I*	4.55 (1.94)
CC1/2	0.967 (0.807)
Completeness (%)	99.78 (99.65)
Redundancy	17.7 (14.8)
**Refinement**	
Resolution (Å)	73.86 - 3.501
No. reflections	5924
*R*work / *R*free	0.3424/0.3154
No. atoms	3332
Protein	3332
Ligand/ion	0
Water	0
B-factors	
Protein	53.47
Ligand/ion	-
R.m.s deviations	
Bond Lengths (Å)	0.016
Bond Angles (°)	1.66
Methods	
Favored (%)	96.56
Allowed (%)	3.44
Outliers (%)	0.00

Statistics for the highest-resolution shell are shown in parentheses.

Table S11. Raw Data of relative enzyme activities.

Plasmids	Area 1	Area 2	Area 3	Average value	Percentage value 1	Percentage value 2	Percentage value 3	Average value	Standard error
*Pc*TS1	11070	9031	9700	9933.53	111.44	90.91	97.65	100.00	10.46
D96A	4	3	0		0.04	0.03	0.00	0.02	0.02
D97A	30	32	39		0.31	0.32	0.39	0.34	0.04
E100A	20	22	26		0.20	0.22	0.26	0.23	0.03
C153A	5471	5413	5407		55.07	54.49	54.43	54.67	0.35
K196A	2937	2787	2788		29.56	28.06	28.07	28.56	0.87
K196D	8	8	12		0.08	0.08	0.12	0.09	0.02
K196E	303	305	300		3.05	3.07	3.02	3.05	0.02
K196R	6307	6137	6244		63.50	61.78	62.86	62.71	0.87
K196T	7115	6570	7120		71.62	66.14	71.67	69.81	3.18
R212A	22	22	31		0.22	0.22	0.31	0.25	0.05
D215A	19	17	22		0.19	0.17	0.22	0.19	0.03
N257A	9	11	17		0.09	0.11	0.17	0.13	0.04
S261A	9	8	15		0.09	0.08	0.15	0.11	0.04
K266A	10107	9594	10414		101.75	96.58	104.83	101.05	4.17
S270A	0	0	0		0.00	0.00	0.00	0.00	0.00
I59A	5023	4394	3989		50.57	44.23	40.16	44.99	5.24
F64A	927	785	716		9.34	7.90	7.20	8.15	1.09
F93A	27	25	36		0.27	0.25	0.36	0.29	0.06
A189F	20	23	27		0.20	0.24	0.27	0.24	0.03
A189Y	19	19	18		0.19	0.19	0.18	0.19	0.01
G216F	18	22	14		0.18	0.23	0.14	0.18	0.04
G216Y	14	19	22		0.14	0.19	0.22	0.18	0.04
V218A	2678	1807	1921		26.96	18.19	19.33	21.49	4.77
F221A	177	156	185		1.78	1.57	1.86	1.74	0.15
F221Y	3358	3840	3755		33.80	38.66	37.80	36.75	2.59
C253A	2088	1788	1897		21.02	18.00	19.10	19.37	1.53
C253M	10	12	19		0.10	0.12	0.19	0.13	0.05
C253V	11	16	22		0.11	0.16	0.22	0.16	0.05
I254A	1922	1717	1316		19.35	17.29	13.25	16.63	3.10
I254M	10	13	22		0.10	0.13	0.22	0.15	0.07
I254V	1299	1180	1701		13.08	11.88	17.12	14.03	2.74
W335A	19	22	24		0.19	0.22	0.24	0.22	0.03
S338A	11786	11522	11687		118.64	115.99	117.66	117.43	1.34
S338W	14	14	22		0.14	0.14	0.22	0.17	0.04
H339A	170	166	185		1.71	1.67	1.87	1.75	0.10
H339W	30	20	27		0.30	0.20	0.27	0.26	0.05
V343A	114	143	586		1.14	1.44	5.89	2.82	2.66
P344A	2627	2903	2594		26.45	29.22	26.11	27.26	1.71

REFERENCES AND NOTES

1. S. A. Look, W. Fenical, R. S. Jacobs, J. Clardy, The pseudopterosins: Anti-inflammatory and analgesic natural products from the sea whip *Pseudopterogorgia elisabethae*. *Proc. Natl. Acad. Sci. U.S.A.* **83**, 6238–6240 (1986).

2. H. Miyaoka, M. Shimomura, H. Kimura, Y. Yamada, H.-S. Kim, W. Yusuke, Antimalarial activity of kalihinol A and new relative diterpenoids from the Okinawan sponge, *Acanthella* sp. *Tetrahedron* **54**, 13467–13474 (1998).

3. C. W. J. Chang, A. Patra, D. M. Roll, P. J. Scheuer, G. K. Matsumoto, J. Clardy, Kalihinol-A, a highly functionalized diisocyano diterpenoid antibiotic from a sponge. *J. Am. Chem. Soc.* **106**, 4644–4646 (1984).

4. G. M. Molina-Salinas, V. M. Rivas-Galindo, S. Said-Fernández, D. C. Lankin, M. A. Muñoz, P. Joseph-Nathan, G. F. Pauli, N. Waksman, Stereochemical analysis of leubethanol, an anti-TB-active serrulatane, from *Leucophyllum frutescens*. *J. Nat. Prod.* **74**, 1842–1850 (2011).

5. T. Zeng, Z. Liu, J. Zhuang, Y. Jiang, W. He, H. Diao, N. Lv, Y. Jian, D. Liang, Y. Qiu, R. Zhang, F. Zhang, X. Tang, R. Wu, TeroKit: A database-driven web server for terpenome research. *J. Chem. Inf. Model.* **60**, 2082–2090 (2020).

6. Y. Yamada, S. Arima, T. Nagamitsu, K. Johmoto, H. Uekusa, T. Eguchi, K. Shin-ya, D. E. Cane, H. Ikeda, Novel terpenes generated by heterologous expression of bacterial terpene synthase genes in an engineered Streptomyces host. *J. Antibiot.* **68**, 385–394 (2015).

7. Y. Yamada, T. Kuzuyama, M. Komatsu, K. Shin-ya, S. Omura, D. E. Cane, H. Ikeda, Terpene synthases are widely distributed in bacteria. *Proc. Natl. Acad. Sci. U.S.A.* **112**, 857–862 (2015).

8. J. Rinkel, P. Rabe, X. Chen, T. G. Köllner, F. Chen, J. S. Dickschat, Mechanisms of the diterpene cyclases β-pinacene synthase from *Dictyostelium discoideum* and hydropyrene synthase from *Streptomyces clavuligerus*. *Chem. A Eur. J.* **23**, 10501–10505 (2017).

9. G. Li, Y.-W. Guo, J. S. Dickschat, Diterpene biosynthesis in *Catenulispora acidiphila*: On the mechanism of catenul-14-en-6-ol synthase. *Angew. Chem. Int. Ed. Engl.* **60**, 1488–1492 (2021).

10. M. Ringel, M. Reinbold, M. Hirte, M. Haack, C. Huber, W. Eisenreich, M. A. Masri, G. Schenk, L. W. Guddat, B. Loll, R. Kerr, D. Garbe, T. Brück, Towards a sustainable generation of pseudopterosin-type bioactives. *Green Chem.* **22**, 6033–6046 (2020).

11. C. G. Newton, M. S. Sherburn, Total synthesis of the pseudopterosin aglycones. *Nat. Prod. Rep.* **32**, 865–876 (2015).

12. O. Gericke, N. L. Hansen, G. B. Pedersen, L. Kjaerulff, D. Luo, D. Staerk, B. L. Møller, I. Pateraki, A. M. Heskes, Nerylneryl diphosphate is the precursor of serrulatane, viscidane and cembrane-type diterpenoids in *Eremophila* species. *BMC Plant Biol.* **20**, 91 (2020).

13. G. P. Miller, W. W. Bhat, E. R. Lanier, S. R. Johnson, D. T. Mathieu, B. Hamberger, The biosynthesis of the anti-microbial diterpenoid leubethanol in *Leucophyllum frutescens* proceeds via an all-*cis* prenyl intermediate. *Plant J.* **104**, 693–705 (2020).

14. P. D. Scesa, Z. Lin, E. W. Schmidt, Ancient defensive terpene biosynthetic gene clusters in the soft corals. *Nat. Chem. Biol.* **18**, 659–663 (2022).

15. I. Burkhardt, T. de Rond, P. Y.-T. Chen, B. S. Moore, Ancient plant-like terpene biosynthesis in corals. *Nat. Chem. Biol.* **18**, 664–669 (2022).

16. J. Li, B. Chen, Z. Fu, J. Mao, L. Liu, X. Chen, M. Zheng, C.-Y. Wang, C. Wang, Y.-W. Guo, B. Xu, Discovery of a terpene synthase synthesizing a nearly non-flexible eunicellane reveals the basis of flexibility. *Nat. Commun.* **15**, 5940 (2024).

17. A. M. Adio, C. Paul, H. Tesso, P. Kloth, W. A. König, Absolute configuration of helminthogermacrene. *Tetrahedron Asymmetry* **15**, 1631–1635 (2004).

18. J. Biard, J. Verbist, Y. Letourneux, R. Floch, Diterpene ketols with antimicrobial activity from Bifurcaria bifurcata. *Planta Med.* **40**, 288–294 (1980).

19. A. C. Huang, S. A. Kautsar, Y. J. Hong, M. H. Medema, A. D. Bond, D. J. Tantillo, A. Osbourn, Unearthing a sesterterpene biosynthetic repertoire in the Brassicaceae through genome mining reveals convergent evolution. *Proc. Natl. Acad. Sci. U.S.A.* **114**, E6005–E6014 (2017).

20. S. Himpich, M. Ringel, R. Schwartz, N. Dimos, R. Driller, C. P. O. Helmer, P. Kumar Gupta, M. Haack, D. Thomas Major, T. Brück, B. Loll, How can the diterpene synthase CotB2^{V80L} alter the product profile? *ChemCatChem* **16**, e202400711 (2024).

21. M. Xu, H. Xu, Z. Lei, B. Xing, J. S. Dickschat, D. Yang, M. Ma, Structural insights into the terpene cyclization domains of two fungal sesterterpene synthases and enzymatic engineering for sesterterpene diversification. *Angew. Chem. Int. Ed.* **63**, e202405140 (2024).

22. W. Zhang, X. Wang, G. Zhu, B. Zhu, K. Peng, T. Hsiang, L. Zhang, X. Liu, Function switch of a fungal sesterterpene synthase through molecular dynamics simulation assisted alteration of an aromatic residue cluster in the active pocket of PfNS. *Angew. Chem. Int. Ed. Engl.* **136**, e202406246 (2024).

23. J.-Y. Liu, F.-L. Lin, K. A. Taizoumbe, J.-M. Lv, Y.-H. Wang, G.-Q. Wang, G.-D. Chen, X.-S. Yao, D. Hu, H. Gao, J. S. Dickschat, A functional switch between asperfumene and fusicoccadiene synthase and entrance to asperfumene biosynthesis through a vicinal deprotonation-reprotonation process. *Angew. Chem. Int. Ed. Engl.* **63**, e202407895 (2024).

24. A. Hou, B. Goldfuss, J. S. Dickschat, Functional switch and ethyl group formation in the bacterial polytrichastrene synthase from *Chryseobacterium polytrichastri*. *Angew. Chem. Int. Ed.* **60**, 20781–20785 (2021).

25. P. E. Hansen, F. M. Nicolaisen, K. Schaumburg, Deuterium isotope effects on nuclear shielding. Directional effects and nonadditivity in acyl derivatives. *J. Am. Chem. Soc.* **108**, 625–629 (1986).

26. P. E. Hansen, F. Duus, P. Schmitt, Deuterium isotope effects on ^{13}C nuclear shielding as a measure of tautomeric equilibria. *Org. Magn. Reson.* **18**, 58–61 (1982).

27. Y. Jin, D. C. Williams, R. Croteau, R. M. Coates, Taxadiene synthase-catalyzed cyclization of 6-fluorogeranylgeranyl diphosphate to 7-fluoroverticillenes. *J. Am. Chem. Soc.* **127**, 7834–7842 (2005).

28. M. D. Bojin, D. J. Tantillo, Nonclassical carbocations as C−H hydrogen bond donors. *J. Phys. Chem. A* **110**, 4810–4816 (2006).

29. Y. J. Hong, D. J. Tantillo, C–H···π interactions as modulators of carbocation structure – Implications for terpene biosynthesis. *Chem. Sci.* **4**, 2512 (2013).

30. D. J. S. Sandbeck, D. J. Markewich, A. L. L. East, The carbocation rearrangement mechanism, clarified. *J. Org. Chem.* **81**, 1410–1415 (2016).

31. J. K. Lee, K. N. Houk, Cation-cyclization selectivity: Variable structures of protonated cyclopropanes and selectivity control by catalytic antibodies. *Angew. Chem. Int. Ed.* **36**, 1003–1005 (1997).

32. H. V. Thulasiram, H. K. Erickson, C. D. Poulter, Chimeras of two isoprenoid synthases catalyze all four coupling reactions in isoprenoid biosynthesis. *Science* **316**, 73–76 (2007).

33. J. Rinkel, L. Lauterbach, J. S. Dickschat, Spata-13,17-diene synthase—An enzyme with sesqui-, di-, and sesterterpene synthase activity from *Streptomyces xinghaiensis*. *Angew. Chem. Int. Ed.* **56**, 16385–16389 (2017).

34. J.-H. Sheu, K.-C. Hung, G.-H. Wang, C.-Y. Duh, New cytotoxic sesquiterpenes from the gorgonian *Isis hippuris*. *J. Nat. Prod.* **63**, 1603–1607 (2000).

35. K. Eljounaidi, B. A. Radzikowska, C. B. Whitehead, D. J. Taylor, S. Conde, W. Davis, A. A. Dowle, S. Langer, S. James, W. P. Unsworth, D. Ezer, T. R. Larson, B. R. Lichman, Variation of terpene alkaloids in *Daphniphyllum macropodum* across plants and tissues. *New Phytol.* **243**, 299–313 (2024).

36. I. Uchida, T. Ando, N. Fukami, K. Yoshida, M. Hashimoto, T. Tada, S. Koda, Y. Morimoto, The structure of vinigrol, a novel diterpenoid with antihypertensive and platelet aggregation-inhibitory activities. *J. Org. Chem.* **52**, 5292–5293 (1987).

37. T. T. Talele, The “Cyclopropyl Fragment” is a versatile player that frequently appears in preclinical/clinical drug molecules. *J. Med. Chem.* **59**, 8712–8756 (2016).

38. WoRMS Editorial Board, World Register of Marine Species (2024); www.marinespecies.org [accessed 16 May 2024].

39. B. J. Novak, D. Fraser, T. H. Maloney, Transforming ocean conservation: Applying the genetic rescue toolkit. *Genes* **11**, 209 (2020).

40. D. E. Cane, J. S. Oliver, P. H. M. Harrison, C. Abell, B. R. Hubbard, C. T. Kane, R. Lattman, Biosynthesis of pentalenene and pentalenolactone. *J. Am. Chem. Soc.* **112**, 4513–4524 (1990).

41. A. B. Woodside, Z. Huang, C. D. Poulter, Trisammonium geranyl diphosphate, *Organic Synth.* **66**, 211–211 (1988).

42. Z. Otwinowski, W. Minor, Processing of X-ray diffraction data collected in oscillation mode. *Methods Enzymol.* **276**, 307–326 (1997).

43. P. D. Adams, P. V. Afonine, G. Bunkóczi, V. B. Chen, I. W. Davis, N. Echols, J. J. Headd, L.-W. Hung, G. J. Kapral, R. W. Grosse-Kunstleve, A. J. McCoy, N. W. Moriarty, R. Oeffner, R. J. Read, D. C. Richardson, J. S. Richardson, T. C. Terwilliger, P. H. Zwart, *PHENIX* : A comprehensive Python-based system for macromolecular structure solution. *Acta Crystallogr. D* **66**, 213–221 (2010).

44. P. Emsley, B. Lohkamp, W. G. Scott, K. Cowtan, Features and development of *Coot*. *Acta Crystallogr. D* **66**, 486–501 (2010).

45. M. Chen, N. Al-lami, M. Janvier, E. L. D’Antonio, J. A. Faraldos, D. E. Cane, R. K. Allemann, D. W. Christianson, Mechanistic insights from the binding of substrate and carbocation intermediate analogues to aristolochene synthase. *Biochemistry* **52**, 5441–5453 (2013).

46. O. Trott, A. J. Olson, AutoDock Vina: Improving the speed and accuracy of docking with a new scoring function, efficient optimization, and multithreading. *J. Comput. Chem.* **31**, 455–461 (2010).

47. Y. Duan, C. Wu, S. Chowdhury, M. C. Lee, G. Xiong, W. Zhang, R. Yang, P. Cieplak, R. Luo, T. Lee, J. Caldwell, J. Wang, P. Kollman, A point-charge force field for molecular mechanics simulations of proteins based on condensed-phase quantum mechanical calculations. *J. Comput. Chem.* **24**, 1999–2012 (2003).

48. W. L. Jorgensen, J. Chandrasekhar, J. D. Madura, R. W. Impey, M. L. Klein, Comparison of simple potential functions for simulating liquid water. *J. Chem. Phys.* **79**, 926–935 (1983).

49. C. I. Bayly, P. Cieplak, W. Cornell, P. A. Kollman, A well-behaved electrostatic potential based method using charge restraints for deriving atomic charges: The RESP model. *J. Phys. Chem.* **97**, 10269–10280 (1993).

50. J. Wang, R. M. Wolf, J. W. Caldwell, P. A. Kollman, D. A. Case, Development and testing of a general amber force field. *J. Comput. Chem.* **25**, 1157–1174 (2004).

51. D. A. Case, H. M. Aktulga, K. Belfon, D. S. Cerutti, G. A. Cisneros, V. W. D. Cruzeiro, N. Forouzesh, T. J. Giese, A. W. Götz, H. Gohlke, S. Izadi, K. Kasavajhala, M. C. Kaymak, E. King, T. Kurtzman, T.-S. Lee, P. Li, J. Liu, T. Luchko, R. Luo, M. Manathunga, M. R. Machado, H. M. Nguyen, K. A. O'Hearn, A. V. Onufriev, F. Pan, S. Pantano, R. Qi, A. Rahnamoun, A. Risheh, S. Schott-Verdugo, A. Shajan, J. Swails, J. Wang, H. Wei, X. Wu, Y. Wu, S. Zhang, S. Zhao, Q. Zhu, T. E. Cheatham III, D. R. Roe, A. Roitberg, C. Simmerling, D. M. York, M. C. Nagan, K. M. Merz Jr, AmberTools. *J. Chem. Inf. Model.* **63**, 6183–6191 (2023).

52. J.-P. Ryckaert, G. Ciccotti, H. J. C. Berendsen, Numerical integration of the cartesian equations of motion of a system with constraints: Molecular dynamics of n-alkanes. *J. Comput. Phys.* **23**, 327–341 (1977).

53. Y. Zhang, H. Liu, W. Yang, Free energy calculation on enzyme reactions with an efficient iterative procedure to determine minimum energy paths on a combined *ab initio* QM/MM potential energy surface. *J. Chem. Phys.* **112**, 3483–3492 (2000).

54. M. J. Rothman, L. L. Lohr Jr, Analysis of an energy minimization method for locating transition states on potential energy hypersurfaces. *Chem. Phys. Lett.* **70**, 405–409 (1980).

55. Y. Zhao, D. G. Truhlar, Exploring the limit of accuracy of the global hybrid meta density functional for main-group thermochemistry, kinetics, and noncovalent interactions. *J. Chem. Theory Comput.* **4**, 1849–1868 (2008).

56. Y. Zhao, D. G. Truhlar, The M06 suite of density functionals for main group thermochemistry, thermochemical kinetics, noncovalent interactions, excited states, and transition elements: Two new functionals and systematic testing of four M06-class functionals and 12 other functionals. *Theor. Chem. Acc.* **120**, 215–241 (2008).

57. N. Chen, J. Zhou, J. Li, J. Xu, R. Wu, Concerted cyclization of lanosterol C-ring and D-ring under human oxidosqualene cyclase catalysis: An ab initio QM/MM MD study. *J. Chem. Theory Comput.* **10**, 1109–1120 (2014).

58. N. Chen, S. Wang, L. Smentek, B. A. Hess Jr, R. Wu, Biosynthetic mechanism of lanosterol: Cyclization. *Angew. Chem. Int. Ed.* **54**, 8693–8696 (2015).

59. X. Chen, Y. Zhang, J. Z. H. Zhang, An efficient approach for *ab initio* energy calculation of biopolymers. *J. Chem. Phys.* **122**, 184105 (2005).

60. Y. Zhou, S. Wang, Y. Li, Y. Zhang, Born–Oppenheimer Ab initio QM/MM molecular dynamics simulations of enzyme reactions. *Methods Enzymol.* **577**, 105–118 (2016).

61. Y. Zhang, T.-S. Lee, W. Yang, A pseudobond approach to combining quantum mechanical and molecular mechanical methods. *J. Chem. Phys.* **110**, 46–54 (1999).

62. H. J. C. Berendsen, J. P. M. Postma, W. F. van Gunsteren, A. DiNola, J. R. Haak, Molecular dynamics with coupling to an external bath. *J. Chem. Phys.* **81**, 3684–3690 (1984).

63. D. Beeman, Some multistep methods for use in molecular dynamics calculations. *J. Comput. Phys.* **20**, 130–139 (1976).

64. Y. Shao, L. F. Molnar, Y. Jung, J. Kussmann, C. Ochsenfeld, S. T. Brown, A. T. B. Gilbert, L. V. Slipchenko, S. V. Levchenko, D. P. O'Neill, R. A. Di Stasio Jr, R. C. Lochan, T. Wang, G. J. O. Beran, N. A. Besley, J. M. Herbert, C. Y. Lin, T. Van Voorhis, S. H. Chien, A. Sodt, R. P. Steele, V. A. Rassolov, P. E. Maslen, P. P. Korambath, R. D. Adamson, B. Austin, J. Baker, E. F. C. Byrd, H. Dachsel, R. J. Doerksen, A. Dreuw, B. D. Dunietz, A. D. Dutoi, T. R. Furlani, S. R. Gwaltney, A. Heyden, S. Hirata, C.-P. Hsu, G. Kedziora, R. Z. Khalliulin, P. Klunzinger, A. M. Lee, M. S. Lee, W. Liang, I. Lotan, N. Nair, B. Peters, E. I. Proynov, P. A. Pieniazek, Y. M. Rhee, J. Ritchie, E. Rosta, C. D. Sherrill, A. C. Simmonett, J. E. Subotnik, H Lee Woodcock III, W. Zhang, A. T. Bell, A. K. Chakraborty, D. M. Chipman, F. J. Keil, A. Warshel, W. J. Hehre, H. F. Schaefer III, J. Kong, A. I. Krylov, P. M. W. Gill, M. Head-Gordon, Advances in methods and algorithms in a modern quantum chemistry program package. *Phys. Chem. Chem. Phys.* **8**, 3172–3191 (2006).

65. J. A. Rackers, Z. Wang, C. Lu, M. L. Laury, L. Lagardère, M. J. Schnieders, J.-P. Piquemal, P. Ren, J. W. Ponder, Tinker 8: Software tools for molecular design. *J. Chem. Theory Comput.* **14**, 5273–5289 (2018).

66. Y.-H. Wang, H. Xu, J. Zou, X.-B. Chen, Y.-Q. Zhuang, W.-L. Liu, E. Celik, G.-D. Chen, D. Hu, H. Gao, R. Wu, P.-H. Sun, J. S. Dickschat, Catalytic role of carbonyl oxygens and water in selinadiene synthase. *Nat. Catal.* **5**, 128–135 (2022).

67. F. Zhang, N. Chen, J. Zhou, R. Wu, Protonation-dependent diphosphate cleavage in FPP cyclases and synthases. *ACS Catal.* **6**, 6918–6929 (2016).

68. H. Diao, N. Chen, K. Wang, F. Zhang, Y.-H. Wang, R. Wu, Biosynthetic mechanism of lanosterol: A completed story. *ACS Catal.* **10**, 2157–2168 (2020).

69. S. F. Boys, F. Bernardi, The calculation of small molecular interactions by the differences of separate total energies. Some procedures with reduced errors. *Mol. Phys.* **19**, 553–566 (1970).

70. A. Kohl, R. Kerr, Pseudopterosin biosynthesis: Aromatization of the diterpene cyclase product, elisabethatriene. *Mar. Drugs* **1**, 54–65 (2003).

71. X. Yan, H. Ouyang, W. Wang, J. Liu, T. Li, B. Wu, X. Yan, S. He, Antimicrobial terpenoids from South China Sea soft coral *Lemnalia* sp. *Mar. Drugs* **19**, 294 (2021).

72. H. Hirota, Y. Tomono, N. Fusetani, Terpenoids with antifouling activity against barnacle larvae from the marine sponge *Acanthella cavernosa*. *Tetrahedron* **52**, 2359–2368 (1996).
